# Comprehensive diagnostics of acute myeloid leukemia by whole transcriptome RNA sequencing

**DOI:** 10.1038/s41375-020-0762-8

**Published:** 2020-03-03

**Authors:** Wibowo Arindrarto, Daniel M. Borràs, Ruben A. L. de Groen, Redmar R. van den Berg, Irene J. Locher, Saskia A. M. E. van Diessen, Rosalie van der Holst, Edith D. van der Meijden, M. Willy Honders, Rick H. de Leeuw, Wina Verlaat, Inge Jedema, Wilma G. M. Kroes, Jeroen Knijnenburg, Tom van Wezel, Joost S. P. Vermaat, Peter J. M. Valk, Bart Janssen, Peter de Knijff, Cornelis A. M. van Bergen, Erik B. van den Akker, Peter A. C. ’t Hoen, Szymon M. Kiełbasa, Jeroen F. J. Laros, Marieke Griffioen, Hendrik Veelken

**Affiliations:** 1 Center for Computational Biology, Leiden University Medical Center, 2300RC Leiden, The Netherlands; 2 Department of Human Genetics, Leiden University Medical Center, 2300RC Leiden, The Netherlands; 3 GenomeScan B.V, 2333 BZ Leiden, The Netherlands; 4 Department of Chemical Cell Biology, Leiden University Medical Center, 2300RC Leiden, The Netherlands; 5 Department of Hematology, Leiden University Medical Center, 2300RC Leiden, The Netherlands; 6 Forensic Laboratory for DNA Research, Department of Human Genetics, Leiden University Medical Center, 2300RC Leiden, The Netherlands; 7 Department of Clinical Genetics, Leiden University Medical Center, 2300RC Leiden, The Netherlands; 8 Department of Pathology, Leiden University Medical Center, 2300RC Leiden, The Netherlands; 9 Department of Hematology, Erasmus University Medical Center, 3015CN Rotterdam, The Netherlands; 10 The Delft Bioinformatics Lab, Delft University of Technology, 2628CD Delft, The Netherlands; 11 Section of Molecular Epidemiology, Leiden University Medical Center, 2300RC Leiden, The Netherlands; 12 The Radboud Institute for Molecular Life Sciences, Radboud University Medical Center, 6525 GA Nijmegen, The Netherlands

**Keywords:** Genetic testing, Acute myeloid leukaemia

## Abstract

Acute myeloid leukemia (AML) is caused by genetic aberrations that also govern the prognosis of patients and guide risk-adapted and targeted therapy. Genetic aberrations in AML are structurally diverse and currently detected by different diagnostic assays. This study sought to establish whole transcriptome RNA sequencing as single, comprehensive, and flexible platform for AML diagnostics. We developed HAMLET (Human AML Expedited Transcriptomics) as bioinformatics pipeline for simultaneous detection of fusion genes, small variants, tandem duplications, and gene expression with all information assembled in an annotated, user-friendly output file. Whole transcriptome RNA sequencing was performed on 100 AML cases and HAMLET results were validated by reference assays and targeted resequencing. The data showed that HAMLET accurately detected all fusion genes and overexpression of *EVI1* irrespective of 3q26 aberrations. In addition, small variants in 13 genes that are often mutated in AML were called with 99.2% sensitivity and 100% specificity, and tandem duplications in *FLT3* and *KMT2A* were detected by a novel algorithm based on soft-clipped reads with 100% sensitivity and 97.1% specificity. In conclusion, HAMLET has the potential to provide accurate comprehensive diagnostic information relevant for AML classification, risk assessment and targeted therapy on a single technology platform.

## Introduction

Acute myeloid leukemia (AML) is caused by functionally complementary genetic mutations that cause uncontrolled proliferation and maturational arrest of myeloid precursor cells [1, 2]. Overt AML carry on average 13 nonsynonymous mutations [3, 4]. Acquisition of AML-initiating mutations in hematopoietic stem cells can precede AML diagnosis by decades [5–7]. The 2016 revision of the WHO classification of hematologic malignancies [8] distinguishes nine AML subtypes of clinical and prognostic importance. Genetic aberrations permit precise classification with risk assessment in 50–55% of AML cases and may guide targeted therapy. A proposed genomic classification based on an extended panel of recurrently mutated genes permits classification of 80–85% of AML cases [9].

**Fig. 1:**
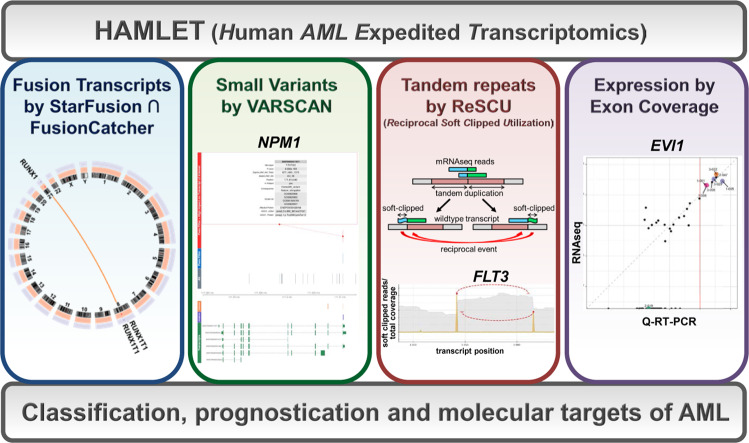
The bioinformatics pipeline HAMLET. HAMLET was developed as a bioinformatics pipeline to call all relevant information for diagnosis and prognosis of AML from raw mRNAseq data. HAMLET integrates four modules using algorithms to detect fusion genes (STARfusion ∩ FusionCatcher), small variants (VARSCAN), large tandem duplications (ReSCU), and gene expression. HAMLET permits immediate AML classification into subtypes according to WHO 2016 or genomic classifications, provides additional prognostic information according to the European Leukemia Net, and identifies molecular targets for therapy.

Accurate classification of AML, however, requires different techniques to capture structurally diverse genetic abnormalities such as larger insertions and deletions (indels), fusion genes, and structural chromosomal aberrations in addition to small variants. Moreover, expression levels of structurally normal genes can have decisive prognostic impact [10–12]. Therefore, AML diagnosis and risk assessment remains complex, expensive, and frequently incomplete with current technological platforms. To address this unmet medical need, we implemented whole transcriptome messenger RNA sequencing (mRNAseq) without concomitant sequencing of germ-line DNA as a single platform to acquire all genetic information relevant for current and future classification and risk categorization. In this diagnostic paradigm, an accredited mRNAseq protocol acquires comprehensive data on a diagnostic sample. For single-run data analysis, we developed an integrated bioinformatic pipeline designated (Human AML Expedited Transcriptomics (HAMLET); Fig. 1). HAMLET is flexibly adaptable and tailored to interrogate sequence variants and expression levels of a selected panel of genes as well as translocations from mRNAseq data.

In this report, we apply HAMLET (version 1.0) for AML classification according to the WHO 2016 [8] and genomic classifications [9] and for risk assessment according to the European Leukemia Net [1].

## Materials and methods

### AML samples

One hundred cryopreserved AML samples obtained with written informed consent were selected from the Hematology Biobank of Leiden University Medical Center (LUMC) with approval by the institutional review board (no. B 18.047). All samples had been genotyped for *NPM1* mutations and *FLT3* internal tandem duplications (*FLT3-*ITD) by the accredited LUMC molecular diagnostic laboratory.

### Whole messenger transcriptome sequencing

At least 1 µg of total RNA was isolated from mononuclear cells per sample without prior enrichment for leukemic blasts (RNAqueous kit, Thermo Fisher Scientific, Bleiswijk, The Netherlands), followed by treatment with DNAse I and silica membrane purification (RNeasy kit, Qiagen, Hilden, Germany). RNA was quantified using the Qubit RNA HS assay kit (Thermo Fisher Scientific) and diluted to 100 ng/µl. The quality of total RNA was confirmed by Fragment Analyzer (Advanced Analytical Technologies, Heidelberg, Germany). Samples with RNA quality numbers ≥6 were selected for RNA library preparation in an ISO/IEC 17025-accredited protocol (TruSeq RNA library preparation kit v2, Illumina, San Diego, CA). After enrichment of mRNA by oligo dT magnetic beads and fragmentation, cDNA synthesis was performed, followed by adapter ligation and PCR amplification. Paired-end sequencing with a read length of 126 bp was performed on an Illumina HiSeq 2500v4 sequencer to at least 12.5 Gbp per sample according to the manufacturer’s protocol, yielding ~50 million read pairs. Image analysis, base calling, and quality check was performed with Illumina data analysis pipeline RTA v1.18.64 and Bcl2fastq v1.8.4. RNAseq reads were provided in compressed Sanger FASTQ format.

### Quality parameters and alignment to human reference genome

Each input sample file was analyzed by FastQC (version 0.10.1). Known overrepresented sequences detected by FastQC were used for adapter clipping using cutadapt (version 1.2) setting the minimum accepted read length to 20 bp. Low-quality bases were then trimmed using sickle (version 1.33). Each read pair was synchronized to maintain the pair ordering using a custom Python script and were merged into a single file. Read pairs were aligned to human reference genome GRCh38 using GSNAP aligner (version 2014-12-23), setting it for novel splice sites discovery (--novel splicing 1) and unique alignments only (--npaths 1 and --quiet-if-excessive). Output SAM files were compressed, position-sorted, and indexed using Picard suite (version 1.141).

### Fusion genes

Fusion genes were detected with STAR-Fusion (version 0.5.4) and FusionCatcher (version 0.99.5a). The resulting calls were intersected using FuMa (version 3.0.5) and plotted using Circos (version 0.69). For AML classification, detected fusion transcripts were filtered for the presence of *RUNX1-RUNX1T1*, *CBFB-MYH11*, *PML-RARA*, *DEK-NUP214* gene fusions, as well as fusions involving *KMT2A*.

### Gene expression

To measure expression levels of individual exons, the total number of single read bases aligned to that exon was calculated. This number was normalized to exon length and to the total number of aligned bases for each sample. Similarly, to calculate the expression level of a gene, bases aligned to any exon of the gene were counted and normalized to the sum of gene exon lengths and to the total number of aligned bases. Expression levels were scaled to the number of single read bases aligned to 1 kbp of an exon or gene, respectively, per 10^9^ of total aligned bases.

### Gene signature for CEBPA double mutants

Gene expression data were also employed to inspect previously published signatures that distinguish between CEBPA double mutant carriers from CEBPA single mutant and wild-type carriers [13, 14]. For this purpose, normalized count data, either per gene or per exon, was filtered on minimal expression with the requirement that at least 75% of the samples should have a minimal expression of 1. Remaining expression values were voom-transformed using limma [15] for R and Z-scaled. Normalized expression levels of genes or exons indicative of CEBPA double mutation status were then plotted in a heatmap using the ggplots2 R package (https://ggplot2.tidyverse.org).

### Small variants

BAM files were processed using samtools mpileup (version 1.3.1) and VarScan (version 2.4.2) for variant calling. We disabled the VarScan strand filter (--strand-filter 0), set the minimum base frequency for variant calling to 10% of the total depth at any given position (--min-var-freq 0.1), and relaxed the p-value threshold to 0.05 (--*p* value 0.05). The resulting VCF files were then filtered with the BEDtools suite (version 2.21.0) for variants in 13 genes. The genes were selected based on a mutation frequency of >5% in cosmic AML samples and relevance for classification and prognostication: *NPM1* (Ensembl ID: ENSG00000181163), *FLT3* (ENSG00000122025), *DNMT3A* (ENSG00000119772), *ASXL1* (ENSG00000171456), *RUNX1* (ENSG00000159216), *CEBPA* (ENSG00000245848), *KIT* (ENSG00000157404), *IDH1* (ENSG00000138413), *IDH2* (ENSG00000182054), *TET2* (ENSG00000168769), *NRAS* (ENSG00000213281), *TP53* (ENSG00000141510), and *WT1* (ENSG00000184937). Since each of the 13 selected genes is mutated in >5% of AML, a panel of 100 samples was selected in order to detect genetic aberrations in each of the respective genes in at least two different samples. Gene positions are defined according to Ensembl ID throughout this manuscript. Filtered VCF files were annotated using Variant Effect Predictor (VEP version 77) and included annotation of allele frequencies from the 1000 Genomes Project [16] and the Genome of The Netherlands (GoNL) [17]. Variants were filtered based on an allele frequency of <5% in any of the subpopulations of the 1000 Genomes Project [16] and the GoNL [17] and annotated for allele frequencies and predicted effects. Based on somatic mutations in AML as reported in COSMIC, mutation hotspot regions were defined for *NPM1*, *FLT3*, *DNMT3A*, *ASXL1*, *KIT*, *IDH1*, *IDH2*, and *NRAS*. Variants are displayed using a custom R script that highlights its VEP-predicted impact on the transcript. Initial screening revealed calling of an identical *TET2* ENST00000380013.6:c.5844dup in every sample that could not be validated by RT-PCR and Sanger sequencing. Therefore, this specific variant was excluded from the analysis as sequencing artefact.

### Detection of tandem duplications in *FLT3* and *KMT2A*

To detect internal tandem duplications in *FLT3 (FLT3*-ITD) and partial tandem duplications in *KMT2A* (*KMT2A*-PTD), FASTQ reads were aligned to the *FLT3* primary gene transcript (ENST00000241453) or *KMT2A* primary gene transcript (ENST00000534358) using BWA MEM (version 0.7.13), setting the penalty of 5′ and 3′ clipping to 2 and 2 (-L 2,2), respectively. Samtools (version 1.3.1) was used to discard unaligned reads. Alignment files were then compressed, sorted, and indexed using the Picard suite (version 1.141). Soft-clipped (SC) fragments of reads partially aligning to *FLT3* exon 14–15 (1787–2024 in ENST00000241453 and 1705–1942 in coding sequence) or *KMT2A* exon 2–13 (456–4719 in ENST00000534358 and 433–4696 in coding sequence) were extracted by Fidus. SC shorter than six bases (the ceiling of log_4_(region size)) were discarded. Each SC is classified by its location at the start (sSC) or end of the aligned region (eSC). Per SC class, local alignment back to a portion of the target region was performed to find its anchor position, defined as the unique terminal position of the local alignment. Alignments with a score of <50% of the possible maximum were discarded. For a given soft clip sequence, partial ITD or PTD candidates are defined as the region enclosed by its position and its anchor position. Final ITD or PTD candidates are defined as pairs of partial ITD or PTD candidates consisting of a sSC and eSC that reciprocate each other within a maximum fuzziness value, i.e., when the anchor region of a sSC contains the soft clip position of an eSC or vice versa. Calling of definite ITD or PTD candidates identified as the region between reciprocal pairs of SC events was performed by the newly developed Reciprocal Soft Clip Utilization (ReSCU) algorithm.

### The integrated HAMLET pipeline

All bioinformatic RNAseq analyses were integrated into the HAMLET pipeline with a pdf file as primary output that displays the analyzed, filtered, and annotated results of fusion genes, small variants, *FLT3*-ITD, *KMT2A*-PTD, and *EVI1* expression in an easily interpretable graphic format. Annotations include small variants with protein consequences as determined by ENSEMBL Variant Effect Predictor, i.e., coding single nucleotide variants (SNV) and small indels. In addition, for all selected variants with protein consequences, a short summary is provided with information on variant type, protein consequence, read depth, location in hotspots, annotation in COSMIC, annotation as SNP with minor allele frequencies in different human subpopulations, and effect on protein function as predicted by PolyPhen and Sift scores.

### Validation of HAMLET output

HAMLET results were interpreted by investigators in a blinded fashion, i.e., without prior knowledge of routine interphase cytogenetics, FISH analysis, and molecular diagnostics of *NPM1* and *FLT3*.

Fusion transcripts were correlated to routine metaphase cytogenetics and FISH assays performed by the accredited diagnostic LUMC Laboratory of Clinical Genetics. In addition, fusion transcripts were extensively validated by PCR with primers flanking the breakpoints.

Sequence variants were validated by PCR with custom primers on cDNA generated from the same RNA source as for RNAseq followed by Sanger sequencing or targeted NGS. PCR amplification was performed using the PWO SuperYield DNA Polymerase kit (Roche, Mannheim, Germany) or Phusion Flash PCR kit (Thermo Fisher Scientific). PCR products were purified using Wizard SV Gel and PCR Clean-Up System kit (Promega Corporation). For targeted NGS on cDNA, libraries were prepared from PCR products by ligating barcoded TruSeq adapters (Illumina) using the KAPA Library Preparation kit (KAPA Biosystems) [18]. After pooling the libraries, sequencing was performed on the MiSeq sequencer (Illumina) using v3 sequencing reagents according to the manufacturer’s protocol.

HAMLET results for small variants were also validated by targeted NGS on genomic DNA isolated from 56 AML cases. These AML were selected for genetic features associated with an increased likelihood for mutations in *TP53*, *RUNX1*, and *TET2* according to Papaemmanuil et al. [9]. AML with complex karyotypes or −5/5q, −7/7q, −17/17p, −12/12p, or +8/8q abnormalities were selected for an increased likelihood for *TP53* mutations. AML with *KMT2A*-PTD, *ASXL1*, *DNMT3A*, *NRAS*, *TET2*, or *FLT3*-ITD mutations were selected for an increased likelihood for *RUNX1* mutations, and AML with mutations in *NPM1*, *DNMT3A*, *NRAS*, *CEBPA*, *ASXL1*, or *RUNX1* for an increased likelihood for *TET2* mutations. Using bar-coded primers for 222 amplicons, protein coding regions of *RUNX1* (8 exons), *TP53* (11 exons), *TET2* (11 exons), *KIT1* (21 exons), and *IDH1* (8 exons) were amplified as well as a large part of the *DNMT3A* gene (exons 13–23). PCR was performed on genomic DNA (14 ng) using primer pools (Thermo Fisher Scientific, Waltham, MA), the Ion AmpliSeqTM Library Kit 2.0 (Thermo Fisher Scientific) and a Bio-Rad C1000 Thermal Cycler (Bio-Rad Laboratories, Hercules, CA). Barcoded samples were and pooled and sequenced with a median depth of 1650 reads per amplicon on the Ion GeneStudio S5 System (Thermo Fisher Scientific). Sequencing data were aligned with the GRCh37 genome using TMAP 5.0.7 software and variants were called by the Torrent Variant Caller (Thermo Fisher Scientific). Detected variants after alignment to GRCh37 were converted to corresponding positions in GRCh38 to the enable validation of variants called by HAMLET. Variants with allele frequencies <10% were excluded from the analysis.

Expression of *EVI1* was validated by quantitative RT-PCR performed in the accredited diagnostic laboratory of Erasmus Medical Center [11]. *EVI1* expression relative to the *PBGD* housekeeping gene was calculated using the delta-delta CT method. Levels >0.1 relative to the EVI1-overexpressing cell line SKOV3 were defined as positive.

### Data deposition

Raw data files are available under controlled access in the European Genome-phenome Archive under accession numbers EGAC00001000956 (DAC), EGAS00001003096 (study), and EGAD00001004187 (dataset).

## Results

Accredited mRNAseq on 100 AML samples (96 diagnostic pre-treatment samples, three longitudinal samples of relapses, one longitudinal sample of a presumed therapy-related AML; Tables 1 and SI) yielded 48–85 million read pairs (97–170 million reads) per case with a median insert size (distance between the 5′ termini of the paired reads) of 149–177 bases (Table SII). The average read length after quality control was 123–124 bases.

**Table 1:** Characteristics of AML samples analyzed by RNAseq.

Characteristics	No. of AML
Number of patients	100
Age (median, years)	53 (22–77)
Gender	
Male	55
Female	45
WHO diagnosis	
AML with t(9;11); *KMT2A-MLLT3*^a^	4
AML with inv(16); *CBFB-MYH11*^a^	10
AML with t(8;21); *RUNX1-RUNX1T1*^a^	3
Acute promyelocytic leukemia with t(15;17); *PML-RARA*^*a*^	2
AML with inv(3); *GATA2, MECOM*^a^	2
AML with t(6;9); *DEK-NUP214*	1
AML with mutated *NPM1*^b^	35
Acute monoblastic/monocytic leukemia	11
AML with myelodysplasia-related changes	13
AML NOS with maturati	9
AML NOS with minimal differentiation	1
AML NOS without maturation	12
Acute myelomonocytic leukemia	3
Therapy-related AML	2
AML	
Primary	95
Relapse	5
Source	
Bone marrow (BM)	78
Peripheral blood (PB)	22
Blast percentage (median)	75 (13–99)
Karyotype^c^	
Normal karyotype	45
Complex karyotype	8
Abnormal karyotype	37
No metaphases	2
FLT3-*ITD*^b^	34

^a^Recurrent fusion transcripts *CBFB-MYH11* inv(16)(p13q22), *KMT2A-MLLT3* t(9;11)(p21;q23), *RUNX1-RUNX1T1* t(8;21)(q22;q22), and *PML-RARA* t(15;17)(q24;q21) as well as chromosomal translocation *GATA2, MECOM* inv(3)(q21q26) were detected by fluorescence in situ hybridization (FISH) on 200 interphases per probe set.

^b^Genotyping for *NPM1* and FLT3-*ITD* mutations was performed by routine diagnostic PCR on genomic DNA followed by electrophoretic fragment size analysis.

^c^Karyotype was determined by cytogenetics on at least 20 metaphases per case.

### Detection of fusion genes

**Fig. 2:**
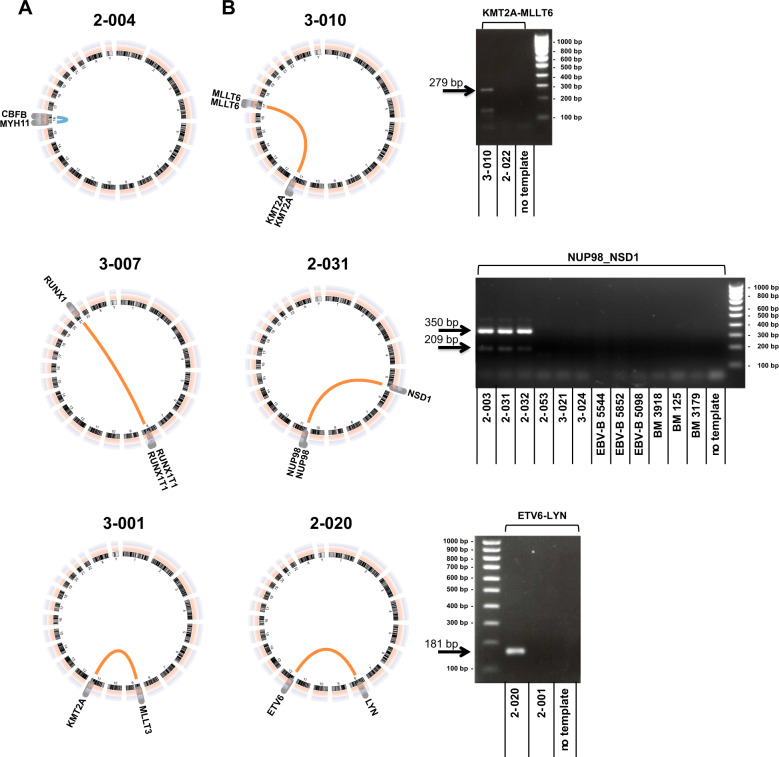
Detection of fusion genes by HAMLET. **a** Detection of fusion genes with prognostic relevance for AML included in the WHO 2016 classification. Examples shown: *CBFB-MYH11* of the inv(16)(p13q22) (case 2-004), *RUNX1-RUNX1T1* of the t(8;21)(q22;q22) (case 3-007), and *KMT2A-MLLT3* of the t(9;11)(p21;q23) (case 3-001). **b** Detection of fusion genes with prognostic relevance for AML not included in the WHO 2016 classification. Examples shown: *KMT2A-MLLT6* of the t(11;17)(q23;q21) (case 3-010), *NUP98-NSD1* of the cryptic t(5;11)(q35;p15.5) (case 2-031 with normal karyotype), and *ETV6-LYN* of a cryptic ins(12;8)(p13;q11q21) in a case (2-020) with add(8)(q24) and del(12)(p13). Gels adjacent to the circus plots depict validation by RT-PCR with custom primers (Table SVI). An amplicon of 279 bp demonstrated fusion of *KMT2A* exon 7 to *MLLT6* intron 10 (case 3-010). Amplicons of 209 and 350 bp demonstrated fusion of *NUP98* exon 11 or 12 to *NSD1* exon 7 (cases 2-003, 2-031, 2-032). An amplicon of 181 bp demonstrated fusion of *ETV6* exon 5 to *LYN* exon 8 (case 2-020). All fusion transcripts were validated by sequencing (Tables SIII and SV).

HAMLET accurately detected all recurrent fusion genes that were predicted by metaphase cytogenetics in 24 cases (Fig. 2a, Tables SIII and SIV). In three additional cases with translocations involving 11q23 other than t(9;11)(p21;q23), HAMLET correctly identified the *KMT2A* fusion partners *MLLT1*, *MLLT4*, and *MLLT6* in accordance with respective balanced translocations (Figs. 2b and S1). In addition, HAMLET correctly identified fusion transcripts in eight cases that were not predicted by metaphase karyotyping but carry known prognostic relevance for AML (Fig. 2b, Table SV). In one case (2-038), the *FUS-ERG* fusion event was detected whose corresponding t(16;21)(p11;q22) was only identified retrospectively within its complex karyotype. The karyotype of one AML (2-020) that carried the *ETV6-LYN* fusion gene had an add(8)(q24) and del(12)(p13) but lacked the characteristic ins(12;8)(p13;q11q21) [19, 20]. Three AML (2-003, 2-031, 2-032) expressed *NUP98-NSD1* fusion transcripts. Due to telomeric positions of both participating genes, the *NUP98-NSD1* originates from a cryptic t(5;11)(q35;p15.5) and is therefore currently neglected by AML classifications despite its association with poor prognosis [21–23]. *PIM3-SCO2* is an important example of an intrachromosomal fusion event that was found in three cases (2-053, 3-021, 3-024). It has previously been found in pediatric AML and can originate from a 0.6 Mb chromosome 22 inversion in chronic neutrophilic leukemia [24].

HAMLET provided detailed structural information on fusion transcripts, including differential splicing (*KMT2A-MLLT3, RUNX1-RUNX1T1, PML-RARA, CBFB-MYH11, and NUP98-NSD1*) and reciprocal transcripts from balanced translocation, e.g., *RARA-PML*, *ERG-FUS*, *NUP214-DEK*, and *NSD1-NUP98* (Table SIII). In total, HAMLET detected 54 different fusions transcripts that were not predicted by metaphase karyotyping (Tables SV and SVI, Fig. S2). Overall, 28 AML carried two or more fusions with a maximum of nine different fusions in an AML with complex karyotype.

### Detection of small variants

The VARSCAN component of HAMLET identified 246 small variants in the selected 13 AML genes with 100% specificity (Tables 2, SVII, and SVIII). The sensitivity of HAMLET was assessed for *FLT3*-ITD and hotspot regions of *NPM1* and *DNMT3A* in all 100 cases and for the remaining gene regions a total of 418 targeted resequencing reactions on samples that were called as wild type by HAMLET (Table 2). In accordance with the well-known limitation of regular variant callers to detect longer insertions [9], VARSCAN identified only seven rather short *FLT3*-ITD of the 34 *FLT3*-ITD-carrying AML. Furthermore, HAMLET failed to call a 23 bp *ASXL1* deletion (c.1900_1922del; case 3-030) and a 30 bp C-terminal *CEBPA* insertion (c.936_937ins30; case 3-003). In both cases, HAMLET correctly identified a canonical C-terminal insertion in *ASXL1* (3-030) and a N-terminal frameshift mutation in *CEBPA* (3-003). The overall type (coding SNVs and in-frame or frameshift indels) and distribution of the small variants as detected by HAMLET corresponded well with their distribution as reported in COSMIC (Fig. S3).

**Table 2:** Validation of sequence variants in 13 selected AML genes as detected by HAMLET.

Gene	Region	HAMLET component	HAMLET:	mut	wt	mut	wt	Sensitivity (%)	Specificity (%)
			Validation:	mut	mut	wt	wt		
NPM1	Hotspot	VARSCAN		35	0	0	65	100	100
DNMT3A	Hotspot	VARSCAN		15	0	0	85	100	100
	Outside hotspot	VARSCAN		17	0	0	17	100	100
FLT3	Hotspot ex20	VARSCAN		8	0	0	17	100	100
	Outside hotspot	VARSCAN		12	0	0	13	100	100
ASXL1	Hotspot	VARSCAN		13	1	0	18	92.9	100
CEBPA	Entire gene	VARSCAN		18	1	0	18	94.7	100
IDH1	Hotspot	VARSCAN		7	0	0	14	100	100
	Outside hotspot	VARSCAN		7	0	0	16	100	100
IDH2	Hotspot	VARSCAN		12	0	0	12	100	100
	Outside hotspot	VARSCAN		4	0	0	22	100	100
KIT	Hotspot	VARSCAN		7	0	0	13	100	100
	Outside hotspot	VARSCAN		3	0	0	8	100	100
NRAS	Hotspot	VARSCAN		14	0	0	15	100	100
RUNX1	Entire gene	VARSCAN		21	0	0	28	100	100
TET2	Entire gene	VARSCAN		34	0	0	34	100	100
TP53	Entire gene	VARSCAN		3	0	0	12	100	100
WT1	Entire gene	VARSCAN		9	0	0	11	100	100
TOTAL		**VARSCAN**		**239**	**2**	**0**	**418**	**99.2**	**100**
FLT3	Hotspot ex14-15	ReSCU		34	0	2	64	100	97.0
KMT2A	Hotspot ex2-13	ReSCU		8	0	0	4	100	100
TOTAL		**ReSCU**		**42**	**0**	**2**	**68**	**100**	**97.1**
TOTAL	**VARSCAN and ReSCU**			**281**	**2**	**2**	**486**	**99.3**	**99.6**

Validation of *NPM1* mutations and *FLT3*-ITD was performed by PCR on genomic DNA and electrophoretic fragment size analysis. Validation of other variants was performed by RT-PCR on cDNA followed by Sanger sequencing or NGS using MiSeq. Gene hotspots are defined in Table SVI.

*mut* variant detected, *wt* wild type.

Variants called by HAMLET that are listed in COSMIC and located within defined genetic hotspots (Table SIX) are readily interpretable as pathogenic class A variants (*n* = 114; 46.3%) (Table SVII). Sixty variants (24.4%) were reported in COSMIC but not located in hotspots. Most of these class B variants were detected in genes where mutations are known to occur throughout the entire coding sequence (*CEBPA*, *TET2, RUNX1*, *WT1, and TP53*). The remaining variants were unreported (class C variants: *n* = 52; 21.1%) or not listed in COSMIC but described as SNP (class D variants, *n* = 20; 8.1%). Class D variants probably represent true genetic polymorphisms with low allele frequency. Class C variants can be interpreted based on their predicted pathogenic effects. According with these principles for interpretation, more than 90% of class A variants, app. 50% of class B and C variants, and <1% of class D variants have been reported as recurrent mutations in myeloid malignancies by Jaiswal et al. [25] (Table SVII).

In the TCGA AML cohort [3], small variants that are present in genomic DNA have been shown to be absent in the corresponding RNA sequence analysis. In particular nonsense variants and frameshift variants with a premature stop codon may be poorly expressed as a result of nonsense-mediated RNA decay [26, 27]. We therefore validated HAMLET results for small variants in *RUNX1*, *TET2*, *TP53*, *DNMT3A*, *KIT1*, and *IDH1* by targeted NGS on genomic DNA isolated from 56 AML. These cases were selected for genetic features associated with an increased likelihood for mutations in *TP53*, *RUNX1*, and *TET2* according to Papaemmanuil et al. [9]. In these 56 AML cases, HAMLET detected 67 variants including 18 nonsense and frameshift variants that are potential targets for nonsense-mediated RNA decay. Of these 67 variants, one *TET2* variant (c.4317dup) in three AML cases was missed by targeted NGS (Table SX). This A insertion occurs in a stretch of 6 other A nucleotides, which is apparently difficult to sequence on the Ion GeneStudio S5 System. Reads for this variant were present in the raw data with allele frequencies of 2–3%. There were also two variants detected by targeted NGS that were missed by HAMLET. These variants included a *RUNX1* missense variant which has been detected by targeted NGS with a low allele frequency of 10% and a polymorphism in *IDH1*. In conclusion, our data showed that HAMLET accurately called variants in *TET2*, *TP53*, *RUNX1*, *KIT*, *IDH1*, and *DNMT3A* including all variants encoded by transcripts that are potential targets for nonsense-mediated decay. We also determined the sequencing depth that is required to detect small variants by HAMLET-RNAseq and ran the pipeline on 20, 30, 40, and 47.5 million read pairs for each of the 100 AML. Of the 246 variants called by HAMLET, 53 variants were nonsense mutations or frameshift mutations with a premature stop. The sensitivity of HAMLET to detect these variants decreased from 100, 96, 85 to 70%, while the decrease for the remaining variants was from 100, 98, 96 to 81%. These data show that a high sequencing depth of ~50 million read pairs is essential to ensure detection of all variant types including potential targets for nonsense-mediated RNA decay (Table SXI and Fig. S4).

**Fig. 3:**
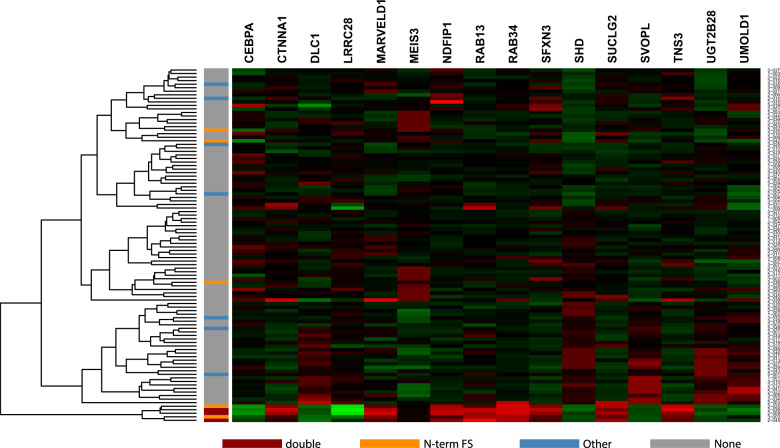
Detection of double *CEBPA* mutants. Expression was analyzed by RNAseq for 16 genes included in the 19-probe signature associated with bi-allelic *CEBPA* mutants [14]. Up- and down-regulated expression levels are shown in green and red, respectively. All 100 AML cases are indicated on the *Y*-axis. All three double *CEBPA* mutants shared the same gene signature (2-009, 2-039, and 2-045). Two single *CEBPA* mutant cases (2-025, 3-003) clustered with the double mutants. Sanger sequencing revealed that one case (3-003) had a 30 bp C-terminal insertion that was missed by HAMLET, while the other case was a true single N-terminal mutant without C-terminal mutation (2-025). Similar results were obtained for the proposed 55-probe signature (data not shown) [13].

Bi-allelic *CEBPA* mutations confer a favorable prognosis to patients with AML with a normal karyotype [1, 8] and typically combine an N-terminal frameshift mutation within the first 357 bp of the coding sequence on one allele with a C-terminal in-frame insertion or deletion between c.834-1074 that disrupts the DNA-binding basic zipper (bZIP) domain on the other allele [28]. The distance between these events frequently precludes formal proof of their location on opposite chromosomes by commonly achievable read lengths on the Illumina platform. However, bi-allelic *CEBPA* mutations are associated with a specific gene expression profile [13, 14]. mRNAseq-based gene expression profiling showed the characteristic bi-allelic *CEBPA* mutation-associated signature for all four AML samples carrying two *CEBPA* mutations (2-009, 2-039, 2-045, and 3-003), including case 3-003 whose C-terminal mutation was missed by HAMLET (Fig. 3). Of eleven AML with single *CEBPA* variants, one case (2-025) with the characteristic N-terminal frameshift mutation clustered with the double *CEBPA* mutants. Combined analyses of variant calling and gene expression profiling can therefore resolve uncertainties of variant calling alone. Since the gene signature for *CEBPA* double mutants is descriptive and not quantitative, it has not been implemented for clinical use in HAMLET.

### Detection of tandem duplications in *FLT3* and *KMT2A*

Reliable detection of the common tandem duplications in exon 14–15 of *FLT3* is of special importance in AML diagnostics since presence of the *FLT3*-ITD is an indication for therapy with a tyrosine kinase inhibitor [29]. In addition to the categorical detection of a *FLT3*-ITD, its position, size, and mutant-to-wild-type allelic ratio have important prognostic impact in AML [30–32]. To improve the insufficient detection of *FLT3*-ITD by VARSCAN, we developed ReSCU as novel algorithm based on calling of partially aligning, SC reads that occur at reciprocal gene positions (Fig. S5).

**Fig. 4:**
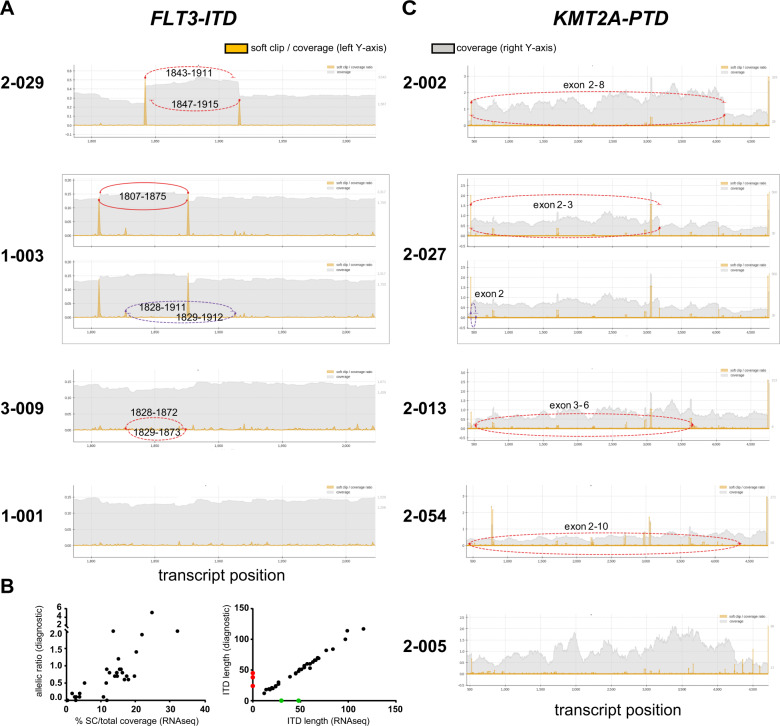
Detection of tandem repeats in *FLT3* and *KMT2A* by the ReSCU algorithm. Graphs depict the ratio of soft-clipped (SC) reads-to-total coverage (left *Y*-axis; yellow peaks) and total read coverage (right *Y*-axis; gray areas) for each position. Reciprocal events at identical and close positions are indicated by solid and dotted lines, while red and purple lines represent dominant and subdominant events, respectively. **a** *FLT3* exons 14–15 (1787–2024 in ENST00000241453 and 1705–1942 in coding sequence). AML cases are representative for a reciprocal event with high SC reads-to-total coverage (2-029), a case with dominant and subdominant reciprocal events (1-003), a reciprocal event with low SC reads-to-total coverage (3-009), and a case without ITD (1-001). **b** Comparison of *FLT3*-ITD results by HAMLET and diagnostic PCR. Diagnostic PCR was performed on genomic DNA using NED-5′-GTAAAACGACGCCCAGTCTGAAGCAATTTAGGTATGAAAGC-3′ and VIC-5′-GGAAACAGCTATGACCATGTACCTTTCAGCATTTTGACG-3′ as forward and reverse primers, respectively. Left panel: SC reads-to-total coverage by HAMLET (*X*-axis) versus allelic ratios by diagnostic PCR calculated from areas under the curve for mutant and wild-type *FLT3* fragments after capillary electrophoresis (*Y*-axis). SC reads-to-total coverage is the sum of dominant and subdominant ITD and average between start and end of ITD. Right panel: ITD length as determined by HAMLET (*X*-axis) versus diagnostic PCR (*Y*-axis). Two subdominant ITD in case 3-027 (green dots) were detected by HAMLET with <0.5% SC reads-to-total coverage but not by routine diagnostics. Three subdominant ITD in cases 2-015, 2-032, and 2-046 (red dots) were detected by routine diagnostics but not by HAMLET. All dominant clones were called by both tests. **c** *KMT2A* exons 2–13 (456–4719 in ENST00000534358 and 433–4696 in coding sequence). AML cases are representative for duplications of exon 2–8 (2-002), exon 2 and exon 2–3 (2-027), exon 3-6 (2-013), exon 2-10 (2-054), and a case without PTD (2-005).

ReSCU identified 36 cases with a *FLT3*-ITD, including all seven cases with a small ITD as detected by VARSCAN and all 34 cases identified by accredited diagnostics (Fig. 4a; Tables SXII and SXIII). Targeted resequencing by NGS additionally confirmed presence and length of the *FLT3*-ITD at positions identified by the soft clipping approach in all but two of these cases (Table SXIV). In the two discrepant cases that were called by ReSCU but not by standard diagnostics, the SC reads represented <1% of total coverage at their respective positions. Careful review of the raw data of the results of the accredited test revealed a weak *FLT3*-ITD signal below the detection threshold in both cases, suggesting higher sensitivity for HAMLET (Fig. S6). The allelic ratio of mutant-to-wild-type *FLT3* fragments correlated well with percentages of SC reads as called by ReSCU (Fig. 4b).

*KMT2A*-PTD in exons 2–13, which are essential to classify AML to the chromatin-spliceosome group [9], lead to elongated proteins that adversely affect clinical outcome of AML [33]. ReSCU identified *KMT2A*-PTD of exon 2–8 and 2–10 in four and two cases, respectively, and duplications of exon 2, 2–3, and 3–6 in single cases (Fig. 4c; Table SXV). All nine *KMT2A*-PTD were validated by RT-PCR and sequencing (Fig. S7 and Table SXVI).

### Detection of gene overexpression

**Fig. 5:**
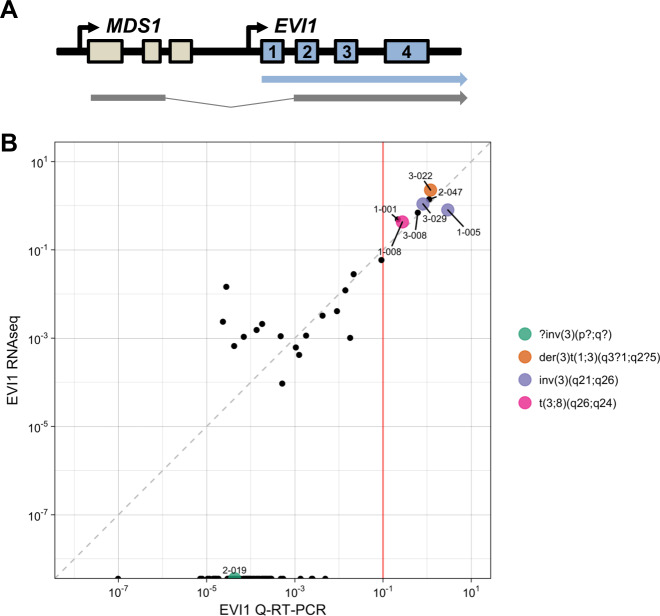
Detection of *EVI1* overexpression. **a** Schematic representation of two mRNA transcripts from the *MECOM* locus on chromosome 3. One transcript contains exon 1–2 of *MDS1* fused to exon 2–15 of *EVI1* (*MDS1-EVI1* transcript), whereas the other transcript contains exon 1–15 of *EVI1* (*EVI1* transcript). **b** Comparison of EVI1 expression by RNAseq and quantitative RT-PCR. *X*-axis: Expression of the first exon of *EVI1* normalized for expression of the *PBGD* housekeeping gene by quantitative RT-PCR (log2 EVI1/PBGD). *Y*-axis: Sum of base coverage of the first exon of *EVI1* per kb transcript and one million mapped reads (log2 BPKM) by RNAseq.

Overexpression of structurally normal genes can influence outcome of AML patients as reported for *BAALC*, *ERG*, *MN1*, *DNMT3B*, *SPARC*, and *EVI1* [8]. The reliability of mRNAseq to measure overexpression was assessed for the *EVI1* transcript that heralds a very poor prognosis in AML (Fig. 5a) [10–12]. Overexpression of *EVI1* can be caused by inv(3)(q21q26) or t(3;3)(q21;q26), but alternative molecular mechanisms exist [12, 34]. *EVI1* exon 1 expression as detected by mRNAseq correlated well to quantitative RT-PCR measurements (Fig. 5b). High *EVI1* expression was seen in both cases (1-005, 3-029) carrying inv(3)(q21q26) and one case (1-008) with t(3;8)(q26;q24) (Table SXVII). Of four additional cases with high *EVI1* expression, one had a der(3)t(1;3)(q3?1;q2?5) without identifiable involvement of the *MECOM1* locus (case 3-022). All three cases overexpressing *EVI1* without 3q26 abnormalities carried *KMT2A* fusion transcripts (cases 1-001 and 3-008: *KMT2A-MLLT3;* case 2-047: *KMT2A-MLLT4;* see Table SIII). This combination of genetic aberrations indicates an extremely poor prognosis in AML [35–37].

### Clonal evolution

HAMLET revealed clonal evolution with acquisition and loss of genetic aberrations in all four pairs of samples taken at diagnosis and subsequent relapse (Table SXVIII). Persistence of an *IDH2* mutation as the presumed founding event established a common origin of a de novo AML and subsequent presumed therapy-related AML despite markedly divergent genotypes (cases 3-021, 3-038). Therefore, the diagnosis of an independent, therapy-related AML was corrected to relapse of the original AML, although both samples would be classified as different AML subtypes.

### Classification and prognostication of AML

**Fig. 6:**
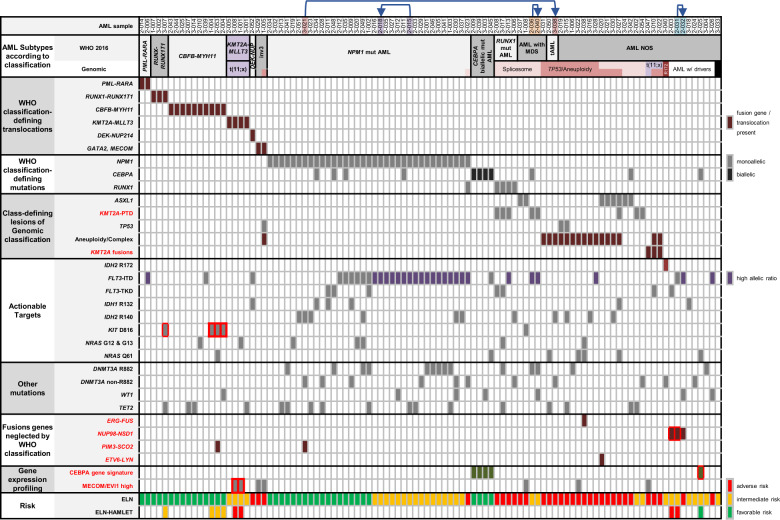
Classification, risk assessment, and actionable targets as derived from HAMLET output and metaphase cytogenetics. AML cases are primarily ordered according to the WHO 2016 classification and secondarily according to the proposed genetic AML classification [9]. Relevant variants with respect to the genetic classification and potentially actionable targets are indicated per case. Red font indicates parameters provided by HAMLET that cannot be reliably addressed by metaphase cytogenetics and targeted NGS sequencing. Prognosis according to the ELN risk [1] is indicated at the bottom. In addition, cases whose ELN risk was altered by HAMLET information are indicated. Red boxes mark the HAMLET parameter that modifies ELN risk. Except for aneuploidies and complex karyotypes, HAMLET provides all information required for risk assessment. Arrows point from a diagnostic sample to the relapse in the same patient. AML with MDS AML with myelodysplasia-associated changes, tAML therapy-related AML, Mut mutated, AML NOS AML not otherwise specified, ELN European leukemia net.

Based on HAMLET and metaphase karyotyping results, 99 cases could be reclassified according to WHO 2016 and genomic classifications (Fig. 6) [8, 9]. For this genetic classification and risk assessment, all variants were selected that have been reported as recurrent mutations in hematological malignancies by Jaiswal et al. [25]. HAMLET information facilitated risk reassessment in six cases, most notably assignment to adverse risk through *NUP98-NSD1* fusions in AML without class-defining lesions, or through *EVI1* overexpression without inv(3)(q21q26).

## Discussion

This study demonstrates the feasibility and accuracy of mRNAseq for the classification and prognostication of AML. Our strategy is built on the novel diagnostic paradigm to capture comprehensive genetic information on a single platform and interrogation of the primary data with respect to currently relevant information. Results of the individual case analysis are reported automatically in a user friendly, easily interpretable format. Both elements of this strategy can improve independently with advances in sample processing and sequencing technology. Relevant new insight into AML subtypes, prognostic factors, and actionable targets can be readily incorporated into the HAMLET algorithm. By linking mRNAseq output to public databases, reliable data interpretation is possible without concomitant germ-line DNA sequencing. Advances in sequencing technology can be implemented to improve diagnostic sensitivity with regard to minor AML subclones without major redevelopment of the data analysis.

HAMLET accurately detects fusion genes with diagnostic relevance for AML, including *KMT2A* fusions not originating from a t(9;11) but from various different partner genes [38]. Accurate detection of the oncogenic fusion genes *ETV6-LYN* [19, 20], *NUP98-NSD1* [21–23], and *PIM3-SCO2* [24] facilitates their recognition as genetically defined entities for AML classification. The remarkable robustness in detecting fusion genes should render HAMLET especially valuable to recognize various fusions of tyrosine kinase genes in Philadelphia chromosome-like acute lymphoblastic leukemia [39].

Independent of the percentage of leukemic blasts in the samples, HAMLET 1.0 identified small variants in 13 frequently mutated AML genes with a chosen threshold of 10% variant reads with 99.2% sensitivity and 100% specificity. Nevertheless, it must be taken into account that very low blast counts in AML samples will limit the sensitivity of HAMLET to detect pathogenic variants. On the other hand, we predict that the detection of minor subclonal variants will be reliably possible with increasing sequencing depth. As known for variant callers such as VARSCAN, two relatively large indels of 23–30 bp were missed. Detection of such indels may be improved by identification of SC reads as already implemented in HAMLET for duplications in *FLT3* and *KMT2A*. Large duplications are notoriously problematic for conventional algorithms including Pindel, even when reads are aligned to large custom libraries of observed duplications and after manual correction [9]. However, integrated calling of SC reads accurately predicted the presence, positions, and length of tandem duplications of interest. Since the majority of *KMT2A-*PTD involve multiple exons and introns, long regions need to be screened to identify these mutations as readily accomplished by ReSCU. The prognostically relevant mutant-to-wild-type *FLT3*-ITD allelic ratio are deducible from mRNAseq data. This aspect, however, currently warrants further calibration. However, determination of this ratio at the transcriptional level as achieved by mRNAseq may be preferable to DNA analysis [40]. Moreover, ITD transcript length of at least 48 bp after splicing of intron 14–15 is associated with poor survival [30, 31, 41, 42].

The intrinsic quantification of gene expression by mRNAseq readily permits measurement of *EVI1* expression, whose overexpression heralds a very poor prognosis even in the absence of structural aberrations involving 3q26. Combined information on gene expression profiles and sequence variants can also resolve difficulties in sequence interpretation as demonstrated for *CEBPA* variants. The characteristic expression profile of double *CEBPA* mutants in a case with a single N-terminal *CEPBA* frameshift mutation could potentially be due to silencing of the wild-type *CEBPA* allele by hypermethylation and could possibly indicate a similarly favorable prognosis [43, 44]. Gene signatures also lend themselves for increasing the diagnostic accuracy of identification of *FLT3*-ITD [45] and *NUP98-NSD1* fusions [21, 23].

We classified our 100 AML according to Papaemmanuil et al. [9] and selected all genetic aberrations called by HAMLET and filtered for predicted importance according to large AML sequencing datasets [25]. Of the 100 AML, 88 cases were classified in single entities, three cases in two subgroups and nine cases had no class-defining lesions. Of the nine cases without class-defining lesions, eight cases had detectable driver mutations and one case had no detectable driver mutation. These numbers perfectly match reported percentages [9], indicating that HAMLET accurately called all genetic information for current classification of AML. Of the 16 additional genes that we are currently implementing in HAMLET (version 2.0), seven genes are relevant for classification and belong to the chromatin-spliceosome group. Mutations in these genes, however, are less frequent and did not affect classification of the 100 AML (data not shown).

Our current experience with RNAseq-HAMLET indicates its potential to replace all genetic tests for classification and risk assessment of AML except for metaphase cytogenetics, which is still required to detect complex karyotypes and aneuploidies. Since RNAseq has successfully been used to detect large chromosomal aberrations in acute lymphoblastic leukemia [46], the aim is to implement this in the next HAMLET version. Although multiplexed PCR and targeted NGS on large gene panels is currently widely implemented in routine diagnostics, long duplications and GC-rich genes remain difficult to sequence by targeted NGS and are therefore often missed (*KMT2A*-PTD) or analyzed separately (*FLT3*-ITD and *CEBPA*). As demonstrated by downsampling the number of RNA reads (Tables SIV, SXI, and SXIII), a sequencing depth of ~50 million read pairs is essential to accurately detect all structurally diverse genetic aberrations in AML. A high sequencing depth is particularly needed to detect nonsense and frameshift variants with a premature stop that may be poorly expressed due to nonsense-mediated RNA decay [3, 26, 27]. This is also the case for genetic aberrations detected by reads that partially align to the GRCh38 genome such as junction reads and spanning fragments for detection of fusion transcripts, as well as SC reads for detection of long tandem duplications in *FLT3* and *KMT2A*. While measurement of *FLT3*-ITD allelic ratios should be performed on DNA according to the ELN recommendations [1], a recent study in pediatric AML suggests measuring *FLT3*-ITD preferentially on RNA, since ITD expression is more strongly associated with poor overall survival than its presence per se [40]. However, RNA-based assays should be sufficiently sensitive to detect *FLT3*-ITDs in AML subclones in order to start treatment with *FLT3* inhibitors [29].

HAMLET results at a sequencing depth of 50 million read pairs can be obtained at a competitive price compared with the conventional diagnostic workflow within 5 days from sample receipt to finished report. The pipeline requires 4 h for sample receipt and isolation of mononuclear cells, 1 h for isolation of total RNA, 24 h for mRNA isolation, and cDNA library preparation, 44 h for RNA sequencing, 24 h for RNAseq analysis and 15 min for data interpretation (excluded time for sample shipment, data transfer and other logistics that are dependent on geographic location, computational networks and negotiations).

In conclusion, HAMLET appears to be a reliable approach to obtain comprehensive genetic information with prognostic and therapeutic relevance for AML in a single assay. RNAseq-based diagnostics also have potential for improved risk assessment and prediction of sensitivity to targeted agents on an individual basis [4, 47]. Based on results of the present study, we are currently implementing HAMLET as routine diagnostic procedure at Leiden University Medical Center to improve risk-classification and personalized treatment of AML.

## Supplementary information

Supplemental file

## Data Availability

The latest version of HAMLET is available under the MIT licence from https://git.lumc.nl/hem/hamlet. The version of HAMLET used in this publication is available in a separate branch, i.e., https://git.lumc.nl/hem/hamlet/tree/publication.

## References

[1] Dohner H, Estey E, Grimwade D, Amadori S, Appelbaum FR, Buchner T (2017). Diagnosis and management of AML in adults: 2017 ELN recommendations from an international expert panel. Blood.

[2] Dohner H, Weisdorf DJ, Bloomfield CD (2015). Acute myeloid leukemia. N. Engl J Med.

[3] Ley TJ, Miller C, Ding L, Raphael BJ, Mungall AJ, Robertson AG, et al. Genomic and epigenomic landscapes of adult de novo acute myeloid leukemia. N Engl J Med. 2013;368:2059–74. 10.1056/NEJMoa1301689 PMC3767041 23634996

[4] Tyner JW, Tognon CE, Bottomly D, Wilmot B, Kurtz SE, Savage SL (2018). Functional genomic landscape of acute myeloid leukaemia. Nature.

[5] Bowman RL, Busque L, Levine RL (2018). Clonal hematopoiesis and evolution to hematopoietic malignancies. Cell Stem Cell.

[6] Jaiswal S, Fontanillas P, Flannick J, Manning A, Grauman PV, Mar BG (2014). Age-related clonal hematopoiesis associated with adverse outcomes. N Engl J Med.

[7] Xie M, Lu C, Wang J, McLellan MD, Johnson KJ, Wendl MC (2014). Age-related mutations associated with clonal hematopoietic expansion and malignancies. Nat Med.

[8] Arber DA, Orazi A, Hasserjian R, Thiele J, Borowitz MJ, Le Beau MM (2016). The 2016 revision to the World Health Organization classification of myeloid neoplasms and acute leukemia. Blood.

[9] Papaemmanuil E, Gerstung M, Bullinger L, Gaidzik VI, Paschka P, Roberts ND (2016). Genomic classification and prognosis in acute myeloid leukemia. N Engl J Med.

[10] Barjesteh van Waalwijk van Doorn-Khosrovani S, Erpelinck C, van Putten WL, Valk PJ, van der Poel-van de Luytgaarde S, Hack R (2003). High EVI1 expression predicts poor survival in acute myeloid leukemia: a study of 319 de novo AML patients. Blood.

[11] Groschel S, Lugthart S, Schlenk RF, Valk PJ, Eiwen K, Goudswaard C (2010). High EVI1 expression predicts outcome in younger adult patients with acute myeloid leukemia and is associated with distinct cytogenetic abnormalities. J Clin Oncol.

[12] Lugthart S, van Drunen E, van Norden Y, van Hoven A, Erpelinck CA, Valk PJ (2008). High EVI1 levels predict adverse outcome in acute myeloid leukemia: prevalence of EVI1 overexpression and chromosome 3q26 abnormalities underestimated. Blood.

[13] van Vliet MH, Burgmer P, de Quartel L, Brand JP, de Best LC, Vietor H (2013). Detection of CEBPA double mutants in acute myeloid leukemia using a custom gene expression array. Genet Test Mol Biomark.

[14] Wouters BJ, Lowenberg B, Erpelinck-Verschueren CA, van Putten WL, Valk PJ, Delwel R (2009). Double CEBPA mutations, but not single CEBPA mutations, define a subgroup of acute myeloid leukemia with a distinctive gene expression profile that is uniquely associated with a favorable outcome. Blood.

[15] Ritchie ME, Phipson B, Wu D, Hu Y, Law CW, Shi W (2015). limma powers differential expression analyses for RNA-sequencing and microarray studies. Nucleic Acids Res.

[16] Genomes Project C, Auton A, Brooks LD, Durbin RM, Garrison EP, Kang HM (2015). A global reference for human genetic variation. Nature.

[17] Boomsma DI, Wijmenga C, Slagboom EP, Swertz MA, Karssen LC, Abdellaoui A (2014). The genome of the Netherlands: design, and project goals. Eur J Hum Genet.

[18] van der Gaag KJ, de Leeuw RH, Hoogenboom J, Patel J, Storts DR, Laros JF (2016). Massively parallel sequencing of short tandem repeats-population data and mixture analysis results for the PowerSeq system. Forensic Sci Int Genet.

[19] Ma ESK, Wan TSK, Au CH, Ho DN, Ma SY, Ng MHL (2017). Next-generation sequencing and molecular cytogenetic characterization of ETV6-LYN fusion due to chromosomes 1, 8 and 12 rearrangement in acute myeloid leukemia. Cancer Genet.

[20] Takeda Y, Nakaseko C, Tanaka H, Takeuchi M, Yui M, Saraya A (2011). Direct activation of STAT5 by ETV6-LYN fusion protein promotes induction of myeloproliferative neoplasm with myelofibrosis. Br J Haematol.

[21] Hollink IH, van den Heuvel-Eibrink MM, Arentsen-Peters ST, Pratcorona M, Abbas S, Kuipers JE (2011). NUP98/NSD1 characterizes a novel poor prognostic group in acute myeloid leukemia with a distinct HOX gene expression pattern. Blood.

[22] Ostronoff F, Othus M, Gerbing RB, Loken MR, Raimondi SC, Hirsch BA (2014). NUP98/NSD1 and FLT3/ITD coexpression is more prevalent in younger AML patients and leads to induction failure: a COG and SWOG report. Blood.

[23] Shiba N, Ichikawa H, Taki T, Park MJ, Jo A, Mitani S (2013). NUP98-NSD1 gene fusion and its related gene expression signature are strongly associated with a poor prognosis in pediatric acute myeloid leukemia. Genes Chromosomes Cancer.

[24] Menezes J, Makishima H, Gomez I, Acquadro F, Gomez-Lopez G, Grana O (2013). CSF3R T618I co-occurs with mutations of splicing and epigenetic genes and with a new PIM3 truncated fusion gene in chronic neutrophilic leukemia. Blood Cancer J.

[25] Jaiswal S, Natarajan P, Silver AJ, Gibson CJ, Bick AG, Shvartz E (2017). Clonal hematopoiesis and risk of atherosclerotic cardiovascular disease. N Engl J Med.

[26] Lindeboom RG, Supek F, Lehner B (2016). The rules and impact of nonsense-mediated mRNA decay in human cancers. Nat Genet.

[27] Hu Z, Yau C, Ahmed AA (2017). A pan-cancer genome-wide analysis reveals tumour dependencies by induction of nonsense-mediated decay. Nat Commun.

[28] Koschmieder S, Halmos B, Levantini E, Tenen DG (2009). Dysregulation of the C/EBPalpha differentiation pathway in human cancer. J Clin Oncol.

[29] Stone RM, Larson RA, Dohner H (2017). Midostaurin in FLT3-mutated acute myeloid leukemia. N Engl J Med.

[30] Gale RE, Green C, Allen C, Mead AJ, Burnett AK, Hills RK (2008). The impact of FLT3 internal tandem duplication mutant level, number, size, and interaction with NPM1 mutations in a large cohort of young adult patients with acute myeloid leukemia. Blood.

[31] Linch DC, Hills RK, Burnett AK, Khwaja A, Gale RE (2014). Impact of FLT3(ITD) mutant allele level on relapse risk in intermediate-risk acute myeloid leukemia. Blood.

[32] Pratcorona M, Brunet S, Nomdedeu J, Ribera JM, Tormo M, Duarte R (2013). Favorable outcome of patients with acute myeloid leukemia harboring a low-allelic burden FLT3-ITD mutation and concomitant NPM1 mutation: relevance to post-remission therapy. Blood.

[33] Dohner K, Tobis K, Ulrich R, Frohling S, Benner A, Schlenk RF (2002). Prognostic significance of partial tandem duplications of the MLL gene in adult patients 16 to 60 years old with acute myeloid leukemia and normal cytogenetics: a study of the Acute Myeloid Leukemia Study Group Ulm. J Clin Oncol.

[34] Zoccola D, Legros L, Cassuto P, Fuzibet JG, Nucifora G, Raynaud SD (2003). A discriminating screening is necessary to ascertain EVI1 expression by RT-PCR in malignant cells from the myeloid lineage without 3q26 rearrangement. Leukemia.

[35] Bindels EM, Havermans M, Lugthart S, Erpelinck C, Wocjtowicz E, Krivtsov AV (2012). EVI1 is critical for the pathogenesis of a subset of MLL-AF9-rearranged AMLs. Blood.

[36] Groschel S, Schlenk RF, Engelmann J, Rockova V, Teleanu V, Kuhn MW (2013). Deregulated expression of EVI1 defines a poor prognostic subset of MLL-rearranged acute myeloid leukemias: a study of the German-Austrian Acute Myeloid Leukemia Study Group and the Dutch-Belgian-Swiss HOVON/SAKK Cooperative Group. J Clin Oncol.

[37] Ho PA, Alonzo TA, Gerbing RB, Pollard JA, Hirsch B, Raimondi SC (2013). High EVI1 expression is associated with MLL rearrangements and predicts decreased survival in paediatric acute myeloid leukaemia: a report from the children’s oncology group. Br J Haematol.

[38] Winters AC, Bernt KM (2017). MLL-rearranged leukemias-an update on science and clinical approaches. Front Pediatr.

[39] Roberts KG, Li Y, Payne-Turner D, Harvey RC, Yang YL, Pei D (2014). Targetable kinase-activating lesions in Ph-like acute lymphoblastic leukemia. N Engl J Med.

[40] Cucchi DGJ, Denys B, Kaspers GJL, Janssen J, Ossenkoppele GJ, de Haas V (2018). RNA-based FLT3-ITD allelic ratio is associated with outcome and ex vivo response to FLT3 inhibitors in pediatric AML. Blood.

[41] Schlenk RF, Kayser S, Bullinger L, Kobbe G, Casper J, Ringhoffer M (2014). Differential impact of allelic ratio and insertion site in FLT3-ITD-positive AML with respect to allogeneic transplantation. Blood.

[42] Versluis J, In ‘t Hout FE, Devillier R, van Putten WL, Manz MG, Vekemans MC (2017). Comparative value of post-remission treatment in cytogenetically normal AML subclassified by NPM1 and FLT3-ITD allelic ratio. Leukemia.

[43] Figueroa ME, Wouters BJ, Skrabanek L, Glass J, Li Y, Erpelinck-Verschueren CA (2009). Genome-wide epigenetic analysis delineates a biologically distinct immature acute leukemia with myeloid/T-lymphoid features. Blood.

[44] Taskesen E, Bullinger L, Corbacioglu A, Sanders MA, Erpelinck CA, Wouters BJ (2011). Prognostic impact, concurrent genetic mutations, and gene expression features of AML with CEBPA mutations in a cohort of 1182 cytogenetically normal AML patients: further evidence for CEBPA double mutant AML as a distinctive disease entity. Blood.

[45] Whitman SP, Maharry K, Radmacher MD, Becker H, Mrozek K, Margeson D (2010). FLT3 internal tandem duplication associates with adverse outcome and gene- and microRNA-expression signatures in patients 60 years of age or older with primary cytogenetically normal acute myeloid leukemia: a Cancer and Leukemia Group B study. Blood.

[46] Gu Z, Churchman ML, Roberts KG, Moore I, Zhou X, Nakitandwe J (2019). PAX5-driven subtypes of B-progenitor acute lymphoblastic leukemia. Nat Genet.

[47] Wang M, Lindberg J, Klevebring D, Nilsson C, Lehmann S, Gronberg H (2018). Development and validation of a novel RNA sequencing-based prognostic score for acute myeloid leukemia. J Natl Cancer Inst.

