# Supplementary material for: Comprehensive diagnostics of acute myeloid leukemia by whole transcriptome RNA sequencing
Source: Leukemia. 2020 Mar 3;35(1):47–61. doi: 10.1038/s41375-020-0762-8 (PMC7787979; doi:10.1038/s41375-020-0762-8)
Supplement: Supplementary file 1 — Supplemental file [file 41375_2020_762_MOESM1_ESM.pdf]

Supplemental Material Arindrarto *et al.*

“Comprehensive diagnostics of acute myeloid leukemia by whole transcriptome RNA sequencing”

Table of contents

	Page
Figure S1	2
Figure S2	3
Figure S3	4
Figure S4	5
Figure S5	6
Figure S6	7
Figure S7	8
Table SI	9
Table SII	12
Table SIII	14
Table SIV	15
Table SV	16
Table SVI	19
Table SVII	20
Table SVIII	29
Table SIX	31
Table SX	32
Table SXI	35
Table SXII	43
Table SXIII	46
Table SXIV	48
Table SXV	50
Table SXVI	51
Table SXVII	52
Table SXVIII	53
Exemplary HAMLET report (case 1-003)	54

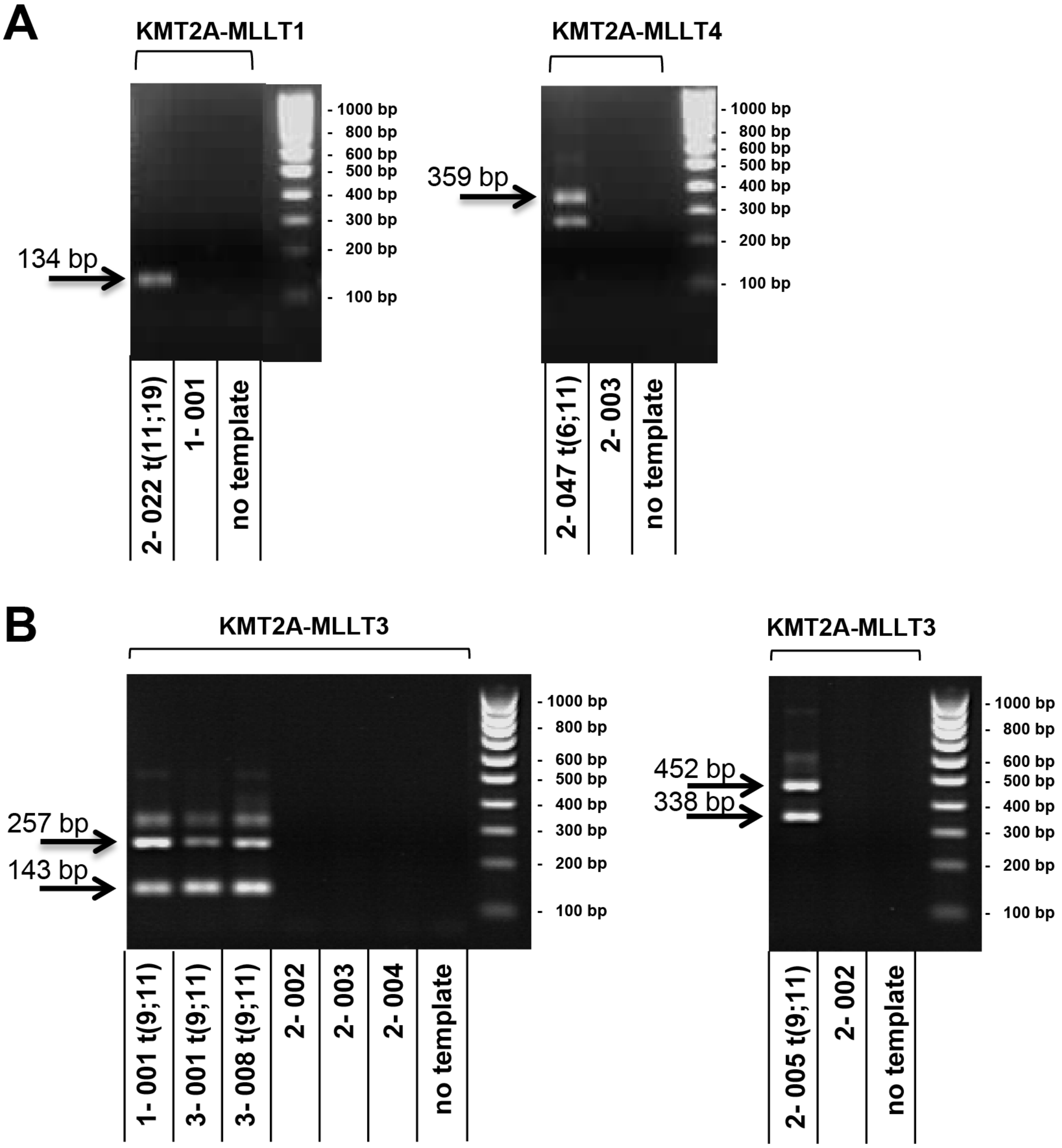

Figure S1. Validation of *KMT2A* fusion transcripts by RT-PCR.

Primers used for amplification are shown in Table SV. The breakpoints of all fusion transcripts were confirmed by sequencing (Table SIII).

A. Three fusion transcripts were detected by HAMLET in which *KMT2A* is fused to other partner genes than *MLLT3*, i.e. *KMT2A-MLLT1* in AML case 2-022, *KMT2A-MLLT4* in case 2-047 and *KMT2A-MLLT6* in case 3-010 (Fig 1B). All 3 fusion transcripts were validated by RT-PCR as a fragment of 134 bp (exon 9 of *KMT2A* fused to exon 2 of *MLLT1*) and a fragment of 359 bp (exon 8 of *KMT2A* fused to exon 2 of *MLLT4*). AML cases 1-001, 2-003, and a reaction without cDNA (no template) were included as negative controls.

B. *KMT2A-MLLT3* fusion transcripts containing exon 9 or 10 of *KMT2A* fused to exon 6 of *MLLT3* in AML cases 1-001, 3-001 and 3-008 were validated by RT-PCR as fragments of 257 and 143 bp (left). Two other *KMT2A-MLLT3* transcripts containing exon 9 or 10 of *KMT2A* fused to exon 5 of *MLLT3* were detected in case 2-005. These transcripts were validated by RT-PCR as fragments of 452 and 338 bp (right). AML cases 2-002, 2-003, 2-004, and a reaction without cDNA were included as negative controls.

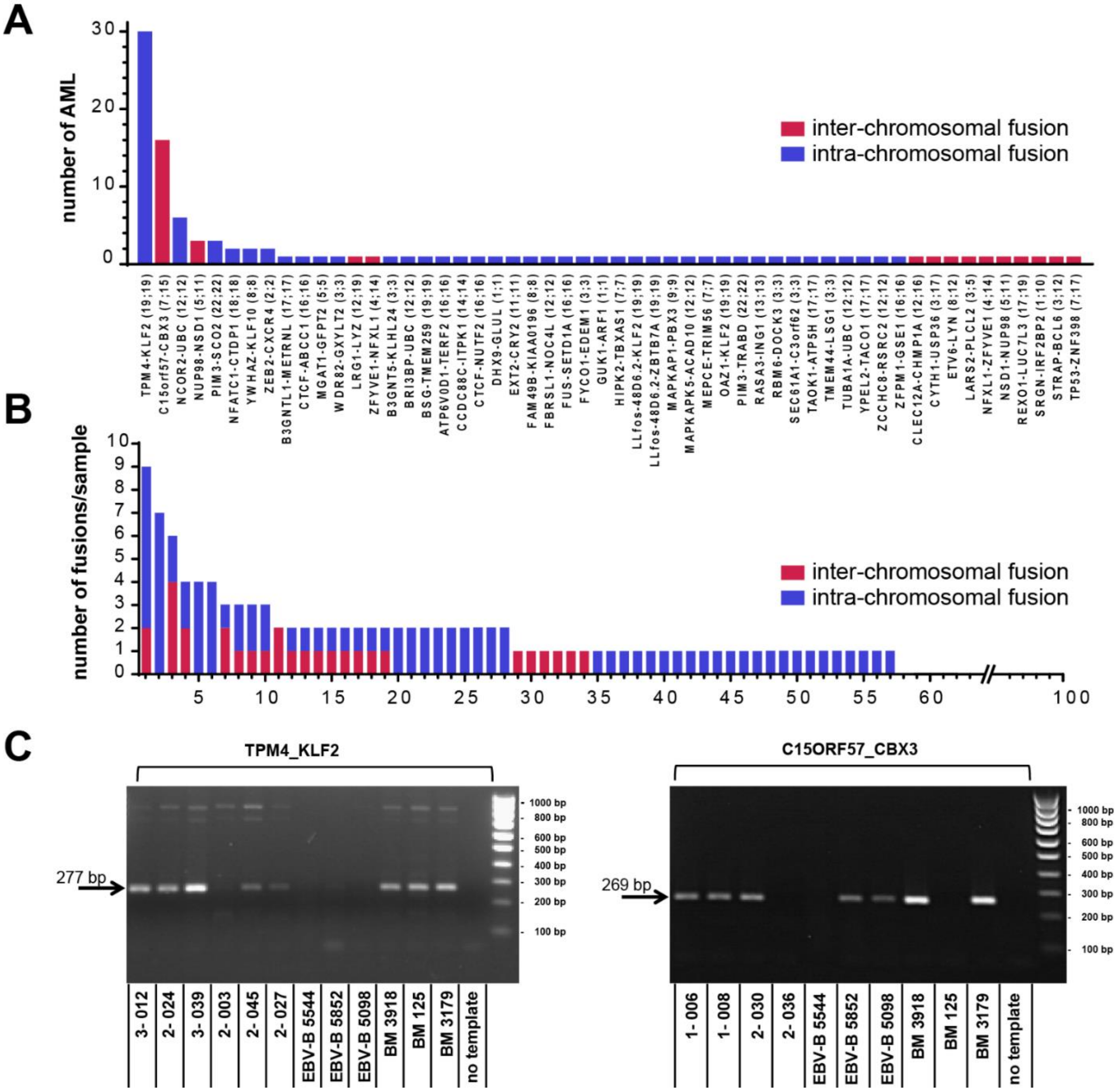

Figure S2. Detection and validation of fusion transcripts without corresponding structural abnormalities by metaphase cytogenetics.

A. Number of AML cases in which fusion transcripts were detected. Fusion transcripts between genes on the same chromosome (intra-chromosomal fusions) and fusion transcripts between genes on different chromosomes (inter-chromosomal fusions) are represented by blue and red bars, respectively. Fusion transcripts *TPM4-KLF2*, *C15ORF57-CBX3*, *NCOR2-UBC*, *NUP98-NSD1*, *NSD1-NUP98*, *PIM3-SCO2*, *ZEB2-CXCR4* and *ETV6-LYN* were validated by RT-PCR and the breakpoints of a number of these transcripts were confirmed by sequencing (Fig. 1B; Tables SIV & SV).

B. Number of different fusion transcripts without corresponding structural abnormalities by metaphase cytogenetics for each AML. AML are ranked by their number of different fusion transcripts.

C. Validation of the highly prevalent *TPM4-KLF2* and *C15ORF57-CBX3* fusion transcripts by RT-PCR with custom primers (Table SV). An amplicon of 277 bp demonstrated intrachromosomal fusion of *TPM4* exon 1 to *KLF2* exon 3 in all 3 positive AML (3-012, 2-024, 3-039) as well as in 2 control cases (2-045 and 2-027) and 3 healthy BM samples, but not in EBV-B cells and in a reaction without cDNA (no template). An amplicon of 269 bp demonstrates fusion of *C15ORF57* intron 2 to *CBX3* exon 1 in 2 positive AML (1-006, 1-008) as well as in one of the 2 control AML (2-030) and in 2 of the 3 EBV-B and healthy BM samples. *C15ORF57-CBX3* is a gene copy number polymorphism caused by retro-transposition with duplication of *CBX3* and insertion between the second and third exons of *C15orf57* (Schrider *et al.*, PLoS Genet 2013).

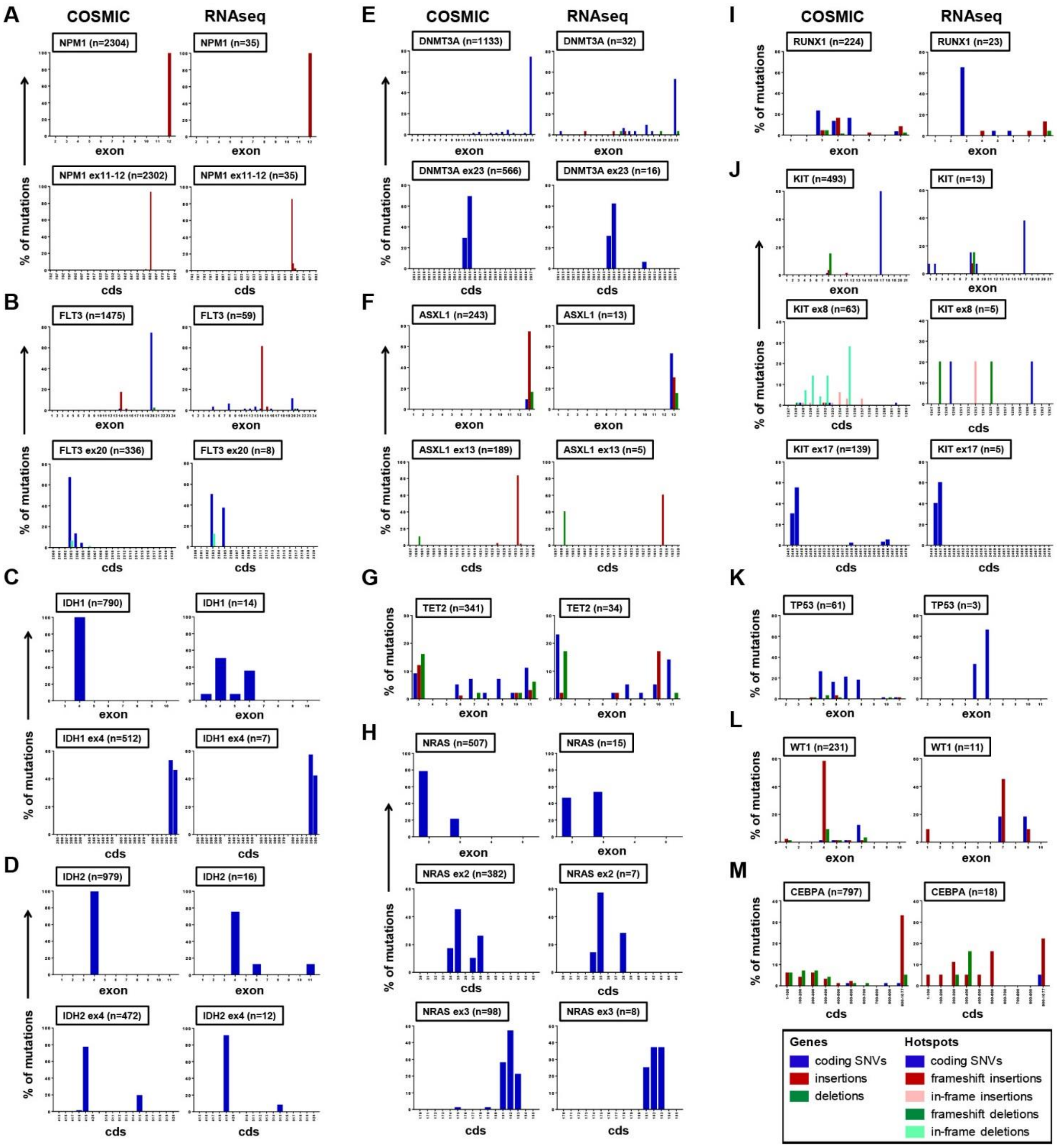

Figure S3. Comparison of distribution of small variants in recurrently mutated AML genes between COSMIC and HAMLET.

In the analysis of COSMIC data, all variants with protein consequences are included for which the exact location is known. For each gene (upper graphs), coding SNV, insertions and deletions in separate exons are represented by blue, red and green bars, respectively. For all hotspots (middle & lower graphs), the position of the variant in the coding sequence is indicated as well as whether indels are in-frame (light bar) or frameshift (dark bar) variants.

A. *NPM1.*

B. *FLT3.* In-frame variants in exon 14-15 are underrepresented in COSMIC due to lack of information on exon location for many *FLT3*-ITD.

C. *IDH1*, D. *IDH2*, E. *DNMT3A*.

F. *ASXL1*.

G. *TET2*, H. *NRAS*, I. *RUNX1*, J. *KIT*, K. *TP53*, L. *WT1*, M. *CEBPA*.

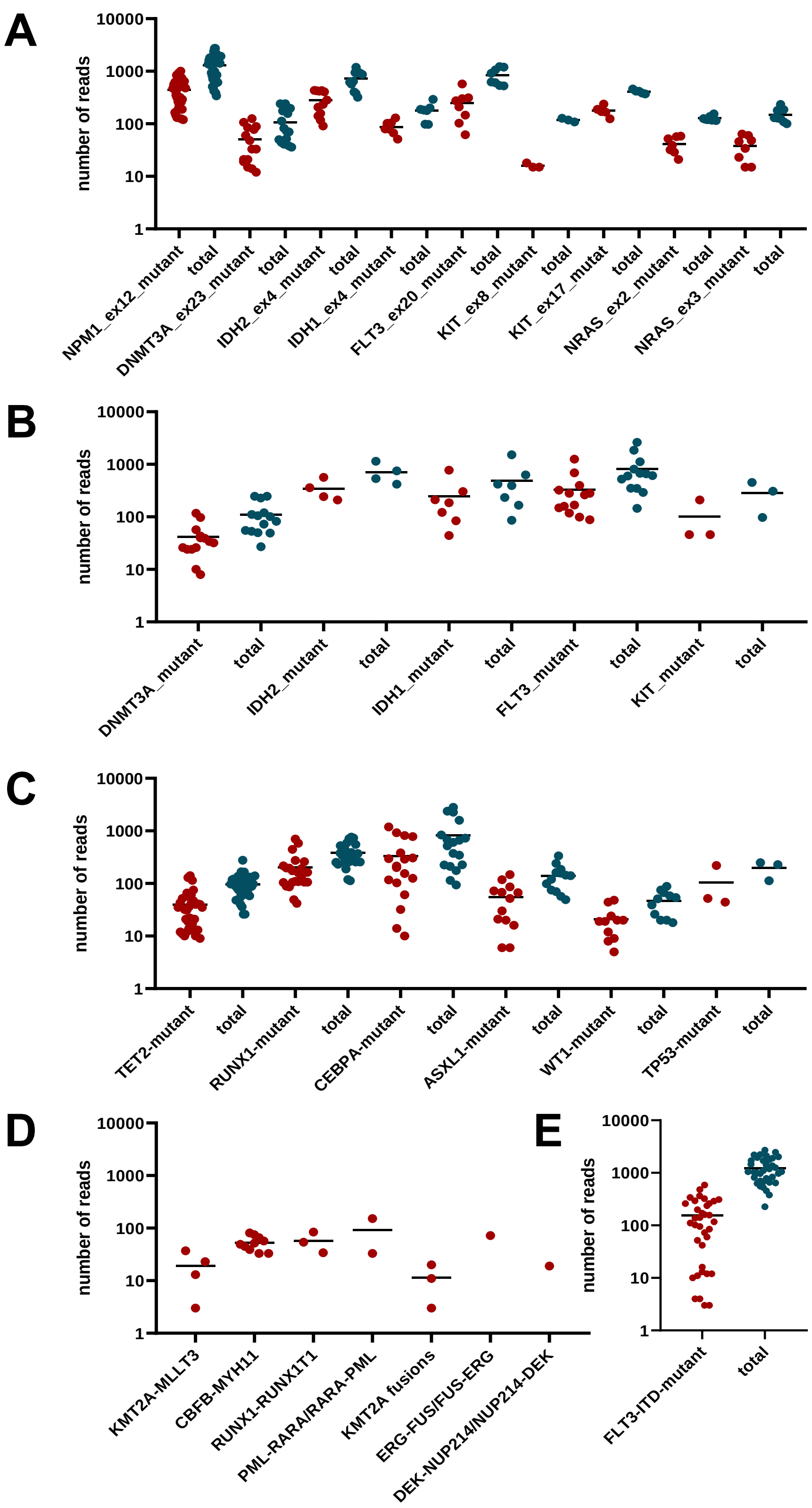

Figure S4. Number of reads for genetic aberrations in AML.

A. Mutant and total reads at the same position in hotspot regions in *NPM1* (exon 12), *DNMT3A* (exon 23), *IDH2* (exon 4), *IDH1* (exon 4), *FLT3*-TKD (exon 20), *KIT* (exon 8 & 17), *NRAS* (exon 2 & 3).
B. Mutant and total reads at the same position outside hotspots in *DNMT3A*, *IDH2*, *IDH1*, *FLT3*, *KIT*.
C. Mutant and total reads at the same position in *TET2*, *RUNX1*, *CEBPA*, *ASXL1*, *WT1* and *TP53*.
D. Number of junction reads and spanning fragments for fusion genes.

Soft clipped reads for *FLT3*-ITD and corresponding total reads in exon 14 & 15.

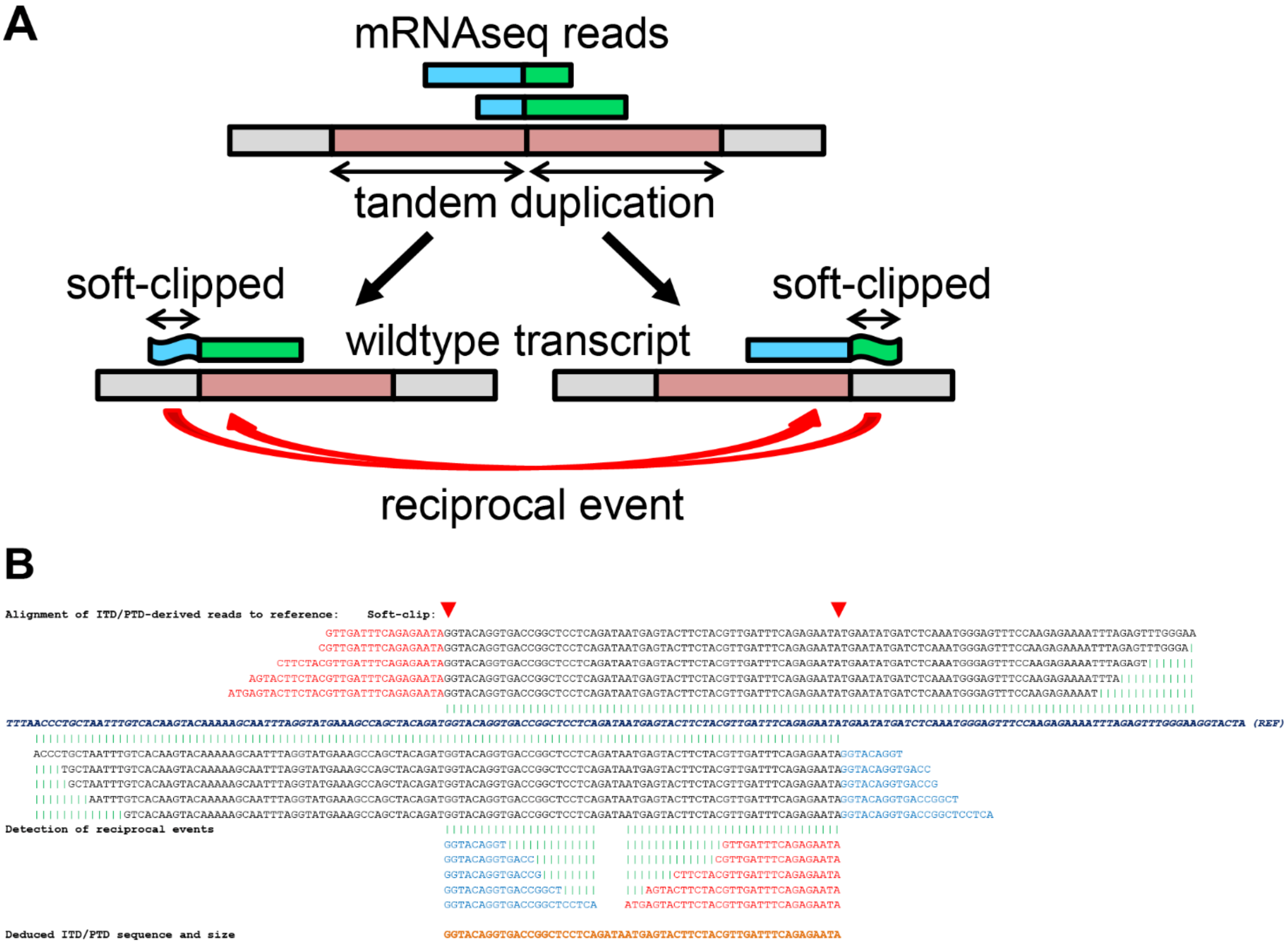

Figure S5. ReSCU for detection of tandem duplication in *FLT3* and *KMT2A*.

A) ReSCU is a new bioinformatics tool integrated in HAMLET to detect long tandem duplications in *FLT3* (*FLT3*-ITD) and *KMT2A* (*KMT2A*-PTD). ReSCU is based on detection of soft clipped reads, i.e. reads that partially align to a reference sequence. The soft clipped fragments of these reads contain bases that fail to align to the reference sequence. In ReSCU, the number of soft clipped reads to total coverage is determined for each position in exon 14-15 of the *FLT3* and exon 2-13 of the *KMT2A* transcript. In addition, the soft clipped fragments are separately searched for mapping to the reference sequence and reciprocity. *FLT3*-ITD or *KMT2A*-PTD are called when reciprocity is established for two or more soft clipped reads., i.e. soft clipped sequences of reads aligning to the start of the ITD or PTD map to aligned sequences of soft clipped reads at the end, and *vice versa*.

B) An example of a *FLT3*-ITD. The positions of soft clips are indicated by red triangles. The reference sequence is in dark blue and cursive. Sequences in red and light blue represent soft clipped fragments of reads partially aligning to the start and end of the ITD, respectively. Soft clipped sequences of reads aligning to the start of the ITD are mapped to aligned sequences of soft clipped reads at the end, and *vice versa*, indicating reciprocity. The ITD is the sequence in orange between two soft clips.

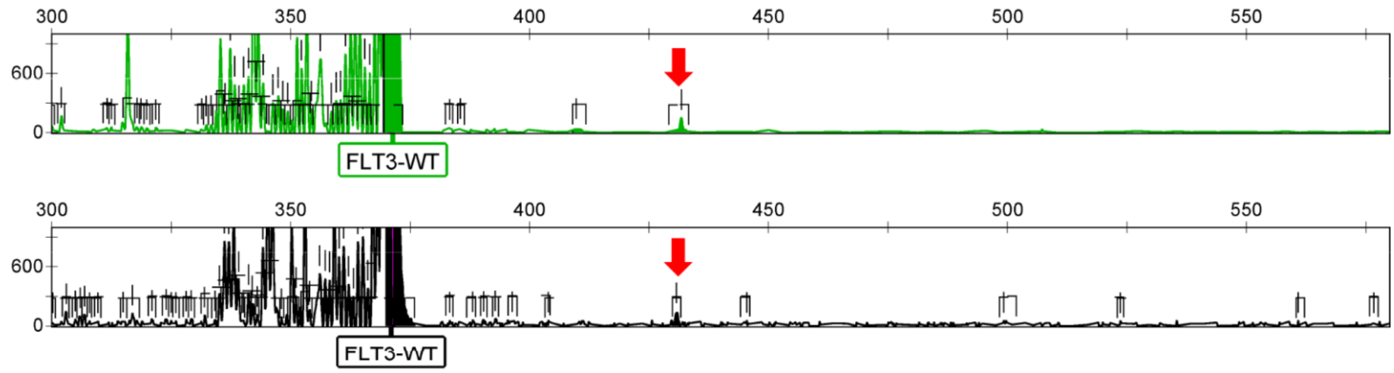

Figure S6. Retrospective analysis of diagnostic *FLT3*-ITD results.

Retrospective analysis of diagnostic results for case 2-028 (positive by RNAseq but negative by routine diagnostics) showed a weak signal of a mutant allele indicated by red arrows below the standard cut-off for FLT3-ITD > 200. PCR was performed on genomic DNA using NED-5'-GTAAAACGACGCCCAGTCTGAAGCAATTTAGGTATGAAAGC-3' and VIC-5'-GGAAACAGCTATGACCATGTACCTTTCAGCATTTTGACG-3' as forward and reverse primers, respectively. PCR fragments were detected after capillary electrophoresis by measuring green (VIC; upper) and yellow (NED; lower) dyes.

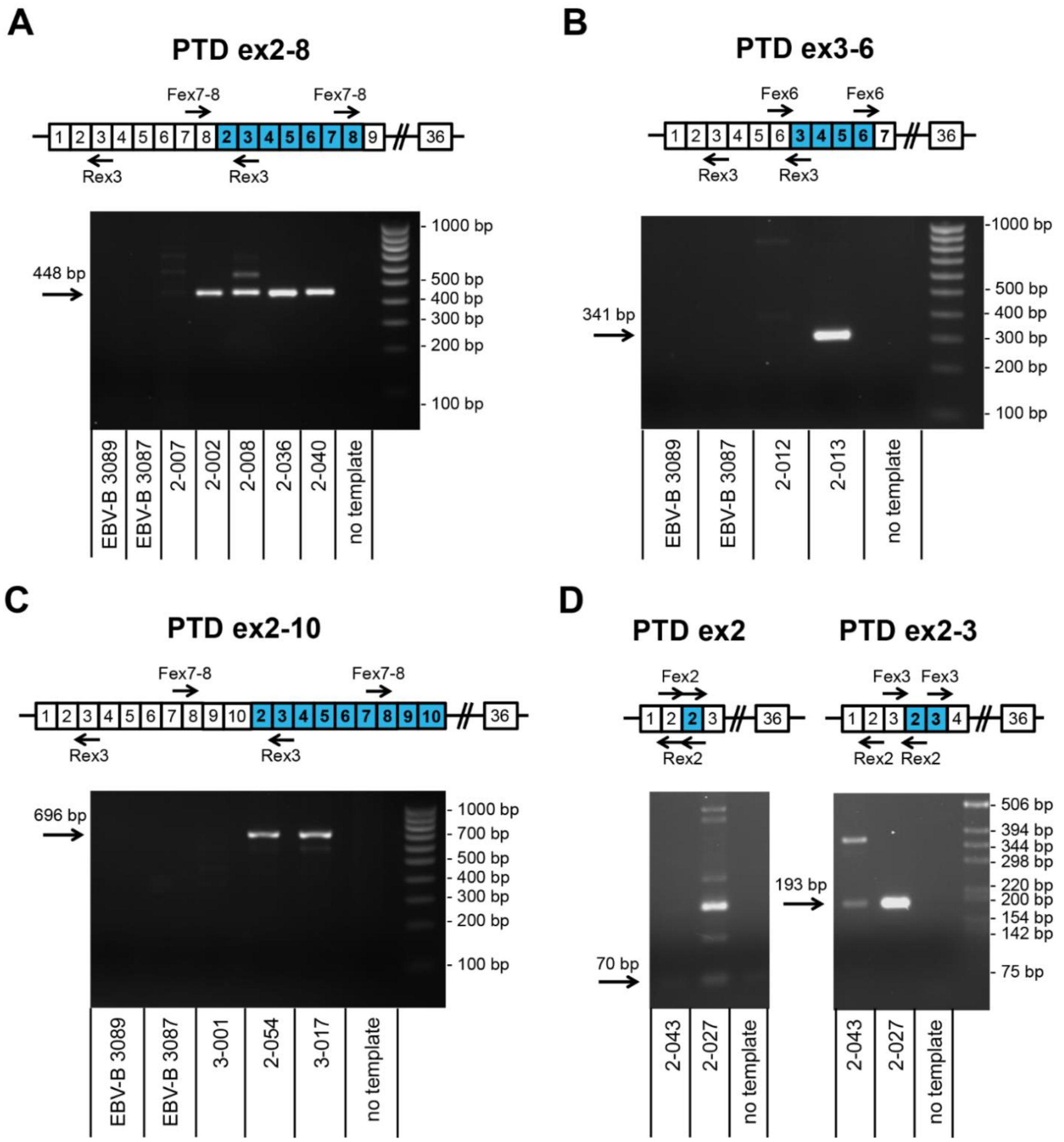

Figure S7. Validation of *KMT2A*-PTD as detected by HAMLET by RT-PCR.

Two EBV-B samples, negative AML cases (2-007, 2-012, 3-001, 2-043) and a reaction without cDNA (no template) were included as negative controls.

A. Exon 2-8 PTD in cases 2-002, 2-008, 2-036, and 2-040 detected as 448 bp amplicons using a forward primer in exon 7-8 and reverse primer in exon 3.

B. Exon 3-6 PTD in case 2-013 detected as 341 bp amplicon using a forward primer in exon 6 and a reverse primer in exon 3.

C. Exon 2-10 PTD in cases 2-054 and 3-017 detected as 696 bp amplicon using a forward primer in exon 7-8 and a reverse primer in exon 3.

D. Exon 2 PTD in case 2-027 detected as 70 bp amplicon using a forward and reverse primer in exon 2 with the reverse primer located upstream of the forward primer (left). The reverse primer in exon 2 also binds to exon 3 sequences, which in combination with the forward primer in exon 2 results in various PCR fragments larger than 70 bp. Exon 2-3 PTD in case 2-027 detected 193 bp amplicon using a forward primer in exon 3 and reverse primer in exon 2 (right). The reverse primer in exon 2 also binds to exon 3 sequences, which in combination with the forward primer in exon 3 results in various PCR fragments representing wildtype *KMT2A* in case 2-043.

Table SI. AML samples analysed by RNAseq.

Sample[1]	Patient	Gender	Age	WHO diagnosis[6]	Primary/ Relapse	Source[7]	Blast (%)	Karyotype[8]	FLT3 -ITD[9]	Mut. NPM1[9]
1-001	8412	M	34	AML with t(9;11); *KMT2A-MLLT3*	Primary	BM	96	46, XX, t(9;11)(p21;q23)	neg	neg
1-002	9559	F	41	AML with mutated NPM1	Primary	BM	87	46, XX	pos	pos
1-003	10197	F	65	AML with mutated NPM1	Primary	BM	98	46, XX	pos	pos
1-004	9848	M	77	AML with inv(16); *CBFB-MYH11*	Primary	BM	70	46, XY, inv(16)(p13q22)	neg	neg
1-005	4781	M	49	AML with inv(3); *GATA2, MECOM*	Primary	PB	22	46, XY, inv(3)(q21q26), del(7)(q21), -17, der(19) t(17;19)(q2?2;q13) / 46-48, XY, del(2)(p21)[8],-3[9], del(3)(q23)[8],+6[6],-7[8], del(11)(p1?3)[9], add(12)(p11)[9], inv(15)(q?13q?23)[6], +ring[8], +mar1[8]	neg	neg
1-006	8522	F	71	Acute monoblastic/monocytic leukemia	Primary	BM	55	46, X, add(X)(q2?1), add(1)(q4?3), add(3)(p21), add(20)(q11.2)	neg	neg
1-007	9009	M	45	AML with t(8;21); *RUNX1-RUNX1T1*	Primary	BM	67	46, XY, t(8;21)(q22;q22)	neg	neg
1-008	10143	M	61	AML with myelodysplasia-related changes	Primary	BM	15	46, XY, t(3;8)(q26;q24)	neg	neg
2-001	587	F	46	Acute monoblastic/monocytic leukemia	Primary	PB	90	46, XX	pos	pos
2-002	1143	M	65	Acute monoblastic/monocytic leukemia	Primary	BM	90	46, XY	neg	neg
2-003	1127	M	46	Acute monoblastic/monocytic leukemia	Primary	PB	95	46,XY	neg	neg
2-004	1310	M	49	AML with inv(16); *CBFB-MYH11*	Primary	BM	85	46, XY, inv(16)(p13q22)	neg	neg
2-005	1466	M	37	AML with t(9;11); *KMT2A-MLLT3*	Primary	BM	28	46, XY, t(9;11)(p21;q23)	neg	neg
2-006	1591	M	22	Acute promyelocytic leukemia with t(15;17); *PML-RARA*	Primary	PB	95	46, XY, t(15;17)(q24;q21)	pos	neg
2-007	1775	M	49	AML with inv(16); *CBFB-MYH11*	Primary	PB	89	46, XY, inv(16)(p13q22)	neg	neg
2-008	2055	M	77	Acute monoblastic/monocytic leukemia	Primary	PB	64	47, XY, +11	neg	neg
2-009	2250	M	38	AML NOS with maturation	Primary	BM	95	46, XY	neg	neg
2-010	2467	M	48	AML with inv(16); *CBFB-MYH11*	Primary	BM	60	49, XY, +8, +14, inv(16)(p13q22), +21	neg	neg
2-011	2536	F	44	AML NOS without maturation	Primary	BM	95	46, XX	neg	pos
2-012	2899	F	44	Acute monoblastic/monocytic leukemia	Primary	PB	80	46, XX	pos	pos
2-013	3009	M	71	Acute monoblastic/monocytic leukemia	Primary	BM	70	46, XY	pos	neg
2-014	3097	M	50	Acute promyelocytic leukemia with t(15;17); *PML-RARA*	Primary	BM	80	46, XX, del(6)(q?), t(15;17)(q24;q21)	neg	neg
2-015[2]	3361	F	35	AML NOS with maturation	Primary	PB	79	46, XX	pos	pos
2-016	3370	F	57	AML NOS with maturation	Relapse	PB	82	46, XX, del(17)(p11)	pos	pos
2-018[2]	3361	F	36	AML NOS with maturation	Relapse	BM	90	no metaphases	pos	pos
2-019	3714	F	66	AML NOS with minimal differentiation	Primary	BM	80	45-46, XX, ?inv(3)(p?q?),4, add(5)(q1?2), der(9)t(4;9)(q21;q34), ?dic(11;?)(p1?2;?), add(12)(p13), der(12)add(12)(p?)del(12)(q22), add(14)(q13), -17, del(17)(q21), +1-2mar[19]/46,XX[1]	neg	neg
2-020	3778	F	41	AML with myelodysplasia-related changes	Primary	PB	25	47, XX, +8, add(8)(q24), del(12)(p13), i(17)(q10)	neg	neg
2-021	3870	M	50	AML NOS with maturation	Primary	BM	27	47, XY, +8	neg	neg
2-022	4072	F	35	Acute monoblastic/monocytic leukemia	Primary	BM	95	46, XX, der(11)t(11;11)(q13;q23) inv(11)(q13q23) t(11;19)(q23;p13), der(11)t(11;11)(q13;q23), del(12)(p13),der(19)t(11;19)(q23;p13)	neg	neg
2-023	4443	F	57	AML with mutated NPM1	Primary	BM	82	46, XX	pos	pos
2-024	4690	F	47	Acute myelomonocytic leukemia	Primary	BM	45	46, XX	neg	neg
2-025	4716	M	35	AML NOS without maturation	Primary	BM	98	45, XY, -7	neg	neg
2-027	5074	M	63	Acute monoblastic/monocytic leukemia	Primary	PB	55	47, XY, del(9)(q?11q?31),+20 / 46, XY, t(10;16)(q21;p13) / 48, XY, +3, +20	neg	neg
2-028	5205	M	56	AML NOS without maturation	Primary	PB	39	46, XY	neg	pos
2-029	5444	F	56	AML with mutated NPM1	Primary	BM	55	46, XX	pos	pos
2-030	5518	F	59	AML with mutated NPM1	Primary	BM	95	46, XX	pos	pos
2-031[3]	5672	M	34	AML with myelodysplasia-related changes	Primary	BM	40	46, XY	pos	neg
2-032[3]	5672	M	36	AML with myelodysplasia-related changes	Relapse	PB	90	46, XY	pos	neg
2-033	6160	M	63	AML with mutated NPM1	Primary	BM	99	46, XY	pos	pos
2-034	6395	M	55	AML with mutated NPM1	Primary	BM	80	46, XY	neg	pos
2-035	6498	F	42	AML with mutated NPM1	Primary	PB	94	46, XX	pos	pos
2-036[4]	6588	F	37	AML with myelodysplasia-related changes	Primary	BM	36	46, XX	pos	neg
2-037	6619	F	62	AML with myelodysplasia-related changes	Primary	BM	20	no metaphases	neg	neg
2-038	6711	M	32	AML NOS without maturation	Primary	BM	94	47, XY, +10, der(16)t(16;21)del(16)(q12.1q24),	neg	neg

								der(21)t(16;21), (p11;q22).ish der(16)t(16;21) del(16)(q12.1q24)(3'CBFB-, 5'CBFB-,TUBB3+), der(21)t(16;21)(p11;q22)(3'MYH11 +,5'MYH11+)		
2-039	6727	F	59	AML NOS without maturation	Primary	BM	90	46, XX, del(9)(q1?3q3?4)	neg	neg
2-040[4]	6588	F	38	AML with myelodysplasia-related changes	Relapse	PB	65	46, XX, t(1;16)(p22;q12)	pos	neg
2-041	7015	F	73	AML with mutated NPM1	Primary	BM	90	46, XX	neg	pos
2-043	8388	F	52	AML with inv(16); *CBFB-MYH11*	Primary	PB	90	47, XX, del(7)(q31q36), +8, inv(16)(p13q22)	neg	neg
2-044	9006	M	42	AML with inv(16); *CBFB-MYH11*	Primary	BM	63	46, XY, inv(16)(p13q22)	neg	neg
2-045	9217	M	49	AML NOS without maturation	Primary	BM	92	46, XY	pos	neg
2-046	9263	F	61	AML with mutated NPM1	Primary	BM	94	46, XX	pos	pos
2-047	9388	F	22	AML NOS without maturation	Primary	BM	92	46, XX, t(6;11)(q27;q23)	neg	neg
2-048	9423	M	39	AML with mutated NPM1	Primary	BM	97	46, XY	neg	pos
2-049	9448	F	37	AML with mutated NPM1	Primary	PB	77	46, XX	pos	pos
2-050	9596	M	53	Therapy-related AML	Primary	BM	60	45, XY, -7	neg	neg
2-051	10024	M	68	AML with mutated NPM1	Primary	BM	98	46, XY	neg	pos
2-052	10146	M	60	AML with t(6;9); *DEK-NUP214*	Primary	PB	66	46, XY, t(6;9)(p22;q34)	pos	neg
2-053	10170	M	27	AML with inv(16); *CBFB-MYH11*	Primary	PB	75	46, XY, inv(16)(p13q22)	neg	neg
2-054	10189	F	71	AML NOS without maturation	Primary	BM	80	46, XX	neg	neg
3-001	4768	F	55	AML with t(9;11); *KMT2A-MLLT3*	Primary	BM	57	46, XX, t(9;11)(p22;q23)	neg	neg
3-002	5233	F	38	AML with t(8;21); *RUNX1-RUNX1T1*	Primary	BM	55	45, X, -X, t(8;21)(q22;q22)	neg	neg
3-003	5818	M	41	AML NOS without maturation	Primary	BM	52	45, XY, del(9)(q13q22)	neg	neg
3-004	5827	M	60	AML with myelodysplasia-related changes	Primary	BM	20	46, XY, idic(14)(p11)	neg	neg
3-005	5904	M	45	AML with mutated NPM1	Primary	BM	93	46, XY	pos	pos
3-006	5905	M	57	AML NOS without maturation	Primary	BM	55	47, XY, +X	neg	neg
3-007	5992	M	54	AML with t(8;21); *RUNX1-RUNX1T1*	Primary	BM	24	47, XY, +4, t(8;21)(q22;q22)	neg	neg
3-008	6033	F	50	AML with t(9;11); *KMT2A-MLLT3*	Primary	BM	43	46, XX, t(9;11)(p21;q23)	neg	neg
3-009	6089	M	60	AML NOS without maturation	Primary	BM	85	46, XY	pos	pos
3-010	6095	F	59	Acute monoblastic/monocytic leukemia	Primary	BM	20	47, XX, +8, t(11;17)(q23;q21)	neg	neg
3-011	6143	M	60	AML with mutated NPM1	Primary	BM	91	46, XY	pos	pos
3-012	7012	M	54	AML with mutated NPM1	Primary	BM	90	46, XY	pos	pos
3-013	7170	F	66	AML with mutated NPM1	Primary	BM	32	46, XX	neg	pos
3-014	7255	F	44	AML with inv(16); *CBFB-MYH11*	Primary	BM	35	47, XX, +X, inv(16)(p13q22)	neg	neg
3-015	8156	M	58	AML NOS with maturation	Primary	BM	51	47, XY, dup(1)(q1?1q4?2),+8[7] / 46, XY,-17, +mar1.ish (WCP17+)[3] / 46, XY, 17, +mar2.ish(WCP17+)[4]	neg	neg
3-016	8228	M	59	AML NOS without maturation	Primary	BM	77	49, XY, +8, +11, +21	neg	neg
3-017	8278	F	60	Acute myelomonocytic leukemia	Primary	BM	50	46, XX	neg	neg
3-018	8334	M	59	AML NOS with maturation	Primary	BM	23	46, XY	neg	neg
3-019	8333	F	37	AML with mutated NPM1	Primary	BM	75	46, XX	neg	pos
3-020	8391	F	72	AML with mutated NPM1	Relapse	BM	29	47, XX, +8	pos	pos
3-021[5]	8390	F	55	AML with mutated NPM1	Primary	PB	20	46, XX	neg	pos
3-022	8524	F	30	AML with myelodysplasia-related changes	Primary	BM	43	45, XX, der(3)t(1;3)(q3?1;q2?5), -7	neg	neg
3-023	8861	M	76	AML with mutated NPM1	Primary	BM	94	46, XY, t(1;10)(q31;q2?6)	neg	pos
3-024	9516	M	65	AML with myelodysplasia-related changes	Primary	BM	25	45, X, -Y	neg	neg
3-025	9691	M	47	AML with inv(16); *CBFB-MYH11*	Primary	BM	95	46, XY, t(9;11;17)(q22;q13;p13), inv(16)(p13q22)	neg	neg
3-026	9750	M	64	AML NOS with maturation	Primary	BM	45	46, XY	pos	neg
3-027	9770	M	65	AML with mutated NPM1	Primary	BM	70	46, XY	pos	pos
3-028	9811	F	30	Acute monoblastic/monocytic leukemia	Primary	BM	80	47, XX, +8	pos	neg
3-029	9978	F	47	AML with inv(3); *GATA2, MECOM*	Primary	BM	60	No metaphases. FISH: EVI1 break	neg	neg
3-030	10232	M	69	AML with myelodysplasia-related changes	Primary	BM	25	48, XY, +8, +15	neg	neg
3-031	10297	M	71	AML with myelodysplasia-related changes	Primary	BM	70	46, XY, del(5)(q21q34)	neg	neg
3-032	10328	F	58	AML with mutated NPM1	Primary	PB	90	47, XX, add(5)(p15), +8	neg	pos
3-033	10381	F	35	Acute myelomonocytic leukemia	Primary	PB	23	45, X, -X	neg	neg
3-034	10418	M	53	AML with mutated NPM1	Primary	BM	98	46, XY	neg	pos
3-035	10437	M	49	AML with mutated NPM1	Primary	BM	96	46, XY	pos	pos
3-037	10594	F	64	AML with mutated NPM1	Primary	BM	80	46, XX	pos	pos
3-038[5]	8390	F	60	Therapy-related AML	Primary	BM	13	48, XX, -7, +10, +19, +21	neg	neg
3-039	10811	M	48	AML with inv(16); *CBFB-MYH11*	Primary	BM	68	46, XY, add(7)(q?), inv(16)(p13q22)	pos	neg
3-040	10831	M	50	AML NOS with maturation	Primary	BM	48	46, XY	neg	neg
3-041	10833	F	51	AML with mutated NPM1	Primary	BM	80	46, XX	pos	pos
3-042	7055	M	56	AML with myelodysplasia-related changes	Primary	BM	25	46, XY	neg	neg

[1] RNAseq analysis was performed in 3 batches as indicated by the first digit of each sample.

[2, 3, 4] Three pairs of primary and relapsed AML samples.

[5] A pair of primary AML and presumed subsequent therapy-related AML in the same patient.

[6] Recurrent fusion transcripts *CBFB-MYH11* inv(16)(p13q22), *KMT2A-MLLT3* t(9;11)(p21;q23), *RUNX1-RUNX1T1* t(8;21)(q22;q22) and *PML-RARA* t(15;17)(q24;q21) as well as chromosomal translocation *GATA2, MECOM* inv(3)(q21q26) were detected by fluorescence in situ hybridization (FISH) on 200 interphases per probe set.

[7] BM, bone marrow; PB, peripheral blood.

[8] AML karyotype was determined by metaphase cytogenetics of at least 20 metaphases per case.

[9] Genotyping for NPM1 hotspot mutations and FLT3-ITD was routinely performed by the accredited LUMC Laboratory of Special Hematology by PCR on genomic DNA followed by electrophoretic fragment size analysis.

Table SII. Quality control of RNAseq data.

sample	num_raw fragments	num_qc fragments	num_total_ reads	Proportion reads aligned	proportion reads aligned pairs	avg_read length	Median insert size	num_total bases	Proportion mrna bases	Proportion intronic bases	Proportion intergenic bases
1-001	54788265	53613331	107248211	0.94	0.94	123.85	167	13282804791	0.83	0.14	0.031
1-002	53075245	51969312	103961552	0.95	0.95	123.81	169	12871338092	0.80	0.17	0.030
1-003	53864742	52731725	105485637	0.95	0.95	123.86	169	13065206475	0.76	0.20	0.042
1-004	59063396	57727265	115479999	0.94	0.94	123.90	167	14308267196	0.82	0.15	0.030
1-005	61187215	59763235	119551529	0.95	0.94	123.87	170	14809066970	0.83	0.14	0.030
1-006	70668030	68870967	137780396	0.95	0.95	123.85	168	17064602606	0.80	0.17	0.031
1-007	55346361	54193453	108411531	0.93	0.93	123.93	165	13435214910	0.81	0.16	0.037
1-008	66197432	64909956	129850843	0.96	0.96	124.02	168	16104746802	0.80	0.17	0.029
2-001	56506334	55591220	111202194	0.95	0.95	123.33	173	13714190090	0.83	0.14	0.027
2-002	57280209	56242701	112511142	0.94	0.93	123.64	177	13910591773	0.76	0.21	0.031
2-003	57821980	56893162	113805150	0.93	0.93	123.32	176	14034305270	0.73	0.24	0.033
2-004	53495171	52681112	105387311	0.94	0.94	123.58	174	13023576646	0.86	0.11	0.027
2-005	62540695	61479035	122989969	0.95	0.95	123.70	169	15214437493	0.87	0.11	0.022
2-006	64236869	63200016	126425295	0.94	0.94	123.49	175	15612222336	0.79	0.17	0.037
2-007	53098093	52236602	104500304	0.94	0.93	123.64	172	12920631494	0.87	0.10	0.027
2-008	59042306	57969023	115964551	0.95	0.94	123.52	173	14323391338	0.80	0.17	0.030
2-009	50992079	50170952	100365594	0.93	0.93	123.71	165	12416636439	0.84	0.12	0.032
2-010	65099763	64021562	128075199	0.95	0.95	123.40	163	15805031281	0.83	0.14	0.024
2-011	73743726	72409403	144854215	0.94	0.94	123.70	170	17918060885	0.80	0.17	0.029
2-012	67096731	66070319	132168330	0.94	0.94	123.60	168	16336605515	0.83	0.15	0.026
2-013	62658949	61576479	123175793	0.94	0.93	123.59	177	15223008708	0.82	0.15	0.032
2-014	52593372	51581084	103180228	0.78	0.78	123.56	167	12748875404	0.83	0.14	0.028
2-015	61512621	60502601	121032998	0.93	0.93	123.38	172	14932730855	0.83	0.14	0.032
2-016	51446976	50588327	101198501	0.94	0.94	123.35	173	12482697291	0.87	0.11	0.026
2-018	60224629	59274153	118571640	0.94	0.94	123.35	165	14625570924	0.72	0.23	0.053
2-019	85577511	84334072	168706819	0.96	0.95	124.10	163	20936045029	0.85	0.12	0.029
2-020	65608004	64526871	129084997	0.94	0.94	123.44	162	15933769735	0.82	0.15	0.029
2-021	69052152	67971793	135978779	0.93	0.93	123.30	165	16766123665	0.84	0.14	0.027
2-022	53165468	52333635	104692895	0.96	0.96	123.97	160	12979187074	0.84	0.13	0.027
2-023	70094054	68770687	137572258	0.94	0.94	123.16	170	16943761374	0.79	0.17	0.038
2-024	65824057	64716945	129465744	0.95	0.95	123.92	163	16043645622	0.84	0.13	0.026
2-025	67282583	66137463	132305455	0.93	0.93	123.28	163	16310074314	0.86	0.11	0.030
2-027	68055617	66866063	133760110	0.96	0.96	123.39	163	16504966201	0.73	0.23	0.035
2-028	51405680	50599116	101219282	0.95	0.95	123.91	165	12541820293	0.84	0.14	0.026
2-029	72743917	71613897	143258798	0.96	0.96	124.07	163	17774685182	0.83	0.15	0.023
2-030	52278151	51354414	102730947	0.95	0.95	123.67	163	12704664569	0.82	0.15	0.036
2-031	56091225	55139771	110303186	0.94	0.93	123.66	166	13639694704	0.82	0.15	0.031
2-032	51047572	50203249	100426680	0.93	0.93	123.82	172	12434347703	0.77	0.19	0.038
2-033	58622449	57735149	115494969	0.95	0.95	123.89	166	14309217897	0.81	0.15	0.037
2-034	81508674	80169841	160372609	0.96	0.96	123.17	167	19753385968	0.83	0.14	0.028
2-035	67361084	66270584	132569286	0.95	0.95	123.92	163	16428547132	0.69	0.26	0.051
2-036	73002305	71822945	143681853	0.93	0.93	123.98	161	17814008303	0.77	0.19	0.035
2-037	55154252	54370907	108767647	0.94	0.95	123.68	163	13452436847	0.79	0.18	0.030
2-038	49643625	48891031	97801049	0.93	0.93	123.96	160	12123701712	0.77	0.19	0.033
2-039	55701447	54891057	109804914	0.94	0.94	124.07	160	13623522546	0.83	0.13	0.032
2-040	68419629	67379627	134790642	0.94	0.94	123.12	167	16595306380	0.83	0.14	0.036
2-041	52605994	51777425	103574129	0.95	0.95	124.02	162	12845612847	0.82	0.15	0.024
2-043	49868994	49185326	98391810	0.94	0.94	123.95	162	12196096087	0.78	0.19	0.032
2-044	53991414	53213714	106452709	0.94	0.94	123.71	161	13168990553	0.86	0.12	0.025
2-045	51265509	50462809	100952097	0.93	0.93	124.00	164	12518515531	0.81	0.17	0.026
2-046	56944242	56060382	112145666	0.95	0.95	123.76	162	13879365217	0.83	0.14	0.026
2-047	49025083	48313248	96650064	0.93	0.93	123.88	161	11972882123	0.83	0.14	0.034
2-048	51073901	50351791	100724042	0.92	0.91	123.89	158	12479013426	0.84	0.13	0.028
2-049	49872940	49145220	98307022	0.94	0.94	123.92	162	12182371921	0.86	0.11	0.024
2-050	49697953	48943181	97908977	0.93	0.93	123.99	160	12139486114	0.84	0.13	0.029
2-051	55208849	54361755	108745947	0.94	0.94	123.94	164	13477848064	0.79	0.17	0.036

2-052	57596568	56525637	113075546	0.93	0.93	123.57	163	13973158591	0.84	0.14	0.029
2-053	75023217	73884797	147805514	0.94	0.94	124.01	163	18328798752	0.85	0.13	0.024
2-054	57384643	56619199	113262879	0.92	0.92	124.17	161	14063974445	0.81	0.16	0.033
3-001	52693870	51820595	103659832	0.94	0.94	123.24	153	12774598770	0.86	0.12	0.026
3-002	51276208	50337961	100699745	0.93	0.93	123.58	152	12444322586	0.85	0.12	0.027
3-003	50071690	49389172	98797507	0.95	0.95	123.68	151	12219077782	0.87	0.10	0.028
3-004	50781816	50119289	100258711	0.95	0.95	123.67	153	12398825316	0.83	0.14	0.026
3-005	53280275	52233351	104486687	0.95	0.95	123.45	152	12899004771	0.81	0.16	0.032
3-006	54427152	53585671	107197691	0.95	0.94	123.53	154	13241887450	0.83	0.14	0.029
3-007	53348958	52517130	105053120	0.95	0.94	123.66	151	12990586544	0.89	0.09	0.023
3-008	73716517	72568509	145164059	0.95	0.95	123.54	154	17933342116	0.80	0.17	0.030
3-009	68943807	67744669	135511083	0.95	0.95	123.52	149	16738035423	0.78	0.18	0.039
3-010	51212748	50452254	100923621	0.94	0.94	123.67	150	12481096240	0.85	0.12	0.027
3-011	51651751	50869417	101757986	0.95	0.95	123.50	152	12567173340	0.86	0.11	0.030
3-012	61175770	60140402	120304899	0.95	0.94	123.67	152	14877660939	0.85	0.12	0.030
3-013	56726988	55853626	111732282	0.95	0.95	123.56	152	13806042804	0.85	0.12	0.026
3-014	52517794	51676464	103376769	0.96	0.96	123.61	153	12778228965	0.86	0.11	0.024
3-015	52345490	51448743	102920197	0.95	0.95	123.55	152	12715610283	0.80	0.16	0.034
3-016	60983733	60059701	120154802	0.94	0.94	123.29	154	14813668446	0.78	0.19	0.027
3-017	59617322	58641675	117306726	0.95	0.95	123.44	150	14480399479	0.85	0.12	0.024
3-018	50433250	49688464	99397881	0.95	0.95	123.45	151	12271158369	0.83	0.14	0.029
3-019	55644436	54693377	109409004	0.95	0.95	123.44	153	13505775375	0.79	0.18	0.028
3-020	53109065	52186743	104400446	0.94	0.94	123.51	154	12894964817	0.79	0.19	0.029
3-021	55601520	54535952	109094019	0.95	0.95	123.40	154	13462279338	0.81	0.16	0.028
3-022	51610206	50530261	101078815	0.96	0.95	123.52	151	12485515754	0.80	0.17	0.035
3-023	51680125	50918651	101868266	0.94	0.94	123.64	152	12595202498	0.79	0.18	0.030
3-024	55043453	54262574	108551342	0.96	0.96	123.48	153	13404458026	0.73	0.24	0.029
3-025	56669383	55913456	111850763	0.95	0.94	123.70	152	13836183097	0.87	0.11	0.021
3-026	68421663	67377624	134781737	0.95	0.94	123.89	153	16698486905	0.82	0.15	0.030
3-027	64506711	63685395	127398266	0.94	0.94	123.99	153	15796396507	0.85	0.12	0.029
3-028	67922615	67079769	134189759	0.93	0.93	123.99	154	16638085793	0.85	0.12	0.029
3-029	64180589	63284193	126594559	0.95	0.95	123.96	153	15692048156	0.84	0.13	0.031
3-030	60882308	60078433	120181141	0.96	0.95	123.91	155	14891563505	0.83	0.14	0.031
3-031	64946578	64012042	128051805	0.95	0.95	123.84	154	15858172077	0.81	0.15	0.034
3-032	84482231	83460133	166960125	0.95	0.95	124.12	154	20722663993	0.83	0.14	0.028
3-033	57977219	57097996	114219954	0.96	0.96	123.83	156	14143885179	0.82	0.15	0.025
3-034	70809403	69843449	139712362	0.94	0.94	123.95	153	17317144601	0.87	0.10	0.029
3-035	69381515	68321472	136671779	0.95	0.95	123.62	156	16895415499	0.74	0.22	0.038
3-037	75282940	74299148	148624447	0.96	0.96	123.89	156	18412998865	0.81	0.16	0.037
3-038	77419245	76225741	152487359	0.95	0.95	123.87	163	18888817283	0.86	0.11	0.023
3-039	62188369	61221821	122470593	0.95	0.95	123.86	152	15168607460	0.86	0.12	0.025
3-040	58196485	57288503	114599655	0.94	0.94	123.62	160	14166312677	0.84	0.13	0.026
3-041	65105632	64207973	128443731	0.94	0.94	123.93	153	15918553488	0.91	0.07	0.020
3-042	68458272	67413990	134855727	0.95	0.94	123.92	154	16711626552	0.83	0.13	0.033

Table SIII. Detection of fusion transcripts with corresponding structural abnormalities by metaphase cytogenetics.

Sample	Fusion Genes	JR[1]	SF[2]	Left GRCh38[3]	Right GRCh38[4]	Validation[5]	Fusion Protein[6]	Cytogenetics
1-001	KMT2A-MLLT3	9	8	chr11:118484314	chr9:20365744	Sanger	NRF-NRF	t(9;11)(p21;q23)
	KMT2A-MLLT3	12	8	chr11:118484975	chr9:20365744	Sanger	NRF-NRF	
1-004	CBFB-MYH11	69	12	chr16:67082308	chr16:15721051	Sanger	NRF-NRF	inv(16)(p13q22)
1-007	RUNX1-RUNX1T1	32	9	chr21:34859474	chr8:92017363	Sanger	NRF-NRF	t(8;21)(q22;q22)
	RUNX1-RUNX1T1	4	9	chr21:34859474	chr8:92062709	Sanger	NRF-***ARF***	
2-004	CBFB-MYH11	67	8	chr16:67082308	chr16:15721051	Sanger	NRF-NRF	inv(16)(p13q22)
2-005	KMT2A-MLLT3	8	1	chr11:118484314	chr9:20414425	NGS	NRF-NRF	t(9;11)(p21;q23)
	KMT2A-MLLT3	3	1	chr11:118484975	chr9:20414425	gel electrophoresis	NRF-NRF	
2-006	PML-RARA	40	9	chr15:74023408	chr17:40348316	Sanger	NRF-NRF	t(15;17)(q24;q21)
	RARA-PML	87	16	chr17:40331396	chr15:74024857	Sanger	NRF-NRF	
2-007	CBFB-MYH11	42	10	chr16:67082308	chr16:15721051	gel electrophoresis	NRF-NRF	inv(16)(p13q22)
2-010	CBFB-MYH11	41	4	chr16:67082308	chr16:15724992	gel electrophoresis	NRF-NRF	inv(16)(p13q22)
2-014	PML-RARA	1	3	chr15:74023408	chr17:40348316	not detected	NRF-NRF	t(15;17)(q24;q21)
	PML-RARA	4	3	chr15:74024927	chr17:40348316	gel electrophoresis	NRF-***ARF***	
	PML-RARA	7	3	chr15:74033231	chr17:40334191	Sanger	NRF-***ARF***	
	RARA-PML	10	2	chr17:40331396	chr15:74034478	Sanger	NRF-NRF	
2-022	KMT2A-MLLT1	8	3	chr11:118484314	chr19:6270759	Sanger	NRF-NRF	t(11;19)(q23;p13)
2-038	ERG-FUS	12	5	chr21:38390995	chr16:31188325	Sanger	NRF-NRF	t(16;21)(p11;q22)
	FUS-ERG	46	9	chr16:31186836	chr21:38383923	Sanger	NRF-NRF	
2-043	CBFB-MYH11	30	3	chr16:67082308	chr16:15721051	n.d.	NRF-NRF	inv(16)(p13q22)
2-044	CBFB-MYH11	28	5	chr16:67082308	chr16:15721051	n.d.	NRF-NRF	inv(16)(p13q22)
2-047	KMT2A-MLLT4	17	3	chr11:118482495	chr6:167864551	gel electrophoresis	NRF-NRF	t(6;11)(q27;q23)
2-052	DEK-NUP214	12	0	chr6:18236452	chr9:131159383	Sanger	NRF-NRF	t(6;9)(p22;q34)
	NUP214-DEK	6	1	chr9:131151894	chr6:18226242	Sanger	NRF-NRF	
2-053	CBFB-MYH11	59	7	chr16:67082308	chr16:15721051	n.d.	NRF-NRF	inv(16)(p13q22)
3-001	KMT2A-MLLT3	3	0	chr11:118484314	chr9:20365744	Sanger	NRF-NRF	t(9;11)(p21;q23)
3-002	RUNX1-RUNX1T1	51	12	chr21:34859474	chr8:92017363	Sanger	NRF-NRF	t(8;21)(q22;q22)
	RUNX1-RUNX1T1	9	12	chr21:34859474	chr8:92062709	Sanger	NRF-***ARF***	
3-007	RUNX1-RUNX1T1	19	5	chr21:34859474	chr8:92017363	Sanger	NRF-NRF	t(8;21)(q22;q22)
	RUNX1-RUNX1T1	5	5	chr21:34859474	chr8:92062709	Sanger	NRF-***ARF***	
3-008	KMT2A-MLLT3	15	2	chr11:118484314	chr9:20365744	Sanger	NRF-NRF	t(9;11)(p21;q23)
	KMT2A-MLLT3	4	2	chr11:118484975	chr9:20365744	Sanger	NRF-NRF	
3-010	KMT2A-MLLT6	3	0	chr11:118481953	chr17:38717270	NGS	NRF-***ARF***	t(11;17)(q23;q21)
	KMT2A-MLLT6	2	0	chr11:118480238	chr17:38717432	n.d.	NRF-NRF	
3-014	CBFB-MYH11	41	8	chr16:67082308	chr16:15721051	n.d.	NRF-NRF	inv(16)(p13q22)
3-025	CBFB-MYH11	35	4	chr16:67082308	chr16:15721051	n.d.	NRF-NRF	inv(16)(p13q22)
3-039	CBFB-MYH11	46	3	chr16:67082308	chr16:15727054	gel electrophoresis	NRF-NRF	inv(16)(p13q22)
	CBFB-MYH11	5	3	chr16:67082339	chr16:15727054	gel electrophoresis	NRF-***ARF***	

[1] JR; junction reads; single reads containing sequences of two different genes.

[2] SF; spanning fragments; read pairs containing sequences of two different genes.

[3] Left GRCh38; position of the 3’ end of the upstream gene in the fusion transcript after alignment to the GRCh38 genome.

[4] Right GRCh38; position of the 5’ end of the downstream gene in the fusion transcript after alignment to the GRCh38 genome.

[5] Fusion transcripts were validated by RT-PCR and fragment size analysis by gel electrophoresis and in some instances by Sanger or NGS. Primers used for validation are shown in Table SV. n.d.; not determined.

[6] Indicated whether the downstream fusion partner in the transcript is translated in the normal reading frame (NRF) or alternative reading frame (ARF).

Table SIV. Sensitivity of HAMLET to detect fusion genes at different sequencing depths[1].

Sample	Fusion Genes	Left GRCh38[2]	Right GRCh38[3]	20M	30M	40M	47.5M
1-001	KMT2A-MLLT3	chr11:118484314	chr9:20365744	+	n.d.	n.d.	n.d.
	KMT2A-MLLT3	chr11:118484975	chr9:20365744	+	n.d.	n.d.	n.d.
1-004	CBFB-MYH11	chr16:67082308	chr16:15721051	+	n.d.	n.d.	n.d.
1-007	RUNX1-RUNX1T1	chr21:34859474	chr8:92017363	+	n.d.	n.d.	n.d.
	RUNX1-RUNX1T1	chr21:34859474	chr8:92062709	+	n.d.	n.d.	n.d.
2-004	CBFB-MYH11	chr16:67082308	chr16:15721051	+	n.d.	n.d.	n.d.
2-005	KMT2A-MLLT3	chr11:118484314	chr9:20414425	-	+	n.d.	n.d.
	KMT2A-MLLT3	chr11:118484975	chr9:20414425	-	-	-	+
2-006	PML-RARA	chr15:74023408	chr17:40348316	+	n.d.	n.d.	n.d.
	RARA-PML	chr17:40331396	chr15:74024857	+	n.d.	n.d.	n.d.
2-007	CBFB-MYH11	chr16:67082308	chr16:15721051	+	n.d.	n.d.	n.d.
2-010	CBFB-MYH11	chr16:67082308	chr16:15724992	+	n.d.	n.d.	n.d.
2-014	PML-RARA	chr15:74023408	chr17:40348316	-	-	+	n.d.
	PML-RARA	chr15:74024927	chr17:40348316	+	n.d.	n.d.	n.d.
	PML-RARA	chr15:74033231	chr17:40334191	-	-	+	n.d.
	RARA-PML	chr17:40331396	chr15:74034478	+	n.d.	n.d.	n.d.
2-022	KMT2A-MLLT1	chr11:118484314	chr19:6270759	+	n.d.	n.d.	n.d.
2-038	ERG-FUS	chr21:38390995	chr16:31188325	+	n.d.	n.d.	n.d.
	FUS-ERG	chr16:31186836	chr21:38383923	+	n.d.	n.d.	n.d.
2-043	CBFB-MYH11	chr16:67082308	chr16:15721051	+	n.d.	n.d.	n.d.
2-044	CBFB-MYH11	chr16:67082308	chr16:15721051	+	n.d.	n.d.	n.d.
2-047	KMT2A-MLLT4	chr11:118482495	chr6:167864551	+	n.d.	n.d.	n.d.
2-052	DEK-NUP214	chr6:18236452	chr9:131159383	+	n.d.	n.d.	n.d.
	NUP214-DEK	chr9:131151894	chr6:18226242	+	n.d.	n.d.	n.d.
2-053	CBFB-MYH11	chr16:67082308	chr16:15721051	+	n.d.	n.d.	n.d.
3-001	KMT2A-MLLT3	chr11:118484314	chr9:20365744	-	-	+	n.d.
3-002	RUNX1-RUNX1T1	chr21:34859474	chr8:92017363	+	n.d.	n.d.	n.d.
	RUNX1-RUNX1T1	chr21:34859474	chr8:92062709	+	n.d.	n.d.	n.d.
3-007	RUNX1-RUNX1T1	chr21:34859474	chr8:92017363	+	n.d.	n.d.	n.d.
	RUNX1-RUNX1T1	chr21:34859474	chr8:92062709	+	n.d.	n.d.	n.d.
3-008	KMT2A-MLLT3	chr11:118484314	chr9:20365744	+	n.d.	n.d.	n.d.
	KMT2A-MLLT3	chr11:118484975	chr9:20365744	+	n.d.	n.d.	n.d.
3-010	KMT2A-MLLT6	chr11:118481953	chr17:38717270	-	+	n.d.	n.d.
	KMT2A-MLLT6	chr11:118480238	chr17:38717432	+	n.d.	n.d.	n.d.
3-014	CBFB-MYH11	chr16:67082308	chr16:15721051	+	n.d.	n.d.	n.d.
3-025	CBFB-MYH11	chr16:67082308	chr16:15721051	+	n.d.	n.d.	n.d.
3-039	CBFB-MYH11	chr16:67082308	chr16:15727054	+	n.d.	n.d.	n.d.
	CBFB-MYH11	chr16:67082339	chr16:15727054	+	n.d.	n.d.	n.d.

[1] Number of read pairs were downscaled for each sample to 47.5, 40, 30 and 20 million. HAMLET was first run on all samples with 20 million read pairs followed by analysis with 30, 40 and 47.5 million read pairs for those fusions that were not detected with 20 million read pairs. The sequential analysis was stopped upon detection of the fusion. Fusions that were detected (+) are indicated in blue, and fusions that were not detected (-) are shown in orange. n.d.; not determined.

[2] Left GRCh38; 3' end of the upstream gene in the fusion transcript after alignment to the GRCh38 genome.

[3] Right GRCh38; 5' end of the downstream gene in the fusion transcript after alignment.

Table SV. Detection of fusion transcripts without corresponding structural abnormalities by metaphase cytogenetics.

Sample	Fusion Genes	JR[1]	SF[2]	Left GRCh38[3]	Right GRCh38[4]	Validation[5]	Fusion Protein[6]	Cytogenetics
1-001	YWHAZ--KLF10	2	0	chr8:100951929	chr8:102652397	not detected	No[7]	46,XX,t(9;11)(p21;q23)
1-002	TPM4--KLF2	5	1	chr19:16076697	chr19:16326856	n.d.	NRF-*ARF*	46,XX
1-003	TPM4--KLF2	6	0	chr19:16076697	chr19:16326856	n.d.	NRF-*ARF*	46,XX
1-004	TPM4--KLF2	7	1	chr19:16076697	chr19:16326856	n.d.	NRF-*ARF*	46,XY,inv(16)(p13q22)
	C15orf57--CBX3	3	1	chr15:40561981	chr7:26201745	n.d.	No	
1-005	REXO1--LUC7L3	21	6	chr19:1848202	chr17:50736960	n.d.	NRF-*ARF*	46,XY,inv(3)(q21q26), del(7)(q21), -17,der(19) t(17;19)(q2?2;q13)/ 46-48,XY,del(2)(p21)[8],-3[9],del(3)(q23)[8],+6[6],-7[8], del(11)(p1?3[9], add(12)(p11)[9], inv(15)(q?13q?23)[6],+ring[8], +mar1[8]
	STRAP--BCL6	16	4	chr12:15882819	chr3:187734907	n.d.	NRF-*ARF*	
	RBM6--DOCK3	4	2	chr3:49975392	chr3:51356407	n.d.	NRF-*ARF*	
	C15orf57--CBX3	4	2	chr15:40561981	chr7:26201745	n.d.	No	
	TP53--ZNF398	4	1	chr17:7687377	chr7:149166158	n.d.	No	
	EXT2--CRY2	3	0	chr11:44171742	chr11:45855982	n.d.	NRF-*ARF*	
1-006	TPM4--KLF2	11	0	chr19:16076697	chr19:16326856	gel electrophoresis	NRF-*ARF*	46,X,add(X)(q2?1), add(1)(q4?3), add(3)(p21),add(20)(q11.2)
	C15orf57--CBX3	7	1	chr15:40561981	chr7:26201745	gel electrophoresis	No	
	B3GNTL1—METRNL	3	1	chr17:83014454	chr17:83084938	n.d.	NRF-*ARF*	
	B3GNTL1—METRNL	1	1	chr17:83035035	chr17:83084938	n.d.	NRF-*ARF*	
	B3GNTL1—METRNL	1	1	chr17:83005115	chr17:83093167	n.d.	NRF-*ARF*	
	B3GNTL1—METRNL	1	1	chr17:83035035	chr17:83093167	n.d.	NRF-*ARF*	
1-007	TPM4--KLF2	2	1	chr19:16076697	chr19:16326856	n.d.	NRF-*ARF*	46,XY,t(8;21)(q22;q22)
1-008	C15orf57--CBX3	11	4	chr15:40561981	chr7:26201745	gel electrophoresis	No	46,XY,t(3;8)(q26;q24)
	C15orf57--CBX3	4	4	chr15:40562772	chr7:26201769	gel electrophoresis	NRF-*ARF*	
2-002	ZEB2--CXCR4	6	0	chr2:144519939	chr2:136115912	gel electrophoresis	No	46,XY
	TPM4--KLF2	2	0	chr19:16076697	chr19:16326856	Sanger	NRF-*ARF*	
2-003	NSD1--NUP98	12	4	chr5:177212195	chr11:3735324	gel electrophoresis	NRF-NRF	46,XY
	NUP98--NSD1	9	3	chr11:3744509	chr5:177235821	gel electrophoresis	NRF-NRF	
	NUP98--NSD1	2	3	chr11:3753316	chr5:177235821	NGS	NRF-NRF	
	HIPK2--TBXAS1	2	0	chr7:139777605	chr7:139872235	n.d.	NRF-*ARF*	
2-005	BRI3BP—UBC	6	0	chr12:124994003	chr12:124913774	n.d.	NRF-NRF	46,XY,t(9;11)(p21;q23)
2-007	C15orf57--CBX3	3	2	chr15:40561981	chr7:26201745	n.d.	No	46,XY,inv(16)(p13q22)
2-008	NCOR2—UBC	3	2	chr12:124419957	chr12:124913774	gel electrophoresis	NRF-NRF	47,XY,+11
	NCOR2—UBC	2	2	chr12:124398119	chr12:124913774	n.d.	NRF-*ARF*	
	NCOR2—UBC	1	2	chr12:124449815	chr12:124913774	n.d.	NRF-*ARF*	
	NCOR2—UBC	1	2	chr12:124372022	chr12:124913774	n.d.	NRF-*ARF*	
	NCOR2—UBC	1	2	chr12:124437930	chr12:124913774	n.d.	NRF-*ARF*	
	NCOR2—UBC	1	2	chr12:124362126	chr12:124913774	n.d.	NRF-*ARF*	
	NCOR2—UBC	1	2	chr12:124400501	chr12:124913774	gel electrophoresis	NRF-*ARF*	
	NCOR2—UBC	1	2	chr12:124535565	chr12:124913774	not detected	No	
2-011	MGAT1--GFPT2	31	11	chr5:180808502	chr5:180338600	n.d.	No	46,XX
	MGAT1--GFPT2	20	11	chr5:180808648	chr5:180338600	n.d.	No	
	TPM4--KLF2	6	1	chr19:16076697	chr19:16326856	n.d.	NRF-*ARF*	
2-012	C15orf57--CBX3	6	1	chr15:40561981	chr7:26201745	n.d.	No	46,XX
	TPM4--KLF2	5	1	chr19:16076697	chr19:16326856	n.d.	NRF-*ARF*	
2-013	ZCCHC8--RSRC2	148	24	chr12:122498827	chr12:122505706	n.d.	NRF-*ARF*	46,XY
	C15orf57--CBX3	3	0	chr15:40561981	chr7:26201745	n.d.	No	
2-019	TAOK1--ATP5H	219	19	chr17:29522519	chr17:75042659	n.d.	NRF-NRF	45-46,XX,?inv(3)(p?q?),-4, add(5)(q1?2),der(9), t(4;9)(q21;q34),?dic(11;?)(p1?2;?) , add(12)(p13), der(12)add(12)(p?)del(12)(q22), add(14)(q13),-17, del(17)(q21) +1-2mar[19]/46,XX[1]
	SEC61A1--C3orf62	51	4	chr3:128052902	chr3:49274140	n.d.	NRF-ARF	
	WDR82--GXYLT2	34	8	chr3:52278201	chr3:72908367	n.d.	NRF-NRF	
	FYCO1--EDEM1	33	3	chr3:45984856	chr3:5215829	n.d.	NRF-ARF	
	WDR82--GXYLT2	29	8	chr3:52270712	chr3:72908367	n.d.	NRF-ARF	
	LARS2--PLCL2	11	3	chr3:45400373	chr3:17089733	n.d.	NRF-NRF	
	YPEL2--TACO1	8	2	chr17:59397488	chr17:63604534	n.d.	No	
	ZFYVE1--NFXL1	3	1	chr14:73024026	chr4:47879117	n.d.	NRF-ARF	

	MEPCE--TRIM56	3	2	chr7:100433665	chr7:101088891	n.d.	No	
	NFXL1--ZFYVE1	3	0	chr4:47884346	chr14:72998315	n.d.	No	
	ZFYVE1--NFXL1	1	1	chr14:73024026	chr4:47878665	n.d.	NRF-NRF	
2-020	ETV6—LYN	582	72	chr12:11869969	chr8:55953832	gel electrophoresis	NRF-NRF	47, XX, +8, add(8)(q24), del(12)(p13), i(17)(q10)[cp14]
2-021	TPM4--KLF2	1	1	chr19:16076697	chr19:16326856	n.d.	NRF-*ARF*	47,XY,+8
2-022	LRG1—LYZ	3	2	chr19:4537861	chr12:69353759	n.d.	No	46,XX,der(11) t(11;11)(q13;q23) inv(11)(q13q23)t(11;19)(q23;p13), der(11) t(11;11)(q13;q23), del(12)(p13), der(19)t(11;19)(q23;p13)
	LRG1—LYZ	6	2	chr19:4537864	chr12:69353767	n.d.	No	
	TPM4--KLF2	2	0	chr19:16076697	chr19:16326856	gel electrophoresis	NRF-*ARF*	
2-024	TPM4--KLF2	2	0	chr19:16076697	chr19:16326856	gel electrophoresis	NRF-*ARF*	46,XX
2-028	TUBA1A—UBC	3	0	chr12:49188977	chr12:124913774	n.d.	NRF-NRF	46,XY
2-031	NUP98--NSD1	2	1	chr11:3753316	chr5:177235821	NGS	NRF-NRF	46,XY
	NUP98--NSD1	2	1	chr11:3744509	chr5:177235821	gel electrophoresis	NRF-NRF	
2-032	NUP98--NSD1	3	0	chr11:3744509	chr5:177235821	NGS	NRF-NRF	46,XY
	CLEC12A--CHMP1A	2	1	chr12:9982129	chr16:89647331	n.d.	No	
2-036	NCOR2—UBC	2	0	chr12:124535565	chr12:124913774	n.d.	No	46,XX
	TPM4--KLF2	2	0	chr19:16076697	chr19:16326856	n.d.	NRF-*ARF*	
2-037	TPM4--KLF2	3	0	chr19:16076697	chr19:16326856	n.d.	NRF-*ARF*	no metaphases
2-038	TPM4--KLF2	18	2	chr19:16076697	chr19:16326856	gel electrophoresis	NRF-*ARF*	47,XY,+10, der(16)t(16;21)del(16)(q12.1q24), der(21)t(16;21) (p11;q22).ish der(16)t(16;21)del(16)(q12.1q24)(3'CBFB-,5'CBFB-,TUBB3+), der(21)t(16;21)(p11;q22)(3'MYH1 1+,5'MYH11+)
2-043	ATP6V0D1--TERF2	70	6	chr16:67480957	chr16:69361489	n.d.	NRF-*ARF*	47,XX,del(7)(q31q36),+8,inv(16)(p 13q22)
	CTCF--ABCC1	11	3	chr16:67571264	chr16:16009776	n.d.	No	
	CTCF--ABCC1	2	3	chr16:67571264	chr16:16014491	n.d.	No	
2-047	TPM4--KLF2	15	2	chr19:16076697	chr19:16326856	n.d.	NRF-*ARF*	46,XX,t(6;11)(q27;q23)
2-048	TPM4--KLF2	7	0	chr19:16076697	chr19:16326856	n.d.	NRF-*ARF*	46,XY
2-052	C15orf57--CBX3	6	0	chr15:40561981	chr7:26201745	n.d.	No	46,XY,t(6;9)(p22;q34)
2-053	TPM4--KLF2	13	2	chr19:16076697	chr19:16326856	n.d.	NRF-*ARF*	46,XY,inv(16)(p13q22)
	C15orf57--CBX3	10	1	chr15:40561981	chr7:26201745	n.d.	No	
	PIM3--SCO2	2	0	chr22:49961811	chr22:50524424	gel electrophoresis	NRF-*ARF*	
3-002	TPM4--KLF2	5	0	chr19:16076697	chr19:16326856	n.d.	NRF-*ARF*	45,X,t(8;21)(q22;q22)
3-006	TPM4--KLF2	3	1	chr19:16076697	chr19:16326856	n.d.	NRF-*ARF*	47,XY,+Xc
3-008	TPM4--KLF2	2	0	chr19:16076697	chr19:16326856	n.d.	NRF-*ARF*	46,XX, t(9;11)(p21;q23)
3-011	C15orf57--CBX3	3	0	chr15:40561981	chr7:26201745	n.d.	No	46,XY
	MAPKAP1--PBX3	2	0	chr9:125543059	chr9:125797395	n.d.	NRF-*ARF*	
3-012	TPM4--KLF2	4	0	chr19:16076697	chr19:16326856	gel electrophoresis	NRF-*ARF*	46,XY
	C15orf57--CBX3	3	1	chr15:40561981	chr7:26201745	gel electrophoresis	No	
3-013	TMEM44--LSG1	6	1	chr3:194623224	chr3:194644746	n.d.	NRF-NRF	46,XX
3-016	RASA3--ING1	3	1	chr13:114132435	chr13:110719229	n.d.	NRF-NRF	48,XY,+8,+11[6]/49,idem,+21[4]
	CYTH1--USP36	3	0	chr17:78782202	chr17:78838750	n.d.	NRF-*ARF*	
	FUS—SETD1A	2	0	chr16:31180227	chr16:30958717	n.d.	NRF-*ARF*	
	PIM3—TRABD	5	0	chr22:49961811	chr22:50197241	n.d.	NRF-*ARF*	
	NFATC1--CTDP1	1	2	chr18:79451816	chr18:79695225	n.d.	NRF-*ARF*	
	NFATC1--CTDP1	1	2	chr18:79433738	chr18:79753652	n.d.	NRF-*ARF*	
	FBRSL1--NOC4L	1	2	chr12:132560230	chr12:132150981	n.d.	No	
	BSG--TMEM259	1	1	chr19:571579	chr19:1014473	n.d.	NRF-*ARF*	
	BSG--TMEM259	1	1	chr19:572701	chr19:1014473	n.d.	NRF-*ARF*	
	NFATC1--CTDP1	1	2	chr18:79467582	chr18:79695225	n.d.	NRF-*ARF*	
	NFATC1--CTDP1	1	2	chr18:79486630	chr18:79736355	n.d.	NRF-NRF	
	NFATC1--CTDP1	1	2	chr18:79396351	chr18:79695225	n.d.	NRF-*ARF*	
	NFATC1--CTDP1	1	2	chr18:79400479	chr18:79695225	n.d.	NRF-*ARF*	
	NFATC1--CTDP1	1	2	chr18:79400479	chr18:79753652	n.d.	NRF-*ARF*	
3-017	TPM4--KLF2	5	0	chr19:16076697	chr19:16326856	n.d.	NRF-*ARF*	46,XX
3-018	CTCF--NUTF2	26	1	chr16:67571264	chr16:67865102	n.d.	No	

	C15orf57--CBX3	1	2	chr15:40562772	chr7:26201757	NGS	No	46,XY
	C15orf57--CBX3	10	0	chr15:40561981	chr7:26201745	NGS	No	
	ZFPM1--GSE1	1	1	chr16:88489153	chr16:85654278	n.d.	NRF-*ARF*	
3-019	C15orf57--CBX3	10	0	chr15:40561981	chr7:26201745	n.d.	No	46,XX
	TPM4--KLF2	3	1	chr19:16076697	chr19:16326856	n.d.	NRF-*ARF*	
3-020	TPM4--KLF2	5	1	chr19:16076697	chr19:16326856	n.d.	NRF-*ARF*	47,XX,+8
	C15orf57--CBX3	4	2	chr15:40561981	chr7:26201745	n.d.	No	
	SRGN--IRF2BP2	4	0	chr10:69088236	chr1:234607852	n.d.	NRF-NRF	
	B3GNT5--KLHL24	2	0	chr3:183253472	chr3:183643480	n.d.	No	
3-021	TPM4--KLF2	8	0	chr19:16076697	chr19:16326856	n.d.	NRF-*ARF*	46,XX
	PIM3--SCO2	3	1	chr22:49961811	chr22:50524424	Sanger	NRF-*ARF*	
	OAZ1--KLF2	3	0	chr19:2269744	chr19:16326856	n.d.	No	
	GUK1--ARF1	2	0	chr1:228140363	chr1:228097078	n.d.	NRF-*ARF*	
3-022	TPM4--KLF2	9	0	chr19:16076697	chr19:16326856	n.d.	NRF-*ARF*	45,XX,der(3)t(1;3)(q3?1;q2?5),-7
	NCOR2—UBC	2	0	chr12:124419957	chr12:124913774	n.d.	NRF-NRF	
3-023	OAZ1--ZBTB7A	3	0	chr19:2269744	chr19:4055247	n.d.	NRF-*ARF*	46,XY,t(1;10)
	NFATC1--CTDP1	1	1	chr18:79467582	chr18:79753652	n.d.	NRF-*ARF*	
3-024	PIM3--SCO2	17	3	chr22:49961811	chr22:50524424	Sanger	NRF-*ARF*	45,X -Y
	YWHAZ--KLF10	4	1	chr8:100951929	chr8:102652397	not detected	No	
	DHX9—GLUL	2	1	chr1:182839456	chr1:182388750	n.d.	No	
	FUS--SETD1A	2	0	chr16:31180227	chr16:30958717	n.d.	No	
3-025	TPM4--KLF2	10	1	chr19:16076697	chr19:16326856	n.d.	NRF-*ARF*	46,XY,t(9;11;17)(q22;q13;p13), inv(16)(p13q22)
	OAZ1--KLF2	2	1	chr19:2269744	chr19:16326856	n.d.	No	
3-029	C15orf57--CBX3	3	1	chr15:40561981	chr7:26201745	NGS	No	46,XX,inv(3)(q21q26)
	C15orf57--CBX3	3	1	chr15:40562772	chr7:26201769	NGS	NRF-*ARF*	
3-030	CCDC88C--ITPK1	2	0	chr14:91408659	chr14:93016801	n.d.	NRF-NRF	48 XY,+8,+15
3-032	NCOR2—UBC	2	0	chr12:124400501	chr12:124913774	n.d.	NRF-*ARF*	47,XX,+8,add(5)(p15)
	NCOR2—UBC	2	0	chr12:124362126	chr12:124913774	n.d.	NRF-*ARF*	
	ZEB2--CXCR4	2	0	chr2:144519939	chr2:136115912	gel electrophoresis	No	
3-033	FAM49B--KIAA0196	9	0	chr8:129903312	chr8:125084022	n.d.	No	45,X,-X
3-034	MAPKAPK5--ACAD10	24	3	chr12:111871180	chr12:111744643	n.d.	NRF-*ARF*	46,XY
3-035	TPM4--KLF2	5	0	chr19:16076697	chr19:16326856	n.d.	NRF-*ARF*	46,XY
3-038	C15orf57--CBX3	3	1	chr15:40561981	chr7:26201745	n.d.	No	48,XX,-7,+10,+19,+21
	NCOR2—UBC	2	1	chr12:124437930	chr12:124913774	n.d.	NRF-*ARF*	
	NCOR2—UBC	2	1	chr12:124466173	chr12:124913774	n.d.	NRF-NRF	
3-039	TPM4--KLF2	9	0	chr19:16076697	chr19:16326856	Sanger	NRF-*ARF*	46,XY,add(7)(q?),inv(16)(p13q22)
3-040	TPM4--KLF2	2	1	chr19:16076697	chr19:16326856	n.d.	NRF-*ARF*	46,XY
	NCOR2—UBC	2	0	chr12:124400501	chr12:124913774	n.d.	NRF-*ARF*	

[1] JR; junction reads; single reads containing sequences of two different genes.

[2] SF; spanning fragments; read pairs containing sequences of two different genes.

[3] Left GRCh38; position of the 3' end of the upstream partner gene in the fusion transcript after alignment to the GRCh38 genome.

[4] Right GRCh38; position of the 5' end of the downstream partner gene in the fusion transcript after alignment to the GRCh38 genome.

[5] Fusion transcripts were validated by RT-PCR and fragment size analysis by gel electrophoresis and in some instances by Sanger or NGS. Primers used for validation are shown in Table SV. n.d.; not determined.

[6] Indicated whether the downstream partner gene in the fusion transcript is translated in the normal reading frame (NRF) or alternative reading frame (ARF).

[7] No fusion protein due to absence of a coding sequence or presence of a stop codon in the upstream partner of the transcript.

Table SVI. Primers used for validation of fusion transcripts.

Primer	Sequence (5'- 3')	ENST transcript	Position		Size (bp)
			start (cds)	stop (cds)	
RUNX1-1F<br>RUNX1T1-1R	CTTCACAAACCCACCGCAAG<br>GGTGGCATTGTTGGAGGAGT	ENST00000344691<br>ENST00000520724	456<br>206	475<br>187	162 & 326
KMT2A-1F<br>MLLT3-1R	CAGCACTCTCTCCAATGGCA<br>TCTGGGATGGTGTGAAGCTG	ENST00000534358<br>ENST00000380338	4140<br>1189	4159<br>1170	143 & 257
KMT2A-1F<br>MLLT3-2R	CAGCACTCTCTCCAATGGCA<br>TGTTGTGATCCCTGGAAGGTT	ENST00000534358<br>ENST00000380338	4140<br>679	4159<br>659	338 & 452
KMT2A-1F<br>MLLT1-1R	CAGCACTCTCTCCAATGGCA<br>GCTTCTTGCGCAGTTGGG	ENST00000534358<br>ENST00000252674	4140<br>67	4159<br>50	134
KMT2A-2F<br>MLLT4-1R	AAGCCCGTCGAGGAAAAGAG<br>GTTGCAAAGTTTCCAGCAGCT	ENST00000534358<br>XM_005266996	3793<br>165	3812<br>150	359
KMT2A-2F<br>MLLT6-1R	AAGCCCGTCGAGGAAAAGAG<br>TGTGGGAGATGGGGTCCTTAT	ENST00000534358<br>ENST00000621332	3793<br>1687	3812<br>1667	279
CBFB-1F<br>MYH11-1R	TGAAGAGGCTCGGAGAAGGA<br>CTCTTCCAGCTGCGTCTTCA	ENST00000290858<br>ENST00000396324	429<br>4671	449<br>4652	139
CBFB-1F<br>MYH11-2R	TGAAGAGGCTCGGAGAAGGA<br>CAGCTTAATGGCCTTCCCCT	ENST00000290858<br>ENST00000396324	429<br>3939	449<br>3920	127
CBFB-1F<br>MYH11-3R	TGAAGAGGCTCGGAGAAGGA<br>CCATCGCTGCACTTGGACT	ENST00000290858<br>ENST00000396324	429<br>3833	449<br>3815	228 & 259
ERG-1F<br>FUS-1R	CTGAAGACCAGCGTCCTCAG<br>ACATTCTCACCCAGGCCTTG	ENST00000398919<br>ENST00000254108	869<br>887	888<br>868	163
FUS-1F<br>ERG-1R	AGCAGTGGTGGCTATGAACC<br>TCTTGAACTCCCCGTTGGTG	ENST00000254108<br>ENST00000398919	703<br>1042	722<br>1023	199
PML-1F<br>RARA-1R	GGTGCAGAGGATGAAGTGCT<br>GCTGACCCCATAGTGGTAGC	ENST00000268058<br>ENST00000254066	996<br>309	1016<br>290	319 & 390
PML-2F<br>RARA-2R	GGTGCAGAGGATGAAGTGCT<br>CTGGGCAGGGAGAGAAGTTG	ENST00000268058<br>ENST00000254066	1056<br>178+2991	1075<br>178+2972	676
RARA-1F<br>PML-1R	CCCTACGCCTTCTTCTTCCC<br>CGTCAATAGGGTCCCTGGGA	ENST00000254066<br>ENST00000268058	67<br>1246	86<br>1227	175
RARA-1F<br>PML-2R	CCCTACGCCTTCTTCTTCCC<br>GGACTCACTGCTGCTGTCAT	ENST00000254066<br>ENST00000268058	67<br>1746	86<br>1727	201
DEK-1F<br>NUP214-1R	AGTTGAAGAAACCCCCTACAGA<br>CCAGATGCTGATCCCACTCC	ENST00000397239<br>ENST00000359428	944<br>2521	965<br>2502	189
NUP214-1F<br>DEK-1R	TTGAGGGCTTTGCTGGTGTT<br>TGGGAACGAGTCATCTTCTCTG	ENST00000359428<br>ENST00000397239	2397<br>1027 *31	2416<br>1027 *10	231
NSD1-1F<br>NUP98-1R	GCTTAGATTCAGCTGGGCCA<br>CGGATTCCGGAAGAGAGGAG	ENST00000439151<br>ENST00000355260	3677<br>1515	3696<br>1496	227
NUP98-1F<br>NSD1-1R	TGGCACAAATACCAGTGGGA<br>GCACCTGCTCCTGTACCTTC	ENST00000355260<br>ENST00000439151	1179<br>3916	1198<br>3897	209 & 350
TPM4-1F<br>KLF2-1R	GTGAAACGCAAGATCCAGGC<br>ATGTGCCGTTTCATGTGCAG	ENST00000300933<br>ENST00000248071	28<br>1064	47<br>1045	277
C15ORF57-1F<br>CBX3-1R	CTGAAGGTGAAGGCCAGAGG<br>TGTAGTTTTGTTGGAGGCCA	NM_001080791<br>ENST00000337620	170<br>21	189<br>2	221, 269,<br>1024
NCOR2-1F<br>UBC-1R	CACCGCCACTTGCCTGAG<br>TCCAGCAAAGATCAGCCTCTG	ENST00000458234<br>ENST00000536769	-240<br>141	-223<br>121	220 & 267
NCOR2-2F<br>UBC-1R	GGCTGAGTGCGTCCTCTATT<br>TCCAGCAAAGATCAGCCTCTG	ENST00000458234<br>ENST00000536769	1389<br>141	1408<br>121	238 & 569
ZEB2-1F<br>CXCR4-1R	CGCGGCTTCTTCATGCTTTT<br>TTCACGGAAACAGGGTTCCT	ENST00000627532<br>ENST00000241393	-133<br>94	-114<br>74	142
ETV6-1F<br>LYN-1R	GGGAAGCCCATCAACCTCTC<br>TATCCCATGGCTTCTGTGGC	ENST00000396373<br>ENST00000519728	901<br>709	920<br>690	181
PIM3-1F<br>SCO2-1R	ACCGCGACATTAAGGACGAA<br>AAAGCCAGGACCTCAGATGC	ENST00000360612<br>ENST00000252785	503<br>112	522<br>93	239

[1] PCR was performed for 33 cycles using PWO SuperYield DNA polymerase or Phusion Flash High-Fidelity PCR Master Mix.

Table SVII. Detection of small variants in 13 genes that are recurrently mutated in AML.

AML	Gene	Chrom	GRCh38[1]	Exon[2]	HGVSc[3]	HGVSp[4]	HS[5]	Cos[6]	SNP[7]	Class[8]	Jaiswal[9]	Existing Variation[10]	DP[11]	RD[12]	AD[13]	VAF[14]	SIFT[15]	PolyPhen[16]	Validation[17]
1-001	NRAS	chr1	114716123	2/7	ENST00000369535.4:c.38G>A	ENSP00000358548.4:p.Gly13Asp	yes	yes	no	A	yes	NRASbase_D0125:g.40791G>A,CM071907,COSM574,COSM575,COSM573	122	96	21	0.18	deleterious (0.05)	benign(0.004)	NGS
1-002	DNMT3A	chr2	25234373	23/23	ENST00000321117.7:c.2645G>A	ENSP00000324375.5:p.Arg882His	yes	yes	yes	A	yes	rs147001633,COSM442676,COSM3356083,COSM1583129,COSM99740,COSM52944	82	33	48	0.59	deleterious (0.03)	Probably damaging(0.993)	Sanger
	FLT3	chr13	28018505	20/24	ENST00000241453.9:c.2503G>C	ENSP00000241453.7:p.Asp835His	yes	yes	no	A	yes	KinMutBase_FLT3_DNA:g.53109G>T,KinMutBase_FLT3_DNA:g.53109G>C,KinMutBase_FLT3_DNA:g.53109G>A,KinMutBase_FLT3_DNA:g.53109delG,COSM783,COSM789,COSM785	919	742	146	0.16	deleterious (0)	Probably damaging(1)	Sanger
	FLT3	chr13	28052579	5/24	ENST00000241453.9:c.580G>A	ENSP00000241453.7:p.Val194Met	no	yes	yes	B	no	rs146030737,COSM28039	675	336	323	0.49	tolerated(0.05)	benign(0.15)	Sanger
	IDH1	chr2	208243577	6/10	ENST00000345146.4:c.548A>G	ENSP00000260985.2:p.Tyr183Cys	no	no	yes	D	no	rs34599179,CM073129	629	322	304	0.49	deleterious (0)	Probably damaging(0.994)	Sanger
	TET2	chr4	105236781	3/11	ENST00000380013.6:c.2839C>T	ENSP00000369351.4:p.Gln947Ter	no	yes	no	B	yes	COSM1318625,COSM1318626	111	88	13	0.13	NA	NA	NGS
	TET2	chr4	105276128	11/11	ENST00000380013.6:c.5618T>C	ENSP00000369351.4:p.Ile1873Thr	no	yes	yes	B	yes	rs116519313,COSM41741	165	34	129	0.79	deleterious (0)	Probably damaging(1)	NGS
	NPM1	chr5	171410539	12/12	ENST00000517671.3:c.860_863dup	ENSP00000428755.1:p.Trp288CysfsTer12	yes	yes	no	A	yes	COSM1319222,COSM255167,COSM46518,COSM158604	849	554	292	0.34	NA	NA	Sanger
1-003	DNMT3A	chr2	25234374	23/23	ENST00000321117.7:c.2644C>T	ENSP00000324375.5:p.Arg882Cys	yes	yes	yes	A	yes	rs377577594,COSM1166704,COSM1583136,COSM53042,COSM87001	241	131	107	0.45	deleterious (0)	Probably damaging(0.997)	Sanger
	TET2	chr4	105261793	8/11	ENST00000380013.6:c.3989C>A	ENSP00000369351.4:p.Ser1330Tyr	no	no	no	C	yes	NA	115	0	114	0.99	deleterious (0)	Probably damaging(0.987)	Sanger
	NPM1	chr5	171410540	12/12	ENST00000517671.3:c.863_864insCCTG	ENSP00000428755.1:p.Trp288CysfsTer12	yes	yes	no	A	yes	COSM20866,COSM20863,COSM1658759,COSM28937	1373	877	490	0.36	NA	NA	Sanger
1-004	KIT	chr4	54733155	17/21	ENST00000288135.5:c.2447A>T	ENSP00000288135.5:p.Asp816Val	yes	yes	yes	A	yes	rs121913507,KinMutBase_KIT_DNA:g.76140A>T,CM952169,COSM1314,COSM24675,COSM12711	460	289	170	0.37	deleterious (0)	Probably damaging(0.977)	Sanger
1-005	IDH2	chr15	90084321	11/11	ENST00000330062.5:c.1304C>T	ENSP00000331897.3:p.Thr435Met	no	no	yes	D	no	rs118053940	533	287	242	0.46	tolerated(0.14)	benign(0.153)	Sanger
1-006	DNMT3A	chr2	25234373	23/23	ENST00000321117.7:c.2645G>A	ENSP00000324375.5:p.Arg882His	yes	yes	yes	A	yes	rs147001633,COSM442676,COSM3356083,COSM1583129,COSM99740,COSM52944	198	115	78	0.40	deleterious (0.03)	Probably damaging(0.993)	Sanger
1-008	KIT	chr4	54695617	2/21	ENST00000288135.5:c.173G>T	ENSP00000288135.5:p.Cys58Phe	no	no	no	C	no	NA	307	261	46	0.15	deleterious (0)	Probably damaging(1)	NGS
	KIT	chr4	54733155	17/21	ENST00000288135.5:c.2447A>T	ENSP00000288135.5:p.Asp816Val	yes	yes	yes	A	yes	rs121913507,KinMutBase_KIT_DNA:g.76140A>T,CM952169,COSM1314,COSM24675,COSM12711	384	194	189	0.49	deleterious (0)	Probably damaging(0.977)	Sanger
	RUNX1	chr21	34792416	8/8	ENST00000300305.5:c.1161del	ENSP00000300305.3:p.Ser388ArgfsTer206	no	no	yes	D	yes	rs1055306	369	203	163	0.45	NA	NA	Sanger
	RUNX1	chr21	34887027	3/8	ENST00000300305.5:c.167T>C	ENSP00000300305.3:p.Leu56Ser	no	yes	yes	B	no	rs111527738,COSM24756	334	167	160	0.49	tolerated(0.66)	Possibly damaging(0.812)	Sanger
	ASXL1	chr20	32434638	13/13	ENST00000375687.6:c.1934dup	ENSP00000364839.4:p.Gly646TrpfsTer12	yes	yes	no	A	yes	COSM1411076	70	52	20	0.28	NA	NA	Sanger
2-001	DNMT3A	chr2	25234374	23/23	ENST00000321117.7:c.2644C>T	ENSP00000324375.5:p.Arg882Cys	yes	yes	yes	A	yes	rs377577594,COSM1166704,COSM1583136,COSM53042,COSM87001	72	39	33	0.46	deleterious (0)	Probably damaging(0.997)	Sanger
	NPM1	chr5	171410539	12/12	ENST00000517671.3:c.860_863dup	ENSP00000428755.1:p.Trp288CysfsTer12	yes	yes	no	A	yes	COSM1319222,COSM255167,COSM46518,COSM158604	512	347	164	0.32	NA	NA	Sanger
2-002	DNMT3A	chr2	25244579	14/23	ENST00000321117.7:c.1628G>T	ENSP00000324375.5:p.Gly543Val	no	yes	no	B	no	COSM249135,COSM1583073,COSM256033	73	39	34	0.47	deleterious (0)	Probably damaging(0.999)	Sanger
	RUNX1	chr21	34834566	6/8	ENST00000300305.5:c.649G>A	ENSP00000300305.3:p.Gly217Arg	no	no	no	C	no	NA	521	224	197	0.47	deleterious (0.01)	Probably damaging(0.948)	Sanger
	TET2	chr4	105235712	3/11	ENST00000380013.6:c.1771del	ENSP00000369351.4:p.Gln591SerfsTer10	no	no	no	C	yes	NA	132	66	56	0.46	NA	NA	Sanger
	TET2	chr4	105272773	10/11	ENST00000380013.6:c.4392C>A	ENSP00000369351.4:p.Cys1464Ter	no	yes	no	B	yes	COSM211624	122	58	61	0.51	NA	NA	Sanger
2-003	FLT3	chr13	28018505	20/24	ENST00000241453.9:c.2503G>C	ENSP00000241453.7:p.Asp835His	yes	yes	no	A	yes	KinMutBase_FLT3_DNA:g.53109G>T,KinMutBase_FLT3_DNA:g.53109G>C,KinMutBase_FLT3_DNA:g.53109G>A,KinMutBase_FLT3_DNA:g.53109delG,COSM783,COSM789,COSM785	622	292	312	0.52	deleterious (0)	Probably damaging(1)	Sanger
	FLT3	chr13	28049450	8/24	ENST00000241453.9:c.970G>A	ENSP00000241453.7:p.Asp324Asn	no	yes	yes	B	no	rs35602083,CM066823,COSM28040	524	259	263	0.50	tolerated(0.33)	benign(0.035)	Sanger
2-004	KIT	chr4	54733154	17/21	ENST00000288135.5:c.2446G>T	ENSP00000288135.5:p.Asp816Tyr	yes	yes	yes	A	yes	rs121913506,KinMutBase_KIT_DNA:g.76139G>C,KinMutBase_KIT_DNA:g.76139G>T,COSM1313,COSM1311,COSM1310	419	251	166	0.40	deleterious (0)	Probably damaging(0.963)	Sanger
	RUNX1	chr21	34887027	3/8	ENST00000300305.5:c.167T>C	ENSP00000300305.3:p.Leu56Ser	no	yes	yes	B	no	rs111527738,COSM24756	552	286	260	0.48	tolerated(0.66)	Possibly damaging(0.812)	Sanger
	ASXL1	chr20	32436457	13/13	ENST00000375687.6:c.3745A>G	ENSP00000364839.4:p.Met1249Val	yes	no	yes	D	no	rs146141075	162	89	72	0.45	tolerated(0.48)	benign(0)	Sanger

	WT1	chr11	32396363	7/10	ENST00000332351.5:c.1133_1142dup	ENSP00000331327.3:p.Ala382CysfsTer6	no	yes	no	B	yes	COSM27304,COSM41869,COSM29406,COSM28968,COSM28954,COSM27303,COSM28947,COSM41854,COSM28955	39	33	5	0.13	NA	NA	NGS
2-005	IDH2	chr15	90087472	6/11	ENST00000330062.5:c.782G>A	ENSP00000331897.3:p.Arg261His	no	no	yes	C	no	rs118101777	1141	571	565	0.50	deleterious (0)	Probably damaging(0.98)	Sanger
2-006	FLT3	chr13	28034118	14/24	ENST00000241453.9: c.1780_1800dup	ENSP00000241453.7:p.Phe594_Asp600dup	yes	yes	no	A	no	COSM1317919,COSM27907,COSM19953,COSM20768,COSM840,COSM19858	888	672	138	0.16	NA	NA	Sanger
	TET2	chr4	105237193	3/11	ENST00000380013.6:c.3251A>C	ENSP00000369351.4:p.Gln1084Pro	no	no	yes	D	no	rs75056899	62	42	19	0.31	tolerated(0.06)	benign(0.01)	Sanger
2-007	KIT	chr4	54726036	9/21	ENST00000288135.5:c.1526A>T	ENSP00000288135.5:p.Lys509Ile	no	yes	no	B	no	CM061829,COSM96885,COSM1430134	452	234	209	0.47	deleterious (0.03)	Probably damaging(0.994)	Sanger
	TET2	chr4	105259708	7/11	ENST00000380013.6:c.3893G>A	ENSP00000369351.4:p.Cys1298Tyr	no	yes	no	B	yes	COSM43474,COSM87138	36	19	17	0.47	deleterious (0)	Probably damaging(0.995)	Sanger
	CEBPA	chr19	33301447	1/1	ENST00000498907.2:c.968G>T	ENSP00000427514.1:p.Arg323Leu	no	yes	no	B	no	COSM3707035	603	303	297	0.50	deleterious (0)	Possibly damaging(0.794)	Sanger
2-008	FLT3	chr13	28018503	20/24	ENST00000241453.9:c.2505T>G	ENSP00000241453.7:p.Asp835Glu	yes	yes	yes	A	yes	rs121913487,KinMutBase_FLT3_DNA:g.53111T>A,KinMutBase_FLT3_DNA:g.53111T>G,COSM788,COSM787	1058	467	573	0.55	deleterious (0)	Probably damaging(0.997)	Sanger
	IDH1	chr2	208243577	6/10	ENST00000345146.4:c.548A>G	ENSP00000260985.2:p.Tyr183Cys	no	no	yes	D	no	rs34599179,CM073129	420	204	212	0.51	deleterious (0)	Probably damaging(0.994)	Sanger
	RUNX1	chr21	34799384	7/8	ENST00000300305.5:c.883dup	ENSP00000300305.3:p.Ser295PhefsTer305	no	no	no	C	yes	NA	764	478	274	0.36	NA	NA	Sanger
2-009	FLT3	chr13	28052579	5/24	ENST00000241453.9:c.580G>A	ENSP00000241453.7:p.Val194Met	no	yes	yes	B	no	rs146030737,COSM28039	145	16	118	0.88	tolerated(0.05)	benign(0.15)	Sanger
	TET2	chr4	105237193	3/11	ENST00000380013.6:c.3251A>C	ENSP00000369351.4:p.Gln1084Pro	no	no	yes	D	no	rs75056899	26	16	10	0.38	tolerated(0.06)	benign(0.01)	Sanger
	CEBPA	chr19	33301478	1/1	ENST00000498907.2:c.934_936dup	ENSP00000427514.1:p.Gln312dup	no	yes	no	B	no	COSM18915,COSM18466,COSM249784,COSM97055	2367	1532	819	0.35	NA	NA	Sanger
	CEBPA	chr19	33302075	1/1	ENST00000498907.2:c.332_339del	ENSP00000427514.1:p.Ala111GlyfsTer56	no	yes	no	B	yes	COSM20917,COSM29253	728	244	381	0.61	NA	NA	Sanger
2-010	TET2	chr4	105275662	11/11	ENST00000380013.6:c.5152G>T	ENSP00000369351.4:p.Val1718Leu	no	yes	yes	B	no	rs142312318,COSM41742	103	67	35	0.34	tolerated(0.34)	benign(0.006)	Sanger
	NRAS	chr1	114716127	2/7	ENST00000369535.4:c.34G>T	ENSP00000358548.4:p.Gly12Cys	yes	yes	no	A	yes	COSM562,COSM561,COSM563	138	78	57	0.42	deleterious (0.04)	Probably damaging(0.955)	Sanger
2-011	FLT3	chr13	28018502	20/24	ENST00000241453.9:c.2503_2505del	ENSP00000241453.7:p.Asp835del	yes	yes	yes	A	yes	rs121913486,COSM796,COSM854	1228	890	210	0.19	NA	NA	Sanger
	FLT3	chr13	28018505	20/24	ENST00000241453.9:c.2503G>T	ENSP00000241453.7:p.Asp835Tyr	yes	yes	no	A	yes	KinMutBase_FLT3_DNA:g.53109G>T,KinMutBase_FLT3_DNA:g.53109G>C,KinMutBase_FLT3_DNA:g.53109G>A,KinMutBase_FLT3_DNA:g.53109delG,COSM783,COSM789,COSM785	1197	554	300	0.35	deleterious (0)	Probably damaging(1)	Sanger
	IDH1	chr2	208248388	4/10	ENST00000345146.4:c.395G>A	ENSP00000260985.2:p.Arg132His	yes	yes	yes	A	yes	COSM28746,COSM28750,rs121913500	292	162	129	0.44	deleterious (0)	Possibly damaging(0.813)	Sanger
	NPM1	chr5	171410539	12/12	ENST00000517671.3:c.860_863dup	ENSP00000428755.1:p.Trp288CysfsTer12	yes	yes	no	A	yes	COSM1319222,COSM255167,COSM46518,COSM158604	1821	1167	645	0.36	NA	NA	Sanger
2-012	NPM1	chr5	171410541	12/12	ENST00000517671.3:c.863_864insCTTG	ENSP00000428755.1:p.Trp288CysfsTer12	yes	yes	no	A	yes	COSM158600,COSM27392,COSM1319221,COSM20861	421	274	146	0.35	NA	NA	Sanger
2-013	RUNX1	chr21	34880640	4/8	ENST00000300305.5:c.423_424dup	ENSP00000300305.3:p.Ala142GlyfsTer4	no	yes	no	B	yes	COSM1717863	323	234	86	0.27	NA	NA	Sanger
2-014	KIT	chr4	54658066	1/21	ENST00000288135.5:c.52C>T	ENSP00000288135.5:p.Leu18Phe	no	no	yes	D	no	rs370787811	97	51	46	0.47	tolerated(0.29)	benign(0.008)	Sanger
	RUNX1	chr21	34887027	3/8	ENST00000300305.5:c.167T>C	ENSP00000300305.3:p.Leu56Ser	no	yes	yes	B	no	rs111527738,COSM24756	257	125	130	0.51	tolerated(0.66)	Possibly damaging(0.812)	Sanger
	CEBPA	chr19	33301825	1/1	ENST00000498907.2:c.584_589dup	ENSP00000427514.1:p.His195_Pro196dup	no	no	no	C	no	NA	94	81	10	0.11	NA	NA	Sanger
2-015	DNMT3A	chr2	25244603	14/23	ENST00000321117.7:c.1583_1603dup	ENSP00000324375.5:p.Tyr528_Gln534dup	no	no	no	C	no	NA	229	202	26	0.11	NA	NA	Sanger
	TET2	chr4	105272692	10/11	ENST00000380013.6:c.4317dup	ENSP00000369351.4:p.Arg1440ThrfsTer38	no	no	no	C	yes	NA	99	85	12	0.12	NA	NA	NGS
	NPM1	chr5	171410539	12/12	ENST00000517671.3:c.860_863dup	ENSP00000428755.1:p.Trp288CysfsTer12	yes	yes	no	A	yes	COSM1319222,COSM255167,COSM46518,COSM158604	2382	1533	840	0.35	NA	NA	Sanger
2-016	DNMT3A	chr2	25244580	14/23	ENST00000321117.7:c.1627G>T	ENSP00000324375.5:p.Gly543Cys	no	yes	no	B	no	COSM87002	105	48	57	0.54	deleterious (0)	Probably damaging(1)	Sanger
	TET2	chr4	105234042	3/11	ENST00000380013.6:c.100C>T	ENSP00000369351.4:p.Leu34Phe	no	no	yes	D	no	rs111948941	73	28	40	0.59	deleterious (0.01)	benign(0.384)	Sanger
	NPM1	chr5	171410539	12/12	ENST00000517671.3:c.860_863dup	ENSP00000428755.1:p.Trp288CysfsTer12	yes	yes	no	A	yes	COSM1319222,COSM255167,COSM46518,COSM158604	1477	911	560	0.38	NA	NA	Sanger
	WT1	chr11	32391978	9/10	ENST00000332351.5:c.1426A>G	ENSP00000331327.3:p.Lys476Glu	no	no	no	C	no	NA	75	47	9	0.16	deleterious (0.02)	Probably damaging(0.996)	Sanger

2-018	FLT3	chr13	28034114	14/24	ENST00000241453.9:c.1781_1804dup	ENSP00000241453.7:p.Leu601_Lys602insIleArgGluTyrGluTyrAspLeu	yes	yes	no	A	no	COSM1317921,COSM19855	736	617	112	0.15	NA	NA	Sanger
	NPM1	chr5	171410539	12/12	ENST00000517671.3:c.860_863dup	ENSP00000428755.1:p.Trp288CysfsTer12	yes	yes	no	A	yes	COSM1319222,COSM255167,COSM46518,COSM158604	1625	861	751	0.46	NA	NA	Sanger
2-019	TP53	chr17	7674885	6/11	ENST00000269305.6:c.646G>A	ENSP00000269305.4:p.Val216Met	no	yes	no	B	yes	TP53_g.12715G>A,TP53_g.12715G>T,TP53_g.12715G>C,TP53_g.12715del,CX952222,COSM1386670,COSM120097,COSM1644280,COSM3388195,COSM1386671,COSM120096,COSM120095,COSM1386669,COSM1386668,COSM120098,COSM10667,COSM11210	229	5	219	0.98	deleterious (0)	Probably damaging(1)	Sanger
2-020	ASXL1	chr20	32434789	13/13	ENST00000375687.6:c.2077C>T	ENSP00000364839.4:p.Arg693Ter	yes	yes	yes	A	yes	rs373221034,COSM51388	141	72	67	0.48	NA	NA	Sanger
2-021	RUNX1	chr21	34886877	3/8	ENST00000300305.5:c.317G>C	ENSP00000300305.3:p.Trp106Ser	no	no	no	C	no	NA	732	7	699	0.99	deleterious (0)	Probably damaging(1)	Sanger
	ASXL1	chr20	32434599	13/13	ENST00000375687.6:c.1900_1922del	ENSP00000364839.4:p.Glu635ArgfsTer15	yes	yes	no	A	yes	COSM51200	99	77	21	0.21	NA	NA	Sanger
2-022	FLT3	chr13	28036022	11/24	ENST00000241453.9:c.1331A>T	ENSP00000241453.7:p.Glu444Val	no	no	no	C	no	NA	1852	1139	691	0.38	tolerated(0.11)	benign(0.024)	Sanger
2-023	DNMT3A	chr2	25240672	18/23	ENST00000321117.7:c.2141C>G	ENSP00000324375.5:p.Ser714Cys	no	yes	yes	B	yes	rs367909007,COSM442677,COSM87011	246	128	117	0.48	deleterious (0.01)	Probably damaging(0.999)	Sanger
	RUNX1	chr21	34792223	8/8	ENST00000300305.5:c.1342_1354dup	ENSP00000300305.3:p.Val452GlufsTer152	no	no	no	C	yes	NA	710	503	195	0.28	NA	NA	Sanger
	NPM1	chr5	171410539	12/12	ENST00000517671.3:c.860_863dup	ENSP00000428755.1:p.Trp288CysfsTer12	yes	yes	no	A	yes	COSM1319222,COSM255167,COSM46518,COSM158604	1792	1171	613	0.34	NA	NA	Sanger
2-024	DNMT3A	chr2	25240666	18/23	ENST00000321117.7:c.2147T>A	ENSP00000324375.5:p.Val716Asp	no	yes	no	B	yes	COSM1583097	50	26	24	0.48	deleterious (0)	Probably damaging(0.999)	Sanger
	RUNX1	chr21	34886871	3/8	ENST00000300305.5:c.323G>C	ENSP00000300305.3:p.Cys108Ser	no	no	no	C	no	NA	258	142	106	0.43	tolerated(0.27)	Probably damaging(0.998)	NGS[18]
		chr21	34886872	3/8	ENST00000300305.5:c.322T>A	ENSP00000300305.3:p.Cys108Ser	no	no	no	C	no	NA	254	140	104	0.43	tolerated(0.27)	Probably damaging(0.998)	NGS[18]
		chr21	34886874	3/8	ENST00000300305.5:c.320G>A	ENSP00000300305.3:p.Arg107His	no	yes	no	B	no	COSM36056	257	141	106	0.43	deleterious (0)	Probably damaging(0.999)	NGS[18]
	TET2	chr4	105275613	11/11	ENST00000380013.6:c.5103G>A	ENSP00000369351.4:p.Met1701Ile	no	no	yes	D	no	rs62623390	167	100	66	0.40	tolerated(0.15)	benign(0.009)	Sanger
2-025	NRAS	chr1	114713908	3/7	ENST00000369535.4:c.182A>G	ENSP00000358548.4:p.Gln61Arg	yes	yes	no	A	yes	COSM583,COSM584,COSM582	186	136	48	0.26	tolerated(0.06)	benign(0.006)	NGS
	CEBPA	chr19	33302154	1/1	ENST00000498907.2:c.260del	ENSP00000427514.1:p.Gln87ArgfsTer73	no	no	no	C	yes	NA	1597	645	922	0.59	NA	NA	Sanger
2-027	IDH1	chr2	208248389	4/10	ENST00000345146.4:c.394C>T	ENSP00000260985.2:p.Arg132Cys	yes	yes	yes	A	yes	COSM28749,COSM28747,COSM28748,rs121913499	178	95	80	0.46	deleterious (0.03)	Possibly damaging(0.907)	Sanger
	ASXL1	chr20	32434638	13/13	ENST00000375687.6:c.1934dup	ENSP00000364839.4:p.Gly646TrpfsTer12	yes	yes	no	A	yes	COSM1411076	75	57	16	0.22	NA	NA	Sanger
	NRAS	chr1	114713909	3/7	ENST00000369535.4:c.181C>A	ENSP00000358548.4:p.Gln61Lys	yes	yes	yes	A	yes	rs121913254,COSM580,COSM581	109	61	46	0.43	deleterious (0.01)	benign(0.045)	Sanger
2-028	DNMT3A	chr2	25248139	7/23	ENST00000321117.7:c.752dup	ENSP00000324375.5:p.Asp252Ter	no	no	no	C	yes	NA	53	6	43	0.88	NA	NA	Sanger
	TET2	chr4	105234350	3/11	ENST00000380013.6:c.409del	ENSP00000369351.4:p.Ser137ValfsTer8	no	no	no	C	yes	NA	140	66	75	0.53	NA	NA	Sanger
	TET2	chr4	105236721	3/11	ENST00000380013.6:c.2784del	ENSP00000369351.4:p.Pro929LeufsTer24	no	no	no	C	yes	NA	98	61	36	0.37	NA	NA	Sanger
	NPM1	chr5	171410539	12/12	ENST00000517671.3:c.860_863dup	ENSP00000428755.1:p.Trp288CysfsTer12	yes	yes	no	A	yes	COSM1319222,COSM255167,COSM46518,COSM158604	612	408	202	0.33	NA	NA	Sanger
2-029	DNMT3A	chr2	25234367	23/23	ENST00000321117.7:c.2651C>T	ENSP00000324375.5:p.Ala884Val	no	yes	no	B	yes	COSM231547	81	46	34	0.43	deleterious (0.01)	Probably damaging(0.998)	Sanger
	IDH1	chr2	208245342	5/10	ENST00000345146.4:c.497C>T	ENSP00000260985.2:p.Thr166Ile	no	no	no	C	no	NA	1520	732	775	0.51	deleterious (0.05)	benign(0.039)	Sanger
	NPM1	chr5	171410539	12/12	ENST00000517671.3:c.860_863dup	ENSP00000428755.1:p.Trp288CysfsTer12	yes	yes	no	A	yes	COSM1319222,COSM255167,COSM46518,COSM158604	713	450	260	0.37	NA	NA	Sanger
2-030	DNMT3A	chr2	25243931	16/23	ENST00000321117.7:c.1903C>T	ENSP00000324375.5:p.Arg635Trp	no	yes	yes	B	yes	rs144689354,COSM1407108,COSM87012	246	147	97	0.40	deleterious (0)	Possibly damaging(0.854)	Sanger
	NPM1	chr5	171410539	12/12	ENST00000517671.3:c.860_863dup	ENSP00000428755.1:p.Trp288CysfsTer12	yes	yes	no	A	yes	COSM1319222,COSM255167,COSM46518,COSM158604	1775	1154	619	0.35	NA	NA	Sanger
	IDH2	chr15	90088702	4/11	ENST00000330062.5:c.419G>A	ENSP00000331897.3:p.Arg140Gln	yes	yes	yes	A	yes	rs121913502,COSM41875,COSM41590	617	332	284	0.46	deleterious (0)	Probably damaging(1)	Sanger
	WT1	chr11	32396358	7/10	ENST00000332351.5:c.1143_1147dup	ENSP00000331327.3:p.Ser383TrpfsTer68	no	no	no	C	yes	NA	88	66	20	0.23	NA	NA	NGS
2-032	FLT3	chr13	28034125	14/24	ENST00000241453.9:c.1793_1794insGCCCTTCAGAGAATATGA	ENSP00000241453.7:p.Glu598_Tyr599insProPheArgGluTyrGlu	yes	yes	no	A	no	COSM19961,COSM1317913,COSM1317912	677	414	248	0.37	NA	NA	Sanger
	WT1	chr11	32396365	7/10	ENST00000332351.5:c.1140dup	ENSP00000331327.3:p.Ser381ValfsTer4	no	yes	no	B	yes	COSM1166635,COSM41864,COSM30772,COSM52939,COSM41855,COSM27301	20	0	19	1.0	NA	NA	Sanger[18]

		chr11	32396368	7/10	ENST00000332351.5:c.1138C>G	ENSP00000331327.3:p.Arg380Gly	no	yes	no	B	no	COSM1585731,COSM28984,COSM926360,COSM30695,COSM28864,COSM35996,COSM49047,COSM49058,COSM27299,COSM30690	20	0	19	1.0	deleterious (0)	probably damaging(0.998)	Sanger[18]
2-033	FLT3	chr13	28034116	14/24	ENST00000241453.9:c.1779_1802dup	ENSP00000241453.7:p.Phe594_Leu601dup	yes	no	no	A	no	NA	974	708	251	0.26	NA	NA	Sanger
	TET2	chr4	105234647	3/11	ENST00000380013.6:c.706del	ENSP00000369351.4:p.Asp236IlefsTer14	no	no	no	C	yes	NA	48	31	13	0.30	NA	NA	Sanger
	TET2	chr4	105275653	11/11	ENST00000380013.6:c.5145_5151del	ENSP00000369351.4:p.His1716Ter	no	no	no	C	yes	NA	123	78	43	0.35	NA	NA	Sanger
	NPM1	chr5	171410539	12/12	ENST00000517671.3:c.860_863dup	ENSP00000428755.1:p.Trp288CysfsTer12	yes	yes	no	A	yes	COSM1319222,COSM255167,COSM46518,COSM158604	1433	922	507	0.35	NA	NA	Sanger
2-034	FLT3	chr13	28034345	13/24	ENST00000241453.9:c.1660T>A	ENSP00000241453.7:p.Phe554Ile	no	no	no	C	no	NA	608	325	280	0.46	tolerated(0.34)	benign(0.41)	Sanger
	FLT3	chr13	28034347	13/24	ENST00000241453.9:c.1658T>C	ENSP00000241453.7:p.Leu553Pro	no	no	no	C	no	NA	601	318	280	0.46	deleterious (0.04)	benign(0)	Sanger
	TET2	chr4	105275613	11/11	ENST00000380013.6:c.5103G>A	ENSP00000369351.4:p.Met1701Ile	no	no	yes	D	no	rs62623390	277	136	141	0.51	tolerated(0.15)	benign(0.009)	Sanger
	NPM1	chr5	171410539	12/12	ENST00000517671.3:c.860_863dup	ENSP00000428755.1:p.Trp288CysfsTer12	yes	yes	no	A	yes	COSM1319222,COSM255167,COSM46518,COSM158604	588	398	188	0.32	NA	NA	Sanger
2-035	FLT3	chr13	28034125	14/24	ENST00000241453.9:c.1776_1793dup	ENSP00000241453.7:p.Tyr597_Glu598insAspAspPheArgGluTyr	yes	yes	no	A	no	COSM19961,COSM1317913,COSM1317912	1123	658	434	0.39	NA	NA	Sanger
	ASXL1	chr20	32436018	13/13	ENST00000375687.6:c.3306G>T	ENSP00000364839.4:p.Glu1102Asp	yes	yes	yes	A	no	rs139115934,COSM36205,COSM1155723	334	185	147	0.44	deleterious (0.03)	probably damaging(0.911)	Sanger
	NPM1	chr5	171410539	12/12	ENST00000517671.3:c.860_863dup	ENSP00000428755.1:p.Trp288CysfsTer12	yes	yes	no	A	yes	COSM1319222,COSM255167,COSM46518,COSM158604	2112	1363	742	0.35	NA	NA	Sanger
2-036	DNMT3A	chr2	25234373	23/23	ENST00000321117.7:c.2645G>C	ENSP00000324375.5:p.Arg882Pro	yes	yes	yes	A	yes	rs147001633,COSM442676,COSM3356083,COSM1583129,COSM99740,COSM52944	157	66	89	0.57	deleterious (0)	probably damaging(0.997)	Sanger
	RUNX1	chr21	34887027	3/8	ENST00000300305.5:c.167T>C	ENSP00000300305.3:p.Leu56Ser	no	yes	yes	B	no	rs111527738,COSM24756	387	204	174	0.46	tolerated(0.66)	possibly damaging(0.812)	Sanger
	CEBPA	chr19	33302075	1/1	ENST00000498907.2:c.332_339del	ENSP00000427514.1:p.Ala111GlyfsTer56	no	yes	no	B	yes	COSM20917,COSM29253	176	95	61	0.39	NA	NA	Sanger
2-037	RUNX1	chr21	34859495	5/8	ENST00000300305.5:c.592G>A	ENSP00000300305.3:p.Asp198Asn	no	yes	no	B	no	CM013280,COSM24721	320	205	108	0.35	deleterious (0.02)	possibly damaging(0.633)	Sanger
	ASXL1	chr20	32434638	13/13	ENST00000375687.6:c.1934dup	ENSP00000364839.4:p.Gly646TrpfsTer12	yes	yes	no	A	yes	COSM1411076	57	52	6	0.10	NA	NA	Sanger
2-038	NRAS	chr1	114713908	3/7	ENST00000369535.4:c.182A>G	ENSP00000358548.4:p.Gln61Arg	yes	yes	no	A	yes	COSM583,COSM584,COSM582	130	110	15	0.12	tolerated(0.06)	benign(0.006)	NGS[18]
		chr1	114713909	3/7	ENST00000369535.4:c.181C>A	ENSP00000358548.4:p.Gln61Lys	yes	yes	yes	A	yes	rs121913254,COSM580,COSM581	128	108	15	0.12	deleterious (0.01)	benign(0.045)	NGS[18]
	NRAS	chr1	114716126	2/7	ENST00000369535.4:c.35G>C	ENSP00000358548.4:p.Gly12Ala	yes	yes	no	A	yes	COSM565,COSM566,COSM564	117	74	39	0.35	deleterious (0.02)	benign(0.267)	Sanger
2-039	RUNX1	chr21	34886865	3/8	ENST00000300305.5:c.329A>G	ENSP00000300305.3:p.Lys110Arg	no	yes	no	B	no	COSM26024	119	73	42	0.37	deleterious (0)	probably damaging(0.996)	Sanger
	CEBPA	chr19	33301475	1/1	ENST00000498907.2:c.937_939dup	ENSP00000427514.1:p.Lys313dup	no	yes	no	B	no	COSM18099,COSM18541,COSM18901,COSM29139,COSM18640,COSM18103	2262	1469	780	0.35	NA	NA	Sanger
	CEBPA	chr19	33302060	1/1	ENST00000498907.2:c.354del	ENSP00000427514.1:p.Val119SerfsTer41	no	no	no	C	yes	NA	517	204	215	0.51	NA	NA	Sanger
2-040	DNMT3A	chr2	25234373	23/23	ENST00000321117.7:c.2645G>C	ENSP00000324375.5:p.Arg882Pro	yes	yes	yes	A	yes	rs147001633,COSM442676,COSM3356083,COSM1583129,COSM99740,COSM52944	241	114	125	0.52	deleterious (0)	probably damaging(0.997)	Sanger
	RUNX1	chr21	34887027	3/8	ENST00000300305.5:c.167T>C	ENSP00000300305.3:p.Leu56Ser	no	yes	yes	B	no	rs111527738,COSM24756	380	184	191	0.51	tolerated(0.66)	possibly damaging(0.812)	Sanger
	WT1	chr11	32396399	7/10	ENST00000332351.5:c.1106dup	ENSP00000331327.3:p.Arg370ThrfsTer15	no	yes	no	B	yes	COSM1317323,COSM144849,COSM52938	58	12	44	0.79	NA	NA	Sanger[18]
		chr11	32396401	7/10	ENST00000332351.5:c.1105C>G	ENSP00000331327.3:p.Arg369Gly	no	yes	no	B	no	CM971594,COSM35989,COSM21441,COSM30689,COSM28960,COSM28970	51	1	48	0.96	deleterious (0)	probably damaging(0.998)	Sanger[18]
2-041	DNMT3A	chr2	25234374	23/23	ENST00000321117.7:c.2644C>T	ENSP00000324375.5:p.Arg882Cys	yes	yes	yes	A	yes	rs377577594,COSM1166704,COSM1583136,COSM53042,COSM87001	52	31	21	0.40	deleterious (0)	probably damaging(0.997)	Sanger
	TET2	chr4	105234995	3/11	ENST00000380013.6:c.1054del	ENSP00000369351.4:p.Phe352SerfsTer20	no	no	no	C	yes	NA	121	70	47	0.40	NA	NA	NGS
	NPM1	chr5	171410539	12/12	ENST00000517671.3:c.860_863dup	ENSP00000428755.1:p.Trp288CysfsTer12	yes	yes	no	A	yes	COSM1319222,COSM255167,COSM46518,COSM158604	339	216	120	0.36	NA	NA	Sanger
2-044	FLT3	chr13	28049450	8/24	ENST00000241453.9:c.970G>A	ENSP00000241453.7:p.Asp324Asn	no	yes	yes	B	no	rs35602083,CM066823,COSM28040	347	178	168	0.49	tolerated(0.33)	benign(0.035)	Sanger
	KIT	chr4	54723599	8/21	ENST00000288135.5:c.1248_1249del	ENSP00000288135.5:p.Thr417LeufsTer30	yes	no	no	C	no	NA	118	102	15	0.13	NA	NA	NGS
		chr4	54723605	8/21	ENST00000288135.5:c.1254_1255insCTTCTT	ENSP00000288135.5:p.Tyr418_Asp419insPhePhe	yes	yes	no	A	no	COSM1319324	108	88	18	0.17	NA	NA	NGS[18]
		chr4	54723606	8/21	ENST00000288135.5:c.1255_1258del	ENSP00000288135.5:p.Asp419GlyfsTer3	yes	yes	no	A	no	COSM1153,COSM1600400	127	110	15	0.12	NA	NA	NGS[18]

2-045	WT1	chr11	32392014	9/10	ENST00000332351.5:c.1390G>C	ENSP00000331327.3:p.Asp464His	no	yes	yes	B	no	rs28941778,CM003970,CM910413,COSM1353484,COSM27292,COSM27370	26	0	24	1.00	deleterious (0)	probably damaging(1)	Sanger
	NRAS	chr1	114713907	3/7	ENST00000369535.4:c.183A>T	ENSP00000358548.4:p.Gln61His	yes	yes	yes	A	yes	rs121913255,COSM587,COSM585,COSM586	180	112	64	0.36	deleterious (0)	possibly damaging(0.531)	Sanger
	CEBPA	chr19	33301475	1/1	ENST00000498907.2:c.937_939dup	ENSP00000427514.1:p.Lys313dup	no	yes	no	B	no	COSM18099,COSM18541,COSM18901,COSM29139,COSM18640,COSM18103	836	634	200	0.24	NA	NA	Sanger
	CEBPA	chr19	33302118	1/1	ENST00000498907.2:c.296dup	ENSP00000427514.1:p.Gly100ArgfsTer8	no	no	no	C	yes	NA	224	64	117	0.64	NA	NA	Sanger
2-046	DNMT3A	chr2	25234373	23/23	ENST00000321117.7:c.2645G>A	ENSP00000324375.5:p.Arg882His	yes	yes	yes	A	yes	rs147001633,COSM442676,COSM3356083,COSM1583129,COSM99740,COSM52944	70	37	33	0.47	deleterious (0.03)	probably damaging(0.993)	Sanger
	ASXL1	chr20	32436018	13/13	ENST00000375687.6:c.3306G>T	ENSP00000364839.4:p.Glu1102Asp	yes	yes	yes	A	no	rs139115934,COSM36205,COSM1155723	186	98	86	0.47	deleterious (0.03)	probably damaging(0.911)	Sanger
	NPM1	chr5	171410539	12/12	ENST00000517671.3:c.860_863dup	ENSP00000428755.1:p.Trp288CysfsTer12	yes	yes	no	A	yes	COSM1319222,COSM255167,COSM46518,COSM158604	938	617	317	0.34	NA	NA	Sanger
2-048	DNMT3A	chr2	25234373	23/23	ENST00000321117.7:c.2645G>A	ENSP00000324375.5:p.Arg882His	yes	yes	yes	A	yes	rs147001633,COSM442676,COSM3356083,COSM1583129,COSM99740,COSM52944	50	27	21	0.44	deleterious (0.03)	probably damaging(0.993)	Sanger
	FLT3	chr13	28018503	20/24	ENST00000241453.9:c.2505T>G	ENSP00000241453.7:p.Asp835Glu	yes	yes	yes	A	yes	rs121913487,KinMutBase_FLT3_DNA:g.53111T>A,KinMutBase_FLT3_DNA:g.53111T>G,COSM788,COSM787	535	242	275	0.53	deleterious (0)	probably damaging(0.997)	Sanger
	NPM1	chr5	171410539	12/12	ENST00000517671.3:c.860_863dup	ENSP00000428755.1:p.Trp288CysfsTer12	yes	yes	no	A	yes	COSM1319222,COSM255167,COSM46518,COSM158604	617	413	202	0.33	NA	NA	Sanger
	CEBPA	chr19	33301484	1/1	ENST00000498907.2:c.930dup	ENSP00000427514.1:p.Gln311AlafsTer10	no	yes	no	B	yes	COSM29601,COSM18472,COSM18648,COSM18902,COSM29259,COSM1318331,COSM29226	346	213	125	0.37	NA	NA	Sanger
2-049	DNMT3A	chr2	25234373	23/23	ENST00000321117.7:c.2645G>C	ENSP00000324375.5:p.Arg882Pro	yes	yes	yes	A	yes	rs147001633,COSM442676,COSM3356083,COSM1583129,COSM99740,COSM52944	41	26	14	0.35	deleterious (0)	probably damaging(0.997)	Sanger
	RUNX1	chr21	34887027	3/8	ENST00000300305.5:c.167T>C	ENSP00000300305.3:p.Leu56Ser	no	yes	yes	B	no	rs111527738,COSM24756	113	61	49	0.45	tolerated(0.66)	possibly damaging(0.812)	Sanger
	NPM1	chr5	171410539	12/12	ENST00000517671.3:c.860_863dup	ENSP00000428755.1:p.Trp288CysfsTer12	yes	yes	no	A	yes	COSM1319222,COSM255167,COSM46518,COSM158604	489	304	185	0.38	NA	NA	Sanger
	IDH2	chr15	90088702	4/11	ENST00000330062.5:c.419G>A	ENSP00000331897.3:p.Arg140Gln	yes	yes	yes	A	yes	rs121913502,COSM41875,COSM41590	320	178	141	0.44	deleterious (0)	probably damaging(1)	Sanger
	NRAS	chr1	114716126	2/7	ENST00000369535.4:c.35G>A	ENSP00000358548.4:p.Gly12Asp	yes	yes	no	A	yes	COSM565,COSM566,COSM564	120	66	52	0.44	deleterious (0)	possibly damaging(0.726)	Sanger
2-050	FLT3	chr13	28015643	21/24	ENST00000241453.9:c.2600T>C	ENSP00000241453.7:p.Ile867Thr	no	no	no	C	no	NA	658	569	88	0.13	deleterious (0.01)	possibly damaging(0.832)	NGS
	RUNX1	chr21	34886866	3/8	ENST00000300305.5:c.328A>C	ENSP00000300305.3:p.Lys110Gln	no	no	yes	D	no	rs121912498	453	6	446	0.99	deleterious (0)	probably damaging(0.995)	Sanger
	TET2	chr4	105272692	10/11	ENST00000380013.6:c.4317dup	ENSP00000369351.4:p.Arg1440ThrfsTer38	no	no	no	C	yes	NA	55	29	21	0.39	NA	NA	Sanger
	NRAS	chr1	114713907	3/7	ENST00000369535.4:c.183A>C	ENSP00000358548.4:p.Gln61His	yes	yes	yes	A	yes	rs121913255,COSM587,COSM585,COSM586	124	64	60	0.48	deleterious (0)	possibly damaging(0.531)	Sanger
2-051	NPM1	chr5	171410539	12/12	ENST00000517671.3:c.860_863dup	ENSP00000428755.1:p.Trp288CysfsTer12	yes	yes	no	A	yes	COSM1319222,COSM255167,COSM46518,COSM158604	2715	1782	924	0.34	NA	NA	Sanger
	IDH2	chr15	90088702	4/11	ENST00000330062.5:c.419G>A	ENSP00000331897.3:p.Arg140Gln	yes	yes	yes	A	yes	rs121913502,COSM41875,COSM41590	911	485	418	0.46	deleterious (0)	probably damaging(1)	Sanger
2-052	IDH1	chr2	208248388	4/10	ENST00000345146.4:c.395G>A	ENSP00000260985.2:p.Arg132His	yes	yes	yes	A	yes	COSM28746,COSM28750,rs121913500	188	109	79	0.42	deleterious (0)	possibly damaging(0.813)	Sanger
2-053	KIT	chr4	54733154	17/21	ENST00000288135.5:c.2446G>T	ENSP00000288135.5:p.Asp816Tyr	yes	yes	yes	A	yes	rs121913506,KinMutBase_KIT_DNA:g.76139G>C,KinMutBase_KIT_DNA:g.76139G>T,COSM1313,COSM1311,COSM1310	414	288	124	0.30	deleterious (0)	probably damaging(0.963)	Sanger
	NRAS	chr1	114713907	3/7	ENST00000369535.4:c.183A>C	ENSP00000358548.4:p.Gln61His	yes	yes	yes	A	yes	rs121913255,COSM587,COSM585,COSM586	233	203	23	0.10	deleterious (0)	possibly damaging(0.531)	NGS
2-054	DNMT3A	chr2	25240727	18/23	ENST00000321117.7:c.2086C>T	ENSP00000324375.5:p.Gln696Ter	no	yes	no	B	yes	COSM1583101	120	78	39	0.33	NA	NA	Sanger
3-001	TET2	chr4	105234042	3/11	ENST00000380013.6:c.100C>T	ENSP00000369351.4:p.Leu34Phe	no	no	yes	D	no	rs111948941	40	26	12	0.32	deleterious (0.01)	benign(0.384)	Sanger
	TET2	chr4	105272692	10/11	ENST00000380013.6:c.4317dup	ENSP00000369351.4:p.Arg1440ThrfsTer38	no	no	no	C	yes	NA	67	55	10	0.15	NA	NA	NGS
3-003	WT1	chr11	32392029	9/10	ENST00000332351.5:c.1374_1375insCTCT	ENSP00000331327.3:p.Lys459LeufsTer2	no	no	no	C	yes	NA	18	7	8	0.53	NA	NA	Sanger
	CEBPA	chr19	33302214	1/1	ENST00000498907.2:c.196_200dup	ENSP00000427514.1:p.Tyr67Ter	no	yes	no	B	yes	COSM330607,COSM27453,COSM27463	678	368	305	0.45	NA	NA	Sanger
3-004	DNMT3A	chr2	25234374	23/23	ENST00000321117.7:c.2644C>T	ENSP00000324375.5:p.Arg882Cys	yes	yes	yes	A	yes	rs377577594,COSM1166704,COSM1583136,COSM53042,COSM87001	113	53	60	0.53	deleterious (0)	probably damaging(0.997)	Sanger
3-005	DNMT3A	chr2	25234373	23/23	ENST00000321117.7:c.2645G>A	ENSP00000324375.5:p.Arg882His	yes	yes	yes	A	yes	rs147001633,COSM442676,COSM3356083,COSM1583129,COSM99740,COSM52944	36	20	15	0.43	deleterious (0.03)	probably damaging(0.993)	Sanger
	TET2	chr4	105234042	3/11	ENST00000380013.6:c.100C>T	ENSP00000369351.4:p.Leu34Phe	no	no	yes	D	no	rs111948941	97	54	41	0.43	deleterious (0.01)	benign(0.384)	Sanger

	TET2	chr4	105236491	3/11	ENST00000380013.6:c.2549A>G	ENSP00000369351.4:p.His850Arg	no	no	yes	D	no	rs369370690	90	46	42	0.48	tolerated(0.18)	benign(0.001)	Sanger
	NPM1	chr5	171410539	12/12	ENST00000517671.3:c.860_863dup	ENSP00000428755.1:p.Trp288CysfsTer12	yes	yes	no	A	yes	COSM1319222,COSM255167,COSM46518,COSM158604	386	262	124	0.32	NA	NA	Sanger
	DNMT3A	chr2	25245284	13/23	ENST00000321117.7:c.1522del	ENSP00000324375.5:p.Leu508SerfsTer143	no	no	no	C	yes	NA	82	43	40	0.48	NA	NA	Sanger
3-006	IDH1	chr2	208243577	6/10	ENST00000345146.4:c.548A>G	ENSP00000260985.2:p.Tyr183Cys	no	no	yes	D	no	rs34599179,CM073129	167	79	84	0.52	deleterious (0)	probably damaging(0.994)	Sanger
	RUNX1	chr21	34792566	8/8	ENST00000300305.5:c.1011dup	ENSP00000300305.3:p.Ala338ArgfsTer262	no	yes	no	B	yes	COSM1717858	507	290	217	0.43	NA	NA	Sanger
	IDH2	chr15	90088702	4/11	ENST00000330062.5:c.419G>A	ENSP00000331897.3:p.Arg140Gln	yes	yes	yes	A	yes	rs121913502,COSM41875,COSM41590	952	515	433	0.46	deleterious (0)	probably damaging(1)	Sanger
	FLT3	chr13	28049450	8/24	ENST00000241453.9:c.970G>A	ENSP00000241453.7:p.Asp324Asn	no	yes	yes	B	no	rs35602083,CM066823,COSM28040	351	200	148	0.43	tolerated(0.33)	benign(0.035)	Sanger
3-007	KIT	chr4	54733155	17/21	ENST00000288135.5:c.2447A>T	ENSP00000288135.5:p.Asp816Val	yes	yes	yes	A	yes	rs121913507,KinMutBase_KIT_DNA:g.76140A>T,CM952169,COSM1314,COSM24675,COSM12711	369	131	237	0.64	deleterious (0)	probably damaging(0.977)	Sanger
	TET2	chr4	105272692	10/11	ENST00000380013.6:c.4317dup	ENSP00000369351.4:p.Arg1440ThrfsTer38	no	no	no	C	yes	NA	78	66	9	0.12	NA	NA	NGS
	ASXL1	chr20	32435346	13/13	ENST00000375687.6:c.2634T>A	ENSP00000364839.4:p.Ser878Arg	yes	no	no	C	no	NA	157	89	68	0.43	tolerated(0.09)	benign(0.019)	Sanger
	DNMT3A	chr2	25240313	19/23	ENST00000321117.7:c.2311C>T	ENSP00000324375.5:p.Arg771Ter	no	yes	no	B	yes	COSM1583105,COSM231563	101	61	32	0.34	NA	NA	Sanger
3-009	IDH1	chr2	208248389	4/10	ENST00000345146.4:c.394C>G	ENSP00000260985.2:p.Arg132Gly	yes	yes	yes	A	yes	COSM28749,COSM28747,COSM28748,rs121913499	183	79	102	0.56	deleterious (0)	probably damaging(0.998)	Sanger
	NPM1	chr5	171410539	12/12	ENST00000517671.3:c.860_863dup	ENSP00000428755.1:p.Trp288CysfsTer12	yes	yes	no	A	yes	COSM1319222,COSM255167,COSM46518,COSM158604	1933	1210	717	0.37	NA	NA	Sanger
3-010	FLT3	chr13	28018503	20/24	ENST00000241453.9:c.2505T>G	ENSP00000241453.7:p.Asp835Glu	yes	yes	yes	A	yes	rs121913487,KinMutBase_FLT3_DNA:g.53111T>A,KinMutBase_FLT3_DNA:g.53111T>G,COSM788,COSM787	526	411	103	0.20	deleterious (0)	probably damaging(0.997)	Sanger
	IDH1	chr2	208243577	6/10	ENST00000345146.4:c.548A>G	ENSP00000260985.2:p.Tyr183Cys	no	no	yes	D	no	rs34599179,CM073129	86	42	44	0.51	deleterious (0)	probably damaging(0.994)	Sanger
	FLT3	chr13	28034113	14/24	ENST00000241453.9:c.1773_1805dup	ENSP00000241453.7:p.Leu601_Lys602insAsnValAspPheArgGluTyrGluTyrAspLeu	yes	yes	no	A	no	COSM19882,COSM1317922	578	517	60	0.10	NA	NA	Sanger
3-011	NPM1	chr5	171410539	12/12	ENST00000517671.3:c.860_863dup	ENSP00000428755.1:p.Trp288CysfsTer12	yes	yes	no	A	yes	COSM1319222,COSM255167,COSM46518,COSM158604	1308	825	479	0.37	NA	NA	Sanger
	CEBPA	chr19	33302046	1/1	ENST00000498907.2:c.368dup	ENSP00000427514.1:p.Ala124SerfsTer46	no	no	no	C	yes	NA	373	168	154	0.48	NA	NA	Sanger
	DNMT3A	chr2	25234374	23/23	ENST00000321117.7:c.2644C>A	ENSP00000324375.5:p.Arg882Ser	yes	yes	yes	A	no	rs377577594,COSM1166704,COSM1583136,COSM53042,COSM87001	176	90	85	0.49	deleterious (0)	probably damaging(0.996)	Sanger
3-012	NPM1	chr5	171410539	12/12	ENST00000517671.3:c.860_863dup	ENSP00000428755.1:p.Trp288CysfsTer12	yes	yes	no	A	yes	COSM1319222,COSM255167,COSM46518,COSM158604	1544	994	546	0.35	NA	NA	Sanger
	WT1	chr11	32434715	1/10	ENST00000332351.5:c.630dup	ENSP00000331327.3:p.Ala211ArgfsTer37	no	no	no	C	yes	NA	54	34	20	0.37	NA	NA	Sanger
	IDH1	chr2	208243532	6/10	ENST00000345146.4:c.593A>G	ENSP00000260985.2:p.Gln198Arg	no	no	yes	D	no	rs374340555	395	208	186	0.47	tolerated(0.19)	benign(0.016)	Sanger
3-013	TET2	chr4	105235995	3/11	ENST00000380013.6:c.2053C>T	ENSP00000369351.4:p.Gln685Ter	no	no	no	C	yes	NA	80	44	32	0.42	NA	NA	Sanger
	NPM1	chr5	171410539	12/12	ENST00000517671.3:c.860_863dup	ENSP00000428755.1:p.Trp288CysfsTer12	yes	yes	no	A	yes	COSM1319222,COSM255167,COSM46518,COSM158604	998	643	351	0.35	NA	NA	Sanger
3-014	TET2	chr4	105272692	10/11	ENST00000380013.6:c.4317dup	ENSP00000369351.4:p.Arg1440ThrfsTer38	no	no	no	C	yes	NA	137	121	14	0.10	NA	NA	NGS
	TP53	chr17	7674220	7/11	ENST00000269305.6:c.743G>A	ENSP00000269305.4:p.Arg248Gln	no	yes	yes	B	yes	rs11540652,TP53_g.13380del,TP53_g.13380G>A,TP53_g.13380G>T,TP53_g.13380G>C,CM920675,COSM340107,COSM241996,COSM99021,COSM1646858,COSM1646857,COSM1640830,COSM3356964,COSM3378346,COSM340106,COSM241994,COSM99020,COSM340105,COSM241995,COSM99602,COSM11491,COSM46135,COSM10662,COSM6549	249	187	52	0.22	deleterious (0)	probably damaging(1)	Sanger
3-015	TET2	chr4	105272591	10/11	ENST00000380013.6:c.4210C>T	ENSP00000369351.4:p.Arg1404Ter	no	yes	no	B	yes	COSM42037	88	76	11	0.13	NA	NA	NGS
	IDH2	chr15	90088702	4/11	ENST00000330062.5:c.419G>A	ENSP00000331897.3:p.Arg140Gln	yes	yes	yes	A	yes	rs121913502,COSM41875,COSM41590	645	408	233	0.36	deleterious (0)	probably damaging(1)	Sanger
3-016	IDH2	chr15	90088702	4/11	ENST00000330062.5:c.419G>A	ENSP00000331897.3:p.Arg140Gln	yes	yes	yes	A	yes	rs121913502,COSM41875,COSM41590	945	519	425	0.45	deleterious (0)	probably damaging(1)	Sanger
3-017	DNMT3A	chr2	25234373	23/23	ENST00000321117.7:c.2645G>A	ENSP00000324375.5:p.Arg882His	yes	yes	yes	A	yes	rs147001633,COSM442676,COSM3356083,COSM1583129,COSM99740,COSM52944	44	24	19	0.44	deleterious (0.03)	probably damaging(0.993)	Sanger
	FLT3	chr13	28018505	20/24	ENST00000241453.9:c.2503G>T	ENSP00000241453.7:p.Asp835Tyr	yes	yes	no	A	yes	KinMutBase_FLT3_DNA:g.53109G>T,KinMutBase_FLT3_DNA:g.53109G>C,KinMutBase_FLT3_DNA:g.53109	607	523	62	0.11	deleterious (0)	probably damaging(1)	NGS

												G>A,KinMutBase_FLT3_DNA:g.53109delG,COSM783,COSM789,COSM785							
	IDH1	chr2	208251458	3/10	ENST00000345146.4:c.94T>G	ENSP00000260985.2:p.Phe32Val	no	yes	yes	B	no	COSM1581043,rs142923780	234	111	122	0.52	tolerated(0.05)	benign(0.025)	Sanger
	RUNX1	chr21	34792448	8/8	ENST00000300305.5:c.1129dup	ENSP00000300305.3:p.Tyr377LeufsTer223	no	no	no	C	yes	NA	253	140	110	0.44	NA	NA	Sanger
	RUNX1	chr21	34887027	3/8	ENST00000300305.5:c.167T>C	ENSP00000300305.3:p.Leu56Ser	no	yes	yes	B	no	rs111527738,COSM24756	189	94	89	0.49	tolerated(0.66)	possibly damaging(0.812)	Sanger
	TET2	chr4	105234763	3/11	ENST00000380013.6:c.822del	ENSP00000369351.4:p.Asn275IlefsTer18	no	yes	no	B	yes	COSM1318631,COSM1318632,COSM43437	118	63	52	0.45	NA	NA	Sanger
	TET2	chr4	105259626	7/11	ENST00000380013.6:c.3812dup	ENSP00000369351.4:p.Cys1271TrpfsTer29	no	yes	no	B	yes	COSM43483	74	43	31	0.42	NA	NA	Sanger
	NRAS	chr1	114716123	2/7	ENST00000369535.4:c.38G>A	ENSP00000358548.4:p.Gly13Asp	yes	yes	no	A	yes	NRASbase_D0125:g.40791G>A,CM071907,COSM574,COSM575,COSM573	125	95	29	0.24	deleterious (0.05)	benign(0.004)	NGS
3-018	IDH1	chr2	208248388	4/10	ENST00000345146.4:c.395G>A	ENSP00000260985.2:p.Arg132His	yes	yes	yes	A	yes	COSM28746,COSM28750,rs121913500	97	46	51	0.53	deleterious (0)	possibly damaging(0.813)	Sanger
3-019	DNMT3A	chr2	25244259	15/23	ENST00000321117.7:c.1747T>A	ENSP00000324375.5:p.Cys583Ser	no	no	no	C	no	NA	27	15	10	0.40	deleterious (0)	probably damaging(0.996)	Sanger
	FLT3	chr13	28035621	12/24	ENST00000241453.9:c.1471G>T	ENSP00000241453.7:p.Val491Leu	no	yes	no	B	no	COSM116249	292	193	99	0.34	tolerated(0.36)	benign(0.022)	Sanger
	NPM1	chr5	171410539	12/12	ENST00000517671.3:c.860_863dup	ENSP00000428755.1:p.Trp288CysfsTer12	yes	yes	no	A	yes	COSM1319222,COSM255167,COSM46518,COSM158604	718	485	229	0.32	NA	NA	Sanger
	NRAS	chr1	114716126	2/7	ENST00000369535.4:c.35G>A	ENSP00000358548.4:p.Gly12Asp	yes	yes	no	A	yes	COSM565,COSM566,COSM564	115	81	32	0.28	deleterious (0)	possibly damaging(0.726)	Sanger
3-020	DNMT3A	chr2	25234308	23/23	ENST00000321117.7:c.2710C>A	ENSP00000324375.5:p.Pro904Thr	no	yes	no	B	no	COSM1583133	93	54	37	0.41	deleterious (0)	probably damaging(0.999)	Sanger
	ASXL1	chr20	32435669	13/13	ENST00000375687.6:c.2957A>G	ENSP00000364839.4:p.Asn986Ser	yes	yes	yes	A	no	rs145132837,COSM96383	240	121	118	0.49	tolerated(0.25)	benign(0.091)	Sanger
	NPM1	chr5	171410539	12/12	ENST00000517671.3:c.860_863dup	ENSP00000428755.1:p.Trp288CysfsTer12	yes	yes	no	A	yes	COSM1319222,COSM255167,COSM46518,COSM158604	1295	882	403	0.31	NA	NA	Sanger
	IDH2	chr15	90087472	6/11	ENST00000330062.5:c.782G>A	ENSP00000331897.3:p.Arg261His	no	no	yes	D	no	rs118101777	420	207	209	0.50	deleterious (0)	probably damaging(0.98)	Sanger
	NRAS	chr1	114716126	2/7	ENST00000369535.4:c.35G>A	ENSP00000358548.4:p.Gly12Asp	yes	yes	no	A	yes	COSM565,COSM566,COSM564	154	87	58	0.40	deleterious (0)	possibly damaging(0.726)	Sanger
3-021	DNMT3A	chr2	25234413	23/23	ENST00000321117.7:c.2604del	ENSP00000324375.5:p.Phe868LeufsTer13	no	no	no	C	yes	NA	67	51	15	0.23	NA	NA	Sanger
	NPM1	chr5	171410539	12/12	ENST00000517671.3:c.860_863dup	ENSP00000428755.1:p.Trp288CysfsTer12	yes	yes	no	A	yes	COSM1319222,COSM255167,COSM46518,COSM158604	710	579	131	0.18	NA	NA	Sanger
	IDH2	chr15	90088702	4/11	ENST00000330062.5:c.419G>A	ENSP00000331897.3:p.Arg140Gln	yes	yes	yes	A	yes	rs121913502,COSM41875,COSM41590	378	257	117	0.31	deleterious (0)	probably damaging(1)	Sanger
3-022	NRAS	chr1	114713908	3/7	ENST00000369535.4:c.182A>G	ENSP00000358548.4:p.Gln61Arg	yes	yes	no	A	yes	COSM583,COSM584,COSM582	100	64	34	0.35	tolerated(0.06)	benign(0.006)	Sanger
3-023	TET2	chr4	105261769	8/11	ENST00000380013.6:c.3965T>G	ENSP00000369351.4:p.Leu1322Arg	no	yes	no	B	yes	COSM211728	26	5	21	0.81	deleterious (0)	probably damaging(0.999)	Sanger
	NPM1	chr5	171410540	12/12	ENST00000517671.3:c.863_864insCCTG	ENSP00000428755.1:p.Trp288CysfsTer12	yes	yes	no	A	yes	COSM20866,COSM20863,COSM1658759,COSM28937	1902	1381	517	0.27	NA	NA	Sanger
	IDH2	chr15	90088702	4/11	ENST00000330062.5:c.419G>A	ENSP00000331897.3:p.Arg140Gln	yes	yes	yes	A	yes	rs121913502,COSM41875,COSM41590	568	475	91	0.16	deleterious (0)	probably damaging(1)	NGS
3-024	TP53	chr17	7674259	7/11	ENST00000269305.6:c.704A>G	ENSP00000269305.4:p.Asn235Ser	no	yes	yes	B	no	rs144340710,TP53_g.13341del,TP53_g.13341A>T,TP53_g.13341A>C,TP53_g.13341A>G,CM951230,COSM1581853,COSM1581852,COSM1581851,COSM45854,COSM45620,COSM43860,COSM43616	113	47	44	0.48	tolerated(0.22)	benign(0.298)	Sanger
	ASXL1	chr20	32434599	13/13	ENST00000375687.6:c.1900_1922del	ENSP00000364839.4:p.Glu635ArgfsTer15	yes	yes	no	A	yes	COSM51200	49	41	6	0.13	NA	NA	Sanger
3-025	CEBPA	chr19	33301825	1/1	ENST00000498907.2:c.584_589dup	ENSP00000427514.1:p.His195_Pro196dup	no	no	no	C	no	NA	114	97	14	0.13	NA	NA	Sanger
3-026	WT1	chr11	32396361	7/10	ENST00000332351.5:c.1141_1144dup	ENSP00000331327.3:p.Ala382ValfsTer4	no	yes	no	B	yes	COSM1166613,COSM97182,COSM21392	67	52	12	0.19	NA	NA	NGS
3-027	FLT3	chr13	28034125	14/24	ENST00000241453.9:c.1770_1793dup	ENSP00000241453.7:p.Tyr597_Glu598insAspTyrValAspPheArgGluTyr	yes	yes	no	A	no	COSM19961,COSM1317913,COSM1317912	1259	1078	169	0.13	NA	NA	Sanger
	NPM1	chr5	171410540	12/12	ENST00000517671.3:c.863_864insCCTG	ENSP00000428755.1:p.Trp288CysfsTer12	yes	yes	no	A	yes	COSM20866,COSM20863,COSM1658759,COSM28937	1817	1165	645	0.36	NA	NA	Sanger
3-029	IDH2	chr15	90084321	11/11	ENST00000330062.5:c.1304C>T	ENSP00000331897.3:p.Thr435Met	no	no	yes	D	no	rs118053940	751	390	359	0.48	tolerated(0.14)	benign(0.153)	Sanger
3-030	ASXL1	chr20	32434733	13/13	ENST00000375687.6:c.2024dup	ENSP00000364839.4:p.Glu676Ter	yes	no	no	C	yes	NA	120	91	30	0.25	NA	NA	Sanger
	IDH2	chr15	90088702	4/11	ENST00000330062.5:c.419G>A	ENSP00000331897.3:p.Arg140Gln	yes	yes	yes	A	yes	rs121913502,COSM41875,COSM41590	867	434	428	0.50	deleterious (0)	probably damaging(1)	Sanger

3-031	FLT3	chr13	28049450	8/24	ENST00000241453.9:c.970G>A	ENSP00000241453.7:p.Asp324Asn	no	yes	yes	B	no	rs35602083,CM066823,COSM28040	2619	1353	1255	0.48	tolerated(0.33)	benign(0.035)	Sanger
	RUNX1	chr21	34886881	3/8	ENST00000300305.5:c.313C>A	ENSP00000300305.3:p.His105Asn	no	no	no	C	no	NA	593	6	584	0.99	deleterious (0.01)	benign(0.363)	Sanger
3-032	RUNX1	chr21	34887027	3/8	ENST00000300305.5:c.167T>C	ENSP00000300305.3:p.Leu56Ser	no	yes	yes	B	no	rs111527738,COSM24756	231	122	105	0.46	tolerated(0.66)	possibly damaging(0.812)	Sanger
	NPM1	chr5	171410542	12/12	ENST00000517671.3:c.863_864insCCGG	ENSP00000428755.1:p.Trp288CysfsTer12	yes	yes	no	A	yes	COSM25117,COSM1319220,COSM20865,COSM1319219	1433	1106	318	0.22	NA	NA	Sanger
	CEBPA	chr19	33301825	1/1	ENST00000498907.2:c.584_589dup	ENSP00000427514.1:p.His195_Pro196dup	no	no	no	C	no	NA	227	188	32	0.14	NA	NA	NGS
3-034	IDH1	chr2	208248389	4/10	ENST00000345146.4:c.394C>T	ENSP00000260985.2:p.Arg132Cys	yes	yes	yes	A	yes	COSM28749,COSM28747,COSM28748,rs121913499	200	95	104	0.52	deleterious (0.03)	possibly damaging(0.907)	Sanger
	TET2	chr4	105275662	11/11	ENST00000380013.6:c.5152G>T	ENSP00000369351.4:p.Val1718Leu	no	yes	yes	B	no	rs142312318,COSM41742	65	30	35	0.54	tolerated(0.34)	benign(0.006)	Sanger
	NPM1	chr5	171410539	12/12	ENST00000517671.3:c.860_863dup	ENSP00000428755.1:p.Trp288CysfsTer12	yes	yes	no	A	yes	COSM1319222,COSM255167,COSM46518,COSM158604	2714	1699	1003	0.37	NA	NA	Sanger
	CEBPA	chr19	33302176	1/1	ENST00000498907.2:c.238dup	ENSP00000427514.1:p.Asp80GlyfsTer28	no	yes	no	B	yes	COSM18618	661	357	289	0.45	NA	NA	Sanger
3-035	IDH1	chr2	208248389	4/10	ENST00000345146.4:c.394C>A	ENSP00000260985.2:p.Arg132Ser	yes	yes	yes	A	yes	COSM28749,COSM28747,COSM28748,rs121913499	98	30	67	0.69	deleterious (0)	probably damaging(0.997)	Sanger
	NPM1	chr5	171410539	12/12	ENST00000517671.3:c.860_863dup	ENSP00000428755.1:p.Trp288CysfsTer12	yes	yes	no	A	yes	COSM1319222,COSM255167,COSM46518,COSM158604	1571	996	561	0.36	NA	NA	Sanger
	CEBPA	chr19	33301984	1/1	ENST00000498907.2:c.430dup	ENSP00000427514.1:p.Glu144GlyfsTer26	no	no	no	C	yes	NA	211	79	103	0.57	NA	NA	Sanger
3-037	DNMT3A	chr2	25300227	3/23	ENST00000321117.7:c.89A>C	ENSP00000324375.5:p.Glu30Ala	no	yes	yes	B	no	rs143730975,COSM307361	55	29	24	0.45	deleterious (0)	benign(0.292)	Sanger
	NPM1	chr5	171410539	12/12	ENST00000517671.3:c.860_863dup	ENSP00000428755.1:p.Trp288CysfsTer12	yes	yes	no	A	yes	COSM1319222,COSM255167,COSM46518,COSM158604	1422	943	472	0.33	NA	NA	Sanger
	IDH2	chr15	90088702	4/11	ENST00000330062.5:c.419G>A	ENSP00000331897.3:p.Arg140Gln	yes	yes	yes	A	yes	rs121913502,COSM41875,COSM41590	923	506	407	0.45	deleterious (0)	probably damaging(1)	Sanger
3-038	DNMT3A	chr2	25239194	20/23	ENST00000321117.7:c.2340_2343del	ENSP00000324375.5:p.Ile780MetfsTer21	no	no	no	C	yes	NA	111	83	26	0.24	NA	NA	Sanger
	IDH2	chr15	90088702	4/11	ENST00000330062.5:c.419G>A	ENSP00000331897.3:p.Arg140Gln	yes	yes	yes	A	yes	rs121913502,COSM41875,COSM41590	1189	977	207	0.17	deleterious (0)	probably damaging(1)	NGS
3-039	FLT3	chr13	28028243	16/24	ENST00000241453.9:c.1988A>G	ENSP00000241453.7:p.Lys663Arg	no	yes	no	B	no	COSM1366193	1114	710	397	0.36	deleterious (0)	probably damaging(1)	Sanger
3-040	DNMT3A	chr2	25246023	12/23	ENST00000321117.7:c.1470dup	ENSP00000324375.5:p.Glu491Ter	no	no	no	C	yes	NA	49	40	8	0.17	NA	NA	Sanger
	IDH2	chr15	90088606	4/11	ENST00000330062.5:c.515G>A	ENSP00000331897.3:p.Arg172Lys	yes	yes	yes	A	yes	rs121913503,COSM1636997,COSM33733,COSM33732	404	243	158	0.39	deleterious (0)	probably damaging(1)	Sanger
3-041	DNMT3A	chr2	25234373	23/23	ENST00000321117.7:c.2645G>A	ENSP00000324375.5:p.Arg882His	yes	yes	yes	A	yes	rs147001633,COSM442676,COSM3356083,COSM1583129,COSM99740,COSM52944	38	24	12	0.33	deleterious (0.03)	probably damaging(0.993)	Sanger
	NPM1	chr5	171410539	12/12	ENST00000517671.3:c.860_863dup	ENSP00000428755.1:p.Trp288CysfsTer12	yes	yes	no	A	yes	COSM1319222,COSM255167,COSM46518,COSM158604	830	562	267	0.32	NA	NA	Sanger
3-042	TET2	chr4	105236554	3/11	ENST00000380013.6:c.2613dup	ENSP00000369351.4:p.Val872CysfsTer29	no	no	no	C	yes	NA	58	36	22	0.38	NA	NA	Sanger
	TET2	chr4	105269725	9/11	ENST00000380013.6:c.4160A>G	ENSP00000369351.4:p.Asn1387Ser	no	no	no	C	yes	NA	89	48	40	0.45	deleterious (0)	probably damaging(1)	Sanger
	TET2	chr4	105272692	10/11	ENST00000380013.6:c.4317dup	ENSP00000369351.4:p.Arg1440ThrfsTer38	no	no	no	C	yes	NA	103	89	13	0.13	NA	NA	NGS
	ASXL1	chr20	32434789	13/13	ENST00000375687.6:c.2077C>T	ENSP00000364839.4:p.Arg693Ter	yes	yes	yes	A	yes	rs373221034,COSM51388	144	92	52	0.36	NA	NA	Sanger
	CEBPA	chr19	33302346	1/1	ENST00000498907.2:c.68dup	ENSP00000427514.1:p.His24AlafsTer84	no	yes	yes	B	yes	rs137852729,COSM18922,COSM29580	2818	1634	1187	0.42	NA	NA	Sanger

[1] Position of the variant in the GRCh38 human genome.

[2] Exon location of the variant / total number of exons of the gene.

[3] HGVSc; coding sequence name as identified by the Human Genome Variation Society (http://www.hgvs.org/).

[4] HGVSp; protein sequence name as identified by the Human Genome Variation Society (http://www.hgvs.org/).

[5] HS; indicated whether the variant occurs in a hotspot (HS) region as identified in Table SVI.

[6] COS; indicated whether the variant is known as cancer mutation in COSMIC (catalogue of somatic mutations in cancer; http://cancer.sanger.ac.uk/cosmic).

[7] SNP; indicated whether the variant is known as single nucleotide polymorphism in dbSNP (https://www.ncbi.nlm.nih.gov/projects/SNP).

[8] Class; A, hotspot variant known in COSMIC (known or unknown as SNP); B, variant not in a hotspot but known in COSMIC (known or unknown as SNP); C, variant unknown in COSMIC and unknown as SNP; D, variant unknown in COSMIC and known as SNP.

[9] Jaiswal; indicated whether the variant is a recurrent mutation in myeloid malignancies by Jaiswal *et al.* (NEJM 2017).

[10] Existing variation; indicated is the identity of the variant as reported in COSMIC and dbSNP.

[11] DP; Quality Read Depth of bases with Phred score >= 15.

[12] RD; depth of reference-supporting bases.

[13] AD; depth of variant-supporting bases.

[14] VAF; variant allele frequency.

[15] SIFT; tool to predict the possible impact of an amino acid substitution on protein function (http://sift.jcvi.org/).

[16] PolyPhen; tool to predict the possible impact of an amino acid substitution on the structure and function of a human protein (http://genetics.bwh.harvard.edu/pph2/).

[17] Validation; small variants were validated by RT-PCR followed by Sanger sequencing or MiSeq NGS. Primers used for validation are shown in Table SVIII.

[18] Targeted re-sequencing showed that these variants are located on the same allele.

Table SVIII. Primers used for validation of small variants[1].

Primer	Sequence (5'- 3')	ENST transcript	Position		Size (bp)
			start (cds)	stop (cds)	
ASXL1-1F<br>ASXL1-1R	GGTGGTTAAAGGTCAGCCCA<br>AGCCCATCTGTGAGTCCAAC	ENST00000375687	1749<br>2228	1768<br>2209	480
ASXL1-2F<br>ASXL1-2R	CCTGCAGACAATGGTCCCAT<br>TCAGAGGCTGTATCCGTGGA	ENST00000375687	2422<br>3011	2441<br>2992	590
ASXL1-3F<br>ASXL1-3R	GTCCGCCAAAAGATCCCAGA<br>TGTCTGCTCTGGACCAAAGG	ENST00000375687	3214<br>3861	3233<br>3842	648
CEBPA-1F<br>CEBPA-1R	CCATGCCGGGAGAACTCTAA<br>CCTTGGCCTTCTCCTGCTG	ENST00000498907	-26<br>277	-7<br>259	304
CEBPA-2F<br>CEBPA-2R	CGACTTCTACGAGGCGGAG<br>GGCTCCTGCTTGATCACCAG	ENST00000498907	12<br>491	30<br>472	480
CEBPA-3F<br>CEBPA-3R	CCGATGAGCAGCCACCTG<br>TCGTACAGGGGCTCCAGC	ENST00000498907	40<br>443	47<br>426	404
CEBPA-4F<br>CEBPA-4R	CCGCTACCTGGACGGCAG<br>GCAGGTGCATGGTGGTCT	ENST00000498907	408<br>661	425<br>644	254
CEBPA-5F<br>CEBPA-5R	AGGAGGATGAAGCCAAGCAG<br>CTTGACCAAGGAGCTCTCTGG	ENST00000498907	497<br>1056	516<br>1036	560
CEBPA-6F<br>CEBPA-6R	AGAACAGCAACGAGTACCGG<br>CCAAACCACTCCCTGGGTC	ENST00000498907	989<br>1288	1008<br>1270	300
CEBPA-7F<br>CEBPA-7R	AGCGCCGCCTTCGGCTTT<br>CCCGGGTAGTCAAAGTCGCCG	ENST00000498907	82<br>329	99<br>309	248
CEBPA-8F<br>CEBPA-8R	CGGTGGACAAGAACAGCAACG<br>CAGGCCAGGCTTTCAGGAGG	ENST00000498907	830<br>1077*140	850<br>1077*120	388
DNMT3A-1F<br>DNMT3A-1R	ATTCCTTCCCCAAAGCCCAG<br>GGGTCCTTTGGCGTGTCA	ENST00000321117	-65<br>206	-46<br>189	272
DNMT3A-2F<br>DNMT3A-2R	AAGCCAAGGTCATTGCAGGA<br>GTACTCTGGCTCGTCATCGC	ENST00000321117	650<br>852	669<br>833	203
DNMT3A-3F<br>DNMT3A-3R	GGAGAAGCCCAAGGTCAAGG<br>CCGTGATGGAGTCCTCACAC	ENST00000321117	1386<br>2014	1405<br>1995	629
DNMT3A-4F<br>DNMT3A-4R	GACTTGGGCATTCAGGTGGA<br>CCATGCTCCAGACACTCCTG	ENST00000321117	1954<br>2465	1973<br>2446	512
DNMT3A-5F<br>DNMT3A-5R	CGCGATTTCTCGAGTCCAAC<br>CATGTCCCTTACACACACGCA	ENST00000321117	2309<br>2747	2328<br>2727	439
FLT3-1F<br>FLT3-1R	ACAGTGAGTATAAGAAATACCCTGCT<br>TCTTCACTTGAATTGGTAGCATTT	ENST00000241453	469<br>1073	494<br>1050	604
FLT3-2F<br>FLT3-2R	TGCAGAAAATGATGATGCCCA<br>AAGCTGTTGCGTTCATCACTT	ENST00000241453	1257<br>1888	1277<br>1868	632
FLT3-3F<br>FLT3-3R	ACTAGGATCAGGTGCTTTTGGA<br>GAGAAGATCACCATAGCAACAGT	ENST00000241453	1845<br>2100	1866<br>2078	256
FLT3-4F<br>FLT3-4R	TGTGTTCACAGAGACCTGGC<br>AACCGGAATGCCAGGGTAAG	ENST00000241453	2419<br>2682	2438<br>2663	264
FLT3-5F<br>FLT3-2R	TTGGTGTTTGTCTCCTCTTCA<br>AAGCTGTTGCGTTCATCACTT	ENST00000241453	1643<br>1888	1663<br>1868	246
IDH1-1F<br>IDH1-1R	TGGGAGGAACTGGGGTGATA<br>GGCACATTTGACGCCAACAT	ENST00000345146	-56<br>222	-37<br>203	279
IDH1-2F<br>IDH1-2R	GTGGCACGGTCTTCAGAGAA<br>GCCAACCCTTAGACAGAGCC	ENST00000345146	311<br>616	330<br>597	306
IDH2-1F<br>IDH2-1R	GGCCACCCAGAAGTACAGTG<br>GACTTGAGGACCTGAGCCAC	ENST00000330062	306<br>899	325<br>880	594
IDH2-2F<br>IDH2-2R	AGATGCTGGAGAAGGTGTGC<br>ATCCCCTAGAAAGGCCTCCA	ENST00000330062	1187<br>1473	1206<br>1454	287
IDH2-1F<br>IDH2-3R	GGCCACCCAGAAGTACAGTG<br>CCAATGGTGATGGGCTTGGT	ENST00000330062	306<br>512	325<br>493	207
KIT-1F<br>KIT-1R	GCTGCACTTGGGCGAGAG<br>AAAGCCCGGATCAGTGCATA	ENST00000288135	-60<br>189	-43<br>170	250
KIT-2F<br>KIT-2R	CCATCTGTGAGTCCAGGGGA<br>GCTTCTGCCTTTTCCGTGATC	ENST00000288135	79<br>266	98<br>246	188
KIT-3F	ACGAGATTAAAAGGCACCGA	ENST00000288135	1138	1157	235

KIT-3R	CCACTGGCAGTACAGAAGCA		1372	1353	
KIT-4F<br>KIT-4R	ACCGTTTGGAAAGCTAGTGGT<br>TCTCCTCAACAACCTTCCACTG	ENST00000288135	1401<br>1687	1421<br>1666	287
KIT-5F<br>KIT-5R	TCTTACCAGGTGGCAAAGGG<br>TCCCATAGGACCAGACGTCA	ENST00000288135	2317<br>2569	2336<br>2550	253
NRAS-1F<br>NRAS-1R	ACATTTTTCCCGGCTGTGGT<br>AGAGGTTAATATCCGCAAATGACT	ENST00000369535	-73<br>286	-54<br>263	360
NRAS-2F<br>NRAS-2R	CCTCAGCCAAGACCAGACAG<br>AGCTTGAAAGTGGCTCTTTTCTG	ENST00000369535	431<br>615	450<br>593	185
RUNX1-1F<br>RUNX1-1R	CTAGAGACGTCCACGATGCC<br>CAGCTGCTCCAGTTCACTGA	ENST00000300305	83<br>693	102<br>674	611
RUNX1-2F<br>RUNX1-2R	CAGATCCAACCATCCCCACC<br>TCAGTAGGGCCTCCACACG	ENST00000300305	814<br>1443	833<br>1425	630
TET2-1F<br>TET2-1R	ACCAACCATGTTGAGGGCAA<br>AGGCCCACTGCAGTTATGTG	ENST00000380013	16<br>522	35<br>503	507
TET2-2F<br>TET2-2R	ACAAGGCAGTGCTAATGCCT<br>AAATACCGTTCAGAGCTGCCA	ENST00000380013	605<br>1112	624<br>1092	508
TET2-3F<br>TET2-3R	GCCAGTAAACTAGCTGCAATGC<br>GTGGTGTGGTAGTGGCAGAA	ENST00000380013	910<br>1195	931<br>1176	286
TET2-4F<br>TET2-4R	CCCCCAACACAGCACTATCT<br>GGCTCAGTCTCTGAAGCCTG	ENST00000380013	1660<br>2135	1679<br>2116	476
TET2-5F<br>TET2-5R	GTCAGACCATGAAATCAAGTGCA<br>TGCTGTGTTTGCTGCTGTTC	ENST00000380013	2459<br>2900	2481<br>2881	442
TET2-6F<br>TET2-6R	AGTTGAAATGTCAGGGCCAGT<br>GGAGTATCTAGTAATTTGGAAGGTGAC	ENST00000380013	3165<br>3344	3185<br>3318	180
TET2-7F<br>TET2-7R	CGCTGTCTCTGGCTGACAAA<br>GCCTTCCTTCAGACCCAGAC	ENST00000380013	3710<br>4095	3729<br>4076	386
TET2-8F<br>TET2-8R	GTCTGGGTCTGAAGGAAGGC<br>TTGTACGTGATGGGGCTGAC	ENST00000380013	4076<br>4498	4095<br>4479	423
TET2-9F<br>TET2-9R	GCTCAGTCTACCACCCATCC<br>TGGATTGCTCAGATTGGGTGG	ENST00000380013	5007<br>5241	5026<br>5221	235
TET2-10F<br>TET2-10R	GGTGCAGAGGACAACGATGA<br>TGGTCCTTTCGGCAAGAGAC	ENST00000380013	5515<br>5926	5534<br>5907	412
TP53-1F<br>TP53-1R	GCCCCTCCTCAGCATCTTATC<br>ACAAACACGCACCTCAAAGC	ENST00000269305	565<br>825	585<br>806	261
WT1-1F<br>WT1-1R	TGTCGCTACGGGCCCTTC<br>TGGGATCCTCATGCTTGAATGA	ENST00000332351	523<br>748	540<br>727	226
WT1-2F<br>WT1-2R	CACAGCACAGGGTACGAGAG<br>GATGCATGTTGTGATGGCGG	ENST00000332351	1003<br>1519	1022<br>1500	517

[1] PCR was performed for 33 cycles using PWO SuperYield DNA polymerase or Phusion Flash High-Fidelity PCR Master Mix.

Table SIX. Definition of hotspot regions[1].

Gene	ENST transcript	Hotspot	CDS position	Type of mutations
**NPM1**	ENST00000517671	exon 11-12	772-885	4 bp frameshift insertions
**DNMT3A**	ENST00000321117	exon 23	2644-2645	missense SNVs
**FLT3**	ENST00000241453	exon 14-15	1705-1942	3-300 bp in-frame insertions (ITD[2])
		exon 20	2503-2505	missense SNVs (TKD[3])
**ASXL1**	ENST00000375687	exon 13	1720-4626	coding SNV and small indels
**IDH1**	ENST00000345146	exon 4	394-395	missense SNVs
**IDH2**	ENST00000330062	exon 4	374-534	missense SNVs
**KIT**	ENST00000288135	exon 8	1248-1258	in-frame indels
		exon 17	2446-2447	missense SNVs
**NRAS**	ENST00000369535	exon 2	34-38	missense SNVs
		exon 3	181-183	missense SNVs

[1] Hotspot regions are defined by positions in the coding sequence with a mutation frequency according to COSMIC.

[2] ITD; internal tandem duplications

[3] TKD; tyrosine kinase domain

Table SX. Validation of RNAseq-HAMLET by targeted NGS on genomic DNA from 50 selected AML cases[1].

AML	Gene	Chrom	GRCh38[2]	Exon[3]	HGVSc[4]	HGVSp[5]	HS[6]	Cos[7]	SNP[8]	Class[9]	Jaiswal[10]	RNAseq-HAMLET	GenDNA-NGS
1-003	DNMT3A	chr2	25234374	23/23	ENST00000321117.7:c.2644C>T	ENSP00000324375.5:p.Arg882Cys	yes	yes	yes	A	yes	+	+
	TET2	chr4	105261793	8/11	ENST00000380013.6:c.3989C>A	ENSP00000369351.4:p.Ser1330Tyr	no	no	no	C	yes	+	+
1-006	DNMT3A	chr2	25234373	23/23	ENST00000321117.7:c.2645G>A	ENSP00000324375.5:p.Arg882His	yes	yes	yes	A	yes	+	+
1-008	KIT	chr4	54695617	2/21	ENST00000288135.5:c.173G>T	ENSP00000288135.5:p.Cys58Phe	no	no	no	C	no	+	+
	KIT	chr4	54733155	17/21	ENST00000288135.5:c.2447A>T	ENSP00000288135.5:p.Asp816Val	yes	yes	yes	A	yes	+	+
	RUNX1	chr21	34792416	8/8	ENST00000300305.5:c.1161del	ENSP00000300305.3:p.Ser388ArgfsTer206	no	no	yes	D	yes	+	+
2-002	DNMT3A	chr2	25244579	14/23	ENST00000321117.7:c.1628G>T	ENSP00000324375.5:p.Gly543Val	no	yes	no	B	no	+	+
	RUNX1	chr21	34834566	6/8	ENST00000300305.5:c.649G>A	ENSP00000300305.3:p.Gly217Arg	no	no	no	C	no	+	+
	TET2	chr4	105235712	3/11	ENST00000380013.6:c.1771del	ENSP00000369351.4:p.Gln591SerfsTer10	no	no	no	C	yes	+	+
	TET2	chr4	105272773	10/11	ENST00000380013.6:c.4392C>A	ENSP00000369351.4:p.Cys1464Ter	no	yes	no	B	yes	+	+
2-006	TET2	chr4	105237193	3/11	ENST00000380013.6:c.3251A>C	ENSP00000369351.4:p.Gln1084Pro	no	no	yes	D	no	+	+
2-010	TET2	chr4	105275662	11/11	ENST00000380013.6:c.5152G>T	ENSP00000369351.4:p.Val1718Leu	no	yes	yes	B	no	+	+
2-011	IDH1	chr2	208248388	4/10	ENST00000345146.4:c.395G>A	ENSP00000260985.2:p.Arg132His	yes	yes	yes	A	yes	+	+
2-014	KIT	chr4	54658066	1/21	ENST00000288135.5:c.52C>T	ENSP00000288135.5:p.Leu18Phe	no	no	yes	D	no	+	+
2-016	DNMT3A	chr2	25244580	14/23	ENST00000321117.7:c.1627G>T	ENSP00000324375.5:p.Gly543Cys	no	yes	no	B	no	+	+
	TET2	chr4	105234042	3/11	ENST00000380013.6:c.100C>T	ENSP00000369351.4:p.Leu34Phe	no	no	yes	D	no	+	+
2-019	TP53	chr17	7674885	6/11	ENST00000269305.6:c.646G>A	ENSP00000269305.4:p.Val216Met	no	yes	no	B	yes	+	+
2-023	DNMT3A	chr2	25240672	18/23	ENST00000321117.7:c.2141C>G	ENSP00000324375.5:p.Ser714Cys	no	yes	yes	B	yes	+	+
	RUNX1	chr21	34792223	8/8	ENST00000300305.5:c.1342_1354dup	ENSP00000300305.3:p.Val452GlufsTer152	no	no	no	C	yes	+	+
2-024	DNMT3A	chr2	25240666	18/23	ENST00000321117.7:c.2147T>A	ENSP00000324375.5:p.Val716Asp	no	yes	no	B	yes	+	+
	TET2	chr4	105275613	11/11	ENST00000380013.6:c.5103G>A	ENSP00000369351.4:p.Met1701Ile	no	no	yes	D	no	+	+
2-027	IDH1	chr2	208248389	4/10	ENST00000345146.4:c.394C>T	ENSP00000260985.2:p.Arg132Cys	yes	yes	yes	A	yes	+	+
2-028	TET2	chr4	105234350	3/11	ENST00000380013.6:c.409del	ENSP00000369351.4:p.Ser137ValfsTer8	no	no	no	C	yes	+	+
	TET2	chr4	105236721	3/11	ENST00000380013.6:c.2784del	ENSP00000369351.4:p.Pro929LeufsTer24	no	no	no	C	yes	+	+
2-029	DNMT3A	chr2	25234367	23/23	ENST00000321117.7:c.2651C>T	ENSP00000324375.5:p.Ala884Val	no	yes	no	B	yes	+	+
	IDH1	chr2	208245342	5/10	ENST00000345146.4:c.497C>T	ENSP00000260985.2:p.Thr166Ile	no	no	no	C	no	+	+
2-030	DNMT3A	chr2	25243931	16/23	ENST00000321117.7:c.1903C>T	ENSP00000324375.5:p.Arg635Trp	no	yes	yes	B	yes	+	+
2-033	TET2	chr4	105234647	3/11	ENST00000380013.6:c.706del	ENSP00000369351.4:p.Asp236IlefsTer14	no	no	no	C	yes	+	+
	TET2	chr4	105275653	11/11	ENST00000380013.6:c.5145_5151del	ENSP00000369351.4:p.His1716Ter	no	no	no	C	yes	+	+
2-036	DNMT3A	chr2	25234373	23/23	ENST00000321117.7:c.2645G>C	ENSP00000324375.5:p.Arg882Pro	yes	yes	yes	A	yes	+	+
2-037	RUNX1	chr21	34859495	5/8	ENST00000300305.5:c.592G>A	ENSP00000300305.3:p.Asp198Asn	no	yes	no	B	no	+	+
2-038	RUNX1	chr21	34886865	3/8	ENST00000300305.5:c.329A>G	ENSP00000300305.3:p.Lys110Arg	no	yes	no	B	no	-	+ (VAF 0.10)
2-039	RUNX1	chr21	34886865	3/8	ENST00000300305.5:c.329A>G	ENSP00000300305.3:p.Lys110Arg	no	yes	no	B	no	+	+
	IDH1	chr2	208239941	8/10	ENST00000345146.4:c.913G>A	ENSP00000260985.2:p.Ala305Thr	no	no	yes	D	no	-	+ (VAF 0.43)

2-040	DNMT3A	chr2	25234373	23/23	ENST00000321117.7:c.2645G>C	ENSP00000324375.5:p.Arg882Pro	yes	yes	yes	A	yes	+	+
2-041	DNMT3A	chr2	25234374	23/23	ENST00000321117.7:c.2644C>T	ENSP00000324375.5:p.Arg882Cys	yes	yes	yes	A	yes	+	+
	TET2	chr4	105234995	3/11	ENST00000380013.6:c.1055del	ENSP00000369351.4:p.Phe352SerfsTer20	no	no	no	C	yes	+	+
2-048	DNMT3A	chr2	25234373	23/23	ENST00000321117.7:c.2645G>A	ENSP00000324375.5:p.Arg882His	yes	yes	yes	A	yes	+	+
2-049	DNMT3A	chr2	25234373	23/23	ENST00000321117.7:c.2645G>C	ENSP00000324375.5:p.Arg882Pro	yes	yes	yes	A	yes	+	+
2-050	RUNX1	chr21	34886866	3/8	ENST00000300305.5:c.328A>C	ENSP00000300305.3:p.Lys110Gln	no	no	yes	D	no	+	+
	TET2	chr4	105272692	10/11	ENST00000380013.6:c.4317dup	ENSP00000369351.4:p.Arg1440ThrfsTer38	no	no	no	C	yes	+ (VAF 0.39)	-
3-005	DNMT3A	chr2	25234373	23/23	ENST00000321117.7:c.2645G>A	ENSP00000324375.5:p.Arg882His	yes	yes	yes	A	yes	+	+
	TET2	chr4	105234042	3/11	ENST00000380013.6:c.100C>T	ENSP00000369351.4:p.Leu34Phe	no	no	yes	D	no	+	+
	TET2	chr4	105236491	3/11	ENST00000380013.6:c.2549A>G	ENSP00000369351.4:p.His850Arg	no	no	yes	D	no	+	+
3-006	DNMT3A	chr2	25245284	13/23	ENST00000321117.7:c.1522del	ENSP00000324375.5:p.Leu508SerfsTer143	no	no	no	C	yes	+	+
	IDH1	chr2	208243577	6/10	ENST00000345146.4:c.548A>G	ENSP00000260985.2:p.Tyr183Cys	no	no	yes	D	no	+	+
	RUNX1	chr21	34792566	8/8	ENST00000300305.5:c.1011dup	ENSP00000300305.3:p.Ala338ArgfsTer262	no	yes	no	B	yes	+	+
3-007	KIT	chr4	54733155	17/21	ENST00000288135.5:c.2447A>T	ENSP00000288135.5:p.Asp816Val	yes	yes	yes	A	yes	+	+
	TET2	chr4	105272692	10/11	ENST00000380013.6:c.4317dup	ENSP00000369351.4:p.Arg1440ThrfsTer38	no	no	no	C	yes	+ (VAF 0.12)	-
3-009	DNMT3A	chr2	25240313	19/23	ENST00000321117.7:c.2311C>T	ENSP00000324375.5:p.Arg771Ter	no	yes	no	B	yes	+	+
	IDH1	chr2	208248389	4/10	ENST00000345146.4:c.394C>G	ENSP00000260985.2:p.Arg132Gly	yes	yes	yes	A	yes	+	+
3-012	DNMT3A	chr2	25234374	23/23	ENST00000321117.7:c.2644C>A	ENSP00000324375.5:p.Arg882Ser	yes	yes	yes	A	no	+	+
3-013	IDH1	chr2	208243532	6/10	ENST00000345146.4:c.593A>G	ENSP00000260985.2:p.Gln198Arg	no	no	yes	D	no	+	+
	TET2	chr4	105235995	3/11	ENST00000380013.6:c.2053C>T	ENSP00000369351.4:p.Gln685Ter	no	no	no	C	yes	+	+
3-015	TP53	chr17	7674220	7/11	ENST00000269305.6:c.743G>A	ENSP00000269305.4:p.Arg248Gln	no	yes	yes	B	yes	+	+
	TET2	chr4	105272591	10/11	ENST00000380013.6:c.4210C>T	ENSP00000369351.4:p.Arg1404Ter	no	yes	no	B	yes	+	+
3-017	DNMT3A	chr2	25234373	23/23	ENST00000321117.7:c.2645G>A	ENSP00000324375.5:p.Arg882His	yes	yes	yes	A	yes	+	+
	IDH1	chr2	208251458	3/10	ENST00000345146.4:c.94T>G	ENSP00000260985.2:p.Phe32Val	no	yes	yes	B	no	+	+
	RUNX1	chr21	34792448	8/8	ENST00000300305.5:c.1129dup	ENSP00000300305.3:p.Tyr377LeufsTer223	no	no	no	C	yes	+	+
	TET2	chr4	105234763	3/11	ENST00000380013.6:c.822del	ENSP00000369351.4:p.Asn275IlefsTer18	no	yes	no	B	Yes	+	+
	TET2	chr4	105259626	7/11	ENST00000380013.6:c.3812dup	ENSP00000369351.4:p.Cys1271TrpfsTer29	no	yes	no	B	yes	+	+
3-023	TET2	chr4	105261769	8/11	ENST00000380013.6:c.3965T>G	ENSP00000369351.4:p.Leu1322Arg	no	yes	no	B	yes	+	+
3-024	TP53	chr17	7674259	7/11	ENST00000269305.6:c.704A>G	ENSP00000269305.4:p.Asn235Ser	no	yes	yes	B	no	+	+
3-034	IDH1	chr2	208248389	4/10	ENST00000345146.4:c.394C>T	ENSP00000260985.2:p.Arg132Cys	yes	yes	yes	A	yes	+	+
	TET2	chr4	105275662	11/11	ENST00000380013.6:c.5152G>T	ENSP00000369351.4:p.Val1718Leu	no	yes	yes	B	no	+	+
3-038	DNMT3A	chr2	25239194	20/23	ENST00000321117.7:c.2340_2343del	ENSP00000324375.5:p.Ile780MetfsTer21	no	no	no	C	yes	+	+
3-042	TET2	chr4	105236554	3/11	ENST00000380013.6:c.2613dup	ENSP00000369351.4:p.Val872CysfsTer29	no	no	no	C	yes	+	+
	TET2	chr4	105269725	9/11	ENST00000380013.6:c.4160A>G	ENSP00000369351.4:p.Asn1387Ser	no	no	no	C	yes	+	+
	TET2	chr4	105272692	10/11	ENST00000380013.6:c.4317dup	ENSP00000369351.4:p.Arg1440ThrfsTer38	no	no	no	C	yes	+ (VAF 0.13)	-

[1] Targeted NGS has been performed on genomic DNA from 50 AML cases for protein coding regions of *RUNX1* (8 exons), *TP53* (11 exons), *TET2* (11 exons), *KIT1* (21 exons) and *IDH1* (8 exons) as well as a large part of the protein coding region of *DNMT3A* (exons 13-23). Results are shown for AML

cases in which variants were detected by HAMLET or targeted NGS. AML cases in which no variants were detected by both HAMLET and targeted NGS are not shown. *DNMT3A* variants that were detected by HAMLET outside exons 13-23 are not covered by targeted NGS and excluded from the analysis. In addition, a limited number of *RUNX1* variants that were detected by HAMLET in 20-30 bp regions between amplicons that were not covered by targeted NGS were excluded. Variants that were detected by HAMLET or targeted NGS are shown in blue (+). Variants that were not detected by HAMLET or targeted NGS are shown in orange (-). Variants indicated in yellow are nonsense variants or frameshift variants with a premature stop codon that are potential targets for nonsense mediated decay.

[2] Position of the variant in the GRCh38 human genome.

[3] Exon location of the variant / total number of exons of the gene.

[4] HGVSc; coding sequence name as identified by the Human Genome Variation Society (http://www.hgvs.org/).

[5] HGVSp; protein sequence name as identified by the Human Genome Variation Society (http://www.hgvs.org/).

[6] HS; indicated whether the variant occurs in a hotspot (HS) region as identified in Table SVI.

[7] COS; indicated whether the variant is known as cancer mutation in COSMIC (catalogue of somatic mutations in cancer; http://cancer.sanger.ac.uk/cosmic).

[8] SNP; indicated whether the variant is known as single nucleotide polymorphism in dbSNP (https://www.ncbi.nlm.nih.gov/projects/SNP).

[9] Class; A, hotspot variant known in COSMIC (known or unknown as SNP); B, variant not in a hotspot but known in COSMIC (known or unknown as SNP); C, variant unknown in COSMIC and unknown as SNP; D, variant unknown in COSMIC and known as SNP.

[10] Jaiswal; indicated whether the variant is a recurrent mutation in myeloid malignancies by Jaiswal *et al.* (NEJM 2017).

Table SXI. Sensitivity of HAMLET to detect small variants at different sequencing depths[1].

AML	Gene	Chrom	GRCh38[2]	Exon[3]	HGVSc[4]	HGVSp[5]	HS[6]	Cos[7]	SNP[8]	Class[9]	Jaiswal[10]	20M	30M	40M	47.5M
1-001	NRAS	chr1	114716123	2/7	ENST00000369535.4:c.38G>A	ENSP00000358548.4:p.Gly13Asp	yes	yes	no	A	yes	+	n.d.	n.d.	n.d.
1-002	DNMT3A	chr2	25234373	23/23	ENST00000321117.7:c.2645G>A	ENSP00000324375.5:p.Arg882His	yes	yes	yes	A	yes	+	n.d.	n.d.	n.d.
	FLT3	chr13	28018505	20/24	ENST00000241453.9:c.2503G>C	ENSP00000241453.7:p.Asp835His	yes	yes	no	A	yes	+	n.d.	n.d.	n.d.
	FLT3	chr13	28052579	5/24	ENST00000241453.9:c.580G>A	ENSP00000241453.7:p.Val194Met	no	yes	yes	B	no	+	n.d.	n.d.	n.d.
	IDH1	chr2	208243577	6/10	ENST00000345146.4:c.548A>G	ENSP00000260985.2:p.Tyr183Cys	no	no	yes	D	no	+	n.d.	n.d.	n.d.
	TET2	chr4	105236781	3/11	ENST00000380013.6:c.2839C>T	ENSP00000369351.4:p.Gln947Ter	no	yes	no	B	yes	-	-	-	+
	TET2	chr4	105276128	11/11	ENST00000380013.6:c.5618T>C	ENSP00000369351.4:p.Ile1873Thr	no	yes	yes	B	yes	+	n.d.	n.d.	n.d.
	NPM1	chr5	171410539	12/12	ENST00000517671.3:c.860_863dup	ENSP00000428755.1:p.Trp288CysfsTer12	yes	yes	no	A	yes	+	n.d.	n.d.	n.d.
1-003	DNMT3A	chr2	25234374	23/23	ENST00000321117.7:c.2644C>T	ENSP00000324375.5:p.Arg882Cys	yes	yes	yes	A	yes	+	n.d.	n.d.	n.d.
	TET2	chr4	105261793	8/11	ENST00000380013.6:c.3989C>A	ENSP00000369351.4:p.Ser1330Tyr	no	no	no	C	yes	+	n.d.	n.d.	n.d.
	NPM1	chr5	171410540	12/12	ENST00000517671.3:c.863_864insCCTG	ENSP00000428755.1:p.Trp288CysfsTer12	yes	yes	no	A	yes	+	n.d.	n.d.	n.d.
1-004	KIT	chr4	54733155	17/21	ENST00000288135.5:c.2447A>T	ENSP00000288135.5:p.Asp816Val	yes	yes	yes	A	yes	+	n.d.	n.d.	n.d.
1-005	IDH2	chr15	90084321	11/11	ENST00000330062.5:c.1304C>T	ENSP00000331897.3:p.Thr435Met	no	no	yes	D	no	+	n.d.	n.d.	n.d.
1-006	DNMT3A	chr2	25234373	23/23	ENST00000321117.7:c.2645G>A	ENSP00000324375.5:p.Arg882His	yes	yes	yes	A	yes	+	n.d.	n.d.	n.d.
1-008	KIT	chr4	54695617	2/21	ENST00000288135.5:c.173G>T	ENSP00000288135.5:p.Cys58Phe	no	no	no	C	no	+	n.d.	n.d.	n.d.
	KIT	chr4	54733155	17/21	ENST00000288135.5:c.2447A>T	ENSP00000288135.5:p.Asp816Val	yes	yes	yes	A	yes	+	n.d.	n.d.	n.d.
	RUNX1	chr21	34792416	8/8	ENST00000300305.5:c.1161del	ENSP00000300305.3:p.Ser388ArgfsTer206	no	no	yes	D	yes	+	n.d.	n.d.	n.d.
	RUNX1	chr21	34887027	3/8	ENST00000300305.5:c.167T>C	ENSP00000300305.3:p.Leu56Ser	no	yes	yes	B	no	+	n.d.	n.d.	n.d.
	ASXL1	chr20	32434638	13/13	ENST00000375687.6:c.1934dup	ENSP00000364839.4:p.Gly646TrpfsTer12	yes	yes	no	A	yes	+	n.d.	n.d.	n.d.
2-001	DNMT3A	chr2	25234374	23/23	ENST00000321117.7:c.2644C>T	ENSP00000324375.5:p.Arg882Cys	yes	yes	yes	A	yes	+	n.d.	n.d.	n.d.
	NPM1	chr5	171410539	12/12	ENST00000517671.3:c.860_863dup	ENSP00000428755.1:p.Trp288CysfsTer12	yes	yes	no	A	yes	+	n.d.	n.d.	n.d.
2-002	DNMT3A	chr2	25244579	14/23	ENST00000321117.7:c.1628G>T	ENSP00000324375.5:p.Gly543Val	no	yes	no	B	no	+	n.d.	n.d.	n.d.
	RUNX1	chr21	34834566	6/8	ENST00000300305.5:c.649G>A	ENSP00000300305.3:p.Gly217Arg	no	no	no	C	no	+	n.d.	n.d.	n.d.
	TET2	chr4	105235712	3/11	ENST00000380013.6:c.1771del	ENSP00000369351.4:p.Gln591SerfsTer10	no	no	no	C	yes	+	n.d.	n.d.	n.d.
	TET2	chr4	105272773	10/11	ENST00000380013.6:c.4392C>A	ENSP00000369351.4:p.Cys1464Ter	no	yes	no	B	yes	+	n.d.	n.d.	n.d.
2-003	FLT3	chr13	28018505	20/24	ENST00000241453.9:c.2503G>C	ENSP00000241453.7:p.Asp835His	yes	yes	no	A	yes	+	n.d.	n.d.	n.d.
	FLT3	chr13	28049450	8/24	ENST00000241453.9:c.970G>A	ENSP00000241453.7:p.Asp324Asn	no	yes	yes	B	no	+	n.d.	n.d.	n.d.
2-004	KIT	chr4	54733154	17/21	ENST00000288135.5:c.2446G>T	ENSP00000288135.5:p.Asp816Tyr	yes	yes	yes	A	yes	+	n.d.	n.d.	n.d.
	RUNX1	chr21	34887027	3/8	ENST00000300305.5:c.167T>C	ENSP00000300305.3:p.Leu56Ser	no	yes	yes	B	no	+	n.d.	n.d.	n.d.
	ASXL1	chr20	32436457	13/13	ENST00000375687.6:c.3745A>G	ENSP00000364839.4:p.Met1249Val	yes	no	yes	D	no	+	n.d.	n.d.	n.d.
	WT1	chr11	32396363	7/10	ENST00000332351.5:c.1133_1142dup	ENSP00000331327.3:p.Ala382CysfsTer6	no	yes	no	B	yes	-	-	-	+
2-005	IDH2	chr15	90087472	6/11	ENST00000330062.5:c.782G>A	ENSP00000331897.3:p.Arg261His	no	no	yes	C	no	+	n.d.	n.d.	n.d.
2-006	FLT3	chr13	28034118	14/24	ENST00000241453.9: c.1780_1800dup	ENSP00000241453.7:p.Phe594_Asp600dup	yes	yes	no	A	no	+	n.d.	n.d.	n.d.
	TET2	chr4	105237193	3/11	ENST00000380013.6:c.3251A>C	ENSP00000369351.4:p.Gln1084Pro	no	no	yes	D	no	-	+	n.d.	n.d.

2-007	KIT	chr4	54726036	9/21	ENST00000288135.5:c.1526A>T	ENSP00000288135.5:p.Lys509Ile	no	yes	no	B	no	+	n.d.	n.d.	n.d.
	TET2	chr4	105259708	7/11	ENST00000380013.6:c.3893G>A	ENSP00000369351.4:p.Cys1298Tyr	no	yes	no	B	yes	+	n.d.	n.d.	n.d.
	CEBPA	chr19	33301447	1/1	ENST00000498907.2:c.968G>T	ENSP00000427514.1:p.Arg323Leu	no	yes	no	B	no	+	n.d.	n.d.	n.d.
2-008	FLT3	chr13	28018503	20/24	ENST00000241453.9:c.2505T>G	ENSP00000241453.7:p.Asp835Glu	yes	yes	yes	A	yes	+	n.d.	n.d.	n.d.
	IDH1	chr2	208243577	6/10	ENST00000345146.4:c.548A>G	ENSP00000260985.2:p.Tyr183Cys	no	no	yes	D	no	+	n.d.	n.d.	n.d.
	RUNX1	chr21	34799384	7/8	ENST00000300305.5:c.883dup	ENSP00000300305.3:p.Ser295PhefsTer305	no	no	no	C	yes	+	n.d.	n.d.	n.d.
2-009	FLT3	chr13	28052579	5/24	ENST00000241453.9:c.580G>A	ENSP00000241453.7:p.Val194Met	no	yes	yes	B	no	+	n.d.	n.d.	n.d.
	TET2	chr4	105237193	3/11	ENST00000380013.6:c.3251A>C	ENSP00000369351.4:p.Gln1084Pro	no	no	yes	D	no	-	+	n.d.	n.d.
	CEBPA	chr19	33301478	1/1	ENST00000498907.2:c.934_936dup	ENSP00000427514.1:p.Gln312dup	no	yes	no	B	no	+	n.d.	n.d.	n.d.
	CEBPA	chr19	33302075	1/1	ENST00000498907.2:c.332_339del	ENSP00000427514.1:p.Ala111GlyfsTer56	no	yes	no	B	yes	+	n.d.	n.d.	n.d.
2-010	TET2	chr4	105275662	11/11	ENST00000380013.6:c.5152G>T	ENSP00000369351.4:p.Val1718Leu	no	yes	yes	B	no	+	n.d.	n.d.	n.d.
	NRAS	chr1	114716127	2/7	ENST00000369535.4:c.34G>T	ENSP00000358548.4:p.Gly12Cys	yes	yes	no	A	yes	+	n.d.	n.d.	n.d.
2-011	FLT3	chr13	28018502	20/24	ENST00000241453.9:c.2503_2505del	ENSP00000241453.7:p.Asp835del	yes	yes	yes	A	yes	+	n.d.	n.d.	n.d.
	FLT3	chr13	28018505	20/24	ENST00000241453.9:c.2503G>T	ENSP00000241453.7:p.Asp835Tyr	yes	yes	no	A	yes	+	n.d.	n.d.	n.d.
	IDH1	chr2	208248388	4/10	ENST00000345146.4:c.395G>A	ENSP00000260985.2:p.Arg132His	yes	yes	yes	A	yes	+	n.d.	n.d.	n.d.
	NPM1	chr5	171410539	12/12	ENST00000517671.3:c.860_863dup	ENSP00000428755.1:p.Trp288CysfsTer12	yes	yes	no	A	yes	+	n.d.	n.d.	n.d.
2-012	NPM1	chr5	171410541	12/12	ENST00000517671.3:c.863_864insCTTG	ENSP00000428755.1:p.Trp288CysfsTer12	yes	yes	no	A	yes	+	n.d.	n.d.	n.d.
2-013	RUNX1	chr21	34880640	4/8	ENST00000300305.5:c.423_424dup	ENSP00000300305.3:p.Ala142GlyfsTer4	no	yes	no	B	yes	+	n.d.	n.d.	n.d.
2-014	KIT	chr4	54658066	1/21	ENST00000288135.5:c.52C>T	ENSP00000288135.5:p.Leu18Phe	no	no	yes	D	no	+	n.d.	n.d.	n.d.
	RUNX1	chr21	34887027	3/8	ENST00000300305.5:c.167T>C	ENSP00000300305.3:p.Leu56Ser	no	yes	yes	B	no	+	n.d.	n.d.	n.d.
	CEBPA	chr19	33301825	1/1	ENST00000498907.2:c.584_589dup	ENSP00000427514.1:p.His195_Pro196dup	no	no	no	C	no	-	+	n.d.	n.d.
2-015	DNMT3A	chr2	25244603	14/23	ENST00000321117.7:c.1583_1603dup	ENSP00000324375.5:p.Tyr528_Gln534dup	no	no	no	C	no	+	n.d.	n.d.	n.d.
	TET2	chr4	105272692	10/11	ENST00000380013.6:c.4317dup	ENSP00000369351.4:p.Arg1440ThrfsTer38	no	no	no	C	yes	-	+	n.d.	n.d.
	NPM1	chr5	171410539	12/12	ENST00000517671.3:c.860_863dup	ENSP00000428755.1:p.Trp288CysfsTer12	yes	yes	no	A	yes	+	n.d.	n.d.	n.d.
2-016	DNMT3A	chr2	25244580	14/23	ENST00000321117.7:c.1627G>T	ENSP00000324375.5:p.Gly543Cys	no	yes	no	B	no	+	n.d.	n.d.	n.d.
	TET2	chr4	105234042	3/11	ENST00000380013.6:c.100C>T	ENSP00000369351.4:p.Leu34Phe	no	no	yes	D	no	+	n.d.	n.d.	n.d.
	NPM1	chr5	171410539	12/12	ENST00000517671.3:c.860_863dup	ENSP00000428755.1:p.Trp288CysfsTer12	yes	yes	no	A	yes	+	n.d.	n.d.	n.d.
	WT1	chr11	32391978	9/10	ENST00000332351.5:c.1426A>G	ENSP00000331327.3:p.Lys476Glu	no	no	no	C	no	-	+	n.d.	n.d.
2-018	FLT3	chr13	28034114	14/24	ENST00000241453.9:c.1781_1804dup	ENSP00000241453.7:p.Leu601_Lys602insIleArgGluTyrGluTyrAspLeu	yes	yes	no	A	no	+	n.d.	n.d.	n.d.
	NPM1	chr5	171410539	12/12	ENST00000517671.3:c.860_863dup	ENSP00000428755.1:p.Trp288CysfsTer12	yes	yes	no	A	yes	+	n.d.	n.d.	n.d.
2-019	TP53	chr17	7674885	6/11	ENST00000269305.6:c.646G>A	ENSP00000269305.4:p.Val216Met	no	yes	no	B	yes	+	n.d.	n.d.	n.d.
2-020	ASXL1	chr20	32434789	13/13	ENST00000375687.6:c.2077C>T	ENSP00000364839.4:p.Arg693Ter	yes	yes	yes	A	yes	+	n.d.	n.d.	n.d.
2-021	RUNX1	chr21	34886877	3/8	ENST00000300305.5:c.317G>C	ENSP00000300305.3:p.Trp106Ser	no	no	no	C	no	+	n.d.	n.d.	n.d.
	ASXL1	chr20	32434599	13/13	ENST00000375687.6:c.1900_1922del	ENSP00000364839.4:p.Glu635ArgfsTer15	yes	yes	no	A	yes	-	-	+	n.d.
2-022	FLT3	chr13	28036022	11/24	ENST00000241453.9:c.1331A>T	ENSP00000241453.7:p.Glu444Val	no	no	no	C	no	+	n.d.	n.d.	n.d.
2-023	DNMT3A	chr2	25240672	18/23	ENST00000321117.7:c.2141C>G	ENSP00000324375.5:p.Ser714Cys	no	yes	yes	B	yes	+	n.d.	n.d.	n.d.
	RUNX1	chr21	34792223	8/8	ENST00000300305.5:c.1342_1354dup	ENSP00000300305.3:p.Val452GlufsTer152	no	no	no	C	yes	+	n.d.	n.d.	n.d.

	NPM1	chr5	171410539	12/12	ENST00000517671.3:c.860_863dup	ENSP00000428755.1:p.Trp288CysfsTer12	yes	yes	no	A	yes	+	n.d.	n.d.	n.d.
2-024	DNMT3A	chr2	25240666	18/23	ENST00000321117.7:c.2147T>A	ENSP00000324375.5:p.Val716Asp	no	yes	no	B	yes	+	n.d.	n.d.	n.d.
	RUNX1	chr21	34886871	3/8	ENST00000300305.5:c.323G>C	ENSP00000300305.3:p.Cys108Ser	no	no	no	C	no	+	n.d.	n.d.	n.d.
		chr21	34886872	3/8	ENST00000300305.5:c.322T>A	ENSP00000300305.3:p.Cys108Ser	no	no	no	C	no	+	n.d.	n.d.	n.d.
		chr21	34886874	3/8	ENST00000300305.5:c.320G>A	ENSP00000300305.3:p.Arg107His	no	yes	no	B	no	+	n.d.	n.d.	n.d.
	TET2	chr4	105275613	11/11	ENST00000380013.6:c.5103G>A	ENSP00000369351.4:p.Met1701Ile	no	no	yes	D	no	+	n.d.	n.d.	n.d.
2-025	NRAS	chr1	114713908	3/7	ENST00000369535.4:c.182A>G	ENSP00000358548.4:p.Gln61Arg	yes	yes	no	A	yes	+	n.d.	n.d.	n.d.
	CEBPA	chr19	33302154	1/1	ENST00000498907.2:c.260del	ENSP00000427514.1:p.Gln87ArgfsTer73	no	no	no	C	yes	+	n.d.	n.d.	n.d.
2-027	IDH1	chr2	208248389	4/10	ENST00000345146.4:c.394C>T	ENSP00000260985.2:p.Arg132Cys	yes	yes	yes	A	yes	+	n.d.	n.d.	n.d.
	ASXL1	chr20	32434638	13/13	ENST00000375687.6:c.1934dup	ENSP00000364839.4:p.Gly646TrpfsTer12	yes	yes	no	A	yes	-	+	n.d.	n.d.
	NRAS	chr1	114713909	3/7	ENST00000369535.4:c.181C>A	ENSP00000358548.4:p.Gln61Lys	yes	yes	yes	A	yes	+	n.d.	n.d.	n.d.
2-028	DNMT3A	chr2	25248139	7/23	ENST00000321117.7:c.752dup	ENSP00000324375.5:p.Asp252Ter	no	no	no	C	yes	+	n.d.	n.d.	n.d.
	TET2	chr4	105234350	3/11	ENST00000380013.6:c.409del	ENSP00000369351.4:p.Ser137ValfsTer8	no	no	no	C	yes	+	n.d.	n.d.	n.d.
	TET2	chr4	105236721	3/11	ENST00000380013.6:c.2784del	ENSP00000369351.4:p.Pro929LeufsTer24	no	no	no	C	yes	+	n.d.	n.d.	n.d.
	NPM1	chr5	171410539	12/12	ENST00000517671.3:c.860_863dup	ENSP00000428755.1:p.Trp288CysfsTer12	yes	yes	no	A	yes	+	n.d.	n.d.	n.d.
2-029	DNMT3A	chr2	25234367	23/23	ENST00000321117.7:c.2651C>T	ENSP00000324375.5:p.Ala884Val	no	yes	no	B	yes	+	n.d.	n.d.	n.d.
	IDH1	chr2	208245342	5/10	ENST00000345146.4:c.497C>T	ENSP00000260985.2:p.Thr166Ile	no	no	no	C	no	+	n.d.	n.d.	n.d.
	NPM1	chr5	171410539	12/12	ENST00000517671.3:c.860_863dup	ENSP00000428755.1:p.Trp288CysfsTer12	yes	yes	no	A	yes	+	n.d.	n.d.	n.d.
2-030	DNMT3A	chr2	25243931	16/23	ENST00000321117.7:c.1903C>T	ENSP00000324375.5:p.Arg635Trp	no	yes	yes	B	yes	+	n.d.	n.d.	n.d.
	NPM1	chr5	171410539	12/12	ENST00000517671.3:c.860_863dup	ENSP00000428755.1:p.Trp288CysfsTer12	yes	yes	no	A	yes	+	n.d.	n.d.	n.d.
	IDH2	chr15	90088702	4/11	ENST00000330062.5:c.419G>A	ENSP00000331897.3:p.Arg140Gln	yes	yes	yes	A	yes	+	n.d.	n.d.	n.d.
	WT1	chr11	32396358	7/10	ENST00000332351.5:c.1143_1147dup	ENSP00000331327.3:p.Ser383TrpfsTer68	no	no	no	C	yes	+	n.d.	n.d.	n.d.
2-032	FLT3	chr13	28034125	14/24	ENST00000241453.9:c.1793_1794insGCCCTTCAGAGAATATGA	ENSP00000241453.7:p.Glu598_Tyr599insProPheArgGluTyrGlu	yes	yes	no	A	no	+	n.d.	n.d.	n.d.
	WT1	chr11	32396365	7/10	ENST00000332351.5:c.1140dup	ENSP00000331327.3:p.Ser381ValfsTer4	no	yes	no	B	yes	-	+	n.d.	n.d.
		chr11	32396368	7/10	ENST00000332351.5:c.1138C>G	ENSP00000331327.3:p.Arg380Gly	no	yes	no	B	no	-	+	n.d.	n.d.
2-033	FLT3	chr13	28034116	14/24	ENST00000241453.9:c.1779_1802dup	ENSP00000241453.7:p.Phe594_Leu601dup	yes	no	no	A	no	+	n.d.	n.d.	n.d.
	TET2	chr4	105234647	3/11	ENST00000380013.6:c.706del	ENSP00000369351.4:p.Asp236IlefsTer14	no	no	no	C	yes	+	n.d.	n.d.	n.d.
	TET2	chr4	105275653	11/11	ENST00000380013.6:c.5145_5151del	ENSP00000369351.4:p.His1716Ter	no	no	no	C	yes	+	n.d.	n.d.	n.d.
	NPM1	chr5	171410539	12/12	ENST00000517671.3:c.860_863dup	ENSP00000428755.1:p.Trp288CysfsTer12	yes	yes	no	A	yes	+	n.d.	n.d.	n.d.
2-034	FLT3	chr13	28034345	13/24	ENST00000241453.9:c.1660T>A	ENSP00000241453.7:p.Phe554Ile	no	no	no	C	no	+	n.d.	n.d.	n.d.
	FLT3	chr13	28034347	13/24	ENST00000241453.9:c.1658T>C	ENSP00000241453.7:p.Leu553Pro	no	no	no	C	no	+	n.d.	n.d.	n.d.
	TET2	chr4	105275613	11/11	ENST00000380013.6:c.5103G>A	ENSP00000369351.4:p.Met1701Ile	no	no	yes	D	no	+	n.d.	n.d.	n.d.
	NPM1	chr5	171410539	12/12	ENST00000517671.3:c.860_863dup	ENSP00000428755.1:p.Trp288CysfsTer12	yes	yes	no	A	yes	+	n.d.	n.d.	n.d.
2-035	FLT3	chr13	28034125	14/24	ENST00000241453.9:c.1776_1793dup	ENSP00000241453.7:p.Tyr597_Glu598insAspAspPheArgGluTyr	yes	yes	no	A	no	+	n.d.	n.d.	n.d.
	ASXL1	chr20	32436018	13/13	ENST00000375687.6:c.3306G>T	ENSP00000364839.4:p.Glu1102Asp	yes	yes	yes	A	no	+	n.d.	n.d.	n.d.
	NPM1	chr5	171410539	12/12	ENST00000517671.3:c.860_863dup	ENSP00000428755.1:p.Trp288CysfsTer12	yes	yes	no	A	yes	+	n.d.	n.d.	n.d.
2-036	DNMT3A	chr2	25234373	23/23	ENST00000321117.7:c.2645G>C	ENSP00000324375.5:p.Arg882Pro	yes	yes	yes	A	yes	+	n.d.	n.d.	n.d.

	RUNX1	chr21	34887027	3/8	ENST00000300305.5:c.167T>C	ENSP00000300305.3:p.Leu56Ser	no	yes	yes	B	no	+	n.d.	n.d.	n.d.
	CEBPA	chr19	33302075	1/1	ENST00000498907.2:c.332_339del	ENSP00000427514.1:p.Ala111GlyfsTer56	no	yes	no	B	yes	+	n.d.	n.d.	n.d.
2-037	RUNX1	chr21	34859495	5/8	ENST00000300305.5:c.592G>A	ENSP00000300305.3:p.Asp198Asn	no	yes	no	B	no	+	n.d.	n.d.	n.d.
	ASXL1	chr20	32434638	13/13	ENST00000375687.6:c.1934dup	ENSP00000364839.4:p.Gly646TrpfsTer12	yes	yes	no	A	yes	-	-	+	n.d.
2-038	NRAS	chr1	114713908	3/7	ENST00000369535.4:c.182A>G	ENSP00000358548.4:p.Gln61Arg	yes	yes	no	A	yes	+	n.d.	n.d.	n.d.
		chr1	114713909	3/7	ENST00000369535.4:c.181C>A	ENSP00000358548.4:p.Gln61Lys	yes	yes	yes	A	yes	+	n.d.	n.d.	n.d.
	NRAS	chr1	114716126	2/7	ENST00000369535.4:c.35G>C	ENSP00000358548.4:p.Gly12Ala	yes	yes	no	A	yes	+	n.d.	n.d.	n.d.
2-039	RUNX1	chr21	34886865	3/8	ENST00000300305.5:c.329A>G	ENSP00000300305.3:p.Lys110Arg	no	yes	no	B	no	+	n.d.	n.d.	n.d.
	CEBPA	chr19	33301475	1/1	ENST00000498907.2:c.937_939dup	ENSP00000427514.1:p.Lys313dup	no	yes	no	B	no	+	n.d.	n.d.	n.d.
	CEBPA	chr19	33302060	1/1	ENST00000498907.2:c.354del	ENSP00000427514.1:p.Val119SerfsTer41	no	no	no	C	yes	+	n.d.	n.d.	n.d.
2-040	DNMT3A	chr2	25234373	23/23	ENST00000321117.7:c.2645G>C	ENSP00000324375.5:p.Arg882Pro	yes	yes	yes	A	yes	+	n.d.	n.d.	n.d.
	RUNX1	chr21	34887027	3/8	ENST00000300305.5:c.167T>C	ENSP00000300305.3:p.Leu56Ser	no	yes	yes	B	no	+	n.d.	n.d.	n.d.
	WT1	chr11	32396399	7/10	ENST00000332351.5:c.1106dup	ENSP00000331327.3:p.Arg370ThrfsTer15	no	yes	no	B	yes	+	n.d.	n.d.	n.d.
		chr11	32396401	7/10	ENST00000332351.5:c.1105C>G	ENSP00000331327.3:p.Arg369Gly	no	yes	no	B	no	+	n.d.	n.d.	n.d.
2-041	DNMT3A	chr2	25234374	23/23	ENST00000321117.7:c.2644C>T	ENSP00000324375.5:p.Arg882Cys	yes	yes	yes	A	yes	+	n.d.	n.d.	n.d.
	TET2	chr4	105234995	3/11	ENST00000380013.6:c.1054del	ENSP00000369351.4:p.Phe352SerfsTer20	no	no	no	C	yes	+	n.d.	n.d.	n.d.
	NPM1	chr5	171410539	12/12	ENST00000517671.3:c.860_863dup	ENSP00000428755.1:p.Trp288CysfsTer12	yes	yes	no	A	yes	+	n.d.	n.d.	n.d.
2-044	FLT3	chr13	28049450	8/24	ENST00000241453.9:c.970G>A	ENSP00000241453.7:p.Asp324Asn	no	yes	yes	B	no	+	n.d.	n.d.	n.d.
	KIT	chr4	54723599	8/21	ENST00000288135.5:c.1248_1249del	ENSP00000288135.5:p.Thr417LeufsTer30	yes	no	no	C	no	-	-	+	n.d.
		chr4	54723605	8/21	ENST00000288135.5:c.1254_1255insCTTCTT	ENSP00000288135.5:p.Tyr418_Asp419insPhePhe	yes	yes	no	A	no	+	n.d.	n.d.	n.d.
		chr4	54723606	8/21	ENST00000288135.5:c.1255_1258del	ENSP00000288135.5:p.Asp419GlyfsTer3	yes	yes	no	A	no	-	-	-	+
2-045	WT1	chr11	32392014	9/10	ENST00000332351.5:c.1390G>C	ENSP00000331327.3:p.Asp464His	no	yes	yes	B	no	+	n.d.	n.d.	n.d.
	NRAS	chr1	114713907	3/7	ENST00000369535.4:c.183A>T	ENSP00000358548.4:p.Gln61His	yes	yes	yes	A	yes	+	n.d.	n.d.	n.d.
	CEBPA	chr19	33301475	1/1	ENST00000498907.2:c.937_939dup	ENSP00000427514.1:p.Lys313dup	no	yes	no	B	no	+	n.d.	n.d.	n.d.
	CEBPA	chr19	33302118	1/1	ENST00000498907.2:c.296dup	ENSP00000427514.1:p.Gly100ArgfsTer8	no	no	no	C	yes	+	n.d.	n.d.	n.d.
2-046	DNMT3A	chr2	25234373	23/23	ENST00000321117.7:c.2645G>A	ENSP00000324375.5:p.Arg882His	yes	yes	yes	A	yes	+	n.d.	n.d.	n.d.
	ASXL1	chr20	32436018	13/13	ENST00000375687.6:c.3306G>T	ENSP00000364839.4:p.Glu1102Asp	yes	yes	yes	A	no	+	n.d.	n.d.	n.d.
	NPM1	chr5	171410539	12/12	ENST00000517671.3:c.860_863dup	ENSP00000428755.1:p.Trp288CysfsTer12	yes	yes	no	A	yes	+	n.d.	n.d.	n.d.
2-048	DNMT3A	chr2	25234373	23/23	ENST00000321117.7:c.2645G>A	ENSP00000324375.5:p.Arg882His	yes	yes	yes	A	yes	+	n.d.	n.d.	n.d.
	FLT3	chr13	28018503	20/24	ENST00000241453.9:c.2505T>G	ENSP00000241453.7:p.Asp835Glu	yes	yes	yes	A	yes	+	n.d.	n.d.	n.d.
	NPM1	chr5	171410539	12/12	ENST00000517671.3:c.860_863dup	ENSP00000428755.1:p.Trp288CysfsTer12	yes	yes	no	A	yes	+	n.d.	n.d.	n.d.
	CEBPA	chr19	33301484	1/1	ENST00000498907.2:c.930dup	ENSP00000427514.1:p.Gln311AlafsTer10	no	yes	no	B	yes	+	n.d.	n.d.	n.d.
2-049	DNMT3A	chr2	25234373	23/23	ENST00000321117.7:c.2645G>C	ENSP00000324375.5:p.Arg882Pro	yes	yes	yes	A	yes	+	n.d.	n.d.	n.d.
	RUNX1	chr21	34887027	3/8	ENST00000300305.5:c.167T>C	ENSP00000300305.3:p.Leu56Ser	no	yes	yes	B	no	+	n.d.	n.d.	n.d.
	NPM1	chr5	171410539	12/12	ENST00000517671.3:c.860_863dup	ENSP00000428755.1:p.Trp288CysfsTer12	yes	yes	no	A	yes	+	n.d.	n.d.	n.d.
	IDH2	chr15	90088702	4/11	ENST00000330062.5:c.419G>A	ENSP00000331897.3:p.Arg140Gln	yes	yes	yes	A	yes	+	n.d.	n.d.	n.d.
	NRAS	chr1	114716126	2/7	ENST00000369535.4:c.35G>A	ENSP00000358548.4:p.Gly12Asp	yes	yes	no	A	yes	+	n.d.	n.d.	n.d.

2-050	FLT3	chr13	28015643	21/24	ENST00000241453.9:c.2600T>C	ENSP00000241453.7:p.Ile867Thr	no	no	no	C	no	+	n.d.	n.d.	n.d.
	RUNX1	chr21	34886866	3/8	ENST00000300305.5:c.328A>C	ENSP00000300305.3:p.Lys110Gln	no	no	yes	D	no	+	n.d.	n.d.	n.d.
	TET2	chr4	105272692	10/11	ENST00000380013.6:c.4317dup	ENSP00000369351.4:p.Arg1440ThrfsTer38	no	no	no	C	yes	+	n.d.	n.d.	n.d.
	NRAS	chr1	114713907	3/7	ENST00000369535.4:c.183A>C	ENSP00000358548.4:p.Gln61His	yes	yes	yes	A	yes	+	n.d.	n.d.	n.d.
2-051	NPM1	chr5	171410539	12/12	ENST00000517671.3:c.860_863dup	ENSP00000428755.1:p.Trp288CysfsTer12	yes	yes	no	A	yes	+	n.d.	n.d.	n.d.
	IDH2	chr15	90088702	4/11	ENST00000330062.5:c.419G>A	ENSP00000331897.3:p.Arg140Gln	yes	yes	yes	A	yes	+	n.d.	n.d.	n.d.
2-052	IDH1	chr2	208248388	4/10	ENST00000345146.4:c.395G>A	ENSP00000260985.2:p.Arg132His	yes	yes	yes	A	yes	+	n.d.	n.d.	n.d.
2-053	KIT	chr4	54733154	17/21	ENST00000288135.5:c.2446G>T	ENSP00000288135.5:p.Asp816Tyr	yes	yes	yes	A	yes	+	n.d.	n.d.	n.d.
	NRAS	chr1	114713907	3/7	ENST00000369535.4:c.183A>C	ENSP00000358548.4:p.Gln61His	yes	yes	yes	A	yes	+	n.d.	n.d.	n.d.
2-054	DNMT3A	chr2	25240727	18/23	ENST00000321117.7:c.2086C>T	ENSP00000324375.5:p.Gln696Ter	no	yes	no	B	yes	+	n.d.	n.d.	n.d.
3-001	TET2	chr4	105234042	3/11	ENST00000380013.6:c.100C>T	ENSP00000369351.4:p.Leu34Phe	no	no	yes	D	no	+	n.d.	n.d.	n.d.
	TET2	chr4	105272692	10/11	ENST00000380013.6:c.4317dup	ENSP00000369351.4:p.Arg1440ThrfsTer38	no	no	no	C	yes	-	+	n.d.	n.d.
3-003	WT1	chr11	32392029	9/10	ENST00000332351.5:c.1374_1375insCTCT	ENSP00000331327.3:p.Lys459LeufsTer2	no	no	no	C	yes	-	+	n.d.	n.d.
	CEBPA	chr19	33302214	1/1	ENST00000498907.2:c.196_200dup	ENSP00000427514.1:p.Tyr67Ter	no	yes	no	B	yes	+	n.d.	n.d.	n.d.
3-004	DNMT3A	chr2	25234374	23/23	ENST00000321117.7:c.2644C>T	ENSP00000324375.5:p.Arg882Cys	yes	yes	yes	A	yes	+	n.d.	n.d.	n.d.
3-005	DNMT3A	chr2	25234373	23/23	ENST00000321117.7:c.2645G>A	ENSP00000324375.5:p.Arg882His	yes	yes	yes	A	yes	+	n.d.	n.d.	n.d.
	TET2	chr4	105234042	3/11	ENST00000380013.6:c.100C>T	ENSP00000369351.4:p.Leu34Phe	no	no	yes	D	no	+	n.d.	n.d.	n.d.
	TET2	chr4	105236491	3/11	ENST00000380013.6:c.2549A>G	ENSP00000369351.4:p.His850Arg	no	no	yes	D	no	+	n.d.	n.d.	n.d.
	NPM1	chr5	171410539	12/12	ENST00000517671.3:c.860_863dup	ENSP00000428755.1:p.Trp288CysfsTer12	yes	yes	no	A	yes	+	n.d.	n.d.	n.d.
3-006	DNMT3A	chr2	25245284	13/23	ENST00000321117.7:c.1522del	ENSP00000324375.5:p.Leu508SerfsTer143	no	no	no	C	yes	+	n.d.	n.d.	n.d.
	IDH1	chr2	208243577	6/10	ENST00000345146.4:c.548A>G	ENSP00000260985.2:p.Tyr183Cys	no	no	yes	D	no	+	n.d.	n.d.	n.d.
	RUNX1	chr21	34792566	8/8	ENST00000300305.5:c.1011dup	ENSP00000300305.3:p.Ala338ArgfsTer262	no	yes	no	B	yes	+	n.d.	n.d.	n.d.
	IDH2	chr15	90088702	4/11	ENST00000330062.5:c.419G>A	ENSP00000331897.3:p.Arg140Gln	yes	yes	yes	A	yes	+	n.d.	n.d.	n.d.
3-007	FLT3	chr13	28049450	8/24	ENST00000241453.9:c.970G>A	ENSP00000241453.7:p.Asp324Asn	no	yes	yes	B	no	+	n.d.	n.d.	n.d.
	KIT	chr4	54733155	17/21	ENST00000288135.5:c.2447A>T	ENSP00000288135.5:p.Asp816Val	yes	yes	yes	A	yes	+	n.d.	n.d.	n.d.
	TET2	chr4	105272692	10/11	ENST00000380013.6:c.4317dup	ENSP00000369351.4:p.Arg1440ThrfsTer38	no	no	no	C	yes	-	-	+	n.d.
	ASXL1	chr20	32435346	13/13	ENST00000375687.6:c.2634T>A	ENSP00000364839.4:p.Ser878Arg	yes	no	no	C	no	+	n.d.	n.d.	n.d.
3-009	DNMT3A	chr2	25240313	19/23	ENST00000321117.7:c.2311C>T	ENSP00000324375.5:p.Arg771Ter	no	yes	no	B	yes	+	n.d.	n.d.	n.d.
	IDH1	chr2	208248389	4/10	ENST00000345146.4:c.394C>G	ENSP00000260985.2:p.Arg132Gly	yes	yes	yes	A	yes	+	n.d.	n.d.	n.d.
	NPM1	chr5	171410539	12/12	ENST00000517671.3:c.860_863dup	ENSP00000428755.1:p.Trp288CysfsTer12	yes	yes	no	A	yes	+	n.d.	n.d.	n.d.
3-010	FLT3	chr13	28018503	20/24	ENST00000241453.9:c.2505T>G	ENSP00000241453.7:p.Asp835Glu	yes	yes	yes	A	yes	+	n.d.	n.d.	n.d.
	IDH1	chr2	208243577	6/10	ENST00000345146.4:c.548A>G	ENSP00000260985.2:p.Tyr183Cys	no	no	yes	D	no	+	n.d.	n.d.	n.d.
3-011	FLT3	chr13	28034113	14/24	ENST00000241453.9:c.1773_1805dup	ENSP00000241453.7:p.Leu601_Lys602insAsnValAspPheArgGluTyrGluTyrAspLeu	yes	yes	no	A	no	+	n.d.	n.d.	n.d.
	NPM1	chr5	171410539	12/12	ENST00000517671.3:c.860_863dup	ENSP00000428755.1:p.Trp288CysfsTer12	yes	yes	no	A	yes	+	n.d.	n.d.	n.d.
	CEBPA	chr19	33302046	1/1	ENST00000498907.2:c.368dup	ENSP00000427514.1:p.Ala124SerfsTer46	no	no	no	C	yes	+	n.d.	n.d.	n.d.
3-012	DNMT3A	chr2	25234374	23/23	ENST00000321117.7:c.2644C>A	ENSP00000324375.5:p.Arg882Ser	yes	yes	yes	A	no	+	n.d.	n.d.	n.d.
	NPM1	chr5	171410539	12/12	ENST00000517671.3:c.860_863dup	ENSP00000428755.1:p.Trp288CysfsTer12	yes	yes	no	A	yes	+	n.d.	n.d.	n.d.

	WT1	chr11	32434715	1/10	ENST00000332351.5:c.630dup	ENSP00000331327.3:p.Ala211ArgfsTer37	no	no	no	C	yes	+	n.d.	n.d.	n.d.
	IDH1	chr2	208243532	6/10	ENST00000345146.4:c.593A>G	ENSP00000260985.2:p.Gln198Arg	no	no	yes	D	no	+	n.d.	n.d.	n.d.
3-013	TET2	chr4	105235995	3/11	ENST00000380013.6:c.2053C>T	ENSP00000369351.4:p.Gln685Ter	no	no	no	C	yes	+	n.d.	n.d.	n.d.
	NPM1	chr5	171410539	12/12	ENST00000517671.3:c.860_863dup	ENSP00000428755.1:p.Trp288CysfsTer12	yes	yes	no	A	yes	+	n.d.	n.d.	n.d.
3-014	TET2	chr4	105272692	10/11	ENST00000380013.6:c.4317dup	ENSP00000369351.4:p.Arg1440ThrfsTer38	no	no	no	C	yes	+	n.d.	n.d.	n.d.
	TP53	chr17	7674220	7/11	ENST00000269305.6:c.743G>A	ENSP00000269305.4:p.Arg248Gln	no	yes	yes	B	yes	+	n.d.	n.d.	n.d.
3-015	TET2	chr4	105272591	10/11	ENST00000380013.6:c.4210C>T	ENSP00000369351.4:p.Arg1404Ter	no	yes	no	B	yes	-	+	n.d.	n.d.
	IDH2	chr15	90088702	4/11	ENST00000330062.5:c.419G>A	ENSP00000331897.3:p.Arg140Gln	yes	yes	yes	A	yes	+	n.d.	n.d.	n.d.
3-016	IDH2	chr15	90088702	4/11	ENST00000330062.5:c.419G>A	ENSP00000331897.3:p.Arg140Gln	yes	yes	yes	A	yes	+	n.d.	n.d.	n.d.
	DNMT3A	chr2	25234373	23/23	ENST00000321117.7:c.2645G>A	ENSP00000324375.5:p.Arg882His	yes	yes	yes	A	yes	+	n.d.	n.d.	n.d.
	FLT3	chr13	28018505	20/24	ENST00000241453.9:c.2503G>T	ENSP00000241453.7:p.Asp835Tyr	yes	yes	no	A	yes	+	n.d.	n.d.	n.d.
	IDH1	chr2	208251458	3/10	ENST00000345146.4:c.94T>G	ENSP00000260985.2:p.Phe32Val	no	yes	yes	B	no	+	n.d.	n.d.	n.d.
3-017	RUNX1	chr21	34792448	8/8	ENST00000300305.5:c.1129dup	ENSP00000300305.3:p.Tyr377LeufsTer223	no	no	no	C	yes	+	n.d.	n.d.	n.d.
	RUNX1	chr21	34887027	3/8	ENST00000300305.5:c.167T>C	ENSP00000300305.3:p.Leu56Ser	no	yes	yes	B	no	+	n.d.	n.d.	n.d.
	TET2	chr4	105234763	3/11	ENST00000380013.6:c.822del	ENSP00000369351.4:p.Asn275IlefsTer18	no	yes	no	B	yes	+	n.d.	n.d.	n.d.
	TET2	chr4	105259626	7/11	ENST00000380013.6:c.3812dup	ENSP00000369351.4:p.Cys1271TrpfsTer29	no	yes	no	B	yes	+	n.d.	n.d.	n.d.
	NRAS	chr1	114716123	2/7	ENST00000369535.4:c.38G>A	ENSP00000358548.4:p.Gly13Asp	yes	yes	no	A	yes	+	n.d.	n.d.	n.d.
3-018	IDH1	chr2	208248388	4/10	ENST00000345146.4:c.395G>A	ENSP00000260985.2:p.Arg132His	yes	yes	yes	A	yes	+	n.d.	n.d.	n.d.
	DNMT3A	chr2	25244259	15/23	ENST00000321117.7:c.1747T>A	ENSP00000324375.5:p.Cys583Ser	no	no	no	C	no	-	+	n.d.	n.d.
3-019	FLT3	chr13	28035621	12/24	ENST00000241453.9:c.1471G>T	ENSP00000241453.7:p.Val491Leu	no	yes	no	B	no	+	n.d.	n.d.	n.d.
	NPM1	chr5	171410539	12/12	ENST00000517671.3:c.860_863dup	ENSP00000428755.1:p.Trp288CysfsTer12	yes	yes	no	A	yes	+	n.d.	n.d.	n.d.
	NRAS	chr1	114716126	2/7	ENST00000369535.4:c.35G>A	ENSP00000358548.4:p.Gly12Asp	yes	yes	no	A	yes	+	n.d.	n.d.	n.d.
	DNMT3A	chr2	25234308	23/23	ENST00000321117.7:c.2710C>A	ENSP00000324375.5:p.Pro904Thr	no	yes	no	B	no	+	n.d.	n.d.	n.d.
	ASXL1	chr20	32435669	13/13	ENST00000375687.6:c.2957A>G	ENSP00000364839.4:p.Asn986Ser	yes	yes	yes	A	no	+	n.d.	n.d.	n.d.
3-020	NPM1	chr5	171410539	12/12	ENST00000517671.3:c.860_863dup	ENSP00000428755.1:p.Trp288CysfsTer12	yes	yes	no	A	yes	+	n.d.	n.d.	n.d.
	IDH2	chr15	90087472	6/11	ENST00000330062.5:c.782G>A	ENSP00000331897.3:p.Arg261His	no	no	yes	D	no	+	n.d.	n.d.	n.d.
	NRAS	chr1	114716126	2/7	ENST00000369535.4:c.35G>A	ENSP00000358548.4:p.Gly12Asp	yes	yes	no	A	yes	+	n.d.	n.d.	n.d.
	DNMT3A	chr2	25234413	23/23	ENST00000321117.7:c.2604del	ENSP00000324375.5:p.Phe868LeufsTer13	no	no	no	C	yes	-	+	n.d.	n.d.
3-021	NPM1	chr5	171410539	12/12	ENST00000517671.3:c.860_863dup	ENSP00000428755.1:p.Trp288CysfsTer12	yes	yes	no	A	yes	+	n.d.	n.d.	n.d.
	IDH2	chr15	90088702	4/11	ENST00000330062.5:c.419G>A	ENSP00000331897.3:p.Arg140Gln	yes	yes	yes	A	yes	+	n.d.	n.d.	n.d.
3-022	NRAS	chr1	114713908	3/7	ENST00000369535.4:c.182A>G	ENSP00000358548.4:p.Gln61Arg	yes	yes	no	A	yes	+	n.d.	n.d.	n.d.
	TET2	chr4	105261769	8/11	ENST00000380013.6:c.3965T>G	ENSP00000369351.4:p.Leu1322Arg	no	yes	no	B	yes	+	n.d.	n.d.	n.d.
3-023	NPM1	chr5	171410540	12/12	ENST00000517671.3:c.863_864insCCTG	ENSP00000428755.1:p.Trp288CysfsTer12	yes	yes	no	A	yes	+	n.d.	n.d.	n.d.
	IDH2	chr15	90088702	4/11	ENST00000330062.5:c.419G>A	ENSP00000331897.3:p.Arg140Gln	yes	yes	yes	A	yes	+	n.d.	n.d.	n.d.
3-024	TP53	chr17	7674259	7/11	ENST00000269305.6:c.704A>G	ENSP00000269305.4:p.Asn235Ser	no	yes	yes	B	no	+	n.d.	n.d.	n.d.
	ASXL1	chr20	32434599	13/13	ENST00000375687.6:c.1900_1922del	ENSP00000364839.4:p.Glu635ArgfsTer15	yes	yes	no	A	yes	-	+	n.d.	n.d.
3-025	CEBPA	chr19	33301825	1/1	ENST00000498907.2:c.584_589dup	ENSP00000427514.1:p.His195_Pro196dup	no	no	no	C	no	-	-	+	n.d.

3-026	WT1	chr11	32396361	7/10	ENST00000332351.5:c.1141_1144dup	ENSP00000331327.3:p.Ala382ValfsTer4	no	yes	no	B	yes	-	-	+	n.d.
3-027	FLT3	chr13	28034125	14/24	ENST00000241453.9:c.1770_1793dup	ENSP00000241453.7:p.Tyr597_Glu598insAspTyrValAspPheArgGluTyr	yes	yes	no	A	no	+	n.d.	n.d.	n.d.
	NPM1	chr5	171410540	12/12	ENST00000517671.3:c.863_864insCCTG	ENSP00000428755.1:p.Trp288CysfsTer12	yes	yes	no	A	yes	+	n.d.	n.d.	n.d.
3-029	IDH2	chr15	90084321	11/11	ENST00000330062.5:c.1304C>T	ENSP00000331897.3:p.Thr435Met	no	no	yes	D	no	+	n.d.	n.d.	n.d.
3-030	ASXL1	chr20	32434733	13/13	ENST00000375687.6:c.2024dup	ENSP00000364839.4:p.Glu676Ter	yes	no	no	C	yes	+	n.d.	n.d.	n.d.
	IDH2	chr15	90088702	4/11	ENST00000330062.5:c.419G>A	ENSP00000331897.3:p.Arg140Gln	yes	yes	yes	A	yes	+	n.d.	n.d.	n.d.
3-031	FLT3	chr13	28049450	8/24	ENST00000241453.9:c.970G>A	ENSP00000241453.7:p.Asp324Asn	no	yes	yes	B	no	+	n.d.	n.d.	n.d.
	RUNX1	chr21	34886881	3/8	ENST00000300305.5:c.313C>A	ENSP00000300305.3:p.His105Asn	no	no	no	C	no	+	n.d.	n.d.	n.d.
3-032	RUNX1	chr21	34887027	3/8	ENST00000300305.5:c.167T>C	ENSP00000300305.3:p.Leu56Ser	no	yes	yes	B	no	+	n.d.	n.d.	n.d.
	NPM1	chr5	171410542	12/12	ENST00000517671.3:c.863_864insCCGG	ENSP00000428755.1:p.Trp288CysfsTer12	yes	yes	no	A	yes	+	n.d.	n.d.	n.d.
	CEBPA	chr19	33301825	1/1	ENST00000498907.2:c.584_589dup	ENSP00000427514.1:p.His195_Pro196dup	no	no	no	C	no	-	+	n.d.	n.d.
3-034	IDH1	chr2	208248389	4/10	ENST00000345146.4:c.394C>T	ENSP00000260985.2:p.Arg132Cys	yes	yes	yes	A	yes	+	n.d.	n.d.	n.d.
	TET2	chr4	105275662	11/11	ENST00000380013.6:c.5152G>T	ENSP00000369351.4:p.Val1718Leu	no	yes	yes	B	no	+	n.d.	n.d.	n.d.
	NPM1	chr5	171410539	12/12	ENST00000517671.3:c.860_863dup	ENSP00000428755.1:p.Trp288CysfsTer12	yes	yes	no	A	yes	+	n.d.	n.d.	n.d.
	CEBPA	chr19	33302176	1/1	ENST00000498907.2:c.238dup	ENSP00000427514.1:p.Asp80GlyfsTer28	no	yes	no	B	yes	+	n.d.	n.d.	n.d.
3-035	IDH1	chr2	208248389	4/10	ENST00000345146.4:c.394C>A	ENSP00000260985.2:p.Arg132Ser	yes	yes	yes	A	yes	+	n.d.	n.d.	n.d.
	NPM1	chr5	171410539	12/12	ENST00000517671.3:c.860_863dup	ENSP00000428755.1:p.Trp288CysfsTer12	yes	yes	no	A	yes	+	n.d.	n.d.	n.d.
	CEBPA	chr19	33301984	1/1	ENST00000498907.2:c.430dup	ENSP00000427514.1:p.Glu144GlyfsTer26	no	no	no	C	yes	+	n.d.	n.d.	n.d.
3-037	DNMT3A	chr2	25300227	3/23	ENST00000321117.7:c.89A>C	ENSP00000324375.5:p.Glu30Ala	no	yes	yes	B	no	-	+	n.d.	n.d.
	NPM1	chr5	171410539	12/12	ENST00000517671.3:c.860_863dup	ENSP00000428755.1:p.Trp288CysfsTer12	yes	yes	no	A	yes	+	n.d.	n.d.	n.d.
	IDH2	chr15	90088702	4/11	ENST00000330062.5:c.419G>A	ENSP00000331897.3:p.Arg140Gln	yes	yes	yes	A	yes	+	n.d.	n.d.	n.d.
3-038	DNMT3A	chr2	25239194	20/23	ENST00000321117.7:c.2340_2343del	ENSP00000324375.5:p.Ile780MetfsTer21	no	no	no	C	yes	+	n.d.	n.d.	n.d.
	IDH2	chr15	90088702	4/11	ENST00000330062.5:c.419G>A	ENSP00000331897.3:p.Arg140Gln	yes	yes	yes	A	yes	+	n.d.	n.d.	n.d.
3-039	FLT3	chr13	28028243	16/24	ENST00000241453.9:c.1988A>G	ENSP00000241453.7:p.Lys663Arg	no	yes	no	B	no	+	n.d.	n.d.	n.d.
3-040	DNMT3A	chr2	25246023	12/23	ENST00000321117.7:c.1470dup	ENSP00000324375.5:p.Glu491Ter	no	no	no	C	yes	-	-	+	n.d.
	IDH2	chr15	90088606	4/11	ENST00000330062.5:c.515G>A	ENSP00000331897.3:p.Arg172Lys	yes	yes	yes	A	yes	+	n.d.	n.d.	n.d.
3-041	DNMT3A	chr2	25234373	23/23	ENST00000321117.7:c.2645G>A	ENSP00000324375.5:p.Arg882His	yes	yes	yes	A	yes	+	n.d.	n.d.	n.d.
	NPM1	chr5	171410539	12/12	ENST00000517671.3:c.860_863dup	ENSP00000428755.1:p.Trp288CysfsTer12	yes	yes	no	A	yes	+	n.d.	n.d.	n.d.
3-042	TET2	chr4	105236554	3/11	ENST00000380013.6:c.2613dup	ENSP00000369351.4:p.Val872CysfsTer29	no	no	no	C	yes	+	n.d.	n.d.	n.d.
	TET2	chr4	105269725	9/11	ENST00000380013.6:c.4160A>G	ENSP00000369351.4:p.Asn1387Ser	no	no	no	C	yes	+	n.d.	n.d.	n.d.
	TET2	chr4	105272692	10/11	ENST00000380013.6:c.4317dup	ENSP00000369351.4:p.Arg1440ThrfsTer38	no	no	no	C	yes	+	n.d.	n.d.	n.d.
	ASXL1	chr20	32434789	13/13	ENST00000375687.6:c.2077C>T	ENSP00000364839.4:p.Arg693Ter	yes	yes	yes	A	yes	+	n.d.	n.d.	n.d.
	CEBPA	chr19	33302346	1/1	ENST00000498907.2:c.68dup	ENSP00000427514.1:p.His24AlafsTer84	no	yes	yes	B	yes	+	n.d.	n.d.	n.d.

[1] Number of read pairs were downscaled for each sample to 47.5, 40, 30 and 20 million. HAMLET was first run on all samples with 20 million read pairs followed by analysis with 30, 40 and 47.5 million read pairs for those variants that were not detected with 20 million read pairs. The sequential analysis was stopped upon detection of the variant. Variants that are detected (+) are indicated in blue, while variants that are not detected (-) are shown in orange.

Variants indicated in yellow are nonsense variants or frameshift variants with a premature stop codon that are potential targets for nonsense mediated decay.
n.d.; not determined.

[2] Position of the variant in the GRCh38 human genome.

[3] Exon location of the variant / total number of exons of the gene.

[4] HGVSc; coding sequence name as identified by the Human Genome Variation Society (http://www.hgvs.org/).

[5] HGVSp; protein sequence name as identified by the Human Genome Variation Society (http://www.hgvs.org/).

[6] HS; indicated whether the variant occurs in a hotspot (HS) region as identified in Table SVI.

[7] COS; indicated whether the variant is known as cancer mutation in COSMIC (catalogue of somatic mutations in cancer; http://cancer.sanger.ac.uk/cosmic).

[8] SNP; indicated whether the variant is known as single nucleotide polymorphism in dbSNP (https://www.ncbi.nlm.nih.gov/projects/SNP).

[9] Class; A, hotspot variant known in COSMIC (known or unknown as SNP); B, variant not in a hotspot but known in COSMIC (known or unknown as SNP); C, variant unknown in COSMIC and unknown as SNP; D, variant unknown in COSMIC and known as SNP.

[10] Jaiswal; indicated whether the variant is a recurrent mutation in myeloid malignancies by Jaiswal *et al.* (NEJM 2017).

Table SXII. Detection of *FLT3*-ITD by ReSCU (*R*eciprocal *S*oft *C*lip *U*tilization)[1].

AML	med cov ex14-15[2]	med cov up itd[3]	med cov itd[4]	med cov down itd[5]	itd start[6]	itd end[7]	Itd length[8]	sc start count[9]	% sc start count[10]	sc end count[11]	% sc end count[12]	sc start pos[13]	sc start recipr pos[14]	sc end pos[15]	sc end recipr pos[16]	boundary type[17]	fuzziness[18]	Validation[19]	diagnostic PCR[20]
1-002	1456	1571	1417	1465	1815	1868	54	52	3.6	58	4.0	1814	1868	1869	1815	exact	0	yes	pos
1-003	2025	1964	2230	2003	1807	1875	69	245	12.5	272	13.8	1806	1875	1876	1807	exact	0	yes	pos
	2025	2067	2218	2003	1828,1829	1911,1912	84	49	2.4	30	1.5	1827	1911	1913	1829	fuzzy	1	not detected	
2-001	681	642	859	678	1859,1863	1906,191	48	119	17.5	102	15.0	1858	1906	1911	1863	fuzzy	4	yes	pos
2-006	1258	1072	1131	1274	1857,1861	1877,1881	21	154	12.2	141	11.2	1856	1877	1882	1861	fuzzy	4	yes	pos
2-012	815	808	761	819	1859,1861	1870,1872	12	15	1.8	12	1.5	1858	1870	1873	1861	fuzzy	2	yes	pos
2-013	702	647	704	713	1821,1824	1883	63	92	13.1	69	9.8	1823	1883	1884	1821	fuzzy-start	3	yes	pos
	702	599	706	713	1824,1828	1883	60	92	13.1	4	0.6	1823	1883	1884	1828	fuzzy-start	4	not detected	
2-014	403	-	-	-	-	-	-	-	-	-	-	-	-	-	-	-	-	-	neg
2-015	1050	1062	1234	1039	1824	1874	51	196	18.7	157	15.0	1823	1874	1875	1824	exact	0	yes	pos
2-016	972	678	1113	712	1803	1918	116	238	24.5	245	25.2	1802	1918	1919	1803	exact	0	not detected	pos
2-018	1104	1262	987	1122	1824	1874	51	14	1.3	7	0.6	1823	1874	1875	1824	exact	0	yes	pos
	1104	1088	1024	1126	1862	1885	24	175	15.9	153	13.9	1861	1885	1886	1862	exact	0	yes	
2-023	2472	2247	2905	2481	1830,1831	1883,1884	54	17	0.7	10	0.4	1829	1883	1885	1831	fuzzy	1	yes	pos
	2472	2233	2946	2472	1833	1871	39	523	21.2	313	12.7	1832	1871	1872	1833	exact	0	yes	
2-028	641	674	682	629	1863	1919,1922	60	3	0.5	6	0.9	1862	1922	1920	1863	fuzzy-end	3	-	neg
2-029	2228	1848	3243	2199	1843,1847	1911,1915	69	848	38.1	588	26.4	1842	1911	1916	1847	fuzzy	4	yes	pos
2-030	1048	1083	1318	1030	1810,1813	1875,1877	66	215	20.5	199	19.0	1812	1877	1876	1810	fuzzy	3	yes	pos
2-031	558	541	533	560	1830	1874	45	6	1.1	4	0.7	1829	1874	1875	1830	exact	0	yes	pos
	558	534	529	560	1861	1874	14	6	1.1	14	2.5	1860	1874	1875	1861	exact	0	yes	
2-032	1004	1031	1066	983	1861	1874	14	111	11.1	124	12.4	1860	1874	1875	1861	exact	0	yes	pos
2-033	1432	1583	1369	1432	1828,1829	1872,1873	45	13	0.9	9	0.6	1827	1872	1874	1829	fuzzy	1	yes	pos
	1432	1431	1429	1434	1860	1883	24	326	22.8	280	19.6	1859	1883	1884	1860	exact	0	yes	
2-035	1702	1722	1800	1684	1857	1874	18	261	15.3	265	15.6	1856	1874	1875	1857	exact	0	yes	pos
2-036	2704	2769	2930	2653	1812	1868	57	39	1.4	26	1.0	1811	1868	1869	1812	exact	0	not detected	pos
	2704	2766	2931	2621	1813	1909	97	277	10.2	231	8.5	1812	1909	1910	1813	exact	0	not detected	
	2704	2881	3207	2631	1875	1891	17	9	0.3	4	0.1	1874	1891	1892	1875	exact	0	yes	
2-040	2117	1805	2689	2091	1843	1919	77	422	19.9	341	16.1	1842	1919	1920	1843	exact	0	not detected	pos
2-045	379	394	353	394	1856	1882	27	4	1.1	3	0.8	1855	1882	1883	1856	exact	0	yes	pos
	379	347	378	390	1857,1859	1919,1921	63	14	3.7	8	2.1	1858	1921	1920	1857	fuzzy	2	not detected	
2-046	656	589	772	651	1836,1840	1900,1905	65	106	16.2	85	13.0	1839	1905	1901	1836	fuzzy	5	yes	pos
2-049	630	714	641	612	1818	1875,1877	60	22	3.5	13	2.1	1817	1877	1876	1818	fuzzy-end	2	yes	pos

2-052	226	223	238	222	1800	1918,1920	121	3	1.3	5	2.2	1799	1920	1919	1800	fuzzy-end	2	not detected	pos
	226	226	276	223	1863	1918	56	13	5.6	17	7.5	1862	1918	1919	1863	exact	0	yes	
3-005	525	512	613	522	1823,1824	1861,1862	39	95	18.1	60	11.4	1822	1861	1863	1824	fuzzy	1	yes	pos
3-009	1780	1678	1601	1811	1828,1829	1872,1873	45	7	0.4	3	0.2	1827	1872	1874	1829	fuzzy	1	not detected	pos
3-011	965	806	1055	978	1853,1854	1885,1886	33	221	22.9	170	17.9	1852	1885	1887	1854	fuzzy	1	yes	pos
3-012	1705	1454	2168	1695	1815	1859	45	678	39.8	364	22.3	1814	1859	1860	1815	exact	0	yes	pos
3-017	1029	972	1074	1045	1865,1866	1918,1919	54	5	0.5	4	0.4	1864	1918	1920	1866	fuzzy	1	-	neg
3-020	455	439	477	454	1825	1919,1923	99	12	2.6	10	2.2	1824	1923	1920	1825	fuzzy-end	4	not detected	pos
3-026	1366	1345	1626	1356	1828,1829	1878,1879	51	247	18.1	137	10.0	1827	1878	1880	1829	fuzzy	1	yes	pos
3-027	2201	2164	2066	2206	1825	1872	48	9	0.4	10	0.5	1824	1872	1873	1825	exact	0	not detected	pos
	2201	2164	2066	2206	1825,1827	1868,1872	48	9	0.4	3	0.1	1824	1872	1869	1827	fuzzy	4	not detected	
	2201	2099	1857	2206	1831,1832	1860,1861	30	7	0.3	4	0.2	1830	1860	1862	1832	fuzzy	1	not detected	
	2201	2064	2219	2206	1851	1871	21	8	0.4	4	0.2	1850	1871	1872	1851	exact	0	yes	
	2201	2064	2216	2207	1851	1871,1874	24	298	13.5	229	10.4	1850	1874	1872	1851	fuzzy-end	3	yes	
	2201	2064	2216	2207	1851	1874	24	298	13.5	229	10.4	1850	1874	1875	1851	exact	0	yes	
3-028	1957	1596	2452	1948	1843,1847	1911,1915	69	486	24.8	310	15.8	1842	1911	1916	1847	fuzzy	4	yes	pos
3-035	836	687	823	873	1861,1863	1914,1916	54	14	1.7	9	1.1	1860	1914	1917	1863	fuzzy	2	yes	pos
	836	687	776	871	1868	1887	20	32	3.8	33	3.9	1867	1887	1888	1868	exact	0	yes	
3-037	1187	941	1341	1183	1826	1874	49	194	16.3	111	9.4	1825	1874	1875	1826	exact	0	yes	pos
	1187	1046	1205	1177	1853,1855	1919,1922	68	12	1.0	6	0.5	1854	1922	1920	1853	fuzzy	3	not detected	
3-039	1894	1706	1712	1912	1865	1891	27	5	0.3	4	0.2	1864	1891	1892	1865	exact	0	not detected	pos
3-041	787	718	799	791	1816	1860	45	135	17.2	95	12.1	1815	1860	1861	1816	exact	0	yes	pos

[1] In ReSCU, RNAseq reads that partially align with the *FLT3* primary gene transcript ENST00000241453 are selected as soft-clipped (SC) reads. The positions of the SC indicate the start and end of the ITD. SC reads aligning to exon 14-15 of the *FLT3* transcript (1787-2024 base pair region of the transcript corresponding to 1705-1942 base pair region in the coding sequence) are extracted and analyzed for reciprocity. Reciprocal events are detected when SC regions of reads at the start of the ITD align with transcript regions at the end of the ITD, and vice versa. Samples are called positive for *FLT3*-ITD upon detection of reciprocal events.

[2] median number of reads at each base pair in exon 14-15.

[3] median number of reads at each base pair in exon 14-15 upstream of the ITD as determined by the positions of SC.

[4] median number of reads at each base pair in the ITD as determined by the positions of SC.

[5] median number of reads at each base pair in exon 14-15 downstream of the ITD as determined by the positions of SC.

[6] Start of ITD as determined by the position of the SC +1.

[7] End of ITD as determined by the position of the SC -1.

[8] Length of the ITD as determined the position s of SCs.

[9] SC read counts at start of the ITD.

[10] Percentage of SC reads of median number of reads at each base pair in exon 14-15 at start of the ITD.

[11] SC read counts at end of the ITD.

[12] Percentage of SC reads of median number of reads at each base pair in exon 14-15 at end of the ITD.
[13] Position of the SC at start of the ITD.
[14] Position of the reciprocal event after alignment of the SC region of reads at start of the ITD.
[15] Position of the SC at end of the ITD.
[16] Position of the reciprocal event after alignment of the SC region of reads at end of the ITD.
[17] Indicated whether the position of the SC at start and/or end of the ITD is exact or fuzzy.
[18] Number of possible bases for the position of the SC at start and/or end of the ITD.
[19] Validation of the presence, position and length of the ITD by MiSeq NGS.
[20] Presence and position of the ITD was validated, but the length of the ITD was too long to determine by NGS.
[21] Diagnostic results for *FLT3*-ITD as measured by PCR on genomic DNA followed by electrophoretic fragment size analysis.

Table SXIII. Sensitivity of HAMLET to detect *FLT3*-ITD at different sequencing depths[1].

AML	itd start[2]	itd end[3]	Itd length[4]	% sc start count[5]	% sc end count[6]	20M	30M	40M	47.5M
1-002	1815	1868	54	3.6	4.0	+	n.d.	n.d.	n.d.
1-003	1807	1875	69	12.5	13.8	+	n.d.	n.d.	n.d.
	1828,1829	1911,1912	84	2.4	1.5	+	n.d.	n.d.	n.d.
2-001	1859,1863	1906,191	48	17.5	15.0	+	n.d.	n.d.	n.d.
2-006	1857,1861	1877,1881	21	12.2	11.2	+	n.d.	n.d.	n.d.
2-012	1859,1861	1870,1872	12	1.8	1.5	+	n.d.	n.d.	n.d.
2-013	1821,1824	1883	63	13.1	9.8	+	n.d.	n.d.	n.d.
	1824,1828	1883	60	13.1	0.6	+	n.d.	n.d.	n.d.
2-015	1824	1874	51	18.7	15.0	+	n.d.	n.d.	n.d.
2-016	1803	1918	116	24.5	25.2	+	n.d.	n.d.	n.d.
2-018	1824	1874	51	1.3	0.6	+	n.d.	n.d.	n.d.
	1862	1885	24	15.9	13.9	+	n.d.	n.d.	n.d.
2-023	1830,1831	1883,1884	54	0.7	0.4	-	+	n.d.	n.d.
	1833	1871	39	21.2	12.7	+	n.d.	n.d.	n.d.
2-028	1863	1919,1922	60	0.5	0.9	-	+	n.d.	n.d.
2-029	1843,1847	1911,1915	69	38.1	26.4	+	n.d.	n.d.	n.d.
2-030	1810,1813	1875,1877	66	20.5	19.0	+	n.d.	n.d.	n.d.
2-031	1830	1874	45	1.1	0.7	-	-	-	+
	1861	1874	14	1.1	2.5	-	-	-	+
2-032	1861	1874	14	11.1	12.4	+	n.d.	n.d.	n.d.
2-033	1828,1829	1872,1873	45	0.9	0.6	-	+	n.d.	n.d.
	1860	1883	24	22.8	19.6	+	n.d.	n.d.	n.d.
2-035	1857	1874	18	15.3	15.6	+	n.d.	n.d.	n.d.
2-036	1812	1868	57	1.4	1.0	+	n.d.	n.d.	n.d.
	1813	1909	97	10.2	8.5	+	n.d.	n.d.	n.d.
	1875	1891	17	0.3	0.1	-	-	-	-
2-040	1843	1919	77	19.9	16.1	+	n.d.	n.d.	n.d.
2-045	1856	1882	27	1.1	0.8	-	-	-	+
	1857,1859	1919,1921	63	3.7	2.1	-	-	+	n.d.
2-046	1836,1840	1900,1905	65	16.2	13.0	+	n.d.	n.d.	n.d.
2-049	1818	1875,1877	60	3.5	2.1	+	n.d.	n.d.	n.d.
2-052	1800	1918,1920	121	1.3	2.2	-	-	-	-
	1863	1918	56	5.6	7.5	-	-	+	n.d.
3-005	1823,1824	1861,1862	39	18.1	11.4	+	n.d.	n.d.	n.d.
3-009	1828,1829	1872,1873	45	0.4	0.2	-	-	-	-
3-011	1853,1854	1885,1886	33	22.9	17.9	+	n.d.	n.d.	n.d.
3-012	1815	1859	45	39.8	22.3	+	n.d.	n.d.	n.d.
3-017	1865,1866	1918,1919	54	0.5	0.4	-	-	-	-
3-020	1825	1919,1923	99	2.6	2.2	+	n.d.	n.d.	n.d.
3-026	1828,1829	1878,1879	51	18.1	10.0	+	n.d.	n.d.	n.d.
3-027	1825	1872	48	0.4	0.5	-	+	n.d.	n.d.
	1825,1827	1868,1872	48	0.4	0.1	-	-	-	+
	1831,1832	1860,1861	30	0.3	0.2	-	-	-	+
	1851	1871	21	0.4	0.2	-	+	n.d.	n.d.
	1851	1871,1874	24	13.5	10.4	+	n.d.	n.d.	n.d.
	1851	1874	24	13.5	10.4	+	n.d.	n.d.	n.d.
3-028	1843,1847	1911,1915	69	24.8	15.8	+	n.d.	n.d.	n.d.
3-035	1861,1863	1914,1916	54	1.7	1.1	-	-	+	n.d.
	1868	1887	20	3.8	3.9	+	n.d.	n.d.	n.d.
3-037	1826	1874	49	16.3	9.4	+	n.d.	n.d.	n.d.
	1853,1855	1919,1922	68	1.0	0.5	-	-	-	-
3-039	1865	1891	27	0.3	0.2	-	-	-	-

3-041	1816	1860	45	17.2	12.1	+	n.d.	n.d.	n.d.

[1] Number of read pairs were downscaled for each sample to 47.5, 40, 30 and 20 million. HAMLET was first run on all samples with 20 million read pairs followed by analysis with 30, 40 and 47.5 million read pairs for those *FLT3*-ITDs that were not detected with 20 million read pairs. The sequential analysis was stopped upon detection of the ITD. ITDs that were detected (+) are indicated in blue, and ITDs that were not detected (-) are shown in orange. n.d.; not determined.

[2] Start of ITD as determined by the position of the SC +1.

[3] End of ITD as determined by the position of the SC -1.

[4] Length of the ITD as determined the position s of SCs.

[5] Percentage of SC reads of median number of reads at each base pair in exon 14-15 at start of the ITD.

[6] Percentage of SC reads of median number of reads at each base pair in exon 14-15 at end of the ITD.

Table SXIV. Diagnostic and NGS results for *FLT3*-ITD.

AML	Diagnostic results[1]												NGS results[2]						
	WT Size	WT AUC[3]	MUT1 Size	MUT1 AUC	MUT1 ITD[4]	MUT2 Size	MUT2 AUC	MUT2 ITD	MUT3 Size	MUT3 AUC	MUT3 ITD	Allelic ratio[5]	MUT1 ITD	MUT1 %[6]	MUT2 ITD	MUT2 %	MUT3 ITD	MUT3 %	MUT total%[7]
1-002	370	97767	427	5577	57	-	-	-	-	-	-	0.1	57	8.2	-	-	-	-	8.2
1-003	371	248620	440	258761	69	455	31403	84	-	-	-	1.2	69	36.8	-	-	-	-	36.8
2-001	371	342838	419	261321	48	-	-	-	-	-	-	0.8	48	64.7	-	-	-	-	64.7
2-006	372	292394	392	261599	20	-	-	-	-	-	-	0.9	21	82.6	-	-	-	-	82.6
2-012	371	374172	383	91292	12	-	-	-	-	-	-	0.2	12	30.1	-	-	-	-	30.1
2-013	371	344935	434	162441	63	424	7313	53	-	-	-	0.5	63	35.1	-	-	-	-	35.1
2-015	371	223500	395	6586	24	422	129805	51	-	-	-	0.6	24	1.9	51	63.3	-	-	65.2
2-016	371	72732	488	368897	117	-	-	-	-	-	-	5.1	54	15.7	20	1.0	-	-	16.7
2-018	371	294169	395	264471	24	422	11518	51	-	-	-	0.9	24	87.5	51	2.8	-	-	90.3
2-023	371	373126	410	252240	39	426	10070	55	-	-	-	0.7	39	82.9	54	1.4	-	-	84.3
2-029	371	112763	440	225823	69	-	-	-	-	-	-	2.0	69	100.0	-	-	-	-	100
2-030	371	402750	437	285082	66	-	-	-	-	-	-	0.7	66	53.7	-	-	-	-	53.7
2-031	371	322941	389	3369	18	416	1469	45	-	-	-	0.0	18	9.4	45	1.9	-	-	11.3
2-032	371	317074	389	5008	18	416	3265	45	-	-	-	0.0	18	100.0	-	-	-	-	100
2-033	372	175475	395	318914	23	416	16256	44	-	-	-	1.9	24	98.6	45	1.4	-	-	100
2-035	372	273144	389	253271	18	-	-	-	-	-	-	0.9	18	100.0	-	-	-	-	100
2-036	371	385179	390	3433	19	431	5606	60	471	33140	100	0.1	18	1.3	-	-	-	-	1.3
2-040	370	228974	452	137466	82	-	-	-	-	-	-	0.6	-	-	-	-	-	-	0.0
2-045	370	115694	400	7559	30	433	18887	63	-	-	-	0.2	30	0.8	-	-	-	-	0.8
2-046	370	144653	436	109706	66	408	2678	38	-	-	-	0.8	66	0.9	-	-	-	-	0.9
2-049	370	112342	430	8550	60	-	-	-	-	-	-	0.1	60	0.5	-	-	-	-	0.5
3-005	371	233935	410	156584	39	-	-	-	-	-	-	0.7	39	21.7	-	-	-	-	21.7
3-009	371	329610	416	10845	45	-	-	-	-	-	-	0.0	-	-	-	-	-	-	0.0
3-020	370	120019	484	9682	114	-	-	-	-	-	-	0.1	-	-	-	-	-	-	0.0
3-026	370	131710	421	90254	51	-	-	-	-	-	-	0.7	51	13.8	-	-	-	-	13.8
3-027	370	115610	394	91660	24	-	-	-	-	-	-	0.8	24	39.0	21	0.6	15	0.5	40.1
3-028	370	106326	438	153970	69	-	-	-	-	-	-	1.4	69	1.6	-	-	-	-	1.6
3-035	371	199588	392	90749	21	425	16710	54	-	-	-	0.5	27	0.6	21	7.3	-	-	7.9
3-037	370	76730	421	145402	51	440	10499	70	-	-	-	2.0	51	4.0	-	-	-	-	4.0
3-039	371	205570	398	3313	27	-	-	-	-	-	-	0.0	-	-	-	-	-	-	0.0
3-041	371	349304	416	278046	45	-	-	-	-	-	-	0.8	45	23.2	-	-	-	-	23.2

[1] Diagnostic results showed that 34 of the 100 AML cases were positive for *FLT3*-ITD. Included are 31 cases for which results were obtained in the accredited diagnostic laboratory of Leiden University Medical Center. PCR was performed on genomic DNA using NED-5'-GTAAAACGACGCCCAGTCTGAAGCAATTTAGGTATGAAAGC-3' and VIC-5'-GGAAACAGCTATGACCATGTACCTTTCAGCATTTTGACG-3' as forward and reverse primers, respectively. PCR fragments were detected after capillary electrophoresis by measuring green (VIC) and yellow (NED) dyes.

[2] NGS was performed for all cases positive for *FLT3*-ITD by routine diagnostics on cDNA using 5'-TTGGTGTTTGTCTCCTCTTCA-3' and 5'-AAGCTGTTGCGTTCATCACTT-3' as forward and reverse primers, respectively (WT fragment = 246 bp). PCR fragments were analyzed by MiSeq.

[3] AUC; average area under the curve of measurements of green (VIC) and yellow (NED) dyes.

[4] ITD; length of ITD (bp) = size MUT – size WT fragments.

[5] Allelic ratio = AUC MUT / AUC WT fragments.

[6] Read percentages are determined relative to the most frequent MUT or WT sequence (100%).

[7] Total percentage of *FLT3*-ITD reads.

Table SXV. Detection of *KMT2A*-PTD by ReSCU (*R*eciprocal *S*oft *C*lip *U*tilization)[1].

AML	ptd start[2]	ptd end[3]	ptd length[4]	ptd exons[5]	sc start count[6]	sc end count[7]	sc start pos[8]	sc start recipr pos[9]	sc end pos[10]	sc end recipr pos[11]	boundary type[12]	fuzziness [13]
2-002	453.457	4106.4110	3654	exon 2-8	53	48	452	4106	4111	457	fuzzy	4
2-008	453.457	4106.4110	3654	exon 2-8	7	12	452	4106	4111	457	fuzzy	4
2-013	523.525	3654.3656	3132	exon 3-6	4	12	522	3654	3657	525	fuzzy	2
2-027	453.455	522.524	70	exon 2	7	4	452	522	525	455	fuzzy	2
	453.456	3176.3179	2724	exon 2-3	48	41	452	3176	3180	456	fuzzy	3
2-036	453.457	4106.4110	3654	exon 2-8	40	44	452	4106	4111	457	fuzzy	4
2-040	453.457	4106.4110	3654	exon 2-8	78	48	452	4106	4111	457	fuzzy	4
2-054	451.455	4350.4354	3900	exon 2-10	3	6	450	4350	4355	455	fuzzy	4
3-017	451.455	4350.4354	3900	exon 2-10	8	6	450	4350	4355	455	fuzzy	4

[1] In ReSCU, RNAseq reads that partially align with the *KMT2A* primary gene transcript ENST00000534358 are selected as soft-clipped (SC) reads. The positions of the SC indicate the start and end of the PTD. SC reads aligning to exon 2-13 of the KMT2A transcript (456-4719 base pair region of the transcript corresponding to 433-4696 base pair region in the coding sequence) are extracted and analyzed for reciprocity. Reciprocal events are detected when SC regions of reads at the start of the PTD align with transcript regions at the end of the PTD, and vice versa. Samples are called positive for *KMT2A*-PTD upon detection of reciprocal events.

[2] Start of PTD as determined by the position of the SC +1.

[3] End of PTD as determined by the position of SC -1.

[4] Length of the PTD as determined by SC positions.

[5] Exons duplicated in the PTD as determined by SC positions.

[6] SC read counts at start of the PTD.

[7] SC read counts at end of the PTD.

[8] Position of the SC at start of the PTD.

[9] Position of the reciprocal event after alignment of the SC region of reads at start of the PTD.

[10] Position of the SC at end of the PTD.

[11] Position of the reciprocal event after alignment of the SC region of reads at end of the PTD.

[12] Indicated whether the position of the SC at start and/or end of the PTD is exact or fuzzy.

[13] Number of possible bases for the position of the SC at start and/or end of the PTD.

Table SXVI. Primers used for validation of *KMT2A*-PTD by PCR[1].

Primer	Sequence (5'- 3')	Position[1]		Size (bp)	PTD
		start (cds)	stop (cds)		
KMT2A-Fex7-8<br>KMT2A-Rex3	ATCAGGTCCAGAGCAGAGCA<br>AACATTCCACTTCCTCCATAGG	4008<br>784	5028<br>803	448<br>696	Exon 2-8<br>Exon 2-10
KMT2A-Fex6<br>KMT2A-Rex3	TGGATGCCTTCCAAAGCCTAC<br>AACATTCCACTTCCTCCATAGG	3595<br>784	3616<br>803	341	Exon 3-6
KMT2A-Fex3<br>KMT2A-Rex2	AACATTCCACTTCCTCCATAGG<br>TCTTCATCTGAGCCAAAACCT	2999<br>447	3020<br>467	193	Exon 2-3
KMT2A-Fex2<br>KMT2A-Rex2	AGTCAGAGTGCGAAGTCCC<br>TCTTCATCTGAGCCAAAACCT	468<br>447	486<br>467	70	Exon 2-2

[1] PCR was performed for 33 cycles using Phusion Flash High-Fidelity PCR Master Mix.

[2] position in coding sequence of ENST00000389506.

Table SXVII. Detection of *MECOM/EVI1* overexpression.

Sample	RNAseq[1]	Q-RT-PCR[2]
1-001	***0.49***	***0.22***
1-002	0	0.00012
1-003	0	5.83e-05
1-004	0	NA
1-005	***0.8***	***2.94***
1-006	0	4.56e-05
1-007	0	0.0049
1-008	***0.43***	***0.28***
2-001	0	1.54e-05
2-002	0	0.00011
2-003	0	9.83e-08
2-004	0	0.00015
2-005	0.00042	0.0012
2-006	0.0015	0.00014
2-007	0.00067	4.21e-05
2-008	0	1.86e-05
2-009	0	3.77e-05
2-010	0	7.89e-06
2-011	0	0.00030
2-012	0	1.4e-05
2-013	0.0021	0.00018
2-014	0	0.0024
2-015	0	4.17e-05
2-016	0	1.16e-05
2-018	0	8.83e-05
2-019	0	4.32e-05
2-020	0.0011	0.00048
2-021	0.0011	6.96e-05
2-022	0	7.56e-05
2-023	0	1.05e-05
2-024	0.059	0.092
2-025	0	3.34e-05
2-027	0.0041	0.009
2-028	0	6.89e-05
2-029	0	3.13e-05
2-030	0	0.0025
2-031	0	9.6e-05
2-032	0	3.23e-05
2-033	0	3.59e-05
2-034	0	7.78e-05
2-035	0	5.21e-05
2-036	0	NA
2-037	0.00063	NA
2-038	0	0.00022
2-039	0	4.69e-05
2-040	0	0.00029
2-041	0	0.00024
2-043	0.0024	2.34e-05
2-044	0	1.44e-05
2-045	0	2.97e-05
2-046	0	0.00016
2-047	***1.4***	***1.12***
2-048	0	8.67e-05
2-049	0	9.23e-05
2-050	0.012	0.014
2-051	0	7.79e-05
2-052	0	NA
2-053	0	4.78e-05
2-054	0	NA
3-001	0	0.00052
3-002	0	0.00026
3-003	0	5.15e-05
3-004	0.00062	0.0011
3-005	0	0.00023
3-006	0	6.94e-05
3-007	0	0.00013
3-008	***0.7***	***0.62***
3-009	0	0.00048
3-010	0	0.00023
3-011	0	7.13e-06
3-012	0	0.00018
3-013	0	4.12e-05
3-014	0	5.82e-05
3-015	0.039	NA
3-016	0.028	0.022
3-017	0	0.0001
3-018	0.0011	0.0018
3-019	0	0.00021
3-020	0	0.00027
3-021	0	0.0013
3-022	***2.27***	***1.2***
3-023	0.015	2.79e-05
3-024	***0.13***	NA
3-025	0	1.61e-05
3-026	0.00025	NA
3-027	0	1.84e-05
3-028	0	1.66e-05
3-029	***1.1***	***0.81***
3-030	0	0.00018
3-031	0.0047	NA
3-032	0.0032	0.0042
3-033	0	0.00019
3-034	0	5.09e-05
3-035	0	0.00054
3-037	0	8.26e-05
3-038	0.0010	0.018
3-039	0	4.02e-05
3-040	0	7.74e-05
3-041	0	5.6e-05
3-042	0	4.42e-05

[1] Expression measured as the sum of base coverage of the first exon of *EVI1* per kb transcript and one million mapped reads (log2 BPKM).

[2] Expression of the first exon of *EVI1* normalized for the *HMBS* housekeeping gene measured by quantitative RT-PCR as previously described (refs).

Table SXVIII. Clonal evolution of longitudinal AML samples from four patients

	Primary	Relapse
**Case**	**2-015**	**2-018**
**Karyotype**	46 XX	no metaphases
**Fusion genes**	none	none
**NPM1**	ENST00000517671.3:c.860_863dup	ENST00000517671.3:c.860_863dup
**DNMT3A**	ENST00000321117.7:c.1583_1603dup	wt
**FLT3-ITD**	1824-1874	1824-1874
**TET2**	ENST00000380013.6:c.4317dup	wt
**WHO**	NPM1mut AML	NPM1mut AML
**Genomic**	NPM1mut AML	NPM1mut AML
**2017 ELN Risk Group**	Intermediate	Intermediate

**Case**	**2-031**	**2-032**
**Karyotype**	46 XY	46 XY
**Fusion genes**	NUP98--NSD1 chr11:3753316 chr5:177235821	NUP98--NSD1 chr11:3753316 chr5:177235821; CLEC12A--CHMP1A
**FLT3-ITD**	1861-1874	1861-1874
**WT1**	wt	ENST00000332351.5:c.1140dup; ENST00000332351.5:c.1138C>G
**WHO**	AML NOS	AML NOS
**Genomic**	AML NOS with drivers	AML NOS with drivers
**2017 ELN Risk Group**	Intermediate	Intermediate

**Case**	**2-036**	**2-040**
**Karyotype**	46 XX	46 XX, t(1;16)(p22;q12)
**Fusion genes**	NCOR2-UBC; TPM4--KLF2	none
**DNMT3A**	ENST00000321117.7:c.2645G>C	ENST00000321117.7:c.2645G>C
**FLT3-ITD**	1812-1868; 1813-1909; 1875-1891	1843-1919
**CEBPA**	ENST00000498907.2:c.332C>G; ENST00000498907.2:c.332_339del ENST00000300305.5:c.167T>C	wt
**RUNX1**		ENST00000300305.5:c.167T>C
**WT1**	wt	ENST00000332351.5:c.1106dup; ENST00000332351.5:c.1105C>G
**WHO**	AML NOS	AML NOS
**Genomic**	Chromatin/Splicing	Chromatin/Splicing
**2017 ELN Risk Group**	Adverse	Adverse

**Case**	**3-021**	**3-038 (presumed therapy-related AML)**
**Karyotype**	46 XX	48 XX, -7, +10, +19, +21
**Fusion genes**	TPM4--KLF2; PIM3--SCO2; OAZ1--KLF2; GUK1--ARF1	C15orf57--CBX3; NCOR2—UBC
**NPM1**	ENST00000517671.3:c.860_863dup	wt
**DNMT3A**	ENST00000321117.7:c.2604del	ENST00000321117.7:c.2340_2343del
**IDH2**	ENST00000330062.5:c.419G>A	ENST00000330062.5:c.419G>A
**WHO**	NPM1mut AML	AML NOS
**Genomic**	NPM1mut AML	AML with drivers
**2017 ELN Risk Group**	Favorable	Intermediate

HAMLET

Acute Myeloid Leukemia mRNA-seq Analysis Pipeline

Analysis Report

Sample '102581-03' - Run 'hamlet-c9d653a2'

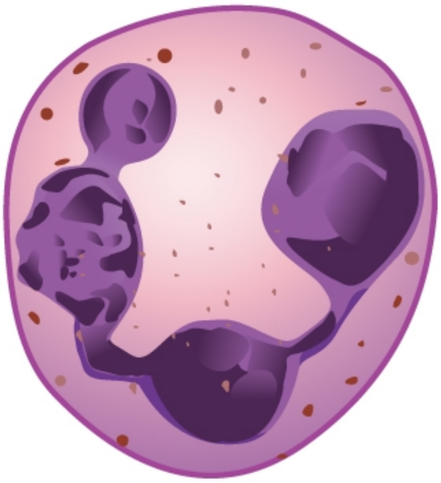

Version 0.1.0-26d901da*

Generated on Wednesday, 09 May 2018 at 15:07

Department of Hematology
Leiden University Medical Center

Table of Contents

Table of Contents 1
Basic Information 2
Results Overview 3
Sequencing Results 4
Alignment Statistics 5
Annotation Statistics 6
Variant Calling Results 7
- TP53 variants 7
- KIT variants 8
- NPM1 variants 9
- IDH2 variants 11
- ASXL1 variants 12
- DNMT3A variants 13
- WT1 variants 16
- NRAS variants 17
- RUNX1 variants 18
- IDH1 variants 19
- TET2 variants 20
- FLT3 variants 21
- MECOM variants 23
- CEBPA variants 25

Fusion Detection Results 26
ITD/PTD Detection Results 27
- FLT3 ITD 27
- KMT2A PTD 27

Overexpression Analysis Results 28
About 29

Basic Information

Table 1 | Pipeline and sample details

Sample name	102581-03
Read group count	3
Run name	hamlet-c9d653a2
Pipeline version	0.1.0-26d901da*

Table 2 | Genes of interest defined for analysis

No.	Gene symbol	Gene ID	Transcript IDs of interest
1.	ASXL1	ENSG00000171456	ENST00000375687
2.	CEBPA	ENSG00000245848	ENST00000498907
3.	DNMT3A	ENSG00000119772	ENST00000321117
4.	FLT3	ENSG00000122025	ENST00000241453
5.	IDH1	ENSG00000138413	ENST00000345146
6.	IDH2	ENSG00000182054	ENST00000330062
7.	KIT	ENSG00000157404	ENST00000288135
8.	NPM1	ENSG00000181163	ENST00000517671
9.	NRAS	ENSG00000213281	ENST00000369535
10.	RUNX1	ENSG00000159216	ENST00000300305
11.	TET2	ENSG00000168769	ENST00000380013
12.	TP53	ENSG00000141510	ENST00000269305
13.	WT1	ENSG00000184937	ENST00000332351
14.	MECOM	ENSG00000085276	ENST00000464456, ENST00000494292

Results Overview

Table 3 | High-impact variants in genes of interest

Gene	HGVS description	Known database identifiers
DNMT3A	ENST00000321117.7:c.2644C>T	rs377577594, COSM1166704, COSM1583136, COSM53042, COSM87001
NPM1	ENST00000517671.3:c.860_861insCTGC	COSM20866, COSM20863, COSM1658759, COSM28937
TET2	ENST00000380013.6:c.3989C>A	NA
	ENST00000380013.6:c.5838_5839insA	COSM1426222

Table 4 | Top 5 detected fusion genes by supporting reads

Fusion name	Fusion type	Junction reads count	Spanning fragments count
TPM4--KLF2	ONLY_REF_SPLICE	6	0

Table 5 | Detected reciprocal soft clip events

Gene	Count
FLT3	2
KMT2A	0

Table 6 | Exon base count ratio over divisor gene

Exon	Divisor gene	Ratio	Above threshold
MECOM:169146722-169147734	HMBS	0	no

Sequencing Results

Table 7 | Combined reads statistics

Metric	R1	R2	R1 + R2
# Raw reads	53,864,742	53,864,742	107,729,484
# Processed reads	52,731,725	52,731,725	105,463,450
% Retained reads	n/a	n/a	97.90%

Table 8 | Per read group statistics

Read Group	Raw				Processed			
	R1		R2		R1		R2	
	#Reads	%GC	#Reads	%GC	#Reads	%GC	#Reads	%GC
rg_1	14,940,329	48	14,940,329	48	14,641,869	48	14,641,869	48
rg_2	31,597,414	48	31,597,414	48	30,892,774	48	30,892,774	48
rg_3	7,326,999	48	7,326,999	48	7,197,082	48	7,197,082	48

Alignment Statistics

Table 9 | Alignment count statistics

Metric	Count	% of total	% of aligned
Total reads	105,485,637	100%	105.10%
Reads aligned	100,369,359	95.15%	100%
Reads aligned properly	99,769,438	94.58%	99.40%
Total bases	13,065,206,475	100%	107.37%
Bases aligned	12,168,410,518	93.14%	100%

Table 10 | Insert size statistics

Median	169
Median absolute deviation	36
Maximum	249,095,288
Minimum	1

Table 11 | Other alignment statistics

Strand balance	0.4999
Mismatch rate	1.97e-03
Indel rate	1.87e-04

Annotation Statistics

Table 12 | Base-level annotation statistics

Metric	Count	% of aligned
Total aligned	12,168,410,518	100%
Aligned to mRNA (CDS + UTR)	9,209,245,449	75.68%
Aligned to CDS	5,416,844,873	44.52%
Aligned to UTR	3,792,400,576	31.17%
Aligned to introns	2,451,281,466	20.14%
Aligned to intergenic regions	505,889,353	4.16%
Aligned to rRNA	2,080,252	0.02%

Variant Calling Results

Table 13 | Variant calling results by type

Type	Count	% of total
Total	219,462	100%
SNV	196,329	89.46%
Insertion	15,023	6.85%
Deletion	8,110	3.70%

TP53 variants

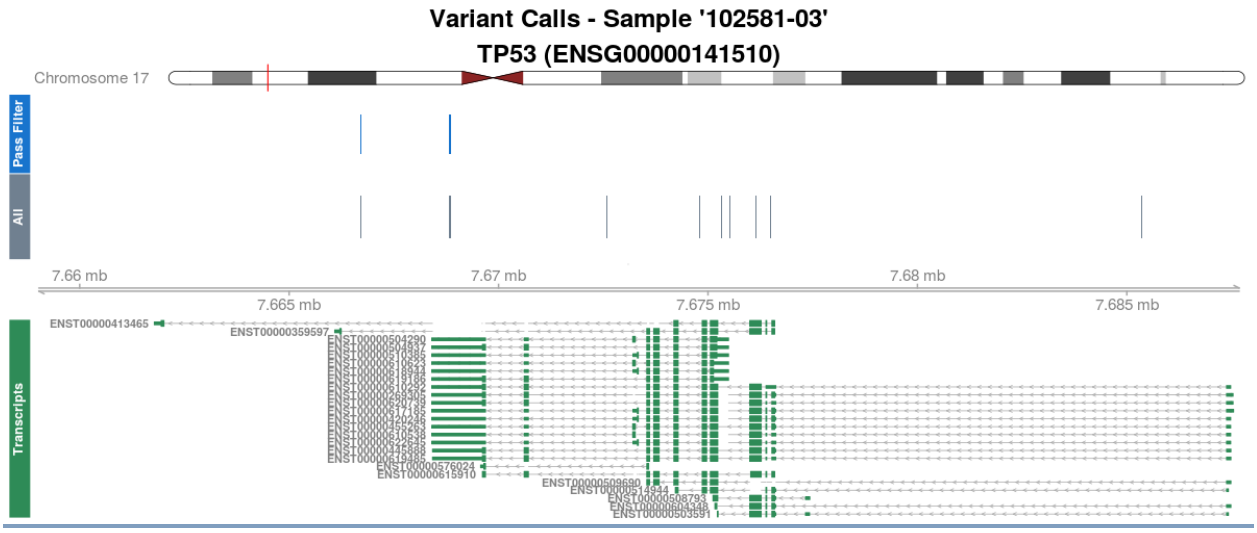

Figure 1 | TP53 variant calling results

Table 14 | Exon statistics of transcript ENST00000269305 (TP53)

Index	Location (chr17)		Coverage			Fraction covered at least		
	Start	End	Median	Avg	Stdev	10x	20x	30x
1	7,668,401	7,669,690	525	478.1	151.2	1.0	1.0	1.0
2	7,670,608	7,670,715	386	381.4	16.6	1.0	1.0	1.0
3	7,673,534	7,673,608	446	444.8	14.3	1.0	1.0	1.0
4	7,673,700	7,673,837	446	442.2	22.5	1.0	1.0	1.0
5	7,674,180	7,674,290	419	412.4	19.8	1.0	1.0	1.0
6	7,674,858	7,674,971	369	371.5	25.5	1.0	1.0	1.0
7	7,675,052	7,675,236	368	367.4	23.1	1.0	1.0	1.0
8	7,675,993	7,676,272	413	406.1	39.7	1.0	1.0	1.0
9	7,676,381	7,676,403	422	417.0	14.2	1.0	1.0	1.0
10	7,676,520	7,676,622	369	370.0	15.1	1.0	1.0	1.0
11	7,687,376	7,687,538	156	166.0	119.7	1.0	1.0	1.0

KIT variants

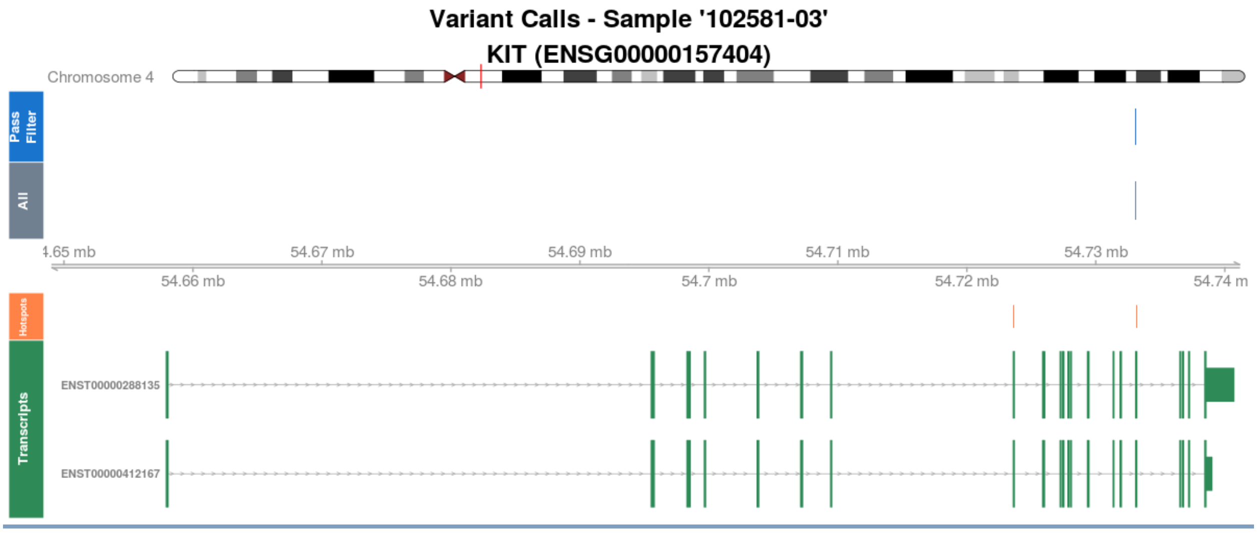

Figure 2 | KIT variant calling results

Table 15 | Exon statistics of transcript ENST00000288135 (KIT)

Index	Location (chr4)		Coverage			Fraction covered at least		
	Start	End	Median	Avg	Stdev	10x	20x	30x
1	54,657,917	54,658,081	15	13.7	10.0	0.6	0.4	0.0
2	54,695,511	54,695,781	20	19.8	6.2	1.0	0.5	0.1
3	54,698,283	54,698,565	19	19.2	3.4	1.0	0.5	0.0
4	54,699,629	54,699,766	10	11.8	6.0	0.5	0.1	0.0
5	54,703,723	54,703,892	12	11.2	2.2	0.7	0.0	0.0
6	54,707,097	54,707,287	15	15.1	1.3	1.0	0.0	0.0
7	54,709,423	54,709,539	13	12.1	1.6	1.0	0.0	0.0
8	54,723,583	54,723,698	22	21.2	3.3	1.0	0.8	0.0
9	54,725,856	54,726,050	25	25.0	6.2	1.0	0.8	0.2
10	54,727,217	54,727,324	26	25.0	3.3	1.0	0.9	0.0
11	54,727,415	54,727,542	14	13.9	2.1	1.0	0.0	0.0
12	54,727,822	54,727,927	13	13.5	2.0	1.0	0.0	0.0
13	54,728,010	54,728,121	18	18.6	2.8	1.0	0.5	0.0
14	54,729,334	54,729,485	21	21.6	5.2	1.0	0.6	0.0
15	54,731,327	54,731,419	32	32.0	1.4	1.0	1.0	1.0
16	54,731,870	54,731,998	22	22.8	3.0	1.0	0.9	0.0
17	54,733,069	54,733,192	30	28.1	3.8	1.0	1.0	0.5
18	54,736,497	54,736,609	23	24.2	5.5	1.0	0.7	0.3
19	54,736,720	54,736,820	38	37.2	3.0	1.0	1.0	1.0
20	54,737,174	54,737,280	42	42.8	4.1	1.0	1.0	1.0
21	54,738,428	54,740,715	43	45.5	16.7	1.0	0.9	0.9

NPM1 variants

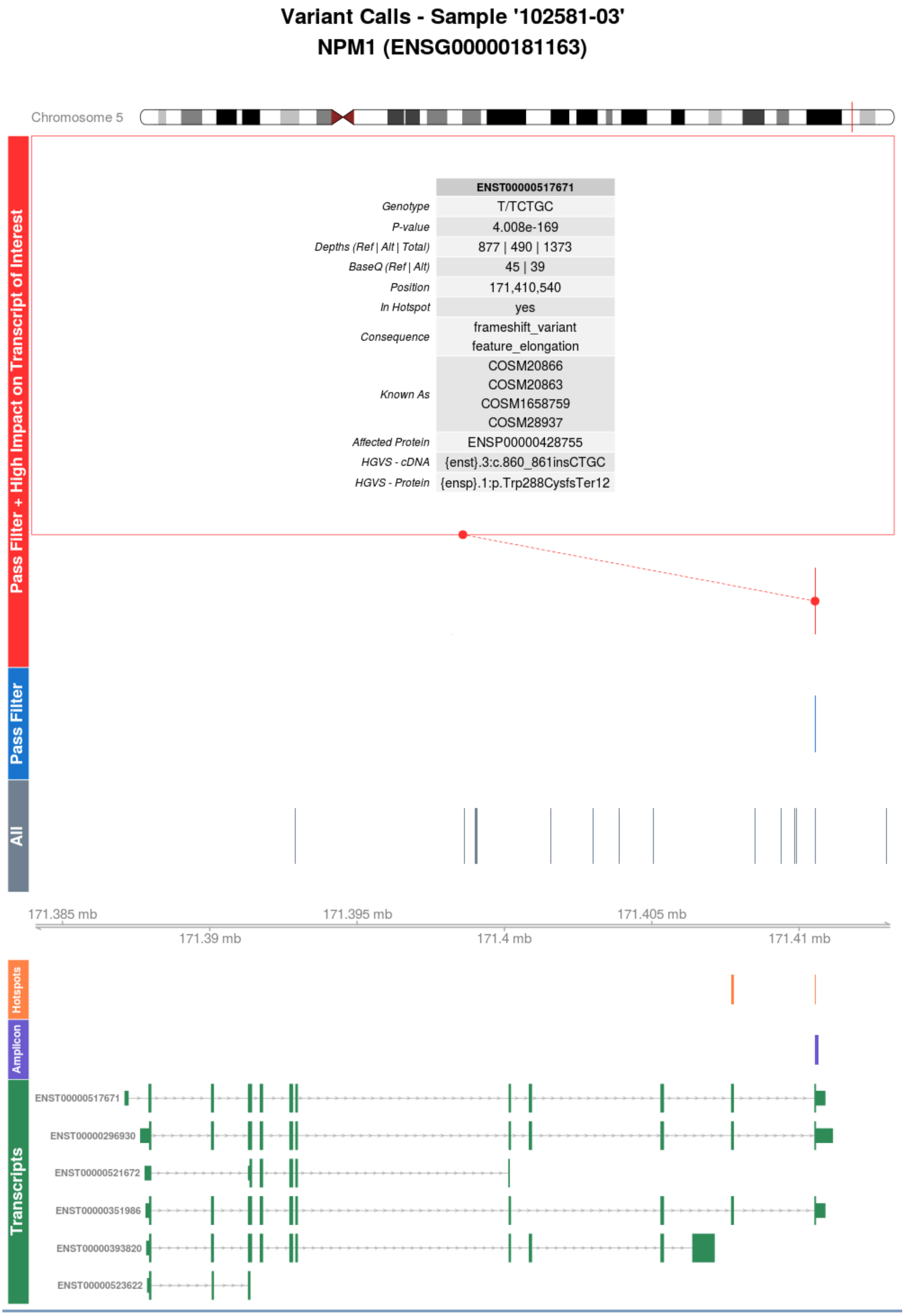

Figure 3 | NPM1 variant calling results

Table 16 | Exon statistics of transcript ENST00000517671 (NPM1)

Index	Location (chr5)		Coverage			Fraction covered at least		
	Start	End	Median	Avg	Stdev	10x	20x	30x
1	171,387,115	171,387,234	5	5.0	0.0	0.0	0.0	0.0
2	171,387,932	171,388,006	4,134	4,020.5	462.6	1.0	1.0	1.0
3	171,390,050	171,390,130	3,779	3,704.7	255.2	1.0	1.0	1.0
4	171,391,304	171,391,424	3,688	3,679.2	449.1	1.0	1.0	1.0
5	171,391,705	171,391,799	2,405	2,339.0	395.2	1.0	1.0	1.0
6	171,392,709	171,392,816	1,138	1,173.1	354.7	1.0	1.0	1.0
7	171,392,913	171,392,978	760	773.3	48.4	1.0	1.0	1.0
8	171,400,152	171,400,210	1,344	1,275.5	249.2	1.0	1.0	1.0
9	171,400,838	171,400,925	2,586	2,919.4	891.8	1.0	1.0	1.0
10	171,405,301	171,405,403	3,961	3,782.1	490.0	1.0	1.0	1.0
11	171,407,699	171,407,774	1,589	1,549.1	119.7	1.0	1.0	1.0
12	171,410,526	171,410,883	3,471	3,022.0	1,259.9	1.0	1.0	1.0

IDH2 variants

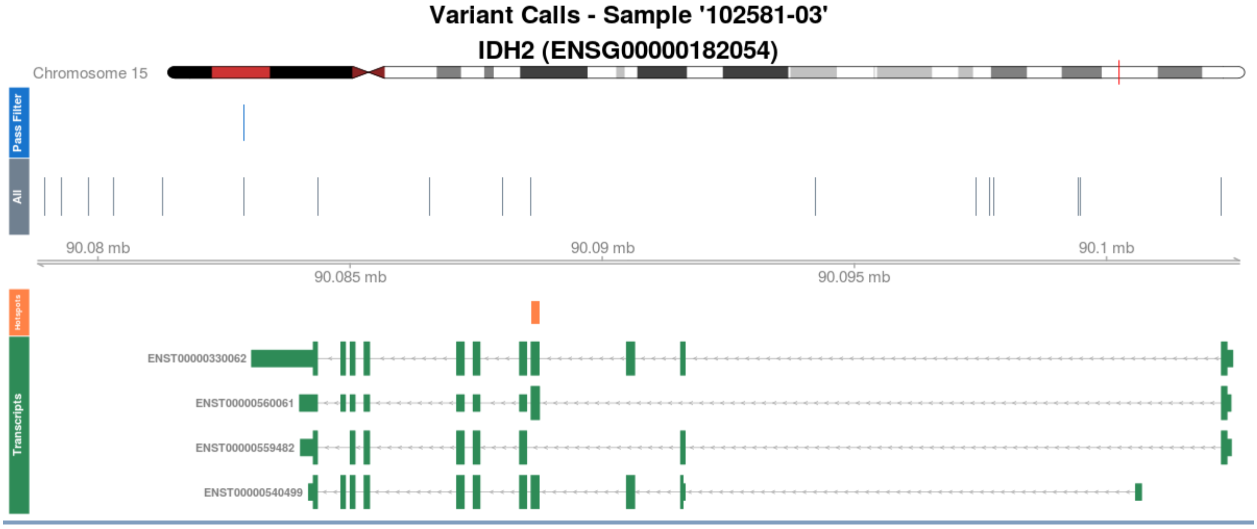

Figure 4 | IDH2 variant calling results

Table 17 | Exon statistics of transcript ENST00000330062 (IDH2)

Index	Location (chr15)		Coverage			Fraction covered at least		
	Start	End	Median	Avg	Stdev	10x	20x	30x
1	90,083,044	90,084,353	183	284.5	309.8	1.0	0.9	0.9
2	90,084,815	90,084,908	1,573	1,571.4	54.3	1.0	1.0	1.0
3	90,085,000	90,085,098	1,298	1,291.5	67.0	1.0	1.0	1.0
4	90,085,274	90,085,387	1,094	1,091.7	41.4	1.0	1.0	1.0
5	90,087,111	90,087,263	1,119	1,110.4	48.8	1.0	1.0	1.0
6	90,087,438	90,087,575	1,057	1,060.2	49.9	1.0	1.0	1.0
7	90,088,358	90,088,502	1,079	1,085.6	103.0	1.0	1.0	1.0
8	90,088,586	90,088,747	1,063	1,059.4	106.7	1.0	1.0	1.0
9	90,090,478	90,090,644	850	860.4	80.9	1.0	1.0	1.0
10	90,091,552	90,091,644	671	646.8	59.1	1.0	1.0	1.0
11	90,102,275	90,102,504	421	355.9	206.3	1.0	0.9	0.9

ASXL1 variants

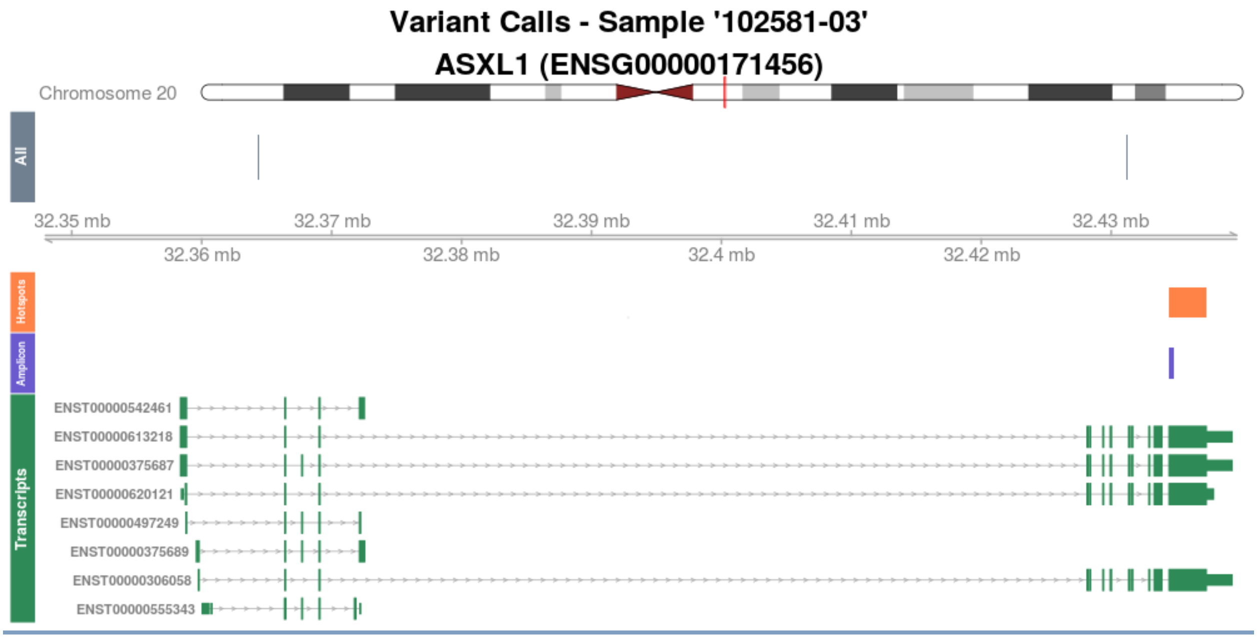

Figure 5 | ASXL1 variant calling results

Table 18 | Exon statistics of transcript ENST00000375687 (ASXL1)

Index	Location (chr20)		Coverage			Fraction covered at least		
	Start	End	Median	Avg	Stdev	10x	20x	30x
1	32,358,351	32,358,832	23	30.4	17.1	1.0	0.7	0.4
2	32,366,383	32,366,466	161	160.2	13.6	1.0	1.0	1.0
3	32,367,726	32,367,729	99	99.0	0.0	1.0	1.0	1.0
4	32,369,014	32,369,123	176	176.2	9.6	1.0	1.0	1.0
5	32,428,127	32,428,248	186	184.8	14.1	1.0	1.0	1.0
6	32,428,324	32,428,422	182	184.1	8.9	1.0	1.0	1.0
7	32,429,337	32,429,431	160	158.8	6.0	1.0	1.0	1.0
8	32,429,900	32,430,053	160	160.0	9.0	1.0	1.0	1.0
9	32,431,320	32,431,484	209	209.5	8.0	1.0	1.0	1.0
10	32,431,582	32,431,679	242	240.9	13.2	1.0	1.0	1.0
11	32,432,879	32,432,985	238	240.4	10.0	1.0	1.0	1.0
12	32,433,283	32,433,917	253	238.8	45.5	1.0	1.0	1.0
13	32,434,431	32,439,319	244	238.6	93.7	1.0	1.0	1.0

DNMT3A variants

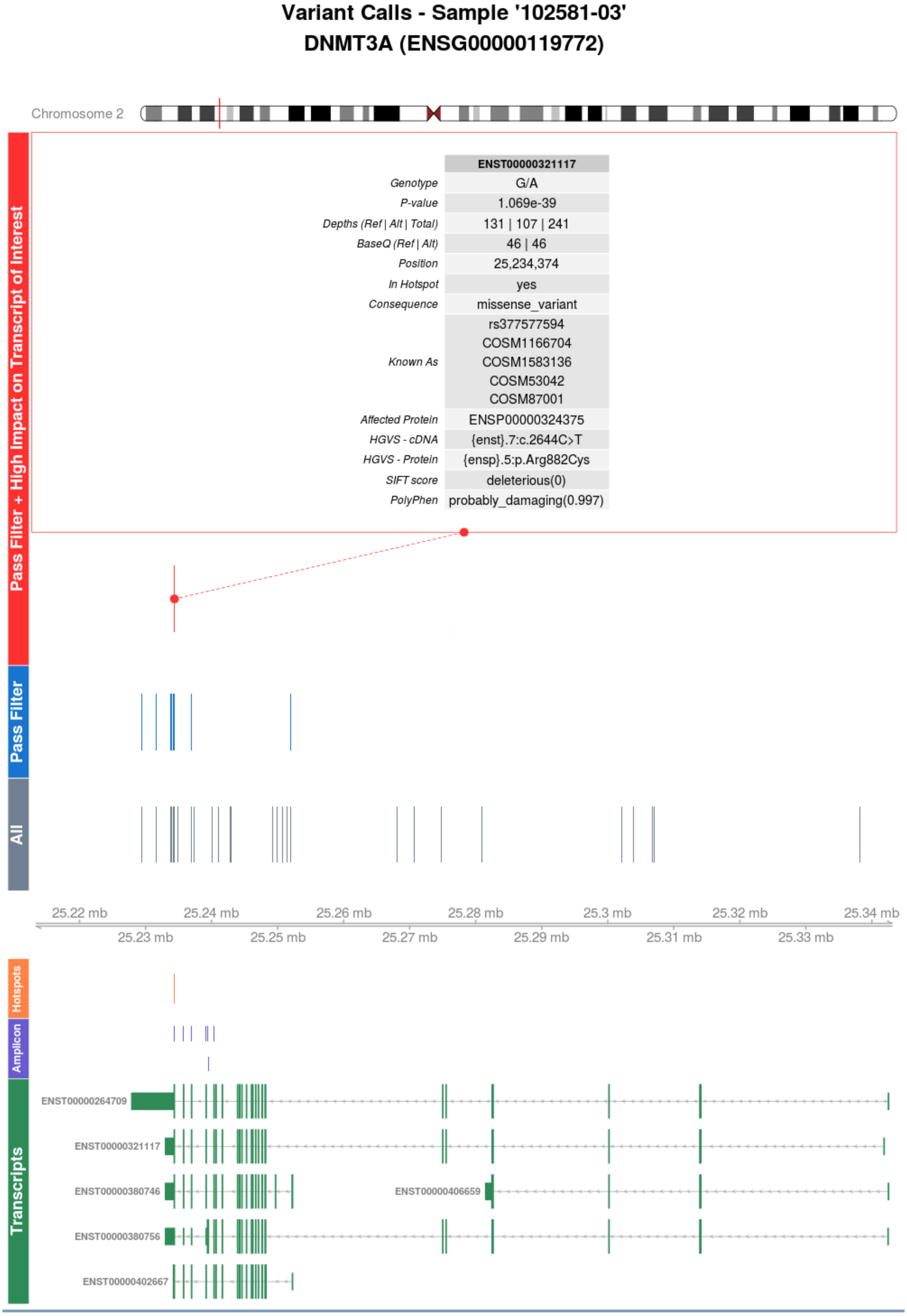

Figure 6 | DNMT3A variant calling results

Table 19 | Exon statistics of transcript ENST00000321117 (DNMT3A)

Index	Location (chr2)		Coverage			Fraction covered at least		
	Start	End	Median	Avg	Stdev	10x	20x	30x
1	25,232,975	25,234,420	325	309.1	124.7	1.0	1.0	1.0
2	25,235,706	25,235,825	374	360.0	35.8	1.0	1.0	1.0
3	25,236,935	25,237,005	407	409.0	17.9	1.0	1.0	1.0
4	25,239,129	25,239,215	323	319.3	25.4	1.0	1.0	1.0
5	25,240,301	25,240,450	272	269.3	13.1	1.0	1.0	1.0
6	25,240,639	25,240,730	296	294.4	8.5	1.0	1.0	1.0
7	25,241,561	25,241,707	340	339.4	16.4	1.0	1.0	1.0
8	25,243,897	25,243,982	313	307.9	20.4	1.0	1.0	1.0
9	25,244,154	25,244,338	234	236.5	13.2	1.0	1.0	1.0
10	25,244,539	25,244,652	282	287.8	24.7	1.0	1.0	1.0
11	25,245,252	25,245,332	309	307.1	11.1	1.0	1.0	1.0
12	25,246,019	25,246,064	244	243.0	7.6	1.0	1.0	1.0
13	25,246,159	25,246,309	264	265.5	28.9	1.0	1.0	1.0
14	25,246,619	25,246,776	271	261.4	22.1	1.0	1.0	1.0
15	25,247,050	25,247,158	314	311.0	17.9	1.0	1.0	1.0
16	25,247,590	25,247,749	166	192.7	53.7	1.0	1.0	1.0
17	25,248,036	25,248,252	278	269.8	53.4	1.0	1.0	1.0
18	25,274,940	25,275,087	50	49.4	4.4	1.0	1.0	1.0
19	25,275,499	25,275,543	38	37.7	2.2	1.0	1.0	1.0
20	25,282,440	25,282,711	74	85.2	30.0	1.0	1.0	1.0
21	25,300,138	25,300,243	120	119.6	5.7	1.0	1.0	1.0
22	25,313,912	25,314,161	88	98.2	19.8	1.0	1.0	1.0
23	25,341,825	25,341,885	46	47.1	7.7	1.0	1.0	1.0

WT1 variants

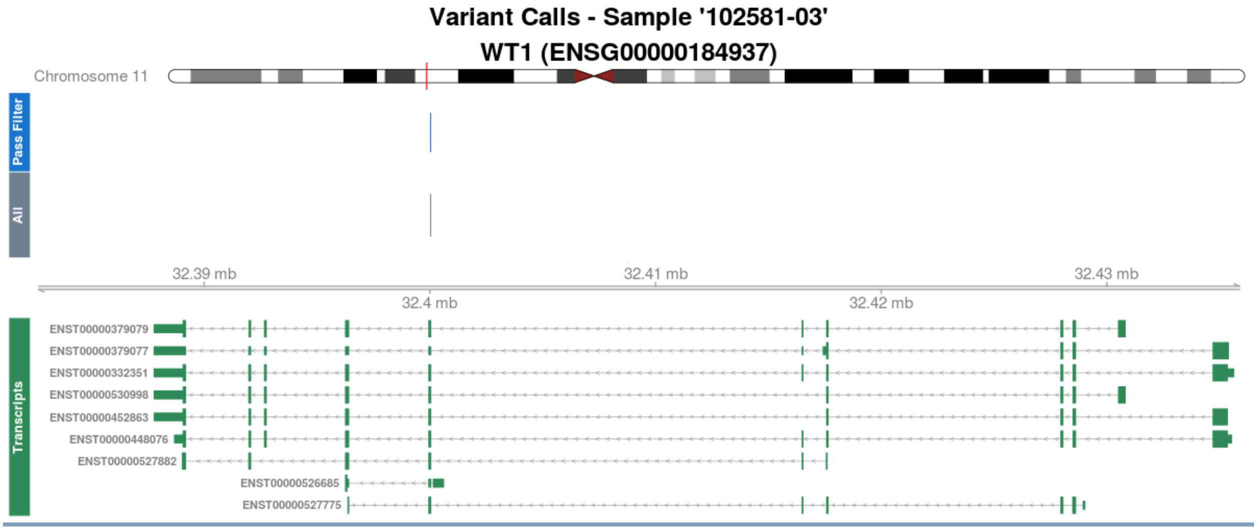

Figure 7 | WT1 variant calling results

Table 20 | Exon statistics of transcript ENST00000332351 (WT1)

Index	Location (chr11)		Coverage			Fraction covered at least		
	Start	End	Median	Avg	Stdev	10x	20x	30x
1	32,387,774	32,389,179	122	117.7	35.0	1.0	1.0	1.0
2	32,391,971	32,392,064	108	112.8	12.2	1.0	1.0	1.0
3	32,392,665	32,392,755	126	124.3	10.8	1.0	1.0	1.0
4	32,396,256	32,396,407	104	105.0	10.5	1.0	1.0	1.0
5	32,399,947	32,400,044	89	88.7	4.3	1.0	1.0	1.0
6	32,416,489	32,416,540	74	73.1	3.2	1.0	1.0	1.0
7	32,417,576	32,417,654	105	102.4	8.2	1.0	1.0	1.0
8	32,427,955	32,428,058	80	78.7	12.6	1.0	1.0	1.0
9	32,428,496	32,428,619	64	62.9	8.3	1.0	1.0	1.0
10	32,434,699	32,435,630	52	52.7	27.1	0.9	0.9	0.8

NRAS variants

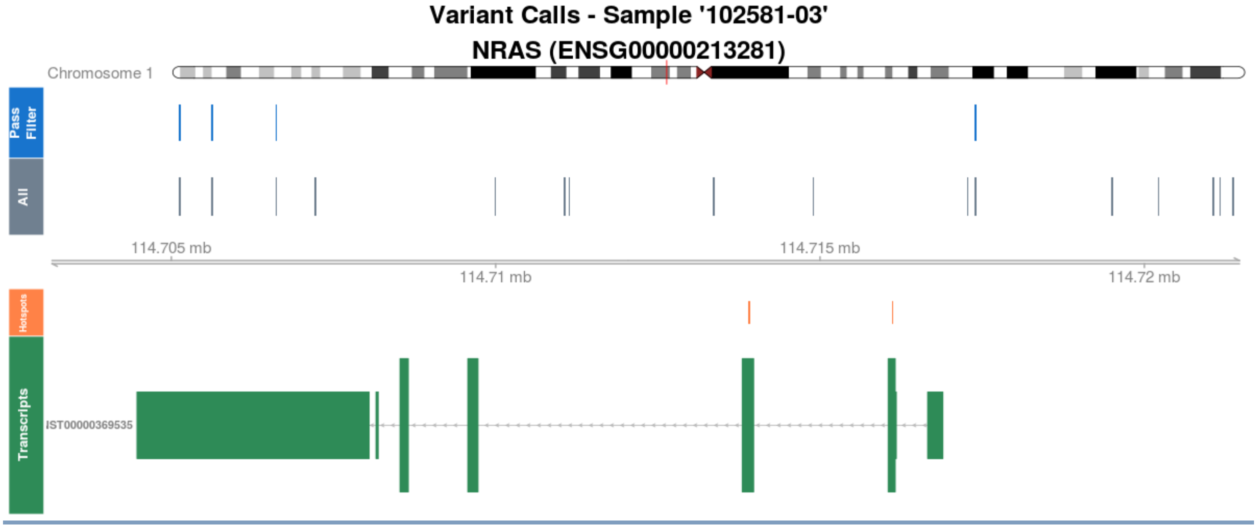

Figure 8 | NRAS variant calling results

Table 21 | Exon statistics of transcript ENST00000369535 (NRAS)

Index	Location (chr1)		Coverage			Fraction covered at least		
	Start	End	Median	Avg	Stdev	10x	20x	30x
1	114,704,468	114,708,050	206	211.3	58.5	1.0	1.0	1.0
2	114,708,153	114,708,192	180	177.7	9.2	1.0	1.0	1.0
3	114,708,530	114,708,654	247	222.0	42.1	1.0	1.0	1.0
4	114,709,568	114,709,728	233	234.8	23.1	1.0	1.0	1.0
5	114,713,799	114,713,978	196	195.2	10.2	1.0	1.0	1.0
6	114,716,049	114,716,177	170	169.0	6.6	1.0	1.0	1.0
7	114,716,657	114,716,894	5	43.4	54.5	0.4	0.4	0.4

RUNX1 variants

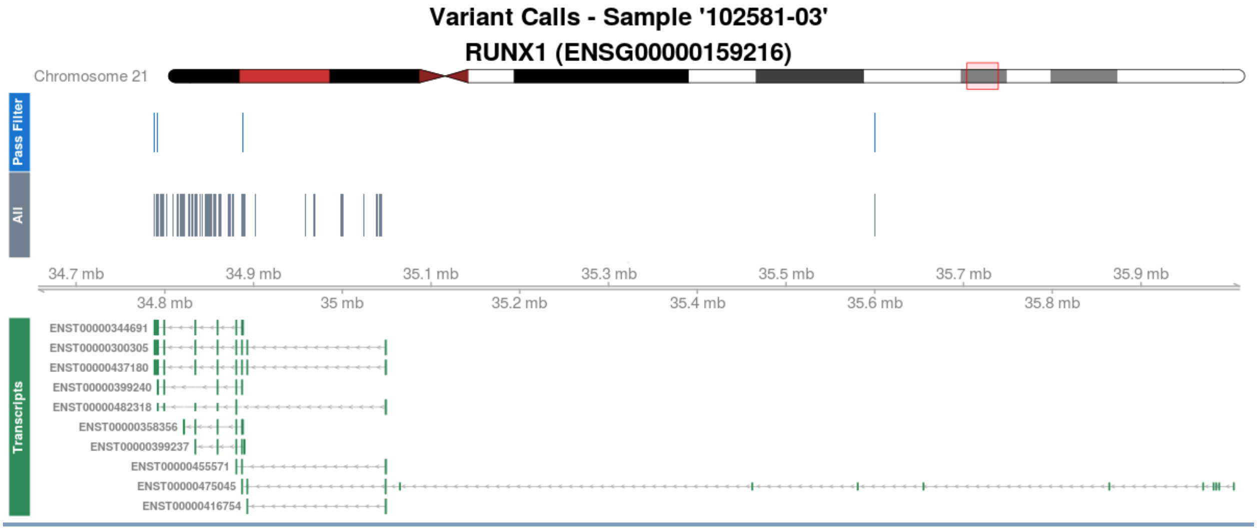

Figure 9 | RUNX1 variant calling results

Table 22 | Exon statistics of transcript ENST00000300305 (RUNX1)

Index	Location (chr21)		Coverage			Fraction covered at least		
	Start	End	Median	Avg	Stdev	10x	20x	30x
1	34,787,800	34,792,610	945	994.2	471.9	1.0	1.0	1.0
2	34,799,300	34,799,462	1,114	1,105.1	85.1	1.0	1.0	1.0
3	34,834,409	34,834,601	1,197	1,188.5	60.8	1.0	1.0	1.0
4	34,859,473	34,859,578	1,206	1,196.2	54.3	1.0	1.0	1.0
5	34,880,556	34,880,713	1,012	1,015.7	68.5	1.0	1.0	1.0
6	34,886,842	34,887,096	609	617.1	75.4	1.0	1.0	1.0
7	34,892,924	34,892,963	154	154.4	4.8	1.0	1.0	1.0
8	35,048,841	35,049,344	169	153.9	73.8	0.9	0.9	0.9

IDH1 variants

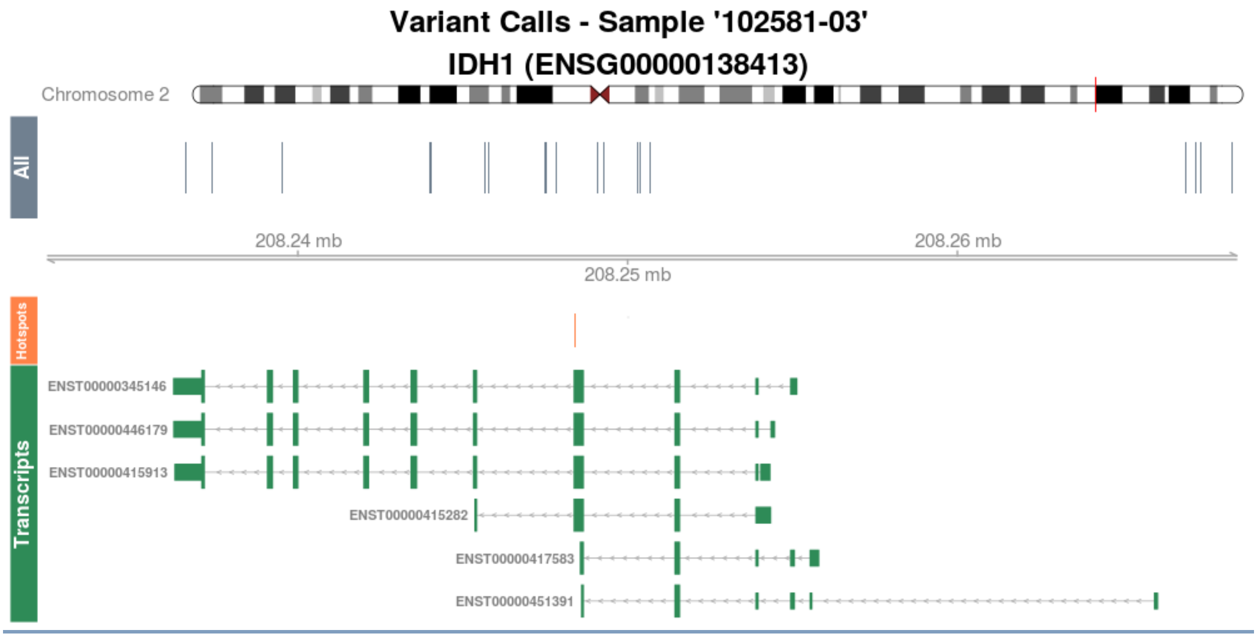

Figure 10 | IDH1 variant calling results

Table 23 | Exon statistics of transcript ENST00000345146 (IDH1)

Index	Location (chr2)		Coverage			Fraction covered at least		
	Start	End	Median	Avg	Stdev	10x	20x	30x
1	208,236,226	208,237,169	392	380.0	144.4	1.0	1.0	1.0
2	208,239,070	208,239,233	422	420.9	35.7	1.0	1.0	1.0
3	208,239,862	208,240,003	462	453.4	40.5	1.0	1.0	1.0
4	208,241,993	208,242,145	362	354.1	28.2	1.0	1.0	1.0
5	208,243,426	208,243,604	367	364.4	37.2	1.0	1.0	1.0
6	208,245,318	208,245,424	330	328.7	16.5	1.0	1.0	1.0
7	208,248,368	208,248,660	319	323.6	29.4	1.0	1.0	1.0
8	208,251,429	208,251,567	279	285.8	27.4	1.0	1.0	1.0
9	208,253,885	208,253,959	331	328.3	19.9	1.0	1.0	1.0
10	208,254,938	208,255,133	149	145.0	99.0	0.8	0.8	0.8

TET2 variants

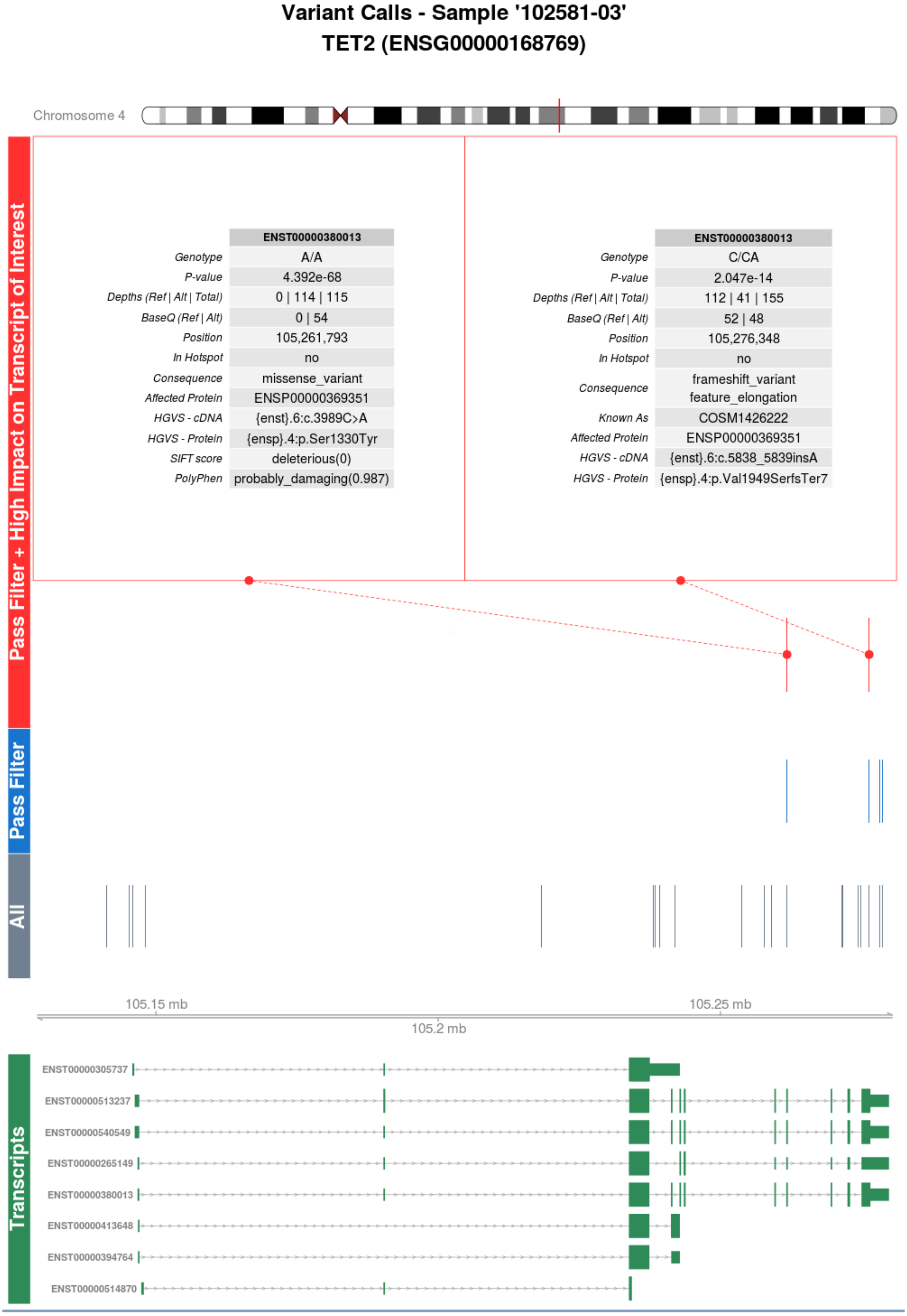

Figure 11 | TET2 variant calling results

Table 24 | Exon statistics of transcript ENST00000380013 (TET2)

Index	Location (chr4)		Coverage			Fraction covered at least		
	Start	End	Median	Avg	Stdev	10x	20x	30x
1	105,146,785	105,146,979	14	32.7	28.3	1.0	0.4	0.4
2	105,190,359	105,190,505	106	105.1	4.5	1.0	1.0	1.0
3	105,233,896	105,237,351	114	115.1	23.3	1.0	1.0	1.0
4	105,241,338	105,241,429	107	108.5	8.1	1.0	1.0	1.0
5	105,242,833	105,242,927	89	88.5	5.1	1.0	1.0	1.0
6	105,243,569	105,243,778	113	110.4	11.3	1.0	1.0	1.0
7	105,259,618	105,259,769	149	142.7	20.3	1.0	1.0	1.0
8	105,261,758	105,261,848	165	160.7	14.6	1.0	1.0	1.0
9	105,269,609	105,269,747	139	143.0	11.6	1.0	1.0	1.0
10	105,272,563	105,272,918	176	180.6	21.9	1.0	1.0	1.0
11	105,275,047	105,279,803	199	203.1	75.3	1.0	1.0	1.0

FLT3 variants

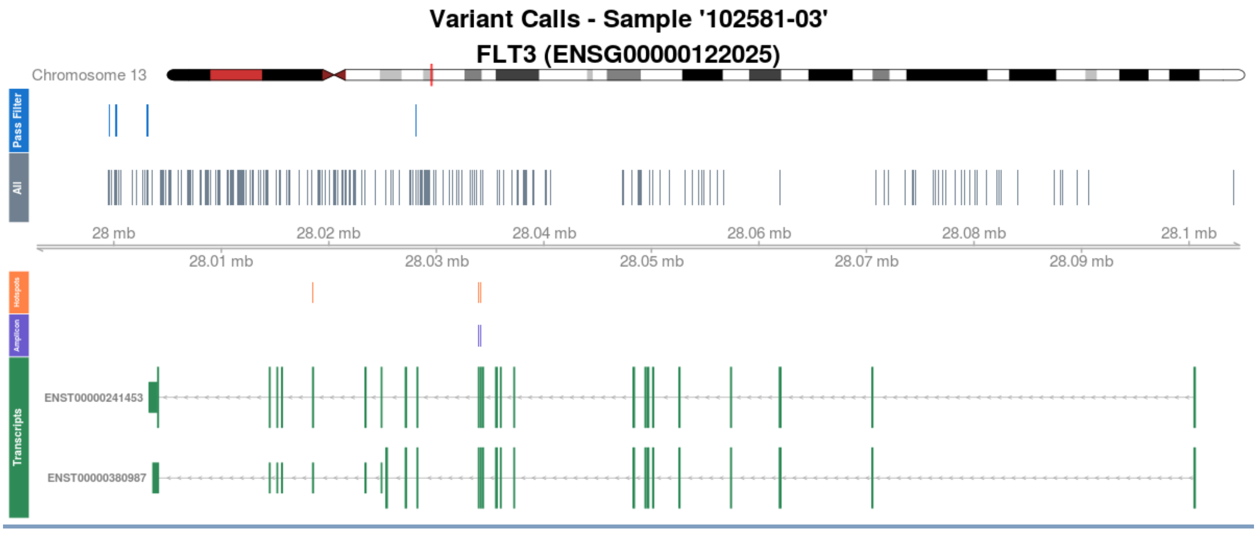

Figure 12 | FLT3 variant calling results

Table 25 | Exon statistics of transcript ENST00000241453 (FLT3)

Index	Location (chr13)		Coverage			Fraction covered at least		
	Start	End	Median	Avg	Stdev	10x	20x	30x
1	28,003,273	28,004,174	1,346	1,321.6	1,017.3	1.0	1.0	1.0
2	28,014,451	28,014,557	2,336	2,376.8	143.1	1.0	1.0	1.0
3	28,015,156	28,015,256	2,270	2,290.5	116.8	1.0	1.0	1.0
4	28,015,589	28,015,701	2,122	2,095.0	90.4	1.0	1.0	1.0
5	28,018,466	28,018,589	2,160	2,165.2	81.0	1.0	1.0	1.0
6	28,023,349	28,023,477	2,250	2,244.5	114.7	1.0	1.0	1.0
7	28,024,860	28,024,943	2,386	2,326.8	219.1	1.0	1.0	1.0
8	28,027,087	28,027,241	2,095	2,156.9	237.5	1.0	1.0	1.0
9	28,028,177	28,028,288	1,818	1,847.8	93.3	1.0	1.0	1.0
10	28,033,886	28,033,991	1,977	1,954.4	90.6	1.0	1.0	1.0
11	28,034,081	28,034,214	1,993	1,968.4	77.1	1.0	1.0	1.0
12	28,034,300	28,034,407	2,120	2,049.3	159.9	1.0	1.0	1.0
13	28,035,494	28,035,673	2,173	2,162.1	104.6	1.0	1.0	1.0
14	28,035,934	28,036,043	2,115	2,088.4	159.2	1.0	1.0	1.0
15	28,037,184	28,037,288	1,907	1,898.8	102.0	1.0	1.0	1.0
16	28,048,274	28,048,443	1,706	1,697.2	77.5	1.0	1.0	1.0
17	28,049,383	28,049,537	1,788	1,765.3	160.4	1.0	1.0	1.0
18	28,049,634	28,049,774	1,537	1,544.6	87.6	1.0	1.0	1.0
19	28,050,094	28,050,222	1,252	1,301.4	113.5	1.0	1.0	1.0
20	28,052,544	28,052,674	1,150	1,133.0	53.8	1.0	1.0	1.0
21	28,057,346	28,057,462	1,153	1,148.0	74.2	1.0	1.0	1.0
22	28,061,866	28,062,069	1,113	1,081.5	104.1	1.0	1.0	1.0
23	28,070,490	28,070,612	804	800.4	36.5	1.0	1.0	1.0
24	28,100,467	28,100,592	507	437.4	258.1	1.0	1.0	1.0

MECOM variants

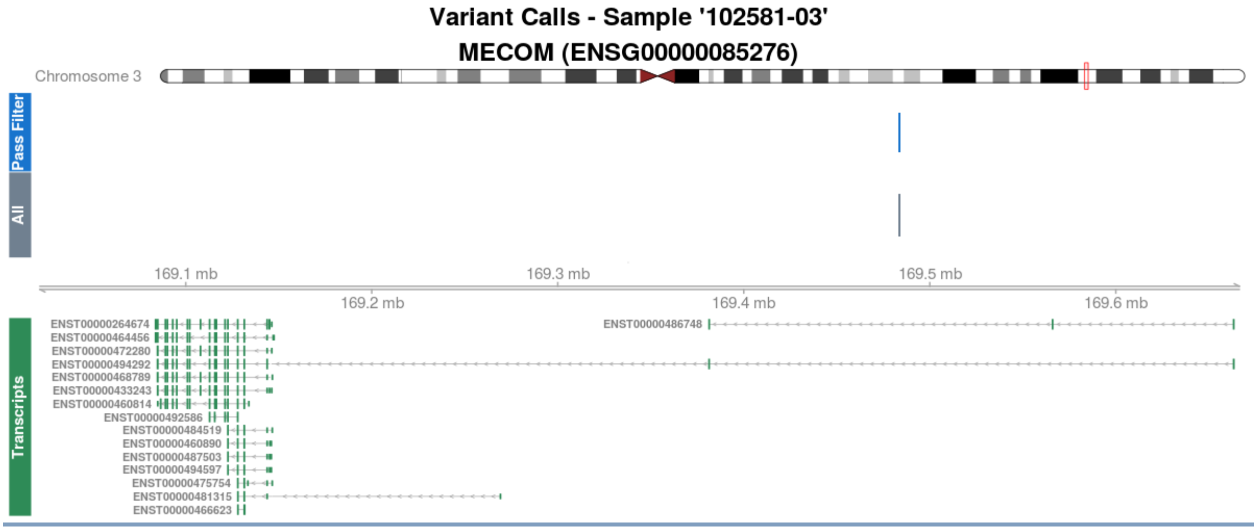

Figure 13 | MECOM variant calling results

Table 26 | Exon statistics of transcript ENST00000464456 (MECOM)

Index	Location (chr3)		Coverage			Fraction covered at least		
	Start	End	Median	Avg	Stdev	10x	20x	30x
1	169,083,506	169,085,043	0	0.0	0.0	0.0	0.0	0.0
2	169,088,999	169,089,183	0	0.0	0.0	0.0	0.0	0.0
3	169,089,999	169,090,236	0	0.0	0.0	0.0	0.0	0.0
4	169,092,957	169,093,102	0	0.0	0.0	0.0	0.0	0.0
5	169,095,075	169,095,245	0	0.0	0.0	0.0	0.0	0.0
6	169,100,884	169,100,962	0	0.0	0.0	0.0	0.0	0.0
7	169,102,059	169,102,226	0	0.0	0.0	0.0	0.0	0.0
8	169,112,786	169,112,874	0	0.0	0.0	0.0	0.0	0.0
9	169,115,382	169,116,739	0	0.0	0.0	0.0	0.0	0.0
10	169,121,055	169,121,209	0	0.0	0.0	0.0	0.0	0.0
11	169,122,579	169,122,727	0	0.0	0.0	0.0	0.0	0.0
12	169,127,843	169,128,060	0	0.0	0.0	0.0	0.0	0.0
13	169,131,428	169,131,531	0	0.0	0.0	0.0	0.0	0.0
14	169,143,697	169,143,832	0	0.0	0.0	0.0	0.0	0.0
15	169,146,722	169,147,734	0	0.0	0.0	0.0	0.0	0.0

Table 27 | Exon statistics of transcript ENST00000494292 (MECOM)

Index	Location (chr3)		Coverage			Fraction covered at least		
	Start	End	Median	Avg	Stdev	10x	20x	30x
1	169,084,760	169,085,043	0	0.0	0.0	0.0	0.0	0.0
2	169,088,999	169,089,183	0	0.0	0.0	0.0	0.0	0.0
3	169,089,999	169,090,236	0	0.0	0.0	0.0	0.0	0.0
4	169,092,957	169,093,102	0	0.0	0.0	0.0	0.0	0.0
5	169,095,075	169,095,245	0	0.0	0.0	0.0	0.0	0.0
6	169,100,884	169,100,962	0	0.0	0.0	0.0	0.0	0.0
7	169,102,059	169,102,226	0	0.0	0.0	0.0	0.0	0.0
8	169,112,786	169,112,874	0	0.0	0.0	0.0	0.0	0.0
9	169,115,382	169,116,739	0	0.0	0.0	0.0	0.0	0.0
10	169,121,055	169,121,209	0	0.0	0.0	0.0	0.0	0.0
11	169,122,579	169,122,727	0	0.0	0.0	0.0	0.0	0.0
12	169,127,843	169,128,060	0	0.0	0.0	0.0	0.0	0.0
13	169,131,428	169,131,531	0	0.0	0.0	0.0	0.0	0.0
14	169,143,697	169,143,832	0	0.0	0.0	0.0	0.0	0.0
15	169,381,186	169,381,524	0	0.0	0.0	0.0	0.0	0.0
16	169,663,335	169,663,470	2	1.8	0.5	0.0	0.0	0.0

CEBPA variants

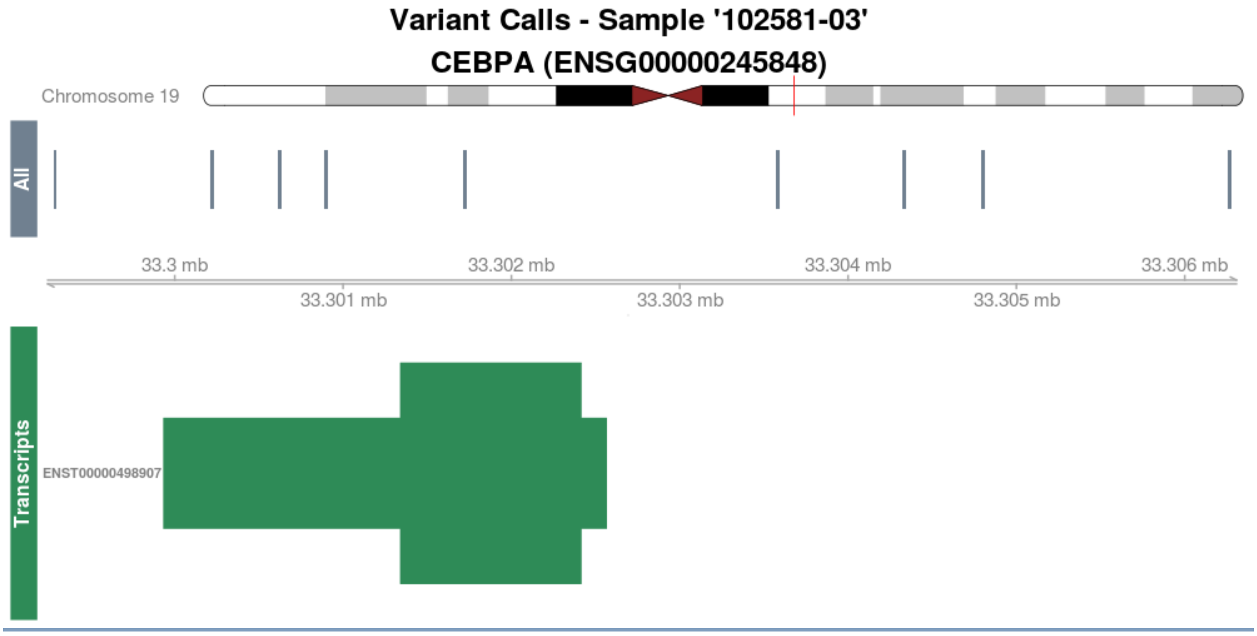

Figure 14 | CEBPA variant calling results

Table 28 | Exon statistics of transcript ENST00000498907 (CEBPA)

Index	Location (chr19)		Coverage			Fraction covered at least		
	Start	End	Median	Avg	Stdev	10x	20x	30x
1	33,299,933	33,302,564	1,464	1,559.4	882.0	1.0	1.0	1.0

Fusion Detection Results

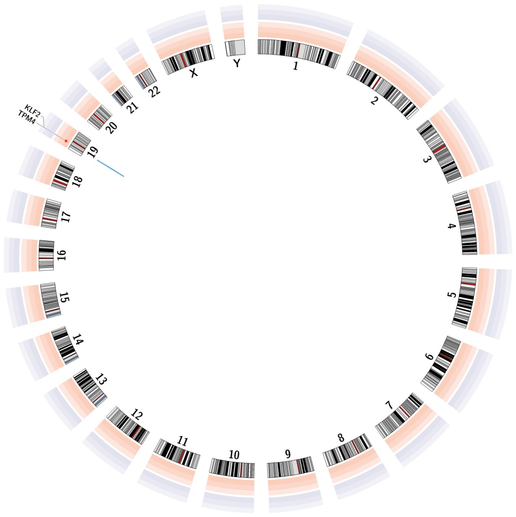

Figure 15 | Intersected fusion candidates

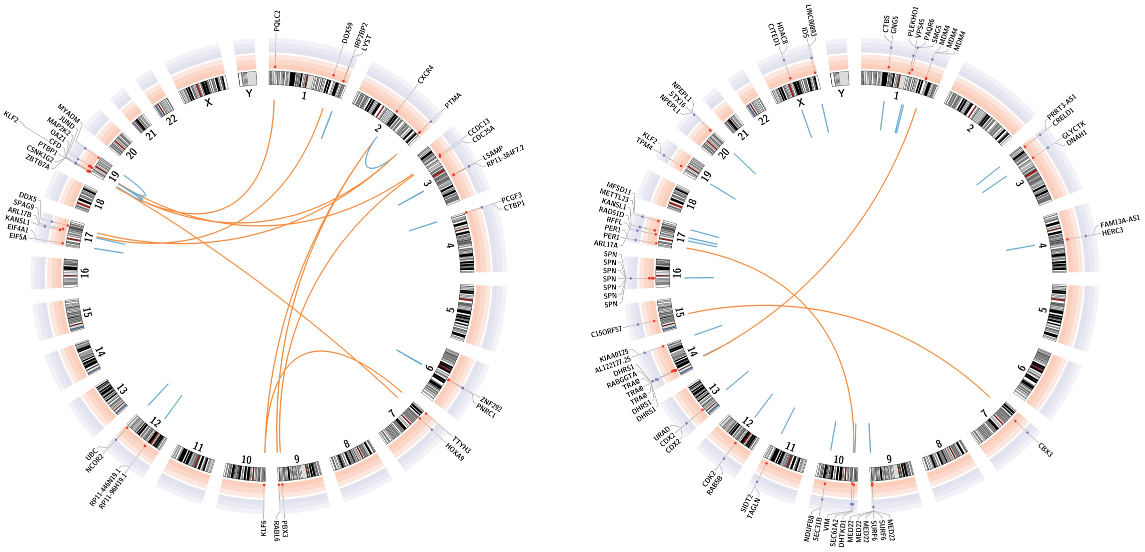

Figure 16 | STAR-Fusion (left) and FusionCatcher (right) fusion candidates

Table 29 | Top 20 intersected fusion candidates

Fusion name	Fusion type	Junction reads count	Spanning fragments count
TPM4--KLF2	ONLY_REF_SPLICE	6	0

ITD/PTD Detection Results

FLT3 ITD

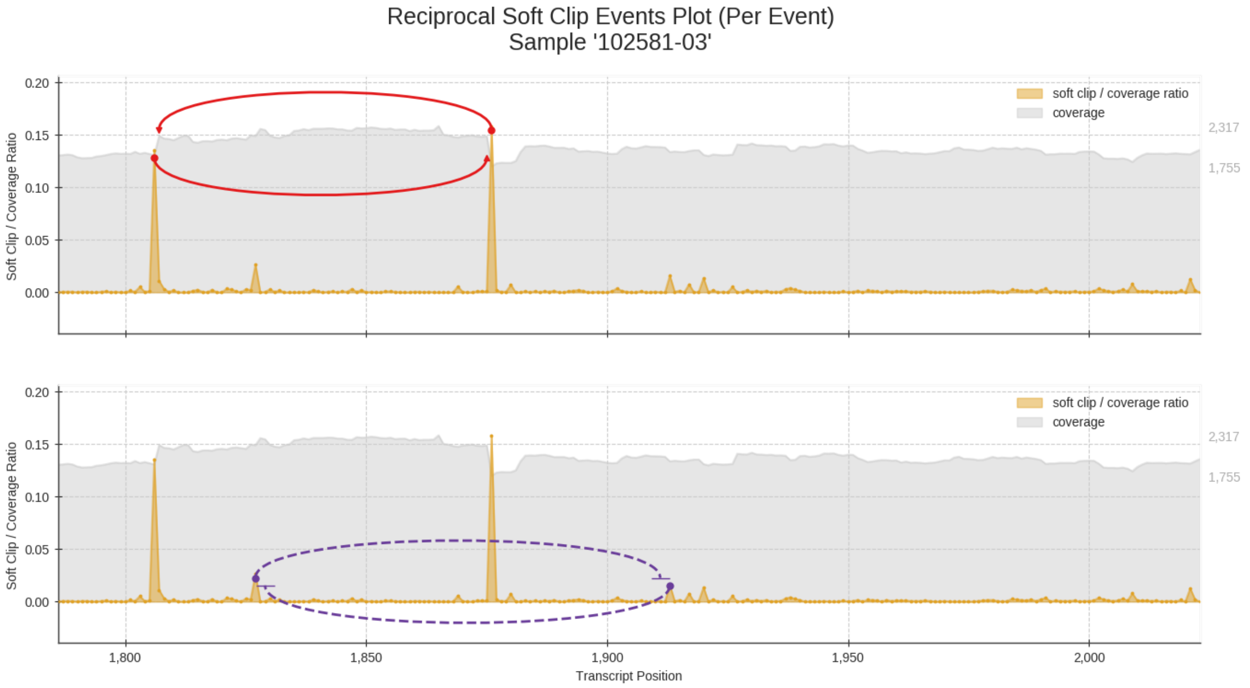

Figure 17 | FLT3 ITD detection results

KMT2A PTD

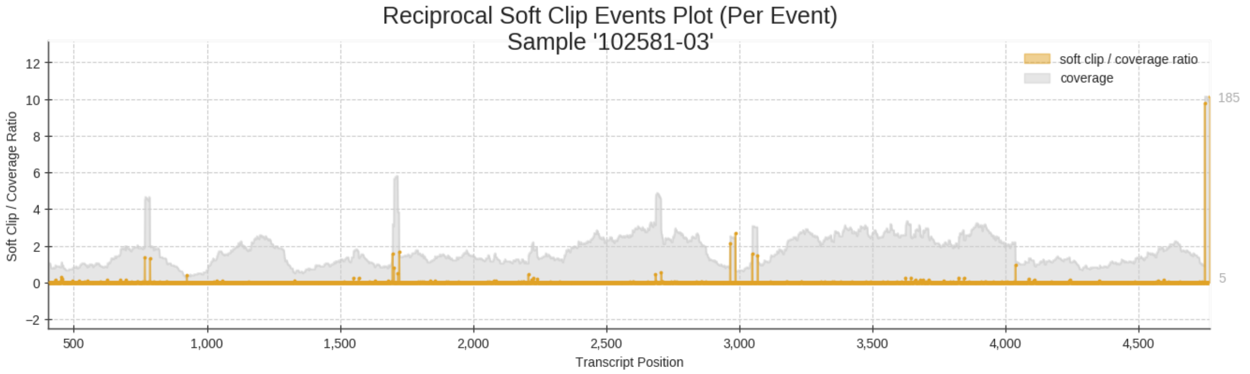

Figure 18 | KMT2A PTD detection results

Overexpression Analysis Results

Table 30 | Selected exon ratio values

Exon	Base count	Divisor gene	Divisor base count	Ratio	Above threshold
MECOM:169146722-169147734	0	HMBS	217,863	0	no

About

Hamlet is developed jointly by the Hematology Department and Human Genetics Department of Leiden University Medical Center. It is written using the Snakemake workflow system (v4.0). The source code can be accessed here.

Front cover image taken and modified from The Anatomy & Physiology Book published on OpenStax.
